# Role of CD14+ monocyte-derived oxidised mitochondrial DNA in the inflammatory interferon type 1 signature in juvenile dermatomyositis

**DOI:** 10.1136/ard-2022-223469

**Published:** 2022-12-23

**Authors:** Meredyth G Ll Wilkinson, Dale Moulding, Thomas C R McDonnell, Michael Orford, Chris Wincup, Joanna Y J Ting, Georg W Otto, Restuadi Restuadi, Daniel Kelberman, Charalampia Papadopoulou, Sergi Castellano, Simon Eaton, Claire T Deakin, Elizabeth C Rosser, Lucy R Wedderburn

**Affiliations:** 1 Infection, Immunity and Inflammation Research and Teaching Department, UCL Great Ormond Street Institute of Child Health, London, UK; 2 Centre for Adolescent Rheumatology Versus Arthritis at UCL UCLH and GOSH, UCL, London, UK; 3 NIHR Biomedical Research Centre, Great Ormond Street Hospital, London, UK; 4 Developmental Biology and Cancer Research & Teaching Department, UCL Great Ormond Street Institute of Child Health, London, UK; 5 Centre for Rheumatology Research, Division of Medicine, University College London, London, UK; 6 Experimental and Personalised Medicine, Genetics and Genomic Medicine, UCL Great Ormond Street Institute of Child Health, London, UK; 7 Genetics and Genomic Medicine Research & Teaching Department, UCL Great Ormond Street Institute of Child Health, London, UK; 8 Rheumatology, Great Ormond Street Hospital NHS Trust, London, UK

**Keywords:** Dermatomyositis, Autoimmune Diseases, Inflammation

## Abstract

**Objectives:**

To define the host mechanisms contributing to the pathological interferon (IFN) type 1 signature in Juvenile dermatomyositis (JDM).

**Methods:**

RNA-sequencing was performed on CD4^+^, CD8^+^, CD14^+^ and CD19^+^ cells sorted from pretreatment and on-treatment JDM (pretreatment n=10, on-treatment n=11) and age/sex-matched child healthy-control (CHC n=4) peripheral blood mononuclear cell (PBMC). Mitochondrial morphology and superoxide were assessed by fluorescence microscopy, cellular metabolism by ^13^C glucose uptake assays, and oxidised mitochondrial DNA (oxmtDNA) content by dot-blot. Healthy-control PBMC and JDM pretreatment PBMC were cultured with IFN-α, oxmtDNA, cGAS-inhibitor, TLR-9 antagonist and/or *n*-acetyl cysteine (NAC). IFN-stimulated gene (ISGs) expression was measured by qPCR. Total numbers of patient and controls for functional experiments, JDM n=82, total CHC n=35.

**Results:**

Dysregulated mitochondrial-associated gene expression correlated with increased ISG expression in JDM CD14+ monocytes. Altered mitochondrial-associated gene expression was paralleled by altered mitochondrial biology, including ‘megamitochondria’, cellular metabolism and a decrease in gene expression of superoxide dismutase (*SOD*)1. This was associated with enhanced production of oxidised mitochondrial (oxmt)DNA. OxmtDNA induced ISG expression in healthy PBMC, which was blocked by targeting oxidative stress and intracellular nucleic acid sensing pathways. Complementary experiments showed that, under in vitro experimental conditions, targeting these pathways via the antioxidant drug NAC, TLR9 antagonist and to a lesser extent cGAS-inhibitor, suppressed ISG expression in pretreatment JDM PBMC.

**Conclusions:**

These results describe a novel pathway where altered mitochondrial biology in JDM CD14+ monocytes lead to oxmtDNA production and stimulates ISG expression. Targeting this pathway has therapeutical potential in JDM and other IFN type 1-driven autoimmune diseases.

**WHAT IS ALREADY KNOWN ON THIS TOPIC**

- Juvenile Dermatomyositis (JDM) has a well-defined upregulated IFN type I signature in peripheral blood and at the inflammatory tissue sites, muscle and skin, but little is known about mechanisms upstream of this IFN type 1 signature.

**WHAT THIS STUDY ADDS**

- Dysregulated mitochondrial gene expression is associated with IFN type 1 upregulation in JDM.
- Mitochondrial morphology and superoxide regulation are abnormal in JDM CD14+ monocytes, leading to increased oxidised mitochondrial DNA (oxmtDNA).
- Targetting oxidative stress and intracellular nucleic acid sensing pathways suppresses both the ISG upregulation following culture of healthy control peripheral blood mononuclear cell with oxmtDNA and the ISG signature in cells from pretreatment JDM patients.

**HOW THIS STUDY MIGHT AFFECT RESEARCH, PRACTICE OR POLICY**

- These data suggest that therapeutically targeting abnormal mitochondrial biology and oxmtDNA production may suppress pathological IFN type 1 signatures.

## Introduction

Juvenile dermatomyositis (JDM) is a rare systemic autoimmune disease with a median age of onset of ~7 years and incidence of 2–4 cases per million/year.^1 2^ Symptoms include proximal muscle weakness and characteristic skin changes.^3^ Currently, the proposed disease pathology includes complement-mediated vasculopathy and autoimmune muscle damage.^4^ Not all patients respond to first-line treatments, prednisolone and methotrexate, and patients can develop treatment side effects and disease complications.^5^ There is as yet no evidence-based biomarker to indicate treatment response. In severe cases complications can lead to myositis-related mortality.^2 6^ There is a significant unmet need to develop more effective targeted treatments by exploring novel underlying pathogenic mechanisms.

One proposed mechanism is interferon (IFN)-driven pathogenesis. An upregulation of IFN type 1 signature has been found in JDM, detected at both gene expression and protein level in peripheral blood and tissue.^7–9^ Currently little is known about mechanisms up-stream of the IFN type 1 signature in JDM.^10^ Evidence suggests that mitochondrial dysfunction may activate IFN type 1 production in the absence of a pathogen.^11^ The innate immune system can recognise mitochondrial (mt)DNA as an immunogenic damage-associated molecular pattern and trigger a proinflammatory response^12^; injecting mtDNA in vivo triggers both local and systemic inflammation.^13 14^ This is due to structural features shared between bacterial DNA and mtDNA which include a circular genome and a high frequency of unmethylated CpG dinucleotide repeats.^15^ Several mechanisms have been highlighted as potential drivers of mtDNA-mediated inflammation. These include ligation of endosomal Toll-like receptor 9 (TLR9) on sensing of hypomethylated DNA with CpG motifs and the cytosolic cGAS-STING (protein cyclic GMP-AMP synthase stimulator of IFN genes) pathway.^16 17^

Reactive oxygen species (ROS) damage mtDNA, which if not repaired can lead to defective complex I and III function leading to increased superoxide production.^18^ This increased superoxide flux, in turn leads to genomic instability, metabolic stress and cellular injury. Once mtDNA is damaged it is susceptible to oxidation which can perpetuate the ROS cycle leading to inappropriate mitochondrial fission and fusion.^19^ This causes changes in mitochondrial morphology such as fragmentation and enlarged mitochondrial networks (mega mitochondria).^18^ Emerging evidence from IFN type 1-driven autoimmune diseases such as systemic lupus erythematosus (SLE) and JDM, and rare monogenic type 1 interferonopathies, suggest that there is a relationship between mitochondrial dysfunction, inappropriate production of ROS and IFN-driven disease pathology.^20–25^ However, the mechanisms underlying this association remain ill-defined.

In this article, we propose a novel pathogenic mechanism whereby CD14+monocyte-derived oxidised mtDNA induces IFN stimulatory gene (ISG) expression in the rare childhood autoimmune disease JDM. We identify that in JDM CD14+monocytes, the pathogenic interferon (IFN) type 1 signature is associated with dysregulation of mitochondrial-associated gene expression. Assessment of mitochondrial biology in JDM CD14+monocytes demonstrates that compared with age-matched healthy controls there is abnormal mitochondrial morphology, cellular metabolism and decreased gene expression of the antioxidant enzyme superoxide dismutase 1 (*SOD1*). This is associated with enhanced generation of mitochondrial superoxide and increased intracellular oxidised mitochondrial DNA (oxmtDNA). We delineated that oxmtDNA directly amplifies ISG expression, as oxmtDNA is able to induce the upregulation of ISG in healthy peripheral blood mononuclear cells (PBMCs) in vitro, which can be blocked by TLR-9 antagonism and the antioxidant drug, *N*-acetylcysteine (NAC). Interestingly, under these experimental conditions, TLR-9 antagonism and NAC, and to a lesser extent cGAS-STING inhibition, can also suppress ISG expression in PBMC from treatment naïve (pretreatment) JDM patients in vitro. This suggests an important role for nucleic acid sensing and oxidative stress in pathological IFN type 1 signatures in JDM. Collectively, these data identify abnormal mitochondrial biology and oxmtDNA as potential targets to therapeutically modulate downstream effects of IFN type 1 in multiple autoimmune diseases.

## Patients, materials and methods

See online supplemental material 1 and methods.

10.1136/ard-2022-223469.supp6 Supplementary data

## Results

### A dysregulated mitochondrial gene signature characterises CD14+ monocytes from JDM patients

**Figure 1:**
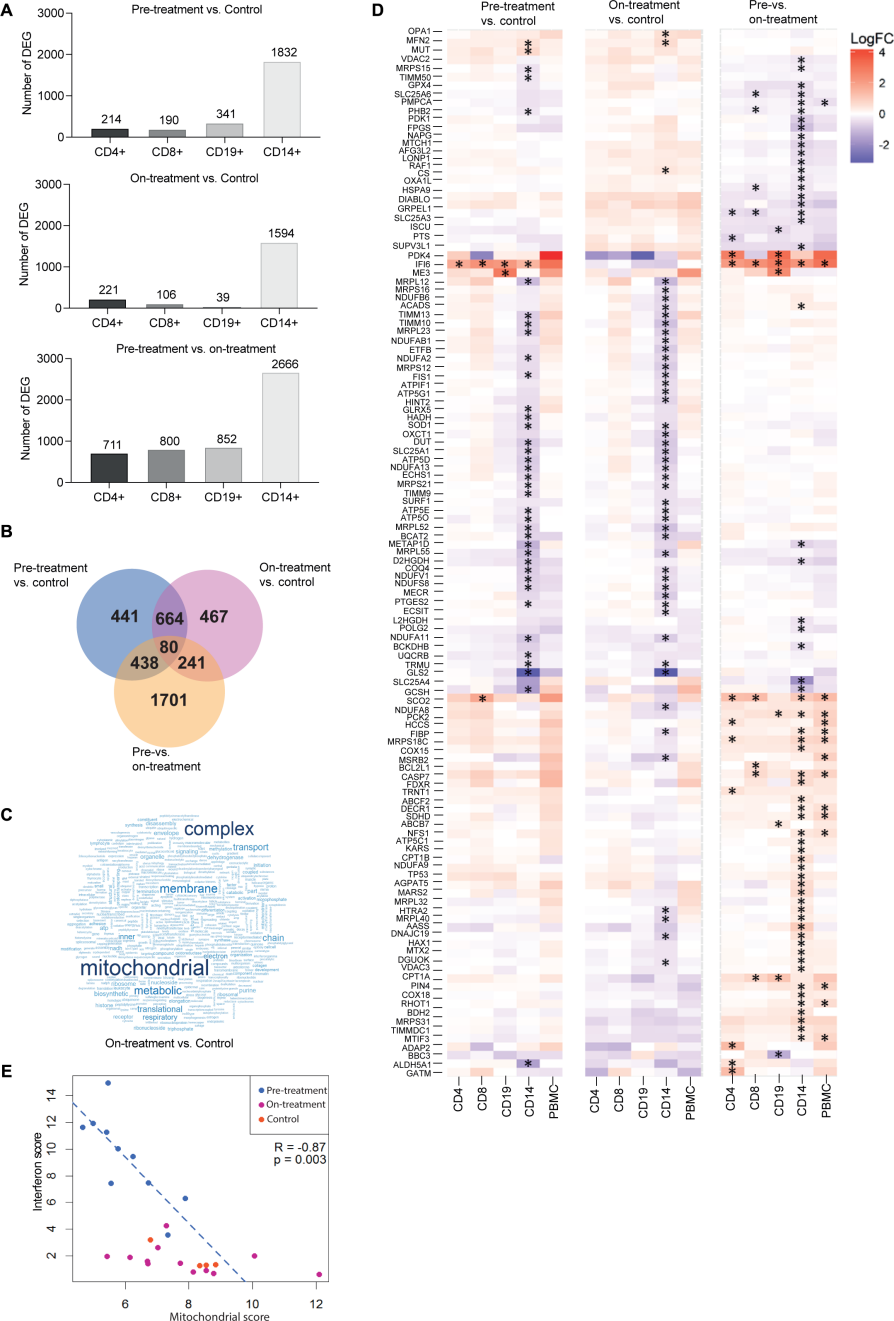
A dysregulated mitochondrial gene signature characterises CD14+ monocytes from JDM patients. Transcriptomic analysis of CD4+, CD8+, CD19+ and CD14+ cells from JDM patients taken pretreatment (n=10) and ~12 months on-treatment (n=11), and age-matched healthy controls (controls, n=4). (A) Numbers of differentially expressed genes (DEG) within each cell type between JDM pretreatment versus control, on-treatment versus control and pretreatment versus on-treatment. (B) Venn diagram showing the overlap of significantly DEG from CD14+ monocytes between JDM pretreatment versus control, on-treatment versus control and pretreatment versus on-treatment. (C) Word cloud representation of gene ontology analysis of CD14+ monocytes in JDM patients on-treatment compared with controls. (D) Heatmap representing overexpression and underexpression of genes within the ‘mitochondrion’ gene ontology (GO) term. Asterisk indicate significant differential expression. (E) Negative correlation of IFN type 1 score (15 known IFN type 1—stimulated genes) with all 13 mitochondrially encoded genes (protein coding); analysis in JDM patients pretreatment and on-treatment compared with controls. IFN scores against mitochondrial scores are displayed for all samples, but Spearman correlation coefficient and p value shown are for pretreatment JDM samples. JDM, juvenile dermatomyositis.

To define possible mechanisms upstream of the strong IFN type 1 signature and identify a transcriptional signature associated with immune dysfunction in JDM, we performed gene expression profiling on isolated CD4+T cells, CD8+T cells, CD19+B cells and CD14+monocytes from PBMC collected from JDM patients naïve to treatment (pre-treatment), at ~12 months on-treatment and age-matched controls (For demographics see online supplemental table 4). Transcriptional analysis showed significant differences in both pretreatment, and on-treatment JDM patients compared with controls and validated a strong IFN type 1 signature across all cell-types, which was largely normalised by treatment (online supplemental figure 1). CD14+monocytes had markedly more differentially expressed genes (DEG) than all other cell types with 1832 DEG in pretreatment JDM compared with controls, 1594 DEG in on-treatment JDM compared with controls, and 2666 DEG in pretreatment compared with on-treatment JDM (figure 1A and online supplemental tables 1–3). This was not due to differences in the total transcript levels detected in each cell type (n=16 751, data not shown). Notably, there were 744 (664+80) shared DEG in both pretreatment and on-treatment JDM compared with controls (figure 1B). This would suggest that the expression of these 744 genes in JDM CD14+monocytes was not normalised with current treatment strategies.

10.1136/ard-2022-223469.supp5 Supplementary data

10.1136/ard-2022-223469.supp1 Supplementary data

10.1136/ard-2022-223469.supp2 Supplementary data

10.1136/ard-2022-223469.supp3 Supplementary data

10.1136/ard-2022-223469.supp4 Supplementary data

To further explore the functional nature of the pathways that remained dysregulated in JDM CD14+monocytes after treatment, we performed gene ontology analysis of the 1594 DEG from JDM patients on treatment compared with controls. This showed a significant differential expression of terms related to mitochondrial function in CD14+monocytes from JDM on-treatment compared with controls (figure 1C). Further analysis of the mitochondrion GO term, identified decreased expression of mitochondrially associated genes, this included 6 mitochondrial-coded genes and 59 nuclear-encoded genes for mitochondrial proteins. In contrast, CD4+Tcells, CD8+T cells and CD19+B cells did not show differential expression in these genes in pretreatment or on-treatment JDM compared with controls (figure 1D left and middle panels), with the exception of the IFN-stimulated gene *IFI6* which is overexpressed in all cell types.

Given existing evidence that mitochondrial dysfunction may activate IFN type 1 production in the absence of a pathogen,^11^ we next investigated whether the expression of mitochondrial-encoded genes was associated with altered IFN type 1 gene expression in JDM CD14+monocytes. IFN type 1 gene expression was assessed using a set of ISG first used in studies of Aicardi-Goutières Syndrome, a prototypical monogenic type 1 interferonpathy.^26^ These data demonstrated reduced expression of mitochondrial genes was associated with increased expression of ISG in JDM pretreatment CD14+monocytes (figure 1E). No such association was seen in CD14+monocytes from patients on-treatment, as IFN type 1 signature was largely normalised by treatment (figure 1E). These data suggest that there is a possible relationship between mitochondrial dysfunction and IFN type 1 in JDM patients, and that although current treatment strategies normalise IFN type 1 signatures, mitochondrial biology remains abnormal.

### Changes in mitochondrial morphology are associated with abnormal cellular metabolism in CD14+ monocytes in JDM patients

**Figure 2:**
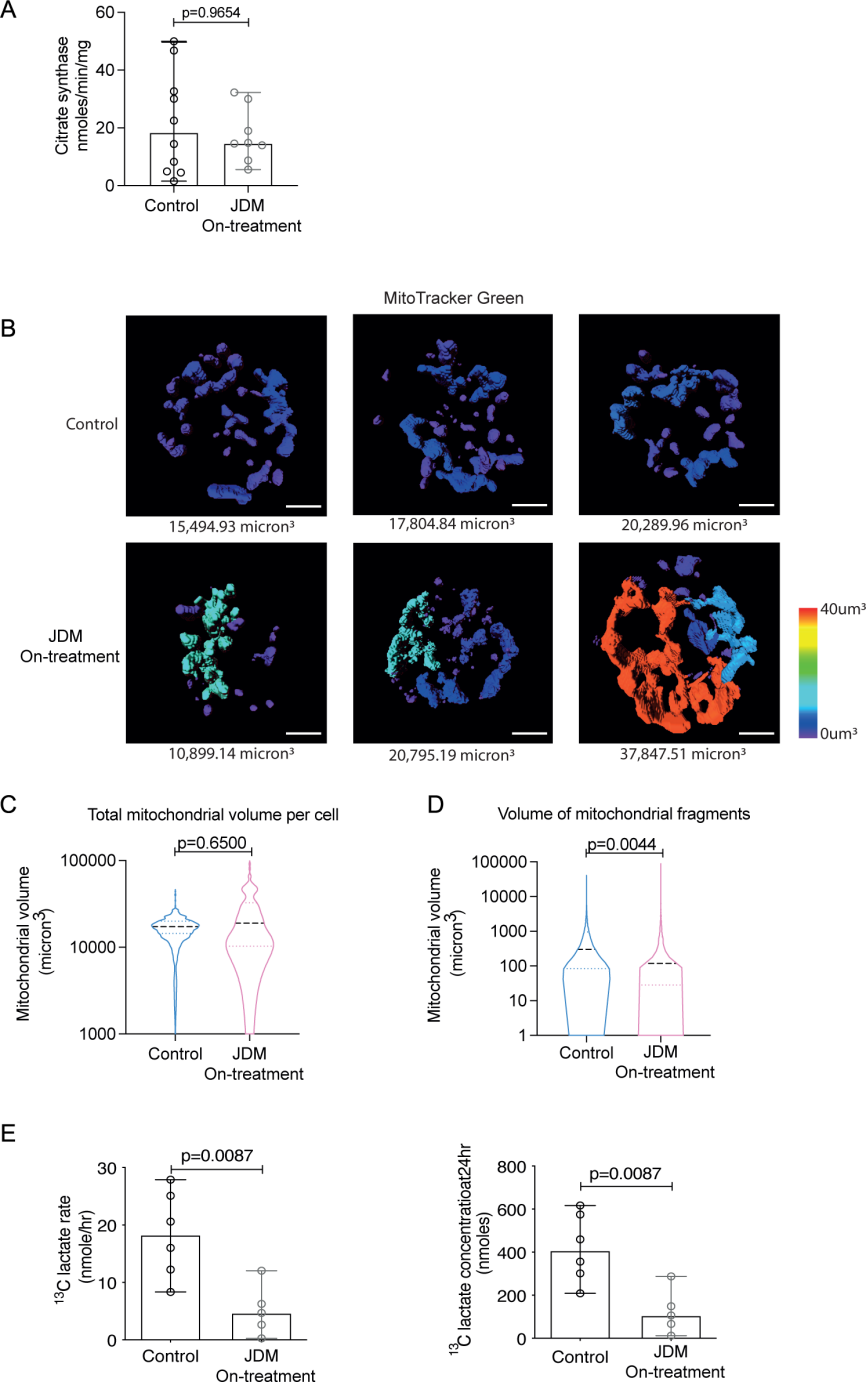
Changes in mitochondrial morphology are associated with abnormal cellular metabolism in CD14+ monocytes in JDM patients. (A) Bar chart shows citrate synthase rate normalised to protein content in JDM on-treatment (n=8) CD14+ monocytes compared with controls (n=10). (B) Representative images showing the range of mitochondrial fragment size in individual CD14+ monocytes from JDM on-treatment (n=6) and control (n=9). Images are representative for the lowest interquartile range (left), median (central) and highest IQR (right) of total mitochondrial volume per cell from control (top) and JDM (bottom) CD14+ monocytes. (C) Violin plot showing the distribution of total mitochondrial volume (µm^3^) per cell in JDM on-treatment CD14+ monocytes (n=6) compared with controls (n=9) (D) Violin plot showing the distribution of the individual mitochondrial fragment volume (µm^3^) from all CD14+ monocytes in JDM on-treatment (n=6) compared with controls (n=9). (E) Bar graphs show ^13^C labelled glucose metabolism rate (left) and final concentration (right) into lactate in CD14+ monocytes from JDM on-treatment (n=5) and controls (n=6). (A, E) Bar graphs: median with range shown. statistical analysis: (A, E) Non-parametric Mann-Whitney tests, p values. (C, D) Violin plots of distribution with median and IQR. Analysis of the effect of JDM compared with controls as a fixed effect in a linear mixed effects model, with a random effect included to account for individual-specific effects. P values were generated using nested ANOVA. ANOVA, analysis of variance; JDM, juvenile dermatomyositis.

We next assessed whether changes in mitochondrial gene expression were reflected in altered mitochondrial biology in JDM CD14+monocytes. To do this, we measured mitochondrial content using citrate synthase activity (a quantitative enzymatic marker for the presence of mitochondria)^27^ and visualised mitochondrial morphology using fluorescence microscopy. Although we identified no significant difference in citrate synthase activity (figure 2A), visualisation of mitochondria by fluorescent microscopy, using MitoTracker green-fluorescent stain, showed that mitochondrial morphology was markedly altered in JDM CD14+monocytes compared with controls. These data demonstrated that although, in keeping with the citrate synthase activity, there was no overall difference in the mitochondrial volume per cell (figure 2B, C), there was a greater range and variance in the size of the mitochondrial fragments themselves (figure 2B, D). Overall, the size of the mitochondrial fragments was significantly decreased in JDM CD14+monocytes suggesting the mitochondria were generally more fragmented compared with controls (figure 2B, C). However, in some JDM CD14+monocytes the wide variance in the size of mitochondrial fragments was depicted by the presence of abnormally enlarged mitochondrial networks or ‘megamitochondria’ (figure 2B and D). To investigate whether altered mitochondrial morphology was associated with altered cellular metabolic capacity we next assessed whether the ability of JDM CD14+monocytes to metabolise glucose was altered using a ^13^C glucose uptake assay. These data showed there was a significant reduction in the rate and final concentration of ^13^C lactate production (figure 2E), but no difference in the rate and final concentration of ^13^C CO_2_ in JDM CD14+ monocytes compared with controls (online supplemental figure 2A). These data demonstrate that glucose metabolism but not glucose oxidation is altered in these cells. Due to the fragility of mitochondria and altered cellular metabolic capacity post-thawing, these functional assays could only be carried out on fresh blood samples. In addition, due to the rarity of JDM, these fresh samples could only be obtained from on-treatment JDM patients for these assays. Differences in the metabolic capacity of the cell was not due to a decrease in glucose transport or potentially uptake as there was no difference in the expression of glucose transporter 1 (GLUT1), a significant increase in the expression of glucose transporter 4 (GLUT4) and an increase in glucose uptake measured using the fluorescent glucose analogue 2-NBDG (2-(*N*-(7-Nitrobenz-2-oxa-1,3-diazol-4-yl)Amino)-2-Deoxyglucose) in JDM CD14+monocytes on-treatment compared with controls (online supplemental figure 2B–D). Given previous reports that the endocytic GTPase protein Rab4 has been shown to regulate GLUT4 expression,^28^ we next checked the gene expression of *RAB4A* in the transcriptional dataset; however, there was no difference in the expression of *RAB4A* in JDM on-treatment compared with control (data not shown).

### Transcriptional data reveals changes to oxidative phosphorylation and superoxide regulation in JDM CD14+ monocytes

**Figure 3:**
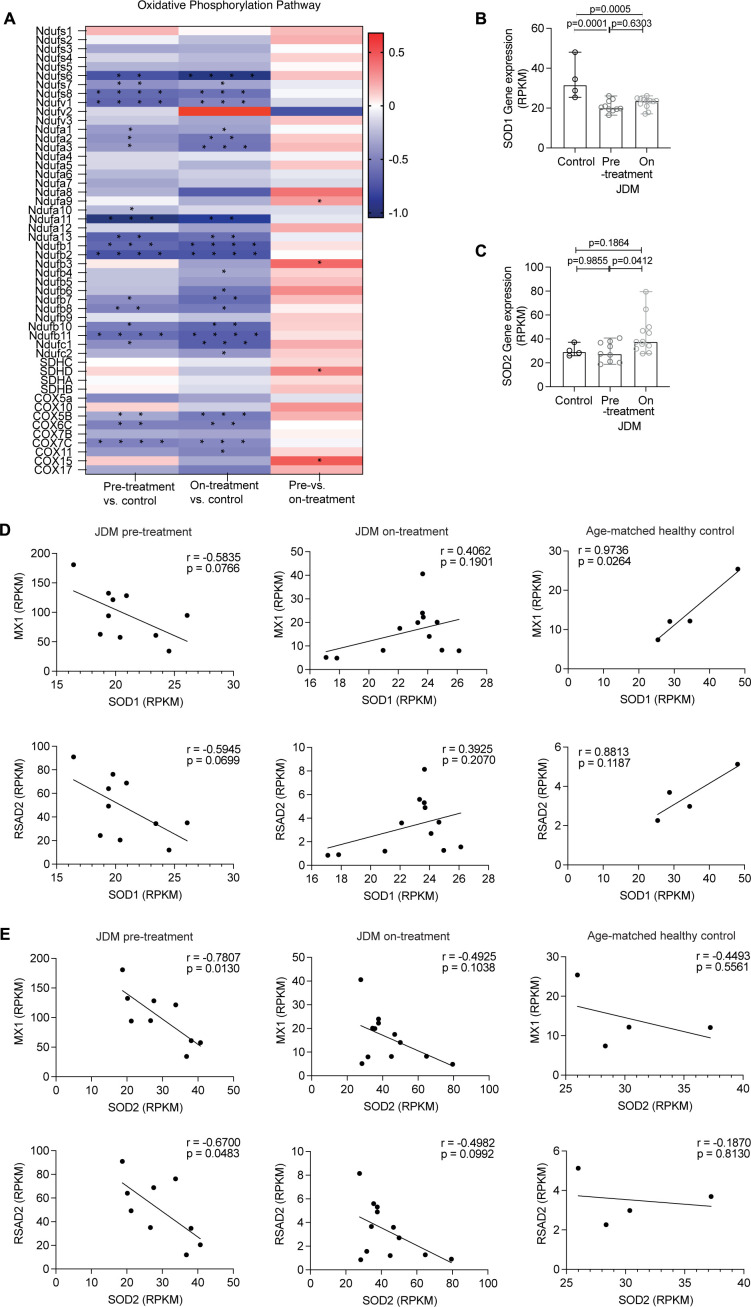
Transcriptional data reveal changes to oxidative phosphorylation and superoxide regulation in JDM CD14+monocytes. (A) Heatmap showing fold change (FC) of differentially expressed genes (DEG) from KEGG oxidative phosphorylation pathway in CD14+ monocytes from JDM patients taken pretreatment (n=10) and ~12 months on-treatment (n=11), and controls (n=4). Red is upregulated (positive Fc) and blue is downregulated (negative Fc), stars represent adjusted p values (*p<0.05, **p<0.01, ***p<0.001, ****p<0.0001). Gene expression analysis of CD14+ monocytes from JDM patients taken pretreatment (n=10) and ~12 months on-treatment (n=11), and age-matched healthy (controls (n=4)). Reads per kilobase pair of transcript (RPKM) gene expression of (B) *SOD1* and (C) *SOD2*. Correlation plots of RPKM gene expression of *SOD1* (D) and *SOD2* (E), to *MX1* and *RSAD2* (top to bottom) in CD14+ monocytes from JDM patients taken pretreatment (n=10) (left) and 12 months on-treatment (n=11) (middle) and controls (n=4) (right). All bar graphs: median with range shown. Statistical analysis: (B, C) Non-parametric Kruskal-Wallis test with Dunn’s multiple comparisons, adjusted p values. (D–E) R and p values calculated by Pearson correlation. JDM, juvenile dermatomyositis.

Aberrant changes to mitochondrial morphology and cellular metabolism are key features of mitochondrial stress and are associated with decreased oxidative phosphorylation.^18 19^ Assessment of the KEGG pathway ‘oxidative phosphorylation’ in the RNAseq data-set demonstrated a global downregulation of the expression of genes in this pathway in both pretreatment and on-treatment JDM CD14+monocytes compared with controls (figure 3A), with limited differences between pretreatment and on-treatment JDM CD14+monocytes (figure 3A). To prevent oxidative stress, changes to mitochondrial morphology and cellular metabolism lead to upregulation of compensatory mechanisms that regulate the production of superoxide.^29^ Interrogation of the transcriptomic data for DE genes involved in superoxide regulation demonstrated that the expression of the gene superoxide dismutase 1 (*SOD1*), which is localised in the cytosol and the mitochondrial intermembrane space and has an important role in protecting against mitochondrially-produced superoxide,^30^ was significantly decreased in both pretreatment and on-treatment JDM CD14+monocytes compared with controls (figure 3B). There was no difference in the gene expression of superoxide dismutase 2 (*SOD2*), which is localised in the mitochondrial matrix and has a role regulating intramitochondrial superoxide levels (figure 3C).^31^ Notably, the expression of *SOD1* and *SOD2* genes only negatively correlated with the up-regulated expression of ISGs *MX1* and *RSAD2* (representative ISGs) in CD14+monocytes from pretreatment JDM patients, but not on-treatment JDM patients and controls which do not have upregulated ISGs, suggesting a possible association between oxidative stress in JDM CD14+monocytes and the IFN type 1 gene signature (figure 3D, E, online supplemental figure 3A).

### Enhanced oxidative stress leads to increased oxmtDNA content in JDM CD14+ monocytes

**Figure 4:**
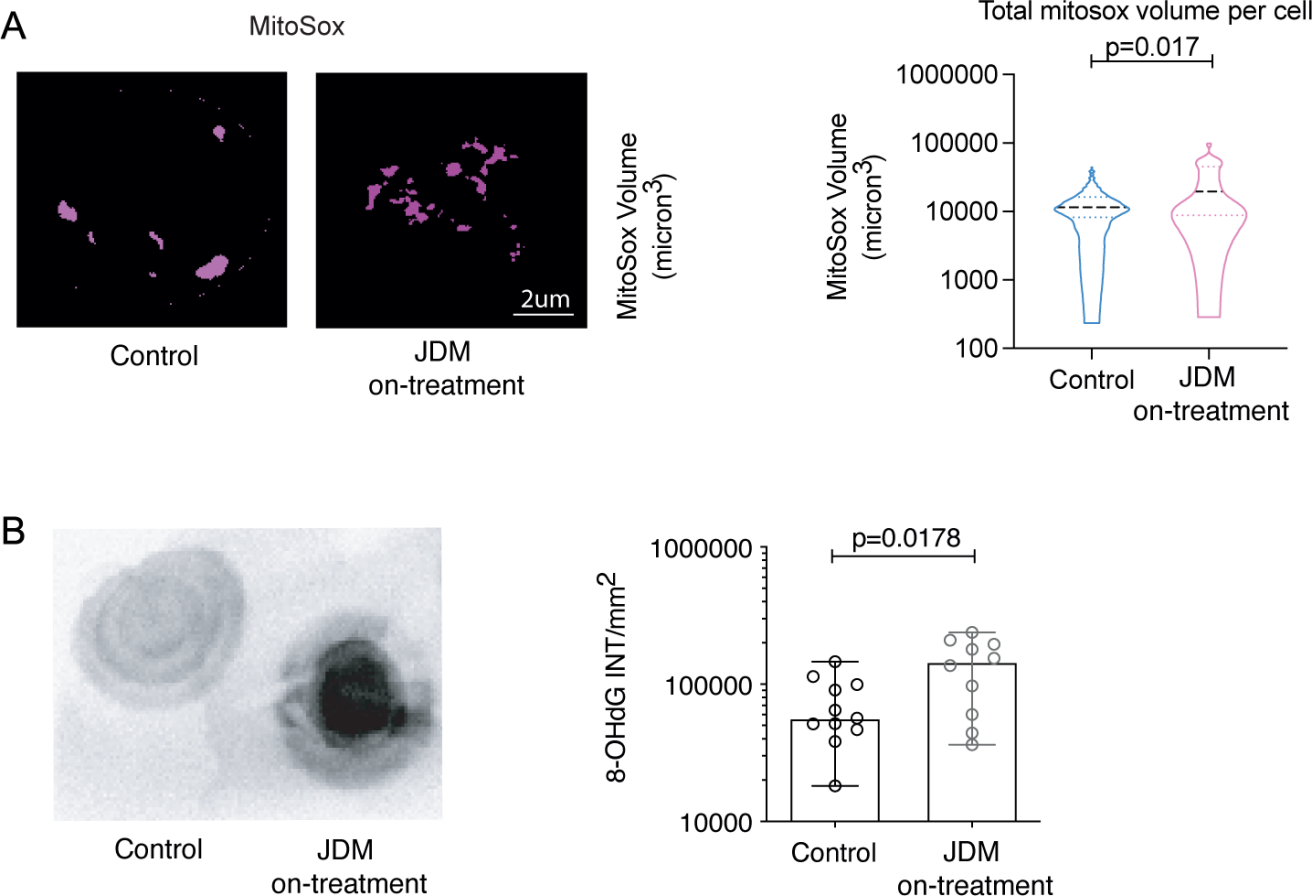
There is enhanced oxidative stress in JDM CD14+ monocytes, which is associated with increased oxidised mitochondrial DNA (oxmtDNA). (A) representative images (left), and violin plot (right) showing MitoSox volume (µm^3^) per cell in CD14+ monocytes from JDM on-treatment (n=6) compared with controls (n=9). (B) Representative densitometry plots (left) and bar graph (right) of 8-hydroxy-2'-deoxyguanosine (8-OHdG) concentration from isolated CD14+ monocyte-derived mitochondria from JDM on-treatment patients (n=10) and controls (n=11). All bar graphs: median with range shown. (A) Violin plots of distribution with median and IQR. Analysis of the effect of JDM versus healthy control as a fixed effect in a linear mixed effects model, with a random effect included to account for individual-specific effects. P values were generated using nested ANOVA. (B) Non-parametric Mann-Whitney tests, p values. ANOVA, analysis of variance; JDM, juvenile dermatomyositis.

To assess oxidative stress in CD14+monocytes, we next measured the abundance and location of mitochondrial superoxide using MitoSox, which fluoresces when oxidised by superoxide specifically in mitochondria. These data showed a significant increase in the amount of superoxide as measured by an increase in MitoSox in JDM CD14+monocytes compared with controls (figure 4A). Dysregulation in superoxide has previously been shown to increase intracellular oxidised mtDNA (oxmtDNA), which is thought to be pathogenic in SLE.^20^ To test whether superoxide dysregulation was associated with an increase in intracellular oxmtDNA in JDM CD14+monocytes, we used dot blot with densitometry to quantify 8-Hydroxy-2'-deoxyguanosine (8-OhDG) from mitochondria isolated from CD14+monocytes. These data showed a significant increase in oxmtDNA from JDM CD14+monocytes compared with controls (figure 4B). Due to requirement for fresh samples, these experiments could only be carried out on samples from JDM patients on-treatment.

To investigate whether release of oxmtDNA into the periphery could contribute to the systemic pathology observed in JDM patients and to assess this signature in pretreatment samples, we finally measured the levels of cell-free circulating mtDNA in plasma of JDM and controls (online supplemental figure 3B). JDM plasma mtDNA levels were increased compared with controls (online supplemental figure 3C). In JDM pretreatment patients with both transcriptional data and data assessing mtDNA levels in plasma (n=3), the levels of cell-free mtDNA positively correlated with *MX1* and *RSAD2* expression (online supplemental figure 3D). These data further support the notion that dysregulated mitochondrial biology is associated with IFN type 1 in patients with JDM.

### Oxidised mtDNA modulates interferon stimulated gene (ISG) expression in vitro

**Figure 5:**
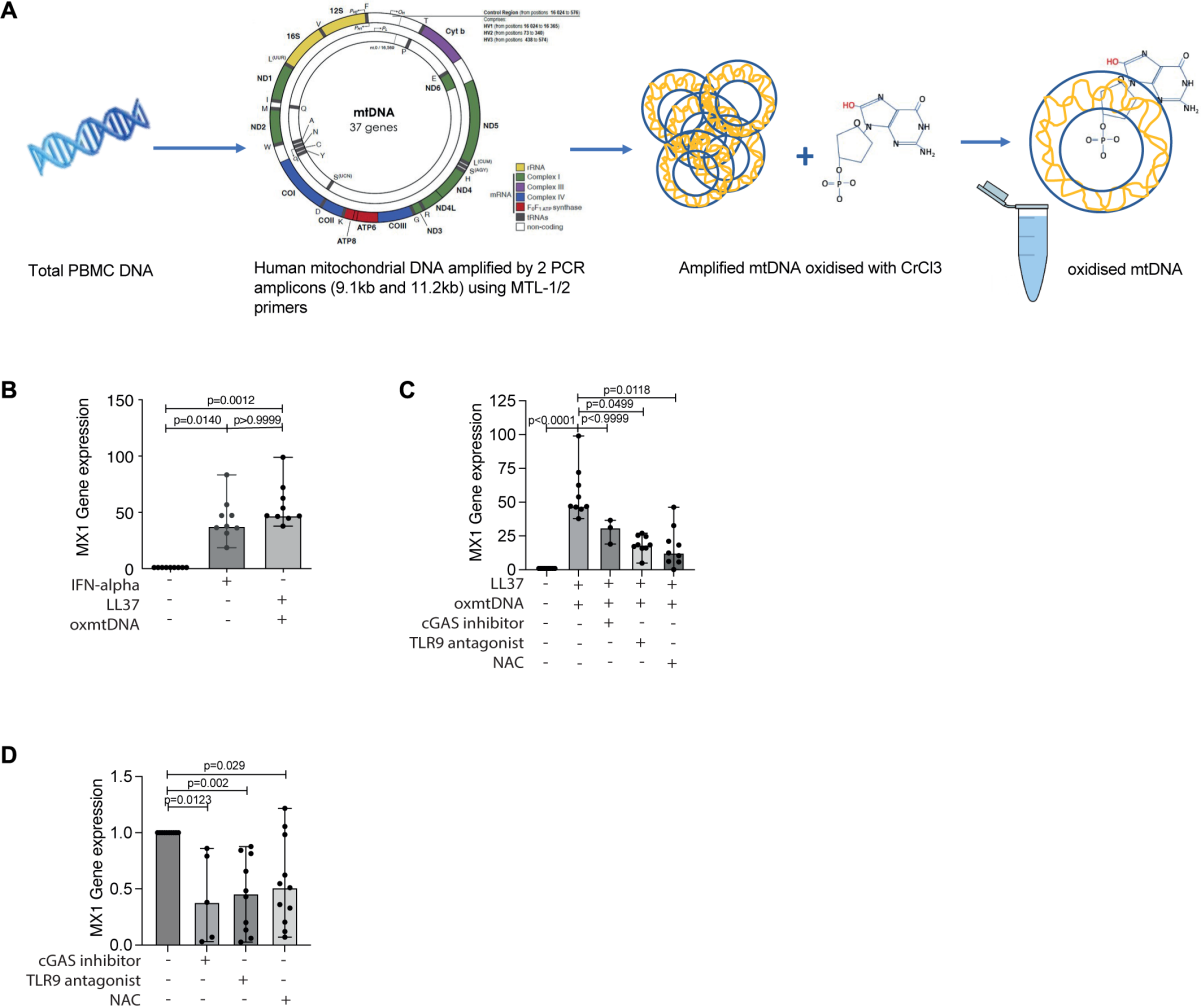
Oxidised mtDNA induces ISGs in vitro which can be blocked by treatment with TLR-9 antagonist and the antioxidant *N*-acetylcysteine (NAC). (A) Schematic shows methodology for generation of oxidised mtDNA (oxmtDNA). (B) Bar graphs show relative expression of IFN stimulated gene (ISG) Mx1 in control PBMC (n=12) incubated with IFN-alpha, oxmtDNA (with LL-37) or no stimuli (unstim). (C) Bar graphs show relative expression of ISG *MX1* in control PBMC incubated with no stimuli, oxmtDNA (+LL-37) (n=12) with or without cGAS inhibitor (n=6), TLR-9 antagonist (ODN TTAGGG(A151)) (n=11) or N-acetylcysteine (NAC) (n=11). (D) Bar graphs show gene expression of ISG *MX1* in PBMC from JDM patients pretreatment cultured with or without cGAS inhibitor (n=5), TLR-9 antagonist (n=10), NAC (n=11) or in medium alone (untreated) (n=11). All bar graphs: median with range shown. Statistical analysis: (B–D) Non-parametric Kruskal-Wallis test with Dunn’s multiple comparisons, adjusted p values. JDM, juvenile dermatomyositis; PBMC, peripheral blood mononuclear cell.

Given these findings suggest a relationship between IFN type 1, superoxide dysregulation and an increase in oxmtDNA within JDM CD14+monocytes compared with controls, we next tested the hypothesis that oxmtDNA can induce expression of ISG. We generated human oxmtDNA using a novel in house method (figure 5A) and then cultured control PBMC from healthy individuals in the presence of IFN-α, non-oxidised mtDNA, oxmtDNA or no stimuli. Both forms of mtDNA were cultured with the cationic peptide LL37 to aid cellular uptake of self-DNA.^32^ These results showed a comparable increase in *MX1* and *RSAD2* gene expression for both IFN-α and oxmtDNA (figure 5B and online supplemental figure 4B). Of note, non-oxidised mtDNA in vitro did not lead to upregulation of *MX1* gene expression suggesting a critical role for mtDNA oxidiation to amplify IFN type 1 gene expression (online supplemental figure 4A).

Pathways downstream of mtDNA-mediated inflammation include activation of TLR9 and the cGAS-STING pathway.^16 17^ To interrogate the mechanistic pathways by which oxmtDNA increases the expression of ISG, we next investigated whether cGAS-inhibitor (G-140), TLR-9 antagonist (ODN TTAGGG (A151)) and the antioxidant drug *N*-acetylcysteine (NAC) could block upregulation of ISG expression by oxmtDNA. Healthy control PBMC were prestimulated with oxmtDNA and then cultured in the presence or absence of cGAS-inhibitor, TLR-9 antagonist or NAC. Under these experimental conditions, TLR-9 antagonist and NAC, but not cGAS-inhibitor, significantly downregulated *MX1* gene expression (figure 5C), while these drugs did not significantly alter the expression of RSAD2 (online supplemental figure 4D). Finally, to investigate the therapeutic potential of targeting these pathways to suppress ISG expression in JDM, we cultured PBMC from pretreatment JDM with cGAS-inhibitor, TLR-9 antagonist, NAC or medium alone. TLR-9 antagonist and NAC significantly down-regulated both *MX1* and *RSAD2* gene expression, while cGAS-inhibition only downregulated *MX1* (figure 5D and online supplemental figure 4D). Collectively, these data provide evidence that targeting broad host mechanisms upstream of oxmtDNA ISG upregulation may be efficacious for the treatment of JDM.

## Discussion

A growing body of literature has underlined a central role for dysregulated IFN type 1 production in the immunopathology of multiple autoimmune rheumatic diseases (ARDs).^7 8 33 34^ Accordingly, study of upstream mechanisms contributing to upregulation of ISGs is likely to identify novel therapeutic targets for multiple ARDs. To date, most studies interrogating dysfunctional host responses leading to enhanced IFN production in autoimmunity have focused on SLE.^35 36^ There remains a paucity of mechanistic data from other IFN-driven ARDs.^21 37 38^ Here, using bulk RNA-sequencing of sorted CD4+T cells, CD8+T cells, CD19+B cells and CD14+monocytes from PBMC, we corroborate previous studies reporting a strong IFN type 1 signature in JDM pretreatment.^7–9^ We also demonstrate that this signature is associated with altered expression of mitochondria associated genes in CD14+monocytes—a gene signature which is not normalised by current treatment strategies. We further report that mitochondrial morphology and biology is altered in JDM CD14+monocytes compared with controls, which leads to enhanced production of oxmtDNA. oxmtDNA, in turn, upregulate ISGs in vitro. Based on these findings, we propose the hypothesis that in JDM, CD14+monocytes act as a source of oxmtDNA, which contributes, at least in part, to the disease’s IFN-driven signature. Further exploration of this hypothesis is needed to understand whether targeting this pathogenic mechanism could be a novel treatment strategy in JDM and other disorders with the aim of suppressing interferonopathy and associated pathology.

This is not the first time that altered mitochondrial function has been linked with IFN production. Indeed, evidence from multiple autoimmune diseases, including JDM, demonstrate a relationship between mitochondrial pathways, and particularly mtDNA, to disease pathology and IFN-driven inflammation - although predominately in the context of neutrophil extracellular traps (NETs).^20 21 39^ In SLE, Caielli and collaborators showed that NETs contained high levels of oxidised mtDNA which up-regulated IFN type 1 in vitro as measured in plasmacytoid dendritic cells,^21^ while a study in JDM demonstrated that engulfment of calcium crystals in neutrophils infiltrating inflamed muscles, lead to cell death and production of NETs containing mtDNA.^39^ In our study, only oxmtDNA, but not mtDNA, was able to upregulate ISG expression suggesting a central pathogenic role for oxidative stress in IFN-driven diseases. This was corroborated by data showing that NAC a potent, clinically approved, anti-oxidant therapy, which decreases cellular levels of oxidised DNA,^40^ reduced ISG expression in pretreatment JDM PBMC. Pilot studies have demonstrated that NAC reduces disease activity in SLE.^41^ Although the ability of NAC to suppress disease activity in SLE was attributed to its ability to block mTOR activation in T cells,^41^ comparing the ability of NAC to suppress oxmtDNA production, ISG expression and disease activity in SLE and JDM could be an interesting focus of future studies. Future research will focus on uncovering the mechanism of action by which NAC reduces oxmtDNA-induced ISG expression, and more specifically what are the upstream mechanisms controlling oxidative stress in JDM. Possibilities include damage to the mitochondrial respiratory chain and altered function of complex I activity and/or changes to mitophagy and scavenging of ROS through alterations in pathways that control redox signalling.^42–44^ Notably, NAC has been previously shown to reduce oxidative stress by blocking mitochondrial oxygen consumption via inhibition of complex I of the electron transport chain^45^ and by inhibiting mitophagy and supporting mitochondrial biogenesis^46^ suggesting multiple possibilities for its mechanisms of action.

In this study, we have proposed that CD14+monocytes are the major source of oxmtDNA in JDM and monocytes have previously been implicated in JDM and our findings further strengthen their potential role.^47–49^ However, gene expression modules associated with mitochondrial dysfunction and IFN type 1 have also been identified in JDM bulk muscle, the inflammatory site of the disease.^23^ A study in adult dermatomyositis showed a correlation between mitochondrial dysfunction and IFN type 1, and using an experimental autoimmune mouse model identified that NAC prevented mitochondrial dysfunction, IFN type I transcript and muscle weakness.^50^ Studies have also demonstrated that single nucleotide polymorphisms or altered copy number within mtDNA are associated with the development of adult idiopathic inflammatory myopathies.^51^ These studies, taken together with the high mitochondrial content in skeletal muscle^52^ and the growing interest in understanding immune cell function within inflamed tissues,^53^ demonstrate that new research is needed to interrogate mitochondrial biology within biopsies from JDM muscle.

Sensing of intracellular DNA via activation of the cGAS-STING pathway and TLR9 have been previously shown to induce IFN production.^16 54^ In this study, under these experimental conditions, we found that while TLR9-antagonism but not cGAS inhibtion significantly downregulated oxmtDNA-induced ISG expression in healthy PBMC, both TLR9 antagonism and cGAS inhibition could suppress *MX1 (representative ISG*) in pretreatment JDM PBMC. Notably, TLR9 antagonism also suppressed the expression of a second ISG in JDM, *RSAD2*. Of note, TLR9 and cGAS-STING pathway sense intracellular DNA oligonucleotides within different cellular compartments.^54–56^ cGAS-STING pathways senses DNA within the cytoplasm, while TLR9 senses DNA within endosomes. The divergent effects of TLR9 antagonism versus cGAS-inhibition could be due to limitation in experimental conditions with the cationic peptide LL37^32^ preferentially facilitating uptake of oxmtDNA by endosomes rather than into the cytosolic compartment. Alternatively our results could also suggest a dominant role for TLR9, and therefore, endosomal oxmtDNA, in controlling ISG upregulation in JDM. Uptake of oxmtDNA in JDM within endosomes could be due to multiple mechanisms including uptake of oxmtDNA from the extracellular space^20 57^ or changes in mitophagy—recent studies demonstrate that endosomes and endosome-related protein actively participate in this process.^58^ Presently our data support the possible contribution of both these pathways. First, mtDNA levels are increased in JDM plasma compared with controls and there is a positive correlation between mtDNA levels in plasma and ISG expression in JDM CD14+monocytes. Second, JDM mitochondria in CD14+monocytes are significantly more fragmented than controls, with increased mitochondrial fragmentation being previously reported to be linked to enhanced mitochondrial fission and mitophagy.^59^ Our data also do not discount an important role for cytosolic release of oxmtDNA by mitochondria, which has been shown to be an important driver of innate immune activation in multiple experimental settings.^60–62^ Future studies are needed to investigate the synergistic and/or diverging roles of TLR9-activation versus the cGAS-STING pathway, and therefore, the relative contribution of endosomal versus cytosolically released oxmtDNA, in the pathology of JDM.

Our study is not without its limitations. Analyses have not been stratified based on the heterogeneous clinical features of JDM, which has been proposed as an umbrella diagnostic term for a group of diseases.^63^ Patients can present with an array of different symptoms, including muscle weakness, skin rashes, calcinosis, dysphagia, joint pain and interstitial lung disease (ILD). Myositis specific/associated autoantibody (MSA) subtypes have been associated with different disease phenotypes. Patients with anti-Mi2 autoantibodies present with a classic JDM characterised^64^ with proximal muscle weakness and skin rash, while patients with anti-MDA5 autoantibodies are more likely to have associated ILD and have milder muscle disease.^65^ Unfortunately, despite accessing samples from the large Juvenile Dermatomyositis Cohort and Biobank study (JDCBS), we were still underpowered to stratify our data based on MSA subtype. Another significant limitation of the study was the necessity of using fresh blood samples to assess mitochondrial biology. Mitochondria, and indeed monocytes, have been previously shown to be significantly affected by freeze-thaw cycles^66–68^ and using frozen samples would have significantly affected the accuracy of these functional experiments. Fresh samples were obtained from patients who were also having blood drawn as part of clinical care. This prevented any selection criteria being used and therefore patients were on-treatment with varying lengths of disease duration. For analyses where we could use frozen samples collected longitudinally for the JDCBS, we were able to include samples from JDM patients both pre and on-treatment adding significant leverage to this study. Due to the rarity of JDM and difficulty in obtaining fresh whole blood samples, for use on the same day in mechanistic studies, another significant limitation was that our study did not assess mitochondrial function, including oxidative stress and production of oxmtDNA, in neutrophils from JDM patients or healthy controls. This would be an important avenue of work in future studies due to the well-described association between mitochondrial dysfunction in neutrophil and ISG signatures in other IFN-driven autoimmune disorders such as SLE.^20–22^

**Figure 6:**
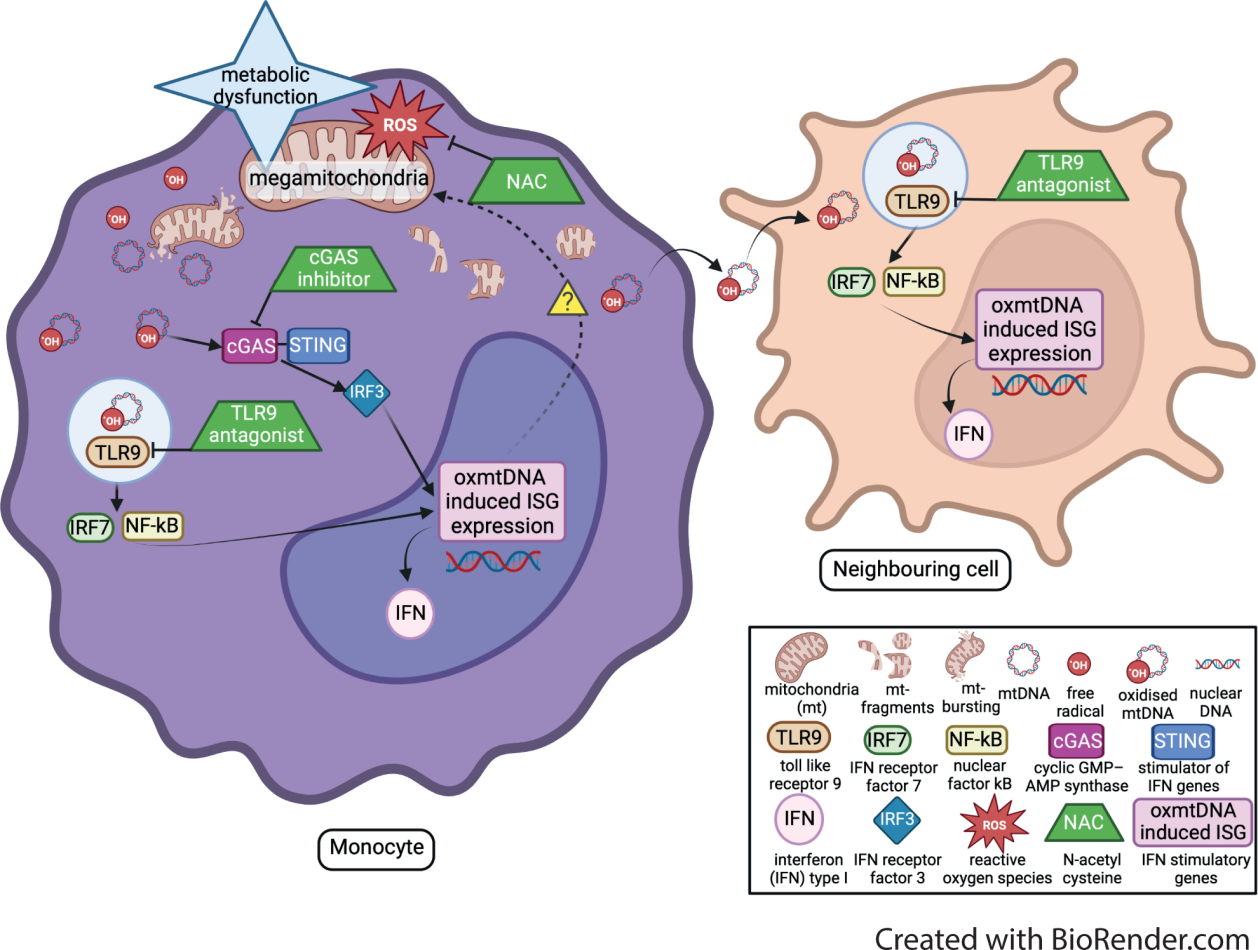
Schematic of the proposed mechanism. mitochondria are damaged by reactive oxygen species (ROS) and other mitochondrial stresses, leading to mitochondrial metabolic dysfunction. This dysfunction leads to fragmented mitochondria and the release of mitochondrial (MT) DNA. An increase of superoxide production leads to mtDNA becoming oxidised (ox). This oxmtDNA then activates the cGAS-STING and endosomic TLR9 pathways inducing the upregulation of IFN stimulatory gene (ISG) expression. Potential therapeutic agents (shown as green trapezium symbol), which can block different levels of the mechanism and down regulate oxmtDNA induced ISG expression. These therapeutic agents include the anti-oxidant NAC, which targets oxidative stress, cGAS inhibitor which targets the cGAS-STING pathway, and TLR9 anatagonist which blocks the TLR9 pathway.

Here, we have identified that altered mitochondrial morphology and biology in JDM CD14+monocytes leads to enhanced oxmtDNA production, which has the potential to drive aberrant ISG expression in this rare childhood disease (schematic in figure 6). We also identify two new potential therapeutic strategies for JDM—targeting nucleic acid sensing and oxidative stress—which suppress ISG expression in vitro. New translational studies are now needed to develop pipelines to take these potential therapeutic agents (cGAS inhibitor, TLR9 antagonist and NAC) closer to clinical trial testing, including further detailed mechanistic studies, dose response curves to determine the most effective concentration of each therapeutic agent and assessment of bio availability in children with JDM or related diseases where appropriate. Considering that up to 50% of JDM patients fail first-line therapies there remains an urgent need for new, more targeted treatments.^69–71^ Thus, the identification of a novel, ‘druggable’ target that modulates the IFN type 1 signature has broad implications for the development of new treatment strategies in JDM and potentially other IFN-driven disorders.

### Patient, public, involvement and engagement

This research has been carried out on biological samples and clinical data collected from young people and adults with and without JDM. In the early development of this body of work, it was presented and discussed at the JDM family day as part of the JDCBS. Patients, families, researchers and clinical professionals were in attendance at this meeting. Additionally, there are two young people on our advisory board at the Centre for Adolescent Rheumatology Versus Arthritis at UCL, UCLH and GOSH. They play a vital part of the Centre and attend major meetings where this project has been presented.

## Data Availability

Data are available in a public, open access repository. Data are available on reasonable request. The full RNA-seq dataset is publically avaiable on GEO with accession number: GSE221091. The RNA-seq files for sorted CD19+ B cells are also part of a previous publication with accession number: E-MTAB-5616.^73^
