# Supplementary material for: Role of CD14+ monocyte-derived oxidised mitochondrial DNA in the inflammatory interferon type 1 signature in juvenile dermatomyositis
Source: Ann Rheum Dis. 2022 Dec 23;82(5):658–69. doi: 10.1136/ard-2022-223469 (PMC10176342; doi:10.1136/ard-2022-223469)
Supplement: Supplementary data [file ard-2022-223469supp006.pdf]

Methods

Patient information

Patients who met the Bohan and Peter criteria for probable or definite JDM were included in this study, with a median age of 7 years at time of sample, recruited through the UK JDM Cohort and Biomarker Study (JDCBS) [33–35]. Inclusion in the JDCBS is offered to all patients with JDM seen in the 17 contributing centres. 99% of families consent to being in this observational study, which had 660 cases at time of this investigation; 6 have declined in >20 years. The 90 patient cases used in this study were consecutive cases available for analysis that were representative of the whole cohort (74 unique cases in functional experiments, 8 unique cases in the RNAseq experiments and 8 cases in both functional experimental and RNAseq experiments). In addition, 39 age-matched child healthy controls (controls) were recruited through the Centre for Adolescent Rheumatology. Parents and patients gave written informed consent or age-appropriate assent. The study had full ethical approval through London-Bloomsbury and North-East Yorkshire Research Ethics Committees (MREC 01/3/022). Controls samples were donated under the North Harrow ethics committee approval (REC 11/0101). All consent was obtained in accordance with the Declaration of Helsinki. Patient and control demographics are summarised in Table 1: number of participants, age at onset, diagnosis and at sample collection were collated for medications, Physicians Global Assessment (PGA; range 0–10; low scores indicate minimal disease) [36,37], Childhood Myositis Assessment Scale (CMAS; range 0–52; high scores indicate no weakness)[38], serum creatine kinase levels (U/l), medications at time of sample (oral steroids, methotrexate, ciclosporin, azathioprine, cyclophosphamide and hydroxychloroquine), myositis specific autoantibody (MSA) status, myositis associated autoantibody (MAA) status and anti-nuclear autoantibody (ANA) status (Table 1).

PBMC isolation

Peripheral blood mononuclear cells (PBMC) were isolated by density centrifugation using Lymphoprep (Stemcell technologies, Cat# 07851/07861). PBMC were stored in 90% Fetal Bovine Serum (FBS) (Gibco, cat# 10270-106) and 10% Dimethyl-sulfoxide (DMSO) (Sigma, Cat# D2650-5x10ml, CAS: 67-68-5) and cryopreserved in liquid nitrogen until use. For some assays PBMC were used fresh, as indicated.

Wilkinson MGL, *et al. Ann Rheum Dis* 2023; 82:658–669. doi: 10.1136/ard-2022-223469

Cell sorting and RNA preperation

Patient and control CD4+, CD8+, CD19+ and CD14+ cells were sorted by flow cytometry (FACS Aria III). DAPI was used to exclude dead cells. Sorted cell RNA was extracted using the PicoPure™ RNA Isolation Kit (Thermo Fisher Scientific, Cat# KIT0204).

Library preparation

Library preparation and sequencing were performed at UCL Genomics. Samples were processed using Illumina's TruSeq Stranded mRNA LT sample preparation kit (p/n RS-122-2101) according to manufacturer's instructions with minor alterations. Briefly, mRNA was isolated from 250 ng total RNA using Oligo dT beads to pull down Poly-Adenylated transcripts. The purified mRNA was fragmented using chemical fragmentation (heat and divalent metal cation) for 10 minutes and primed with random hexamers. Strand specific first strand cDNA was generated using Reverse Transcriptase and Actinomycin D. This allows for RNA dependent synthesis while preventing spurious DNA-dependent synthesis. The second cDNA strand was synthesised using dUTP in place of dTTP, to mark the second strand. The resultant cDNA is then "A-tailed" at the 3' end to prevent self-ligation and adapter dimerisation. Full length TruSeq adaptors, containing a T overhang are ligated to the A-Tailed cDNA. These adaptors contain sequences that allow the libraries to be uniquely identified by way of a 6 bp Index sequence. Successfully ligated fragments were enriched with 14 cycles of PCR. The polymerase is unable to read through uracil, so only the first strand is amplified, thus making the library strand specific.

RNA Sequencing

Libraries to be multiplexed in the same run were pooled in equimolar quantities, calculated from Qubit and Bioanalyser fragment analysis. Samples were sequenced on the NextSeq 500 instrument (Illumina, San Diego, US) using a 43 bp paired end run resulting in >15million reads per sample.

RNA Sequencing analysis

Run data were demultiplexed and converted to fastq files using Illumina's bcl2fastq

Conversion Software v2.16. Sequencing reads (in fastq format) were aligned to the GRCh38 reference sequence using TopHat v2.1.0 [39]. Alignments were processed using samtools version 1.2 and Picard tools version 1.140 (http://picard.sourceforge.net/) [40]. Aligned reads were filtered for mapq >= 4, i.e. uniquely mapping reads, and putative PCR duplicates were removed. Read summarization was performed using featureCounts [41]. Expression analysis was carried out using R version 3.2.2 [42], and differential gene expression was analysed using edgeR [43]. RNAseq data are available from ArrayExpress, accession number E-MTAB-5616.

Gene Set Enrichment Analysis

GO term and pathway enrichment analysis was carried out using 'goseq' which uses a test based on the Wallenius' noncentral hypergeometric distribution [44]. Gene set enrichment analysis was carried out with "Hallmark" gene sets from databases [45] based on calculated normalised enrichment scores (NES) and multiple adjusted p-values (q-values) were calculated. Gene scores were established for IFN type 1 (*Ly6E, MX1, USP18, RSAD2, OAS1, IFFI44L, IFI27, ISG15, IFIT1, IFI44, IFI6, SIGLEC1, IFIT3, IRF7, STAT1* [15 genes]) and mitochondrially encoded-genes (*MT-ND1, MT-ND2, MT-ND3, MT-ND4, MT-ND4L, MT-ND5, MT-ND6, MT-CO1, MT-CO2, MT-CO3, MT-CYB, MT-ATP6, MT-ATP8* [13 genes].

Flow cytometry

Frozen PBMC were used for all flow cytometry analysis. After thawing, PBMC were plated in a 96 well plate at 200,000 per well, washed with 1xPBS and incubated with Live/Dead fixable cell stains (Life Technologies) according to manufacturer's instructions. For extracellular staining, antibodies to surface proteins (see Methods table 2) were diluted in FACS buffer (500ml 1x PBS (Sigma Aldrich), 1%FBS, 0.01% sodium azide) and cells were incubated at 4°C for 20 minutes with appropriate antibody cocktails. Samples were either fixed with intracellular fixation buffer (eBioscience™ Intracellular Fixation & Permeabilization Buffer Set, Thermo Fisher Scientific, Cat# 88-8824-00) for 20 minutes at 4°C or for MitoTracker and MitoSox stains were incubated for 30 minutes at 37°C with the respective mitochondrial dyes in 1x PBS (Methods table 2) and left unfixed. For intra-cellular staining the PBMC were washed with permeabilization buffer and then incubated in the presence of

intracellular markers for 30 minutes at 4°C. The samples were washed in FACS buffer and run on BD LSR II Flow Cytometer (BD Biosciences) or on CytoFLEX Flow Cytometer (Beckman Coulter Life Sciences).

For the 2NBDG assay PBMC were stained with surface markers (as above) and then stained with the fluorescent tagged glucose tracer, 2NBDG (2-(*N*-(7-Nitrobenz-2-oxa-1,3-diazol-4-yl)Amino)-2-Deoxyglucose) according to manufacturer's instructions (2-NBDG Glucose Uptake Assay Kit, Abcam, Cat# Ab235976).

Methods table 2 – Antibodies and dyes used for flowcytometry, fluorescence microscopy and dot blot western

Antibody	Company	Catalogue and PRID
GLUT 1 monoclonal anti-human	Invitrogen	Cat#MA5-11315; PRID: AB_2809254
Goat anti-mouse IgG (H+L), AF488	Invitrogen	Cat# A11001; PRID: AB_2534069
GLUT 4 monoclonal anti-human	Invitrogen	Cat# PA5-23052; PRID: AB_11153908
Goat ant-rabbit IgG (H+L), AF555	Invitrogen	Cat# A21428; PRID: AB_2535849
CD3 anti-human monoclonal, PerCP-cy5.5	eBioscience	Cat# 45-0037-42; PRID: AB_10548513
CD4 anti-human monoclonal, APC-cy7	BioLegend	Cat# 317418; PRID: AB_571947
CD8 anti-human monoclonal, BV421	BioLegend	Cat# 301036; PRID: AB_10960142
CD19 anti-human monoclonal, Pe-cy7	BioLegend	Cat# 302216; PRID: AB_314246
CD14 anti-human monoclonal, AF700	BioLegend	Cat# 367114; PRID: AB_2566716
CD16 anti-human monoclonal, BV711	BioLegend	Cat# 367114; PRID: AB_2563802
HLA-DR anti-human monoclonal, BV510	BioLegend	Cat# 307646; PRID: AB_2561948
CD8 anti-human monoclonal, AF700	BioLegend	Cat# 344724; PRID: AB_2562790
CD3 anti-human monoclonal, BUV395	BD Bioscience	Cat# 563546; PRID: AB_2744387
CD19 anti-human monoclonal, BUV805	BD Bioscience	Cat# 749173; PRID: AB_2873553
CD4 anti-human monoclonal, Pe-cy7	BioLegend	Cat# 317414; PRID: AB_571959
CD14 anti-human monoclonal, BV421	BioLegend	Cat# 301830; PRID:AB_10959324

Wilkinson MGL, *et al. Ann Rheum Dis* 2023; 82:658–669. doi: 10.1136/ard-2022-223469

TO-PRO-3 Iodide (642/661)- 1mM solution in DMSO	ThermoFisher Scientific	Cat# T3605
LIVE/DEAD™ Fixable Blue Dead Cell Stain Kit, for UV excitation	ThermoFisher Scientific	Cat# L23105

Citrate synthase activity assay

CD14+ Monocytes were isolated from PBMC by positive selection (EasySep™ human CD14 positive selection kit II – Stemcell - 17858). The monocyte cell pellet was spun down (1000xg, 5 minutes), supernatant removed. Monocyte cell pellet was stored in -80°C freezer. All samples were thawed and re-suspended in 50μl of citrate synthase (CS) buffer (1L at pH 8.0: 12.011g Trizma base, 990ml of ddH_2O and 1ml of Triton x100). Assay carried out at 37°C in Uvikon XL spectrophotometer, pre-warmed to correct temperature. Spectrophotometer set up to measure 'autorate assay'; absorbance 412nm; one measurement per 0.5 min; set cell changer mode to 6S/6R; autozero on. Pre-warmed citrate synthase (CS) assay buffer to 37°C and keep warm throughout experiment. To each 1.5ml polystyrene cuvette for sample: 10μl 5,5'-dithio-bis-(2-nitrobenzoic acid) (DTNB; 20mM), 10μl Acetyl-CoA (10mM), 20μl sample homogenate and 950μl CS assay buffer. For reference cuvette: 10μl DTNB (20mM), 10μl Acetyl-CoA (10mM), and 960μl CS assay buffer. Cuvette inverted to mix. Assay run for 2 minutes to confirm flat baseline. 10μl oxaloacetate (20mM) added, cuvette inverted to mix and assay run for another 8 minutes. Protein content quantified against standard curve using Bradford assay (Pierce™ Coomassie (Bradford) Protein Assay Kit-23200) measured by Tecan F200 plate reader, with i-control 2.0 Tecan software, at 600nm absorbance. The calculation of CS activity is derived from the Beer-Lambert law: $A = c * \varepsilon * l$, where A is the measured absorbance, c is the sample concentration, ε is the molar extinction coefficient of 2-nitro-5-thiobenzoate (TNB (13.6×10^3 M^{-1} cm^{-1}) and l is the path length (1cm cuvettes). CS activity expressed as nmol/min/mg protein.

Imaging

Live cell imaging was performed on a Zeiss Observer 7 microscope with a 63xNA1.4 Oil objective and Hamamatsu Flash 4 camera. PBMC were stained with CD14+ (APC) in FACS buffer for 20 minutes and then with MitoTracker and MitoSox, incubated for 30 minutes at 37°C with the respective mitochondrial dyes in 1x PBS

Wilkinson MGL, *et al. Ann Rheum Dis* 2023; 82:658–669. doi: 10.1136/ard-2022-223469

(Methods table 1) and left unfixed. PBMC were suspended in FACS buffer in 96-well glass bottom imaging plates with a #1.5 coverslip base. LED illumination at 470nm, 555nm or 630nm with single band filters at (CD14 APC, MitoTracker green (excitation/emission 490⁄516nm) MitoSox (excitation/emission 510/580 nm) were used to take z-stacks at Nyquist sampling in xy & z for deconvolution with SVI Huygens software. Exposure times (20ms) and illumination intensity were kept to minimal levels to avoid phototoxicity and allow rapid imaging of each cell. Typically 500 grey levels (on a 16 bit detector) were used for each channel. Individual cells were manually cropped using Zen2.3 software, deconvolved (SVI Huygens), then processed with in house macros (GitHub) to measure within a cropped spheroid around each cell the volume & surface area of each stain (MitoTracker and MitoSox) and regions of overlap. 3D model images were created using Imaris cell imaging software (Oxford instruments).

Methods table 3 – Antibodies and dyes used for fluorescence microscopy

Antibody	Company	Catalogue and PRID
CD14 anti-human monoclonal, APC	BioLegend	Cat# 325608; RRID:AB_830681
MitoTracker green	ThermoFisher Scientific	Cat# M7514
MitoSox red	ThermoFisher Scientific	Cat# M36008

Incubation of monocytes with [U-$^{13}C_6$]glucose

CD14+ monocytes were isolated from PBMC by positive selection (EasySep™ human CD14 positive selection kit II – Stemcell - 17858). 0.5 million CD14+ monocytes were cultured in 1ml per well glucose starved media (Gibco™ DMEM, no glucose, Fisher scientific, cat# 11520416) plus 3mM glucose (D-(+)-Glucose, Sigma-Aldrich, #G8270-100G) over-night in a 24 well plate. The medium was changed to 1ml per well DMEM plus [U-$^{13}C_6$]glucose (10mM) (D-glucose (U-13C6,99%), Cambridge isotope laboratories, Inc, Cat# CLM-1396-1). 1ml mineral oil was layered on top of the cell suspension to trap gas release. Every hour for six hours and a further 24hr time point a 100μl and 10μl media samples were taken and stored before analysis for $^{13}CO_2$ and^{13}C lactate.

$^{13}CO_2$ Gas Chromatography-Isotope ratio mass spectrometry

Samples in 12ml Exetainer Tubes (Labco Ltd) were thawed at room temperature, and 100µl 1M hydrochloric acid was injected through the septum into each vial to release CO_2 from the medium. Vials were centrifuged for 30s at 500xg. Samples were then analysed on a GasBench II coupled to a Thermo Delta-XP isotope-ratio mass spectrometer (Thermo-Finnigan, Bremen, Germany). Ten repeat injections were carried out per sample, with $^{13}CO_2/^{12}CO_2$ ratios measured against Vienna Pee Dee Belemnite (VPDB) using a calibrated CO_2 reference gas. Following this, $^{13}CO_2/^{12}CO2$ ratios were then converted to mole percent excess using absolute molar ratio of ^{13}C to ^{12}C (0.0111796) in VPDB. The change in mole percent excess was then converted to pmol $^{13}CO_2$ generated using the volume of medium and concentration of bicarbonate (2.9 mM) present, which was then corrected for the number of labelled carbon atoms (6 for glucose) to obtain picomoles of substrate oxidised [46].

Lactate Gas Chromatography Mass Spectrometry

Samples were defrosted; 10-50µl sample +25µl internal standard (1mM $^{13}C_4$-beta hydroxybutyrate (BOHB)) plus 225µl of acetonitrile were mixed in an 1.5ml microcentrifuge tube. The samples were vortexed, and spun in a microcentrifuge for 5 minutes at 15,000xg. Supernatant was transferred to a GC vial and dried down under N_2 gas at room temperature. For the standards (100-1µM): 50µl standard ($^{13}C_3$-lactate) +25µl internal standard (1mM $^{13}C_4$ BOHB) were added to a GC vial. Dried down under N_2 gas at room temperature. To ensure all water was removed, 200µl of 100% ethanol was added, and dried down under N_2 gas at room temperature. To each vial, 30µl of ethyl acetate + 30µl of Bis(trimethylsilyl)trifluoroacetamide /10% trimethylsilyl chloride was added. Samples and standards were vortexed and incubated for 30 minutes at 37°C on a heat block. The samples and standards were analysed by GC/MS (Thermo DSQII trace Ultra GC) using a RXI-5Sil MS fused silica (30m x 0.25mm I.D, 0.25µm film thickness) column (Thames Restek) at a helium flow rate of 1.2ml/minute. 2µl of sample was injected into the inlet (250°C) with a split ratio of 1:8 using the following temperature programme: 60°C hold for 1 minute, 140°C increased at a rate of 10°C/minute, 240°C at a rate of 40°C/minute. Ionisation mode was positive chemical ionisation with methane at 2ml/minute. The detection mode used was Selected Ion Monitoring

Wilkinson MGL, *et al. Ann Rheum Dis* 2023; 82:658–669. doi: 10.1136/ard-2022-223469

(SIM), collecting peak-area data on the following ions: Lactate (m/z 219), $^{13}C_3$-lactate (m/z 222), and $^{13}C_4$-BOHB (internal standard) (m/z 237). The sample concentrations were calculated against a standard curve of peak area ratio 222/237. Method was modified from [47].

Mitochondria isolation and western/dot blot assay

CD14+ Monocytes were isolated from PBMC by positive selection (EasySep™ human CD14 positive selection kit II, Stemcell, cat#17858) and then mitochondria were isolated from monocytes using Mitochondria Isolation Kit for Cultured Cells (Thermo Fisher, cat#89874). Samples were prepared by diluting with 4x loading dye. PVDF membrane strips were prepared in Methanol for 1-2 minutes. Membranes were blotted and sample mix was applied in dots and left to dry for an hour. The membrane was blocked with 5% skimmed milk in PBST for an hour. The membrane was incubated with the antibody (8-OHdG – Santa Cruz – cat#sc-393871) in 5ml of 5% skimmed milk in 0.1%PBST for an hour/overnight. Membrane was washed with 5ml of 5% skimmed milk in 0.1%PBST. The membrane was incubated with the secondary antibody (rabbit anti-mouse 1:1000) in 5ml of 5% skimmed milk in 0.1% PBST for an hour. Membranes washed with 0.1%PBST. Substrate was applied to the membrane (ECL™ Prime Western Blotting System, Merck, Cat# RPN2232), image was recorded and analysed (Quantity One 1-D analysis software, Bio-rad, RRID:SCR_014280).

MT-CO3 plasmid transformation

100μl XL 10-Gold® Ultracompetent cells (Stratagene, Cat#200314) were thawed on ice and put into a new RNA/DNA free Eppendorf. 4μl of mercaptoethanol was added to the bacteria cells and incubated on ice for 10 minutes. Next, 1μl of 70ng/μl 10^{10} *Mt-CO3* plasmid was added to the sample, mixed and incubated on ice for 30 minutes. Then the sample was heat shocked at 42°C for 30 seconds and 900μl LB broth was added. The sample was then incubated for 1 hour at 37°C and shaken at 300rpm. To grow the cells, 200μl of the sample was spread over an agar plate (containing LB agar and 100mg/ml ampicillin), the plate was sealed and incubated at 37°C overnight. 3 cell populations were picked from the agar plate and added to 5ml of LB broth in a 50ml falcon which was incubated and shaken at 37°C overnight.

Mitochondrial DNA amplification by qPCR

MT-CO3 sequence was amplified by PCR insertion of DNA molecule containing MT-CO3 into plasmid vector. The plasmid vector was diluted to a known concentration of 10^{10} copy standard with the template containing 10^{10} copies per 5μl. The plasmid vector sample was serially diluted to 1:10 to make standards down to 10^{0}. Myoblast genomic DNA was used as a non-template control (NTC) and was made to a concentration of 63.4ng/μl in 200μl. From this the NTC was made up as 2x 1350μl of 0.5ng/μl (675ng in 1350μl). The positive control was diluted 1:10 and 1:100 (neat gives Ct ~ 21, 1:10 gives Ct ~ 24, 1:100 gives Ct ~ 27). DNA from the plasma samples, JDM and control, were extracted using Norgen Biotek kit Plasma/Serum RNA Purification Mini Kit (Norgen Biotek Corp, Cat#55000)– these were eluted in a minimal volume (25μl), 5μl used per reaction. 20μl of the PCR master mix was pipetted into each well; 5μl dH_2O, 1.25μl 5μM forward primer (5’-CCTTCACCATTTCCGACGGCAT), 1.25μl 5μM reverse primer (5’-ACGTGAAGTCCGTGGAAGCC), 12.5μl iQ™ SYBR® Green Supermix (BioRad, Cat# 170-8882). 5μl of NTC, standard or sample was added to each well plus 20μl master mix. Thermo cycling programme used: 95°C for 5 minutes, 44 cycles of: 95°C for 15 seconds, 63°C for 60 seconds, hold 63 degrees for 3 minutes. Melt curve; start at 68°C, increase by increments of 0.5°C for 30 seconds to 95°C. CFX Maestro software (RRID:SCR_018057) was used to analyse qPCR data against the standard curve to quantify MT-CO3 copy number per volume of plasma sample.

Oxidised mitochondrial DNA reagent generation

Whole blood was collected into EDTA, lysed, and DNA was purified using UltraPure™ Phenol:Chloroform:Isoamyl Alcohol (25:24:1, v/v) (ThermoFisher Scientific, cat#15593031) and precipitated using ethanol (method previously described) [48]. Amplification of the DNA sample generated two long PCR amplicons (9.1kb and 11.2kb) spanning the entire mtDNA genome using PCR primers MTL-1/2 (*MTL-F1* 5’-AAAGCACATACCAAGGCCAC, *MTL-R1* 5’-TTGGCTCTCCTTGCAAAGTT, *MTL-F2* 5’-TATCCGCCATCCCATACATT, *MTL-R2* 5’-AATGTTGAGCCGTAGATGC (Merck)). 10μl of human genomic DNA per 200μl PCR tube, 40μl PCR master mix per 200μl PCR tube (5μl 10X LA PCR Buffer II

(25mM MgC_2), 2µl 10µM forward primer (MTL-F1, MRL-F2), 2µl 10µM reverse primer (MTL-R1, MRL-R2), 8µl 2.5mM dNTP mix, 22.5µl nuclease free water, 0.5µl TaKaRa LA Taq (5U/µl) (TaKaRa LA Taq® DNA Polymerase (Mg2+ free buffer), TaKaRa, Cat# RR002A). Sample ran on pre-programmed thermal cycler. Programme: pre-heat lid set to 100°C. 94°C for 5 minutes. 30 cycles of; 98°C for 15s, 68°C for 10s 9slow ramp from 68°C to 60°C at 0.2°C per second), 60°C for 15s, 68°C for 11 minutes. 72°C for 10 minutes. Hold at 10°C. Amplicons of mtDNA were purified from residual primers and dNTPs by MSB Spin PCRapace (B-Bridge International). The DNA samples were quantified using a nanodrop, and agarose gel run with 1kb plus DNA ladder (BioLabs inc.). The mtDNA samples were oxidised using the oxidation method: 3µl of H_2O, 50µl mtDNA (500µg) in phosphate buffer (100mM 1M K_2HPO_4 and 1M KH_2PO_4 pH 7.4), 42µl 2.4mM $CrCl_3$, 6µl H_2O_2 (1:1000) (Merck, Cat# 230723-100G). The mix was incubated at 37°C for 2hr in water bath. To precipitate the mtDNA, 10µl sodium acetate (3M, pH5) and 200µl of -20°C 100% ethanol (ETOH) was added to the mtDNA sample and incubated at -20°C for 10 minutes. The sample was spun at 13,000xg at 4°C for 10 minutes, then the supernatant was discarded. 500µl of 70% ETOH was added to the remaining mtDNA pellet. The sample was spun again at 13,000xg at 4°C for 10 minutes, then the ETOH was removed. The sample pellet was spun again at 13,000xg at 4°C for 10 minutes to dry. The mtDNA sample was re-suspended in DNA/RNA free ddH_2O at the required concentration. To confirm the mtDNA was oxidised, the oxmtDNA samples were quantified for oxidation using HT 8-oxo-dG ELISA kit II (Bio-techne R&D systems, Cat# 4380-096-K) according to manufacturer’s instructions.

Oxidised mitochondrial DNA culture

PBMC were thawed and re-suspended in RPMI medium 1640 (Gibco, cat# 21875-034), supplemented with 10% FBS and 1% penicillin streptomycin (Pen Strep, Gibco, Cat# 15140-122). 0.2M thawed PBMC were cultured with either; RPMI medium (unstimulated or untreated control), IFN-α (1000U/ml – R&D – Cat#11100-1-5UM), oxidised/non-oxidised mtDNA (4µg/ml) with LL37 (InvivoGen, Cat#tlrl-l37), with or without cGAS inhibitor (G140) (1mM) (InvivoGen, Cat# inh-g140), TLR-9 antagonist (ODN TTAGGG (A151)) (5mg/ml - 1:500 dilution) (InvivoGen, Cat#tlrl-ttag151) or N-acetyl-L-cysteine (7.5mM) (Sigma Aldrich, Cat#A7250-5G) added 24hr into the

culture with oxidised mtDNA for a further 24hr. PBMC prepared for RNA extraction and isolation using the PicoPure™ RNA Isolation Kit (Thermo Fisher Scientific, Cat# KIT0204). Nanodrop quantification; all samples diluted to the same concentration with ddH_2O (RNA/DNA free) to make 10μl of RNA. cDNA generated using iScript™ cDNA synthesis kit (Bio-Rad, Cat#1708891) according to manufacturer's instructions.

Gene expression analysis by qPCR

qPCR was run for 3 genes; *PPIA* (House keeper), *MX1* and *RSAD2* (Hs99999904_m1 *PPIA*, Hs00895608_m1 *MX1*, Hs00369813_m1 *RSAD2*, TaqMan gene Expression Assay (FAM), Applied Biosystems™, Cat#4331182). Master mix per well (16μl): 1μl primer, 5μl dH_2O, 10μl master mix (TaqMan™ fast advanced master mix, Applied Biosystems™, Cat#4444557). 16μl of the master mix was added to 4μl cDNA sample. Thermo cycler programme: 50°C for 2 minutes, 95°C for 10 minutes 39 cycles of: 95°C for 15 seconds, 60°C for 60 seconds, plate read. Analysis by Bio-Rad CFX Maestro software. Gene expression normalised to house keeper (PPIA) by dCT, normalised to experimental control by ddCT and expressed as power of control.

Statistics

RNA-seq expression analysis was carried out using R version 3.2.2 [42], and differential gene expression was analysed using edgeR [43]. GO term and pathway enrichment analysis was carried out using 'goseq' which uses a test based on the Wallenius' noncentral hypergeometric distribution [44]. Gene set enrichment analysis (GSEA) was carried out with "Hallmark" gene sets from databases [45] based on calculated normalised enrichment scores (NES) and multiple adjusted p-values (q-values) were calculated. For the imaging data analysis of the effect of JDM versus health control as a fixed effect in a linear mixed effects model, with a random effect included to account for individual-specific effects. For each outcome, models were built sequentially starting with an intercept-only model with a random effect for individual, followed by an otherwise identical model with the outcome log- or square root-transformed as appropriate, followed by the addition of disease status as a fixed effect. P-values were generated using nested ANOVA. All other statistics were performed using Prism 9. Bar charts shown with median values and whole range.

Wilkinson MGL, *et al. Ann Rheum Dis* 2023; 82:658–669. doi: 10.1136/ard-2022-223469

For parametric data, t-test or one-way ANOVA with Tukey's multiple comparison were used to calculate significance. For non-parametric data Mann-Whitney test or Kruskal-Wallis test with Dunn's multiple comparisons were used to calculate significance. Pearson's correlation test was used to calculate correlation among variables of interest, significance and r-value.

Wilkinson MGL, *et al. Ann Rheum Dis* 2023; 82:658–669. doi: 10.1136/ard-2022-223469
