# Supplementary material for: Role of CD14+ monocyte-derived oxidised mitochondrial DNA in the inflammatory interferon type 1 signature in juvenile dermatomyositis
Source: Ann Rheum Dis. 2022 Dec 23;82(5):658–69. doi: 10.1136/ard-2022-223469 (PMC10176342; doi:10.1136/ard-2022-223469)
Supplement: Supplementary data [file ard-2022-223469supp005.pdf]

Table 1 - Patient and control demographics and clinical characteristics

Patient characteristics	RNA-seq #			Functional experiments	
	JDM pre-treatment	JDM on-treatment	Controls	JDM	Controls
**Number of participants**	**10**	**11**	**4**	**82**	**35**
Age at onset (years), median [range]	9.14 [3.71-15.19]	8.03 [2.56-15.19]	N/A	5.81 [0.79-15.37]	N/A
Age at diagnosis (years), median [range]	9.35 [3.96-15.36]	8.45 [2.73-15.36]	N/A	6.11 [1.28-16.20]	N/A
Age at sample (years), median [range]	9.78 [4.00-15.39]	9.56 [3.79-16.13]	9.34 [7.15-11.12]	7.50 [1.28-25.74]	11.45 [6.33-17.98]
Time since diagnosis (years), median [range]	0.12 [0.00--0.82]	0.97 [0.77-1.12]	N/A	0.07 [0.00-14.69]	N/A
Sex Female/Male (%F)	8/2 [80.00]	7/4 [63.64]	2/2	56/26 [68.29]	23/12 [65.71]
**Clinical information at time of sample:**					
PGA*(median [range])	7.1 [3.0-9.6]	1.5 [0.0-6.7]	N/A	3.5 [0.0-9.0]	N/A
CMAS* (median [range])	16 [0-31]	46 [23-53]	N/A	41 [0-53]	N/A
CK* value (median [range])	7282 [68-25937]	1355 [66-12689]	N/A	118 [17-25937]	N/A
**Medication at time of sample** [n=yes(%)]:					
Oral steroids	1 (10.00)	9 (81.82)	N/A	36 (34.28)	N/A
Methotrexate	0 (0.00)	11 (100.0)	N/A	40 (38.09)	N/A
Ciclosporin	0 (0.00)	0 (0.00)	N/A	1 (0.95)	N/A
Azathioprine	0 (0.00)	1 (9.09)	N/A	3 (2.86)	N/A
Cyclophosphamide	0 (0.00)	0 (0.00)	N/A	4 (3.81)	N/A
Hydroxychloroquine	0 (0.00)	1 (9.09)	N/A	9 (8.57)	N/A
**Myositis Specific Autoantibody (MSA)~** [n=positive(%)]:					
anti-Mi2	2 (20.00)	3 (27.27)	N/A	5 (5.75)	N/A
anti-TIF1g	1 (10.00)	2 (18.18)	N/A	11 (12.64)	N/A
anti-NXP2	3 (30.00)	1 (9.09)	N/A	15 (17.24)	N/A
anti-MDA5^	0 (0.00)	0 (0.00)	N/A	9 (10.34)	N/A
anti-SRP	1 (10.00)	1 (9.09)	N/A	4 (4.60)	N/A
anti-OJ	0 (0.00)	0 (0.00)	N/A	1 (1.15)	N/A
anti-HMGCR	0 (0.00)	0 (0.00)	N/A	1 (1.15)	N/A
**Myositis Associated Autoantibody (MAA)** [n=positive(%)]:					
anti-PmScl 75	0 (0.00)	1 (9.09)	N/A	5 (5.75)	N/A
anti-Ro52^	0 (0.00)	0 (0.00)	N/A	2 (2.30)	N/A
anti-U1RNP	0 (0.00)	0 (0.00)	N/A	3 (3.45)	N/A
Unknown bands	2 (20.00)	1 (9.09)	N/A	8 (9.20)	N/A
Negative	1 (10.00)	2 (18.18)	N/A	23 (26.44)	N/A
Not Done	0 (0.00)	0 (0.00)	N/A	2 (2.30)	N/A
**ANA"** [n=positive(%)]:	4 (40.00)	6 (54.55)	N/A	43 (55.84)"	N/A

6 RNA-seq samples were paired
*Physical Global Assessment (PGA) [0-10] (2.65% of samples mssing data), Childhood Myositis Assessment Scale (CMAS) [0-53] (19.47% of samples mssing data), Creatine Kinase (CK) [U/L - >0 measured in serum] (15.92% of samples mssing data)
^2 patients were positive for both anti-MDA5 and anti-Ro52
~MSA/MAA method - Immunoprecipitation (70.11%), Extended myositis Immunoblot, RUH, Bath (26.44%), Extended Myositis Immunoblot, RMCH, Manchester (1.15%), Not done (2.30%)
ANA" - data for ANA result missing in n=5 patients
