# Supplementary material for: Role of CD14+ monocyte-derived oxidised mitochondrial DNA in the inflammatory interferon type 1 signature in juvenile dermatomyositis
Source: Ann Rheum Dis. 2022 Dec 23;82(5):658–69. doi: 10.1136/ard-2022-223469 (PMC10176342; doi:10.1136/ard-2022-223469)
Supplement: Supplementary data [file ard-2022-223469supp001.pdf]

Supplementary Figure 1

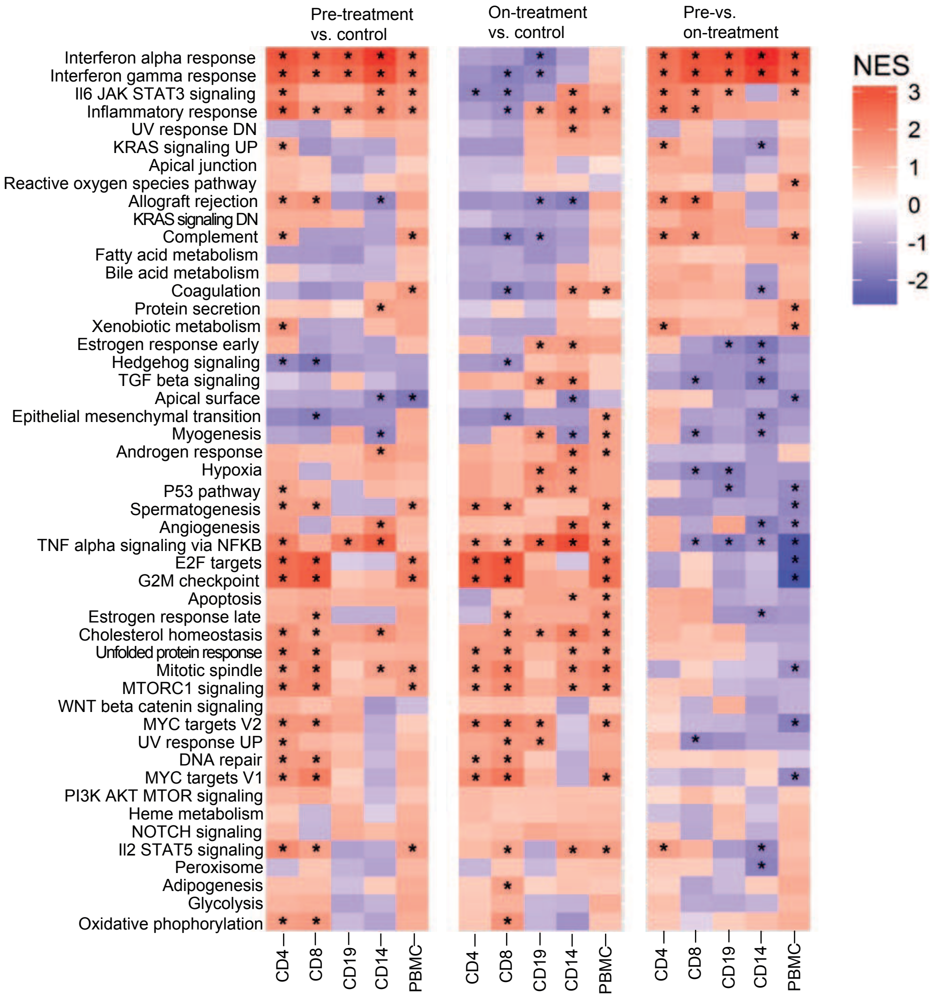

Supplementary Figure 1. Transcriptional analysis validates strong IFN type 1 signaturein in all cell types in JDM pre-treatment. Transcriptomic analysis of flow-sorted CD4+, CD8+, CD19+ and CD14+ cells from JDM patients taken pre-treatment [n=10] and 12 months on-treatment [n=11], and age-matched healthy controls [n=4]. Heatmap of Gene-Set Enrichment Analysis (GSEA) for enriched Hallmark gene-sets. For each cell type every gene was ranked comparing pre-treatment vs. control, on-treatment vs. control and pre- vs. on-treatment. Normalised Enrichment Score (NES): red represents an up-regulated pathway and blue a down-regulated pathway, stars represent adjusted p-values (* p<0.05).

Supplementary Figure 2

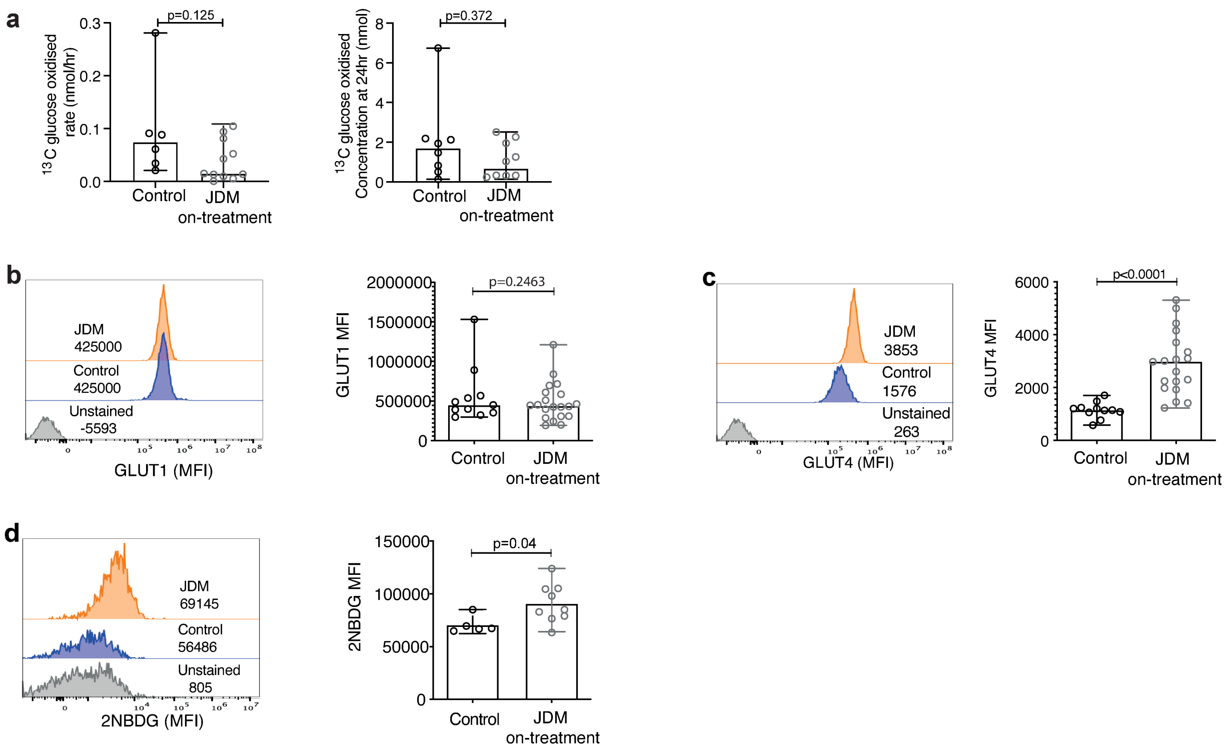

Supplementary Figure 2. There is abnormal cellular metabolism in JDM CD14+ monocytes.
(**a**) Bar graphs show 13C labelled glucose metabolism rate (left) and final concentration into oxidised glucose in CD14+ monocytes from JDM [n=6] and controls [n=6]. Representative histogram (left) and bar graphs (right) show (**b**) GLUT1 and (**c**) GLUT4 MFI in CD14+ monocytes from JDM [n=20] vs. control [n=11]. (**d**) Representative histogram (left) and bar graphs (right) show 2NBDG MFI in JDM CD14+monocytes [n=9] compared to controls [n=5]. All bar graphs: median with range shown. Statistical analysis: non-parametric Mann-Whitney test, p-value.

Supplementary Figure 3

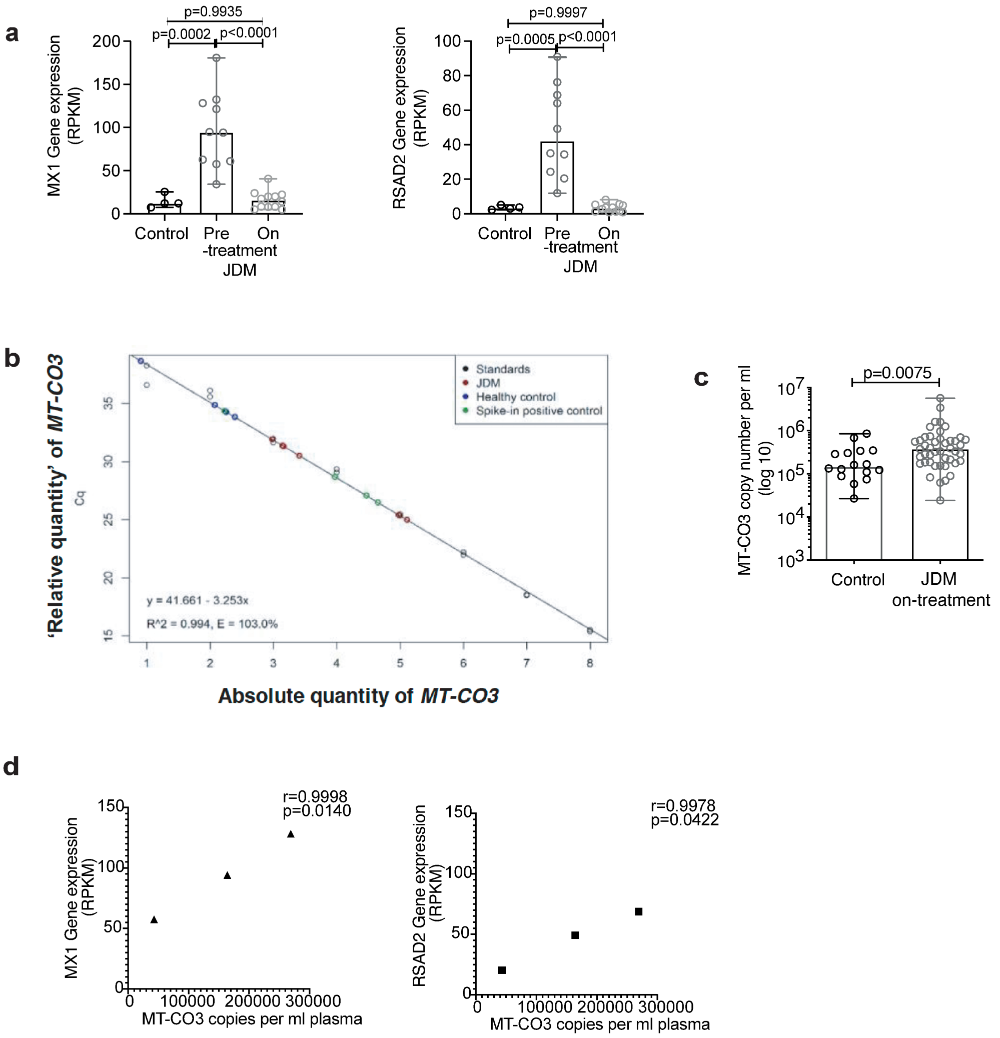

Supplementary Figure 3. Non-oxidised mitochondrial DNA correlated with increased ISG in pre-treatment JDM
(**a**) Gene expression analysis of CD14+ monocytes from JDM patients taken pre-treatment [n=10] and 12 months on-treatment [n=12], and controls [n=3]. Reads per kilobase pair of transcript (RPKM) gene expression of *MX1* and *RSAD2* (left to right). (**b**) Standard curve of MT-CO3 for standards, spike-in positive controls, JDM and age-matched healthy control samples. (**c**) MT-CO3 copy number per ml of plasma (mtDNA) for JDM pre-treatment [n=45] and controls [n=16]. (**d**) MT-CO3 copy number per ml of blood for JDM pre-treatment [n=3] correlated to matched sample RPKM gene expression values for MX1 and RSAD2 (left to right).Bar graph: median with range shown.Statistical analysis: (**a**) Non-parametric Kruskal-Wallis test with Dunn's multiple comparisons, adjusted p-values. (**c**) Non-parametric Mann-Whitney tests, p-values. (**d**) r and p-values calculated by Pearson correlation.

Supplementary Figure 4

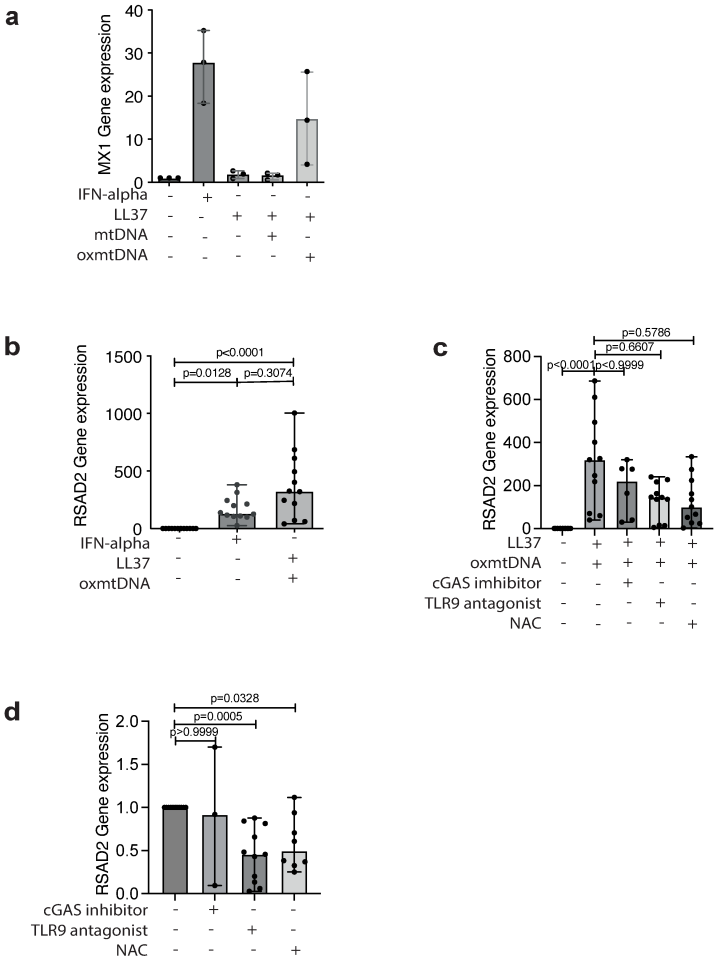

Supplementary Figure 3. Non-oxidised mitochondrial does not induce ISG, but oxidised does and can be blocked by TLR9 antagonist and NAC

(**a**) Bar graph shpws relative expression of IFN stimulated gene (ISG) *MX1* in control PBMC [n=3] incubated with IFN-alpha, LL37, mtDNA (+LL37), oxidised mtDNA (with LL37) or no stimuli. (b) Bar graphs show relative expression of ISG *RSAD2* in control PBMC [n=12] incubated with IFN-alpha, oxmtDNA (with LL-37) or no stimuli (unstim). (c) Bar graphs show relative expression of ISG *RSAD2* in control PBMC incubated with no stimuli, oxmtDNA (+LL-37) [n=12] with or without cGAS inhibitor [n=6], TLR-9 antagonist [ODN TTAGGG(A151)] [n=11] or N-acetylcysteine (NAC) [n=11]. (d) Bar graphs show gene expression of ISG *RSAD2* in PBMC from JDM patients pre-treatment cultured with or without cGAS inhibitor [n=3], TLR-9 antagonist [n=11], NAC [n=8] or in medium alone (untreated) [n=6]. Bar graph: median with range shown.Statistical analysis: (**a-d**) Non-parametric Kruskal-Wallis test with Dunn's multiple comparisons, adjusted p-values.
