# Supplementary material for: Role of CD14+ monocyte-derived oxidised mitochondrial DNA in the inflammatory interferon type 1 signature in juvenile dermatomyositis
Source: Ann Rheum Dis. 2022 Dec 23;82(5):658–69. doi: 10.1136/ard-2022-223469 (PMC10176342; doi:10.1136/ard-2022-223469)
Supplement: Supplementary data [file ard-2022-223469supp002.pdf]

Supplementary Table 1 - JDM pre-treatment vs age-matched healthy control significantly DEG (p≤0.05)

Ensemble ID	hgnc_symbol	log fold change	Adjusted p value
ENSG00000172575	RASGRP1	-4.37211198	2.24E-24
ENSG00000167286	CD3D	-4.666564649	1.72E-22
ENSG00000107742	SPOCK2	-4.360415147	1.72E-22
ENSG00000180096		-2.213826247	4.96E-22
ENSG00000198821	CD247	-4.212664852	6.77E-22
ENSG00000198286	CARD11	-3.980638073	9.31E-21
ENSG00000211751	TRBC1	-4.429073007	2.03E-18
ENSG00000111371	SLC38A1	-2.385863349	2.03E-18
ENSG00000167984	NLRC3	-2.851013671	2.03E-18
ENSG00000211772	TRBC2	-3.848305286	4.30E-17
ENSG00000185101	ANO9	-4.119222231	4.76E-17
ENSG00000182866	LCK	-4.062812135	5.72E-17
ENSG00000115085	ZAP70	-4.147512211	9.18E-17
ENSG00000069667	RORA	-4.869341336	1.04E-16
ENSG00000101096	NFATC2	-2.831487036	1.35E-16
ENSG00000135127	BICDL1	-3.576759892	2.62E-16
ENSG00000160185	UBASH3A	-4.657218676	4.20E-15
ENSG00000172005	MAL	-5.377418997	6.46E-15
ENSG00000142173	COL6A2	-5.335273567	9.75E-15
ENSG00000088387	DOCK9	-3.222329725	1.77E-14
ENSG00000138795	LEF1	-4.449435465	2.96E-14
ENSG00000099204	ABLIM1	-3.584001413	5.79E-14
ENSG00000165929	TC2N	-4.804614256	1.08E-13
ENSG00000113263	ITK	-3.192369031	1.22E-13
ENSG00000197540	GZMM	-3.579783868	1.40E-13
ENSG00000198851	CD3E	-4.400703314	1.89E-13
ENSG00000127152	BCL11B	-4.806303961	3.23E-13
ENSG00000100385	IL2RB	-4.83925198	4.29E-13
ENSG00000142303	ADAMTS10	-2.457574491	4.40E-13
ENSG00000280135		-4.826963634	4.98E-13
ENSG00000152495	CAMK4	-3.355410449	6.37E-13
ENSG00000110448	CD5	-3.765399452	7.45E-13
ENSG00000111796	KLRB1	-5.177955918	1.17E-12
ENSG00000139193	CD27	-4.160431804	1.43E-12
ENSG00000215788	TNFRSF25	-2.605278827	1.55E-12
ENSG00000141293	SKAP1	-3.675465711	2.39E-12
ENSG00000277734	TRAC	-3.934848077	2.57E-12
ENSG00000013725	CD6	-3.720643916	4.88E-12
ENSG00000081059	TCF7	-3.983272099	6.34E-12
ENSG00000245164	LINC00861	-4.331322342	1.19E-11
ENSG00000163519	TRAT1	-4.069345096	2.49E-11
ENSG00000159753	CARMIL2	-2.23931152	3.71E-11
ENSG00000067840	PDZD4	-4.607733843	4.43E-11
ENSG00000162894	FCMR	-3.132791023	4.83E-11

ENSG00000168685	IL7R	-4.307669584	5.03E-11
ENSG00000146674	IGFBP3	-7.787654438	5.50E-11
ENSG00000174080	CTSF	-2.407169086	5.50E-11
ENSG00000175265	GOLGA8A	-2.453462265	5.50E-11
ENSG00000124181	PLCG1	-2.348427418	5.64E-11
ENSG00000134954	ETS1	-2.08406885	5.94E-11
ENSG00000065675	PRKCQ	-3.00987945	6.44E-11
ENSG00000211829	TRDC	-4.777951319	4.53E-10
ENSG00000130787	HIP1R	-2.935653694	5.00E-10
ENSG00000089012	SIRPG	-4.022974816	6.87E-10
ENSG00000165185	KIAA1958	1.963697048	7.68E-10
ENSG00000179639	FCER1A	-5.514315116	8.07E-10
ENSG00000112182	BACH2	-3.006094519	9.97E-10
ENSG00000008517	IL32	-3.607461402	1.04E-09
ENSG00000226950	DANCR	-1.844394618	1.14E-09
ENSG00000255198	SNHG9	-1.584034177	1.45E-09
ENSG00000187109	NAP1L1	-1.053601182	1.46E-09
ENSG00000100346	CACNA1I	-4.863194194	1.68E-09
ENSG00000079616	KIF22	-1.151215826	3.09E-09
ENSG00000116106	EPHA4	-5.567887063	3.15E-09
ENSG00000124203	ZNF831	-3.510236283	4.43E-09
ENSG00000173208	ABCD2	-4.72311326	4.46E-09
ENSG00000111674	ENO2	-3.196163577	6.30E-09
ENSG00000101224	CDC25B	-1.129481246	7.84E-09
ENSG00000107485	GATA3	-3.353634116	7.95E-09
ENSG00000055130	CUL1	1.136163256	8.91E-09
ENSG00000228903	RASA4CP	-1.536207884	9.19E-09
ENSG00000116251	RPL22	-0.877542988	9.87E-09
ENSG00000163359	COL6A3	-3.888005371	9.96E-09
ENSG00000114942	EEF1B2	-0.939459883	1.74E-08
ENSG00000027869	SH2D2A	-3.852281814	1.84E-08
ENSG00000101082	SLA2	-2.520074843	1.97E-08
ENSG00000184613	NELL2	-3.946342491	1.97E-08
ENSG00000181847	TIGIT	-5.155733666	3.11E-08
ENSG00000233355	CHRM3-AS2	-3.400645916	3.87E-08
ENSG00000107833	NPM3	-1.578034237	4.14E-08
ENSG00000197635	DPP4	-3.733699696	4.70E-08
ENSG00000130589	HELZ2	2.2513553	5.58E-08
ENSG00000178562	CD28	-3.098517385	6.00E-08
ENSG00000160710	ADAR	1.215480815	6.50E-08
ENSG00000227507	LTB	-1.862720539	6.84E-08
ENSG00000089009	RPL6	-0.748397376	9.87E-08
ENSG00000085662	AKR1B1	-1.089965676	1.10E-07
ENSG00000128739	SNRPN	-1.290619576	1.25E-07
ENSG00000112514	CUTA	-0.756058338	1.55E-07
ENSG00000213626	LBH	-2.388225632	1.67E-07
ENSG00000127586	CHTF18	-1.221094486	1.72E-07
ENSG00000196705	ZNF431	-1.021681712	1.84E-07

ENSG00000149527	PLCH2	-3.276347883	2.03E-07
ENSG00000115607	IL18RAP	-4.42371749	2.12E-07
ENSG00000172428	COPS9	-0.808347429	2.68E-07
ENSG00000160654	CD3G	-2.123428048	2.92E-07
ENSG00000118922	KLF12	-1.513965177	2.94E-07
ENSG00000178922	HYI	-2.027160481	3.36E-07
ENSG00000135960	EDAR	-5.309092358	3.67E-07
ENSG00000063180	CA11	-1.687963992	3.87E-07
ENSG00000204475	NCR3	-4.61496023	4.12E-07
ENSG00000183486	MX2	2.118433822	4.12E-07
ENSG00000137970	RPL7P9	-0.916475831	4.21E-07
ENSG00000224877	NDUFAF8	-1.514406584	4.21E-07
ENSG00000227008		-1.349140593	4.41E-07
ENSG00000198034	RPS4X	-0.787257334	4.53E-07
ENSG00000220205	VAMP2	-0.865276062	4.53E-07
ENSG00000162910	MRPL55	-0.98446521	4.65E-07
ENSG00000172673	THEMIS	-2.612768154	5.50E-07
ENSG00000144741	SLC25A26	-0.937348354	5.73E-07
ENSG00000105369	CD79A	-2.533950726	5.73E-07
ENSG00000211747	TRBV20-1	-5.001990395	6.45E-07
ENSG00000178429	RPS3AP5	-1.119313459	6.65E-07
ENSG00000153283	CD96	-3.718072664	6.94E-07
ENSG00000169116	PARM1	-3.833469332	6.99E-07
ENSG00000141542	RAB40B	-1.584822227	7.12E-07
ENSG00000136816	TOR1B	1.661466843	7.14E-07
ENSG00000167792	NDUFV1	-0.748355878	7.57E-07
ENSG00000105808	RASA4	-1.51149014	7.68E-07
ENSG00000255026		-3.923439409	8.16E-07
ENSG00000204252	HLA-DOA	-2.090582557	8.20E-07
ENSG00000157778	PSMG3	-0.957103712	9.02E-07
ENSG00000178982	EIF3K	-0.707875815	9.42E-07
ENSG00000113319	RASGRF2	-3.131596639	9.46E-07
ENSG00000109452	INPP4B	-2.808950053	9.80E-07
ENSG00000186468	RPS23	-0.801626846	1.06E-06
ENSG00000114023	FAM162A	-1.070150643	1.12E-06
ENSG00000170486	KRT72	-4.804621154	1.15E-06
ENSG00000249115	HAUS5	-0.900095482	1.24E-06
ENSG00000146285	SCML4	-2.5735471	1.29E-06
ENSG00000156110	ADK	-1.426476686	1.36E-06
ENSG00000055332	EIF2AK2	1.922776604	1.37E-06
ENSG00000151612	ZNF827	-3.605420689	1.44E-06
ENSG00000224078	SNHG14	-1.307941758	1.48E-06
ENSG00000161405	IKZF3	-1.575632044	1.58E-06
ENSG00000143947	RPS27A	-0.835493668	1.71E-06
ENSG00000138035	PNPT1	1.755181454	1.78E-06
ENSG00000169583	CLIC3	-5.395727002	1.93E-06
ENSG00000172366	MCRIP2	-1.306734612	2.31E-06
ENSG00000260314	MRC1	-2.728732538	2.38E-06

ENSG00000122406	RPL5	-0.740590485	2.44E-06
ENSG00000165272	AQP3	-3.260598656	2.49E-06
ENSG00000116824	CD2	-1.501793069	2.74E-06
ENSG00000168913	ENHO	-2.311462504	2.76E-06
ENSG00000262049		-1.147766491	2.76E-06
ENSG00000188157	AGRN	2.326251896	3.05E-06
ENSG00000142168	SOD1	-0.685890108	3.05E-06
ENSG00000135736	CCDC102A	-4.883665353	3.28E-06
ENSG00000132274	TRIM22	1.370483686	3.34E-06
ENSG00000198780	FAM169A	-3.813724924	3.46E-06
ENSG00000172508	CARNS1	-3.055639748	3.54E-06
ENSG00000076685	NT5C2	0.770884796	3.55E-06
ENSG00000126756	UXT	-0.744742703	3.63E-06
ENSG00000166889	PATL1	1.091549368	3.66E-06
ENSG00000106628	POLD2	-1.06206495	3.72E-06
ENSG00000188186	LAMTOR4	-0.916393548	3.72E-06
ENSG00000135919	SERPINE2	-3.558353077	3.76E-06
ENSG00000278133		-0.932403977	3.94E-06
ENSG00000117419	ERI3	-0.721420156	4.50E-06
ENSG00000124201	ZNFX1	1.267927481	4.50E-06
ENSG00000078596	ITM2A	-2.477412718	4.59E-06
ENSG00000132704	FCRL2	-2.799716883	4.89E-06
ENSG00000188322	SBK1	-2.399130703	4.89E-06
ENSG00000105373	GLTSCR2	-0.898508387	4.99E-06
ENSG00000126353	CCR7	-2.679354515	5.00E-06
ENSG00000174917	C19orf70	-0.871664951	5.25E-06
ENSG00000034053	APBA2	-2.53295175	5.27E-06
ENSG00000235162	C12orf75	-2.973063118	5.51E-06
ENSG00000142937	RPS8	-0.817559569	5.53E-06
ENSG00000153107	ANAPC1	-0.64329563	5.83E-06
ENSG00000213609	RPL7AP50	-1.845605555	6.32E-06
ENSG00000136573	BLK	-3.314176969	6.53E-06
ENSG00000185885	IFITM1	2.444848625	6.71E-06
ENSG00000115604	IL18R1	-3.674621514	6.90E-06
ENSG00000232869	TRBV29-1	-4.319513882	7.00E-06
ENSG00000147604	RPL7	-0.762630505	7.98E-06
ENSG00000137200	CMTR1	1.045635766	8.03E-06
ENSG00000173193	PARP14	1.73485482	8.53E-06
ENSG00000188785	ZNF548	-1.066082348	8.79E-06
ENSG00000106624	AEBP1	-2.950264749	9.39E-06
ENSG00000102245	CD40LG	-4.01657469	9.42E-06
ENSG00000271109		-3.797923107	9.42E-06
ENSG00000197180		-1.555610547	1.01E-05
ENSG00000007255	TRAPPC6A	-1.159126999	1.02E-05
ENSG00000117298	ECE1	1.020685313	1.07E-05
ENSG00000073861	TBX21	-3.363953663	1.08E-05
ENSG00000182774	RPS17	-0.762953277	1.09E-05
ENSG00000100836	PABPN1	-0.637710325	1.16E-05

ENSG00000186395	KRT10	-0.970691422	1.17E-05
ENSG00000232442		-1.609582123	1.18E-05
ENSG00000137154	RPS6	-0.712467915	1.18E-05
ENSG00000167106	FAM102A	-1.30879207	1.18E-05
ENSG00000119922	IFIT2	3.752091831	1.18E-05
ENSG00000160856	FCRL3	-2.978467614	1.24E-05
ENSG00000099622	CIRBP	-0.657377765	1.24E-05
ENSG00000188243	COMMD6	-0.655725038	1.24E-05
ENSG00000237702	TRBV3-1	-4.213118599	1.28E-05
ENSG00000186567	CEACAM19	-1.770816673	1.28E-05
ENSG00000185697	MYBL1	-2.32613539	1.30E-05
ENSG00000100316	RPL3	-0.705990222	1.34E-05
ENSG00000279865		-1.312120358	1.41E-05
ENSG00000174444	RPL4	-0.662627634	1.41E-05
ENSG00000100814	CCNB1IP1	-0.811013886	1.43E-05
ENSG00000180644	PRF1	-3.423921806	1.47E-05
ENSG00000089157	RPLP0	-0.733627731	1.47E-05
ENSG00000163600	ICOS	-2.73399507	1.48E-05
ENSG00000204580	DDR1	-1.78061951	1.61E-05
ENSG00000178035	IMPDH2	-1.092394288	1.62E-05
ENSG00000090266	NDUFB2	-0.839182412	1.62E-05
ENSG00000021300	PLEKHB1	-2.525709748	1.63E-05
ENSG00000105202	FBL	-0.625597097	1.63E-05
ENSG00000211896	IGHG1	-4.268185717	1.66E-05
ENSG00000072858	SIDT1	-1.381490694	1.73E-05
ENSG00000145649	GZMA	-3.776231117	1.73E-05
ENSG00000100351	GRAP2	-1.973045386	1.94E-05
ENSG00000263731		-1.264369828	1.95E-05
ENSG00000114127	XRN1	0.867428862	1.99E-05
ENSG00000205413	SAMD9	2.377337732	1.99E-05
ENSG00000198467	TPM2	-1.528219096	2.01E-05
ENSG00000196531	NACA	-0.570138659	2.01E-05
ENSG00000168028	RPSA	-0.753447691	2.06E-05
ENSG00000134321	RSAD2	3.564939193	2.21E-05
ENSG00000244968	LIFR-AS1	3.624087319	2.31E-05
ENSG00000145425	RPS3A	-0.801073471	2.50E-05
ENSG00000102921	N4BP1	1.093827658	2.50E-05
ENSG00000083845	RPS5	-0.834016354	2.74E-05
ENSG00000232573	RPL3P4	-0.932267398	2.82E-05
ENSG00000173114	LRRN3	-3.644425747	2.84E-05
ENSG00000225200		-1.295341307	2.88E-05
ENSG00000211897	IGHG3	-5.163121427	3.05E-05
ENSG00000166016	ABTB2	3.448181273	3.05E-05
ENSG00000168813	ZNF507	-1.327248828	3.05E-05
ENSG00000110717	NDUFS8	-0.717896287	3.29E-05
ENSG00000269892		-1.847138793	3.29E-05
ENSG00000214485	RPL7P1	-1.260392854	3.40E-05
ENSG00000162585	FAAP20	-0.74898593	3.45E-05

ENSG00000111716	LDHB	-0.844356557	3.50E-05
ENSG00000173436	MINOS1	-0.771644993	3.57E-05
ENSG00000174748	RPL15	-0.556475452	3.57E-05
ENSG00000148303	RPL7A	-0.661606989	3.57E-05
ENSG00000181754	AMIGO1	-2.495577933	3.63E-05
ENSG00000198755	RPL10A	-0.692288949	3.75E-05
ENSG00000089220	PEBP1	-0.806614569	3.90E-05
ENSG00000140464	PML	1.133861417	3.90E-05
ENSG00000198932	GPRASP1	-1.831353092	4.18E-05
ENSG00000169567	HINT1	-0.574863325	4.34E-05
ENSG00000108821	COL1A1	-5.660872721	4.35E-05
ENSG00000153933	DGKE	-1.000084265	4.36E-05
ENSG00000269911		-4.60673373	4.39E-05
ENSG00000173821	RNF213	1.400489637	4.54E-05
ENSG00000141664	ZCCHC2	1.437673678	4.63E-05
ENSG00000170989	S1PR1	-2.303846168	4.73E-05
ENSG00000228956	SATB1-AS1	-2.179636384	4.74E-05
ENSG00000005448	WDR54	-1.225430467	4.75E-05
ENSG00000114391	RPL24	-0.645439042	4.89E-05
ENSG00000137628	DDX60	2.140555487	4.99E-05
ENSG00000104490	NCALD	-2.941458087	4.99E-05
ENSG00000078589	P2RY10	-2.578006789	5.12E-05
ENSG00000124256	ZBP1	2.313964477	5.12E-05
ENSG00000160075	SSU72	-0.490398411	5.33E-05
ENSG00000127184	COX7C	-0.591688198	5.50E-05
ENSG00000175061	LRRC75A-AS1	-0.712997209	5.70E-05
ENSG00000187514	PTMA	-0.66370439	5.74E-05
ENSG00000164692	COL1A2	-5.311908165	5.74E-05
ENSG00000107201	DDX58	1.948164449	5.74E-05
ENSG00000247774	PCED1B-AS1	-1.138208676	5.74E-05
ENSG00000124172	ATP5E	-0.564331315	5.74E-05
ENSG00000184979	USP18	3.862304262	5.74E-05
ENSG00000232112	TMA7	-0.607479602	6.26E-05
ENSG00000183691	NOG	-3.790342064	6.33E-05
ENSG00000157570	TSPAN18	-3.930782241	6.38E-05
ENSG00000054654	SYNE2	-1.438675458	6.38E-05
ENSG00000163682	RPL9	-0.837035041	6.56E-05
ENSG00000137078	SIT1	-3.410523475	6.70E-05
ENSG00000100453	GZMB	-3.48671265	7.07E-05
ENSG00000138646	HERC5	2.73760257	7.23E-05
ENSG00000268205		-0.947871769	7.25E-05
ENSG00000167526	RPL13	-0.838321988	7.45E-05
ENSG00000168118	RAB4A	-0.570659018	8.24E-05
ENSG00000198934	MAGEE1	-4.44609927	8.30E-05
ENSG00000107223	EDF1	-0.640913802	8.32E-05
ENSG00000109475	RPL34	-0.774306329	8.49E-05
ENSG00000123349	PFDN5	-0.650102849	8.60E-05
ENSG00000269293	ZSCAN16-AS1	-0.895664556	8.93E-05

ENSG00000147123	NDUFB11	-0.704269437	8.93E-05
ENSG00000175854	SWI5	-0.989809919	9.57E-05
ENSG00000111912	NCOA7	1.315900313	9.87E-05
ENSG00000102030	NAA10	-0.832584601	1.00E-04
ENSG00000115267	IFIH1	1.787770848	0.000101806
ENSG00000280237	MIR4697HG	-4.082282477	0.000103373
ENSG00000213904	LIPE-AS1	-1.310858245	0.000103724
ENSG00000132185	FCRLA	-3.311934728	0.000104026
ENSG00000237943	PRKCQ-AS1	-1.856660035	0.000104932
ENSG00000213139	CRYGS	-1.280665004	0.000105641
ENSG00000259959		-1.394450458	0.000105641
ENSG00000168646	AXIN2	-4.548723571	0.000105641
ENSG00000163520	FBLN2	-2.726185674	0.000105795
ENSG00000111331	OAS3	2.922369795	0.000106087
ENSG00000172432	GTPBP2	0.835450041	0.000108081
ENSG00000196683	TOMM7	-0.829828497	0.00010827
ENSG00000104529	EEF1D	-0.58691008	0.000111495
ENSG00000165138	ANKS6	-1.514079768	0.000111495
ENSG00000213862		-0.801439697	0.000111495
ENSG00000105699	LSR	-3.418783114	0.000111731
ENSG00000155366	RHOC	-1.637991254	0.000114139
ENSG00000174500	GCSAM	-3.917508071	0.000114179
ENSG00000280120		-1.261390784	0.000114214
ENSG00000100027	YPEL1	-1.703045367	0.000114214
ENSG00000157601	MX1	2.59560948	0.000114214
ENSG00000113088	GZMK	-3.822962222	0.00011818
ENSG00000274383		-1.542970616	0.000120276
ENSG00000161970	RPL26	-0.715400108	0.000130254
ENSG00000164615	CAMLG	-0.629958807	0.000130772
ENSG00000065717	TLE2	-3.347003756	0.000131315
ENSG00000167747	C19orf48	-1.049405836	0.000132886
ENSG00000182004	SNRPE	-0.713832565	0.000136285
ENSG00000172943	PHF8	0.538152116	0.000136865
ENSG00000139537	CCDC65	-4.443297198	0.000141543
ENSG00000122026	RPL21	-0.723086137	0.000141632
ENSG00000130332	LSM7	-0.746420001	0.000147483
ENSG00000133112	TPT1	-0.658410948	0.000148679
ENSG00000231113		-0.7788648	0.000151269
ENSG00000185163	DDX51	-0.687193113	0.000158872
ENSG00000134326	CMPK2	2.977159049	0.000159499
ENSG00000169884	WNT10B	-2.333576677	0.000159516
ENSG00000102878	HSF4	-1.147820473	0.000159808
ENSG00000133065	SLC41A1	-0.89957492	0.000163039
ENSG00000183648	NDUFB1	-0.728642114	0.000163039
ENSG00000154764	WNT7A	-4.449363065	0.000165811
ENSG00000236778	INTS6-AS1	-1.077927255	0.000185365
ENSG00000260807		-3.673407639	0.000185618
ENSG00000099624	ATP5D	-0.832580335	0.000188578

ENSG00000145912	NHP2	-0.773872402	0.000192316
ENSG00000168824		-3.494911903	0.000197172
ENSG00000204628	RACK1	-0.537567004	0.000197172
ENSG00000104907	TRMT1	-0.73790355	0.000209028
ENSG00000237550	RPL9P9	-0.746466662	0.000219689
ENSG00000213015	ZNF580	-0.888078276	0.000223953
ENSG00000172663	TMEM134	-0.797327479	0.000232242
ENSG00000270189		-1.463036366	0.000238693
ENSG00000183918	SH2D1A	-2.864633564	0.000238693
ENSG00000174886	NDUFA11	-1.04574285	0.000238693
ENSG00000269044		-1.037537922	0.000238693
ENSG00000108679	LGALS3BP	2.291162548	0.000241285
ENSG00000172878	METAP1D	-1.448666428	0.000241285
ENSG00000111907	TPD52L1	2.690874455	0.000248813
ENSG00000105939	ZC3HAV1	0.933584465	0.000251153
ENSG00000155265	GOLGA7B	-3.06933314	0.000262446
ENSG00000105427	CNFN	-2.164900281	0.00026337
ENSG00000167799	NUDT8	-1.956469458	0.000267099
ENSG00000213757		-1.127686732	0.000267232
ENSG00000102471	NDFIP2	-3.956319941	0.000280208
ENSG00000184990	SIVA1	-0.726001441	0.000286118
ENSG00000147804	SLC39A4	-1.101649423	0.000286208
ENSG00000234797	RPS3AP6	-0.778314029	0.000290261
ENSG00000106355	LSM5	-0.689110229	0.000305367
ENSG00000167283	ATP5L	-0.496159342	0.000305367
ENSG00000214548	MEG3	-3.07822972	0.000307298
ENSG00000235552	RPL6P27	-0.861926108	0.000307298
ENSG00000278030	TRBV7-9	-4.25691186	0.000307447
ENSG00000211749	TRBV23-1	-4.263401984	0.000309205
ENSG00000174946	GPR171	-3.539090325	0.00031141
ENSG00000158321	AUTS2	-2.087307202	0.00031141
ENSG00000148516	ZEB1	-2.393228406	0.000314244
ENSG00000067066	SP100	0.896173461	0.000316288
ENSG00000105193	RPS16	-0.672198552	0.000324618
ENSG00000241837	ATP5O	-0.613730551	0.000329148
ENSG00000221983	UBA52	-0.599121868	0.000329169
ENSG00000140391	TSPAN3	-1.060016498	0.000334674
ENSG00000156411	C14orf2	-0.615265546	0.000336796
ENSG00000007264	MATK	-2.859583582	0.000339424
ENSG00000162244	RPL29	-0.673454697	0.000347712
ENSG00000130635	COL5A1	-3.550996646	0.000352748
ENSG00000212694	LINC01089	-1.197254106	0.000352748
ENSG00000160226	C21orf2	-1.088532364	0.000360717
ENSG00000101596	SMCHD1	0.931369794	0.000360786
ENSG00000156467	UQCRB	-0.645589241	0.000361718
ENSG00000164483	SAMD3	-2.564111255	0.000362078
ENSG00000115155	OTOF	7.186284502	0.000364835
ENSG00000148362	C9orf142	-0.847290552	0.000365754

ENSG00000103266	STUB1	-0.588735726	0.000365782
ENSG00000248275	TRIM52-AS1	-0.964355792	0.000369752
ENSG00000110700	RPS13	-0.622623297	0.000374754
ENSG00000180530	NRIP1	1.351034401	0.000376561
ENSG00000163534	FCRL1	-2.617711203	0.000377729
ENSG00000211677	IGLC2	-3.642816904	0.000378943
ENSG00000117322	CR2	-2.515040768	0.000380931
ENSG00000204922	UQCC3	-1.013559247	0.000383374
ENSG00000240509	RPL34P18	-1.494481611	0.000387277
ENSG00000162227	TAF6L	-0.827682662	0.000398126
ENSG00000152778	IFIT5	2.080060897	0.000403181
ENSG00000166428	PLD4	-2.205792302	0.000407427
ENSG00000186049	KRT73	-3.932644419	0.00041245
ENSG00000144218	AFF3	-1.392336948	0.000416883
ENSG00000112306	RPS12	-0.703565345	0.000419577
ENSG00000163820	FYCO1	0.809399307	0.000423912
ENSG00000137198	GMPR	3.072909968	0.000431438
ENSG00000172183	ISG20	1.501926779	0.000431438
ENSG00000156873	PHKG2	-0.586125261	0.0004467
ENSG00000138642	HERC6	2.011489193	0.000453786
ENSG00000153563	CD8A	-3.217171453	0.000462698
ENSG00000198087	CD2AP	1.153941069	0.000471446
ENSG00000189227	C15orf61	-0.916327124	0.000482372
ENSG00000135426	TESPA1	-1.379724059	0.000488308
ENSG00000136942	RPL35	-0.690777745	0.000488637
ENSG00000276136		-1.561701407	0.000488637
ENSG00000128185	DGCR6L	-0.869029126	0.000492227
ENSG00000213553	RPLP0P6	-0.732793466	0.000500474
ENSG00000172116	CD8B	-2.758943834	0.000501998
ENSG00000168002	POLR2G	-0.55077571	0.000501998
ENSG00000117691	NENF	-0.912177248	0.000502636
ENSG00000197756	RPL37A	-0.68148802	0.000502636
ENSG00000204257	HLA-DMA	-1.149520954	0.000502636
ENSG00000225964	NRIR	3.248312012	0.000508147
ENSG00000005075	POLR2J	-0.660280637	0.000508147
ENSG00000162614	NEXN	2.732093552	0.000512538
ENSG00000141698	NT5C3B	-1.203616145	0.00051257
ENSG00000256338	RPL41P2	-1.490949053	0.000531511
ENSG00000161381	PLXDC1	-1.793972135	0.000532809
ENSG00000111335	OAS2	2.436463858	0.000537786
ENSG00000099385	BCL7C	-0.660592553	0.000545778
ENSG00000104814	MAP4K1	-0.858644073	0.000551874
ENSG00000198168	SVIP	-1.080144347	0.000552879
ENSG00000130731	METTL26	-0.897489836	0.000559458
ENSG00000185920	PTCH1	-2.347887924	0.000580108
ENSG00000211679	IGLC3	-3.788516443	0.000584189
ENSG00000164442	CITED2	1.083600372	0.000585482
ENSG00000197696	NMB	-1.362725379	0.000593211

ENSG00000274307		1.616351746	0.000603422
ENSG00000235621	LINC00494	-4.274984155	0.000603422
ENSG00000181163	NPM1	-0.601003416	0.000639249
ENSG00000272034	SNORD14A	-1.193770842	0.000653186
ENSG00000225828	FAM229A	-0.830548392	0.000657598
ENSG00000137441	FGFBP2	-3.790425572	0.00065815
ENSG00000175390	EIF3F	-0.52428776	0.000659607
ENSG00000125743	SNRPD2	-0.526163585	0.000659607
ENSG00000076554	TPD52	-1.722912955	0.000679518
ENSG00000042088	TDP1	-0.598070112	0.0006851
ENSG00000137959	IFI44L	3.262678279	0.000694365
ENSG00000103024	NME3	-1.112249155	0.000694365
ENSG00000214253	FIS1	-0.573966318	0.000698112
ENSG00000239569	KMT2E-AS1	-0.986097199	0.000698112
ENSG00000115239	ASB3	-0.691984058	0.000708812
ENSG00000123144	C19orf43	-0.601773846	0.000714047
ENSG00000002549	LAP3	1.527329536	0.000734181
ENSG00000166405	RIC3	-1.953717093	0.000734181
ENSG00000156738	MS4A1	-2.687154081	0.000734181
ENSG00000178449	COX14	-0.790579915	0.000734181
ENSG00000082458	DLG3	-3.029361896	0.000735233
ENSG00000229677		2.37648562	0.000740348
ENSG00000229833	PET100	-0.833837555	0.000788199
ENSG00000273271		-1.13364832	0.000788199
ENSG00000130520	LSM4	-0.698924172	0.000789245
ENSG00000272990		-1.594571719	0.000792484
ENSG00000211789	TRAV12-2	-3.449639215	0.000796876
ENSG00000122224	LY9	-1.779083331	0.00080638
ENSG00000137818	RPLP1	-0.689717968	0.00080638
ENSG00000230124	ACBD6	-0.56347087	0.000809756
ENSG00000176533	GNG7	-1.254440406	0.000809756
ENSG00000268836		-2.221562663	0.000835904
ENSG00000059378	PARP12	1.067986187	0.000839654
ENSG00000170542	SERPINB9	1.265338028	0.000839834
ENSG00000008988	RPS20	-0.62137657	0.000845933
ENSG00000198816	ZNF358	-1.02355722	0.000845933
ENSG00000232788		-1.903169505	0.000850641
ENSG00000172053	QARS	-0.433830254	0.000851892
ENSG00000174915	PTDSS2	-0.581039639	0.000851892
ENSG00000174171		-2.84227074	0.000851892
ENSG00000134419	RPS15A	-0.597579856	0.000851892
ENSG00000198931	APRT	-0.698983586	0.000851892
ENSG00000244398		-0.728765348	0.000853682
ENSG00000028116	VRK2	0.916992924	0.00085393
ENSG00000169976	SF3B5	-0.566241373	0.000857732
ENSG00000171159	C9orf16	-0.723287904	0.000869224
ENSG00000206190	ATP10A	1.778549959	0.000877263
ENSG00000167113	COQ4	-0.819426269	0.000895068

ENSG00000166289	PLEKHF1	-2.423207789	0.000895068
ENSG00000186854	TRABD2A	-1.505448806	0.000896599
ENSG00000101298	SNPH	-2.019688124	0.000896599
ENSG00000171858	RPS21	-0.733252591	0.000906927
ENSG00000117859	OSBPL9	0.671279343	0.000915519
ENSG00000181381	DDX60L	1.480272687	0.000925248
ENSG00000226287	TMEM191A	-1.398998211	0.000925248
ENSG00000007392	LUC7L	-0.623415398	0.000930727
ENSG00000278942		-4.04277027	0.000932838
ENSG00000274752	TRBV12-3	-4.062410555	0.000936739
ENSG00000143793	C1orf35	-0.81751732	0.000944431
ENSG00000111224	PARP11	0.981807815	0.00094515
ENSG00000120915	EPHX2	-2.782795904	0.000945858
ENSG00000139343	SNRPF	-0.622475292	0.000945858
ENSG00000272256		-1.282807815	0.000947051
ENSG00000226660	TRBV2	-4.050964885	0.000950942
ENSG00000197111	PCBP2	-0.510434783	0.000950942
ENSG00000063177	RPL18	-0.629860584	0.000950942
ENSG00000244313		-0.726036477	0.000951188
ENSG00000272579		-1.283930549	0.000955848
ENSG00000262528		-1.513211868	0.000964192
ENSG00000145016	RUBCN	0.692873429	0.000974716
ENSG00000092098	RNF31	0.817304263	0.000980019
ENSG00000211949	IGHV3-23	-3.661538369	0.000981957
ENSG00000133106	EPSTI1	2.03174243	0.000995216
ENSG00000141759	TXNL4A	-0.540128229	0.001014911
ENSG00000147403	RPL10	-0.620919688	0.001027031
ENSG00000166136	NDUFB8	-0.595560865	0.001048067
ENSG00000186010	NDUFA13	-0.763046639	0.001048067
ENSG00000100353	EIF3D	-0.370004705	0.001048067
ENSG00000161016	RPL8	-0.58028892	0.001048281
ENSG00000241351	IGKV3-11	-3.814088215	0.001065742
ENSG00000105402	NAPA	0.89282043	0.001066532
ENSG00000142541	RPL13A	-0.58764181	0.001066532
ENSG00000245910	SNHG6	-0.615873018	0.001074624
ENSG00000211655	IGLV1-36	-3.925276528	0.001093393
ENSG00000205307	SAP25	-1.34213169	0.001094842
ENSG00000099797	TECR	-0.791804103	0.001095487
ENSG00000155313	USP25	0.705780993	0.001095487
ENSG00000100129	EIF3L	-0.647889814	0.001105604
ENSG00000224094	RPS24P8	-0.845518034	0.001110634
ENSG00000227678		-4.069594174	0.001110634
ENSG00000121060	TRIM25	1.051969862	0.001112355
ENSG00000145494	NDUFS6	-0.838805501	0.001122967
ENSG00000242299		-0.853538668	0.001137483
ENSG00000246898	LINC00920	-1.973204412	0.001137483
ENSG00000054148	PHPT1	-0.936621982	0.001139699
ENSG00000264608		-1.105491679	0.001179681

ENSG00000130748	TMEM160	-1.256579576	0.001182756
ENSG00000143727	ACP1	-0.490573907	0.001184478
ENSG00000187051	RPS19BP1	-0.659686861	0.001184478
ENSG00000152558	TMEM123	1.156386842	0.00120837
ENSG00000237886	NALT1	-1.376118894	0.001230807
ENSG00000188878	FBF1	-1.113251078	0.001277927
ENSG00000223865	HLA-DPB1	-1.455681856	0.001294508
ENSG00000273361		-1.249836565	0.001302778
ENSG00000180354	MTURN	-0.956829225	0.001319483
ENSG00000211694	TRGV10	-4.059447193	0.00131964
ENSG00000115523	GNLY	-3.205977592	0.001323716
ENSG00000165821	SALL2	-4.057857259	0.001332566
ENSG00000109686	SH3D19	-3.056590787	0.001353039
ENSG00000149273	RPS3	-0.632249355	0.001353039
ENSG00000269858	EGLN2	-0.637138193	0.001353039
ENSG00000140905	GCSH	-1.704869283	0.001353462
ENSG00000177600	RPLP2	-0.744696481	0.001367523
ENSG00000260257		-0.972711331	0.001385133
ENSG00000025770	NCAPH2	-0.881499361	0.001385133
ENSG00000071655	MBD3	-0.657395733	0.001386439
ENSG00000255135		-1.419252405	0.001386508
ENSG00000167515	TRAPPC2L	-0.592403767	0.001394041
ENSG00000278784		-0.904804063	0.001399262
ENSG00000186501	TMEM222	-0.588097508	0.001417079
ENSG00000205078	SYCE1L	-1.430591716	0.001426743
ENSG00000002330	BAD	-0.647117355	0.001435023
ENSG00000197457	STMN3	-1.145236243	0.00144081
ENSG00000211899	IGHM	-2.574942964	0.001449424
ENSG00000141577	CEP131	-0.74588634	0.001449424
ENSG00000100380	ST13	-0.484762754	0.001451092
ENSG00000188786	MTF1	0.811727258	0.001452303
ENSG00000198918	RPL39	-0.644881892	0.001472786
ENSG00000228205		-1.216856117	0.001492991
ENSG00000133134	BEX2	-2.145700919	0.001503998
ENSG00000170074	FAM153A	-2.953352319	0.001508862
ENSG00000164919	COX6C	-0.595258792	0.001508862
ENSG00000166441	RPL27A	-0.614488637	0.001508862
ENSG00000100220	RTCB	0.905331566	0.00151378
ENSG00000100450	GZMH	-3.724857618	0.001518872
ENSG00000091129	NRCAM	-4.253359495	0.001526301
ENSG00000149541	B3GAT3	-0.625789499	0.001532483
ENSG00000189171	S100A13	-1.114933609	0.001536871
ENSG00000189046	ALKBH2	-0.919972205	0.001541163
ENSG00000091640	SPAG7	-0.4975212	0.001541352
ENSG00000100575	TIMM9	-0.659123505	0.001545275
ENSG00000130313	PGLS	-0.612708684	0.001561499
ENSG00000110200	ANAPC15	-0.562857539	0.001565445
ENSG00000182899	RPL35A	-0.605205841	0.001591652

ENSG00000215021	PHB2	-0.430860393	0.001618179
ENSG00000187630	DHRS4L2	-0.946029428	0.001626834
ENSG00000155657	TTN	-1.78681676	0.00162855
ENSG00000131591	C1orf159	-0.688736972	0.001632085
ENSG00000169020	ATP5I	-0.623640967	0.001633719
ENSG00000105197	TIMM50	-0.491195493	0.001635692
ENSG00000213339	QTRT1	-0.749302748	0.001654195
ENSG00000211898	IGHD	-2.55267733	0.001672761
ENSG00000174292	TNK1	-3.290263786	0.001681447
ENSG00000229659	RPL26P6	-0.965676181	0.001699132
ENSG00000186230	ZNF749	-1.364432637	0.001699132
ENSG00000163564	PYHIN1	-1.984386029	0.001699953
ENSG00000237976		-1.509758139	0.00171109
ENSG00000138496	PARP9	1.801034041	0.00171727
ENSG00000211750	TRBV24-1	-4.048281756	0.00171727
ENSG00000198695	MT-ND6	-0.986855952	0.00171727
ENSG00000085563	ABCB1	-3.433711933	0.001729403
ENSG00000170581	STAT2	1.156446139	0.001736038
ENSG00000117475	BLZF1	0.791724459	0.001740117
ENSG00000123989	CHPF	-3.450819145	0.001745049
ENSG00000132661	NXT1	-1.000164202	0.001749114
ENSG00000242485	MRPL20	-0.484480762	0.001759918
ENSG00000214706	IFRD2	-0.695175693	0.001759918
ENSG00000017483	SLC38A5	1.894708707	0.001759918
ENSG00000137054	POLR1E	-0.762689218	0.001759918
ENSG00000180089	TMEM86B	-0.912626736	0.001759918
ENSG00000184207	PGP	-0.719111418	0.001782345
ENSG00000170634	ACYP2	-0.749196686	0.001789678
ENSG00000119514	GALNT12	-2.139334428	0.001789678
ENSG00000163644	PPM1K	1.329797369	0.001799887
ENSG00000233927	RPS28	-0.673091597	0.001822475
ENSG00000204946	ZNF783	-0.64326063	0.001836448
ENSG00000171863	RPS7	-0.500801482	0.001853573
ENSG00000165688	PMPCA	-0.518455131	0.001853573
ENSG00000186184	POLR1D	-0.488676474	0.00185704
ENSG00000244723	ASLP1	-1.637695406	0.0018602
ENSG00000162066	AMDHD2	-0.742738166	0.001864273
ENSG00000058804	NDC1	-0.751211513	0.001888927
ENSG00000149809	TM7SF2	-0.878526484	0.001888927
ENSG00000250479	CHCHD10	-0.841421525	0.001888927
ENSG00000172543	CTSW	-1.924427039	0.00190583
ENSG00000128951	DUT	-0.892362449	0.001927018
ENSG00000100075	SLC25A1	-0.706864944	0.001937437
ENSG00000242574	HLA-DMB	-1.19739345	0.001963359
ENSG00000169100	SLC25A6	-0.505082516	0.001972833
ENSG00000204228	HSD17B8	-1.232960818	0.001985164
ENSG00000243147	MRPL33	-0.58097028	0.001987346
ENSG00000135617	PRADC1	-0.947177642	0.001987346

ENSG00000136938	ANP32B	-0.585334749	0.001987346
ENSG00000183508	FAM46C	-2.505402765	0.002012319
ENSG00000132465	JCHAIN	-3.097013532	0.002012319
ENSG00000163794	UCN	-1.346560013	0.002061377
ENSG00000135899	SP110	1.209762012	0.002061377
ENSG00000262089		-0.93514743	0.002068321
ENSG00000240230	COX19	-0.510066253	0.002092485
ENSG00000198604	BAZ1A	0.566140772	0.002092485
ENSG00000105726	ATP13A1	0.743610974	0.002092618
ENSG00000171960	PPIH	-0.601896186	0.002109336
ENSG00000243199		-1.051029871	0.002109336
ENSG00000151623	NR3C2	-1.587172362	0.002116577
ENSG00000211689	TRGC1	-2.732172873	0.002134044
ENSG00000247317		2.126325574	0.002134044
ENSG00000272030		-1.234836076	0.002137164
ENSG00000105974	CAV1	-4.932177264	0.002165857
ENSG00000188747	NOXA1	-1.068235379	0.002165857
ENSG00000148950	IMMP1L	-1.172082041	0.002189815
ENSG00000103528	SYT17	-1.790244717	0.002222068
ENSG00000104936	DMPK	-1.255140736	0.002234058
ENSG00000232472	EEF1B2P3	-0.83829887	0.00224768
ENSG00000134262	AP4B1	-0.568181244	0.002276349
ENSG00000137288	UQCC2	-1.038190718	0.002276349
ENSG00000145390	USP53	-1.876875698	0.002284196
ENSG00000234009	RPL5P34	-0.879525116	0.002286318
ENSG00000257303		-1.543800209	0.00229383
ENSG00000176476	SGF29	-0.599437713	0.002295797
ENSG00000198258	UBL5	-0.47887206	0.002307212
ENSG00000211648	IGLV1-47	-3.188128985	0.002307212
ENSG00000006015	C19orf60	-0.840431313	0.00231205
ENSG00000206828		-1.377334777	0.002320347
ENSG00000272195		-0.978505399	0.002325887
ENSG00000140988	RPS2	-0.607745473	0.002336322
ENSG00000104852	SNRNP70	-0.615869688	0.002339089
ENSG00000173486	FKBP2	-0.860658965	0.002356163
ENSG00000130204	TOMM40	-0.570189763	0.002387003
ENSG00000179331	RAB39A	1.698831443	0.002418351
ENSG00000130487	KLHDC7B	1.733113513	0.002424667
ENSG00000008018	PSMB1	-0.464907359	0.002496126
ENSG00000030419	IKZF2	-1.919486819	0.002510426
ENSG00000228434		-1.063039478	0.002516966
ENSG00000177409	SAMD9L	2.423278265	0.002523246
ENSG00000167633	KIR3DL1	-4.223411078	0.002529204
ENSG00000146215	CRIP3	-1.406669613	0.002559541
ENSG00000171425	ZNF581	-0.719104478	0.002561286
ENSG00000162576	MXRA8	-3.572319884	0.002566536
ENSG00000185418	TARSL2	-0.784803254	0.002566536
ENSG00000145741	BTF3	-0.461851102	0.002581089

ENSG00000122643	NT5C3A	1.317325129	0.002585847
ENSG00000125991	ERGIC3	-0.43800389	0.00260207
ENSG00000071859	FAM50A	-0.476306782	0.002616467
ENSG00000281404	LINC01176	-1.164551407	0.002618652
ENSG00000075568	TMEM131	0.535251034	0.002629514
ENSG00000081052	COL4A4	-4.037508604	0.002629579
ENSG00000242060	RPS3AP49	-1.273964318	0.002629579
ENSG00000110011	DNAJC4	-0.516651123	0.002648646
ENSG00000186020	ZNF529	-0.908780081	0.002662185
ENSG00000226777	FAM30A	-2.661282073	0.002668113
ENSG00000137965	IFI44	2.196433674	0.002683332
ENSG00000139428	MMAB	-0.733197563	0.002699017
ENSG00000272888	LINC01578	-0.772752541	0.002739942
ENSG00000144713	RPL32	-0.600024025	0.002749482
ENSG00000154102	C16orf74	-1.258071379	0.002751801
ENSG00000185722	ANKFY1	0.737371206	0.002751801
ENSG00000142676	RPL11	-0.566461908	0.002778411
ENSG00000232629	HLA-DQB2	-1.916052661	0.002778411
ENSG00000119943	PYROXD2	-0.930166582	0.002778411
ENSG00000170430	MGMT	-0.954731592	0.002778411
ENSG00000168395	ING5	-0.750204282	0.002818468
ENSG00000251580		-1.315980363	0.002818468
ENSG00000072121	ZFYVE26	0.593596358	0.002835307
ENSG00000163430	FSTL1	-2.806135045	0.002849376
ENSG00000071082	RPL31	-0.569869918	0.002885416
ENSG00000163599	CTLA4	-3.272974814	0.002888091
ENSG00000215845	TSTD1	-0.942904412	0.002929217
ENSG00000244720		-3.810560581	0.002931755
ENSG00000107719	PALD1	-2.691280411	0.002936921
ENSG00000125652	ALKBH7	-0.790550229	0.002949662
ENSG00000205544	TMEM256	-0.88921173	0.002965688
ENSG00000101546	RBFA	-0.802965533	0.002975178
ENSG00000092203	TOX4	0.462109871	0.002987653
ENSG00000108771	DHX58	1.375746139	0.002995762
ENSG00000185745	IFIT1	3.378298653	0.002996848
ENSG00000267281		-1.02312114	0.002996848
ENSG00000211785	TRAV12-1	-3.81000108	0.003005494
ENSG00000163666	HESX1	2.753459921	0.003040557
ENSG00000131116	ZNF428	-0.597583836	0.00310363
ENSG00000211794	TRAV12-3	-3.809812145	0.003111502
ENSG00000176083	ZNF683	-4.031645831	0.003114556
ENSG00000172936	MYD88	0.759411381	0.003130104
ENSG00000235043	TECRP1	-1.250033025	0.003145723
ENSG00000130255	RPL36	-0.690341816	0.003152721
ENSG00000149806	FAU	-0.530142028	0.003164132
ENSG00000234327		-1.578682041	0.003182264
ENSG00000211795	TRAV8-6	-3.806193568	0.003187335
ENSG00000125691	RPL23	-0.560708956	0.003228424

ENSG00000167930	FAM234A	-0.612626129	0.003239193
ENSG00000100823	APEX1	-0.557053616	0.003261212
ENSG00000103260	METRN	-1.248967867	0.003305281
ENSG00000211598	IGKV4-1	-3.558413387	0.003341618
ENSG00000005022	SLC25A5	-0.636064927	0.003350222
ENSG00000272556	GTF2IP13	-1.18145431	0.003351949
ENSG00000167543	TP53I13	-0.775157309	0.003388679
ENSG00000260911		-1.447742281	0.003399717
ENSG00000258472		-0.78515948	0.003399717
ENSG00000100416	TRMU	-0.598521785	0.00344042
ENSG00000124614	RPS10	-0.628770672	0.003442988
ENSG00000281205	LINC00950	-1.386752916	0.003442988
ENSG00000167393	PPP2R3B	-0.984206651	0.003516662
ENSG00000135469	COQ10A	-0.898849317	0.003524877
ENSG00000211788	TRAV13-1	-3.475946962	0.003561299
ENSG00000221957	KIR2DS4	-4.037811821	0.003607637
ENSG00000138326	RPS24	-0.569181556	0.003618007
ENSG00000273338		-1.383005876	0.003621488
ENSG00000235194	PPP1R3E	-0.914048074	0.003666741
ENSG00000173660	UQCRH	-0.468939113	0.003687002
ENSG00000168229	PTGDR	-3.293510903	0.003687002
ENSG00000120885	CLU	2.915827551	0.003687796
ENSG00000105258	POLR2I	-0.775322734	0.003742581
ENSG00000211803	TRAV23DV6	-3.464460059	0.003756506
ENSG00000115760	BIRC6	0.631050579	0.003762099
ENSG00000172590	MRPL52	-0.718302792	0.003762099
ENSG00000042753	AP2S1	-0.729217046	0.003762099
ENSG00000108984	MAP2K6	1.423757981	0.003814168
ENSG00000099977	DDT	-0.739303715	0.003837421
ENSG00000130813	C19orf66	0.869096867	0.00385685
ENSG00000105519	CAPS	-0.845084843	0.003862811
ENSG00000180543	TSPYL5	-1.166334836	0.003887091
ENSG00000224631	RPS27AP16	-0.675220599	0.003887091
ENSG00000115274	INO80B	-0.895649136	0.003897304
ENSG00000105088	OLFM2	-3.790403614	0.003897304
ENSG00000100227	POLDIP3	0.450905706	0.003897304
ENSG00000148291	SURF2	-0.655670304	0.003906084
ENSG00000237772		-1.439841811	0.003966043
ENSG00000164649	CDCA7L	-1.040443289	0.003975205
ENSG00000257093	KIAA1147	-0.727090515	0.003975205
ENSG00000228444		-2.247101102	0.004099374
ENSG00000211956	IGHV4-34	-3.138745881	0.004123423
ENSG00000260910	LINC00565	-2.529063483	0.004166361
ENSG00000152795	HNRNPDL	-0.421574064	0.004191667
ENSG00000150401	DCUN1D2	-0.551935879	0.00424472
ENSG00000136643	RPS6KC1	0.551647774	0.004367828
ENSG00000254709	IGLL5	-3.286423802	0.004398542
ENSG00000117010	ZNF684	1.02726853	0.004400343

ENSG00000177455	CD19	-1.77450544	0.004400343
ENSG00000242125	SNHG3	-0.718633146	0.00441103
ENSG00000119917	IFIT3	3.31607972	0.00444772
ENSG00000173581	CCDC106	-1.069160411	0.004512892
ENSG00000232346		-1.400608068	0.004629858
ENSG00000054523	KIF1B	0.867949541	0.004663531
ENSG00000166435	XRRA1	2.277636235	0.004756594
ENSG00000274605		-1.617476553	0.004783684
ENSG00000101391	CDK5RAP1	-0.441121525	0.004823892
ENSG00000095906	NUBP2	-0.648566954	0.004902994
ENSG00000279605		-1.817388061	0.004916951
ENSG00000275413		-1.692304565	0.004916951
ENSG00000091409	ITGA6	-1.554797367	0.004926076
ENSG00000269893	SNHG8	-0.915979704	0.004930682
ENSG00000115415	STAT1	1.631028864	0.005030293
ENSG00000260676	LINC01541	1.909867909	0.005030293
ENSG00000158552	ZFAND2B	-0.459763435	0.005035996
ENSG00000007402	CACNA2D2	-2.329048542	0.005035996
ENSG00000145287	PLAC8	1.207345184	0.005076234
ENSG00000269951		-1.510942157	0.005100218
ENSG00000215375	MYL5	-1.026525087	0.005103966
ENSG00000150456	EEF1AKMT1	-1.268231703	0.005109127
ENSG00000171703	TCEA2	-0.622922133	0.00512065
ENSG00000149743	TRPT1	-0.74074439	0.005141504
ENSG00000119682	AREL1	0.520490859	0.005182733
ENSG00000125520	SLC2A4RG	-0.613552049	0.005196793
ENSG00000168393	DTYMK	-0.983196818	0.00522779
ENSG00000254911	SCARNA9	-1.088930455	0.00522779
ENSG00000197776	KLHDC1	-1.022240961	0.00522779
ENSG00000090238	YPEL3	-0.710406775	0.00522779
ENSG00000083457	ITGAE	-0.854994519	0.00522779
ENSG00000118418	HMGN3	-0.693139145	0.005287534
ENSG00000261662		-0.722111336	0.005305765
ENSG00000137310	TCF19	-1.450430084	0.005345435
ENSG00000228106		-1.161429656	0.005364505
ENSG00000238142		-1.12786702	0.005376731
ENSG00000147687	TATDN1	-0.651493085	0.005376731
ENSG00000163508	EOMES	-3.617920749	0.005394996
ENSG00000251023		-1.235655791	0.005404381
ENSG00000213397	HAUS7	-0.841543258	0.005488566
ENSG00000257621	PSMA3-AS1	-0.590866684	0.005488566
ENSG00000088356	PDRG1	-0.743535963	0.005488566
ENSG00000198646	NCOA6	0.530406081	0.005488566
ENSG00000141096	DPEP3	-1.338357677	0.005516815
ENSG00000211592	IGKC	-2.561833705	0.005550161
ENSG00000186265	BTLA	-2.090466358	0.005550161
ENSG00000161021	MAML1	0.557207852	0.005550161
ENSG00000197958	RPL12	-0.567477825	0.005550161

ENSG00000179632	MAF1	-0.45651211	0.005570885
ENSG00000136874	STX17	0.711504393	0.005613442
ENSG00000221963	APOL6	1.146832025	0.005650226
ENSG00000143515	ATP8B2	-1.126132109	0.005696364
ENSG00000176978	DPP7	-0.511238488	0.005696364
ENSG00000214026	MRPL23	-0.747557922	0.005701526
ENSG00000110497	AMBRA1	0.515659085	0.005701526
ENSG00000169992	NLGN2	-1.255464012	0.005710946
ENSG00000141858	SAMD1	-0.867063732	0.005719449
ENSG00000163840	DTX3L	1.463076465	0.005749765
ENSG00000182685	BRICD5	-1.163377704	0.005749765
ENSG00000188599	NPIPP1	-1.016073719	0.005771272
ENSG00000108961	RANGRF	-0.556180848	0.005771272
ENSG00000175756	AURKAIP1	-0.548553001	0.005780322
ENSG00000196116	TDRD7	0.978683141	0.005817583
ENSG00000112419	PHACTR2	0.984741176	0.005846391
ENSG00000119650	IFT43	-0.723296864	0.005953883
ENSG00000159958	TNFRSF13C	-1.733520596	0.005990995
ENSG00000263465	SRSF8	-0.619473845	0.006022803
ENSG00000143222	UFC1	-0.408895501	0.006047557
ENSG00000229809	ZNF688	-0.6131909	0.006102597
ENSG00000105640	RPL18A	-0.680237544	0.006102597
ENSG00000091972	CD200	-2.772195836	0.006115276
ENSG00000105968	H2AFV	-0.549793474	0.006115276
ENSG00000106133	NSUN5P2	-1.23934778	0.006115276
ENSG00000282034		-0.927210969	0.006115276
ENSG00000198242	RPL23A	-0.535653341	0.006115276
ENSG00000160050	CCDC28B	-1.127235447	0.006248614
ENSG00000131143	COX4I1	-0.615828106	0.006248614
ENSG00000125458	NT5C	-0.732257873	0.00625249
ENSG00000143297	FCRL5	-1.66640251	0.006257931
ENSG00000167671	UBXN6	-0.503331314	0.006275423
ENSG00000187912	CLEC17A	-1.71336925	0.006316004
ENSG00000133818	RRAS2	-2.12788443	0.00638121
ENSG00000211890	IGHA2	-3.768716107	0.00638121
ENSG00000170448	NFXL1	-0.96615348	0.006390453
ENSG00000279491		-1.341594364	0.006390453
ENSG00000154358	OBSCN	-1.116533745	0.006390892
ENSG00000175193	PARL	-0.42071223	0.006412079
ENSG00000164168	TMEM184C	0.675968208	0.006412079
ENSG00000242071	RPL7AP6	-0.507626688	0.006412079
ENSG00000170476	MZB1	-2.600215232	0.006412354
ENSG00000112651	MRPL2	-0.590654242	0.006412354
ENSG00000260711		-2.386545413	0.006412354
ENSG00000106268	NUDT1	-0.794925259	0.006430892
ENSG00000143365	RORC	-3.295107354	0.006448205
ENSG00000125877	ITPA	-0.629610102	0.006501465
ENSG00000189007	ADAT2	-0.67748119	0.006502166

ENSG00000239246		-0.967740524	0.006502166
ENSG00000277476		-0.84517529	0.006502166
ENSG00000262814	MRPL12	-1.126811985	0.006520707
ENSG00000109084	TMEM97	-1.565088168	0.006552117
ENSG00000105618	PRPF31	-0.320470529	0.006566947
ENSG00000164587	RPS14	-0.591232133	0.00656868
ENSG00000116983	HPCAL4	-2.564606275	0.006608617
ENSG00000234608	MAPKAPK5-AS1	-0.736441449	0.006608617
ENSG00000196814	MVB12B	1.194285859	0.006715757
ENSG00000167775	CD320	-1.157253018	0.006787393
ENSG00000160683	CXCR5	-2.663328355	0.006794946
ENSG00000173991	TCAP	-1.137628099	0.00684445
ENSG00000132530	XAF1	2.062417745	0.006941968
ENSG00000187801	ZFP69B	1.615322312	0.006976912
ENSG00000180739	S1PR5	-3.19314575	0.006996496
ENSG00000162739	SLAMF6	-1.609125103	0.007035685
ENSG00000106211	HSPB1	-0.86800819	0.007052093
ENSG00000177294	FBXO39	3.196448003	0.007101112
ENSG00000154451	GBP5	1.617552062	0.007108739
ENSG00000226396		-1.17527956	0.007132617
ENSG00000168297	PXK	0.61349204	0.007173042
ENSG00000204514	ZNF814	-0.862573314	0.00724662
ENSG00000159674	SPON2	-1.574587655	0.007250759
ENSG00000245954		-3.478701616	0.007250759
ENSG00000021355	SERPINB1	0.539946934	0.007250759
ENSG00000122140	MRPS2	-0.618453026	0.007250759
ENSG00000116898	MRPS15	-0.502582497	0.007261059
ENSG00000205336	ADGRG1	-2.665424849	0.007270512
ENSG00000165716	FAM69B	-3.63476677	0.007274028
ENSG00000137414	FAM8A1	0.78297763	0.007353886
ENSG00000178498	DTX3	-1.273556045	0.007353886
ENSG00000118523	CTGF	-4.009995666	0.007430689
ENSG00000011590	ZBTB32	-3.796516973	0.007439447
ENSG00000108298	RPL19	-0.459548471	0.007453444
ENSG00000119778	ATAD2B	0.632073686	0.007461982
ENSG00000230155		-0.878134987	0.007461982
ENSG00000188542	DUSP28	-0.532924972	0.007489458
ENSG00000115286	NDUFS7	-0.510651899	0.00750534
ENSG00000172809	RPL38	-0.586652241	0.007509344
ENSG00000159445	THEM4	-1.083386903	0.007513328
ENSG00000205581	HMGN1	-0.588019394	0.007529621
ENSG00000134371	CDC73	0.545193613	0.007577534
ENSG00000169738	DCXR	-0.796433956	0.007632459
ENSG00000114388	NPRL2	-0.606056714	0.007653417
ENSG00000182749	PAQR7	-0.988648959	0.00770553
ENSG00000139211	AMIGO2	1.112058967	0.007728162
ENSG00000170291	ELP5	-0.451100699	0.007728162
ENSG00000148308	GTF3C5	-0.38828101	0.007832655

ENSG00000211746	TRBV19	-3.261524859	0.007834932
ENSG00000235776		-0.570357062	0.007857373
ENSG00000146066	HIGD2A	-0.508846717	0.007858926
ENSG00000138615	CILP	-2.164202421	0.007881525
ENSG00000101337	TM9SF4	0.507323909	0.007915246
ENSG00000167658	EEF2	-0.433244129	0.007923108
ENSG00000071462	WBSCR22	-0.564191381	0.0079999
ENSG00000240972	MIF	-0.687637951	0.008014965
ENSG00000115159	GPD2	0.770871213	0.008019556
ENSG00000163867	ZMYM6	-0.49786665	0.00805075
ENSG00000163156	SCNM1	-0.469275116	0.00805075
ENSG00000218175		-1.081957236	0.00805075
ENSG00000272758		-0.94366131	0.00805075
ENSG00000258704	SRP54-AS1	-1.150107289	0.00805075
ENSG00000165516	KLHDC2	-0.546779085	0.00805075
ENSG00000170860	LSM3	-0.552215245	0.008084872
ENSG00000136444	RSAD1	-0.732249455	0.00814951
ENSG00000144161	ZC3H8	-0.700336371	0.008183046
ENSG00000137876	RSL24D1	-0.612271686	0.008266927
ENSG00000174721	FGFBP3	-1.051599989	0.008283057
ENSG00000254815		-2.522746154	0.008294645
ENSG00000063046	EIF4B	-0.58158112	0.008364561
ENSG00000144645	OSBPL10	-1.651067855	0.008398028
ENSG00000174547	MRPL11	-0.538131263	0.008398028
ENSG00000184436	THAP7	-0.612059548	0.008398028
ENSG00000235954	TTC28-AS1	-0.695683084	0.008443031
ENSG00000105372	RPS19	-0.567716037	0.008500143
ENSG00000143543	JTB	-0.42606116	0.008503651
ENSG00000273466		-0.940854962	0.008503651
ENSG00000161179	YDJC	-0.590714262	0.008595385
ENSG00000211734	TRBV5-1	-2.961716816	0.008627493
ENSG00000107872	FBXL15	-0.780552922	0.008627493
ENSG00000135114	OASL	2.577039865	0.008683968
ENSG00000170835	CEL	-1.690082517	0.008712379
ENSG00000089639	GMIP	0.424562652	0.008712379
ENSG00000158457	TSPAN33	-1.350702828	0.008751976
ENSG00000161395	PGAP3	-0.524266369	0.008751976
ENSG00000130684	ZNF337	-0.631394636	0.008751976
ENSG00000122218	COPA	0.670270176	0.008762951
ENSG00000205837	LINC00487	3.37091216	0.008762951
ENSG00000111879	FAM184A	-3.25943725	0.008762951
ENSG00000108515	ENO3	-0.830504914	0.008762951
ENSG00000196262	PPIA	-0.442714248	0.008768219
ENSG00000235065	RPL24P2	-0.966378673	0.00878134
ENSG00000272688		-1.227797565	0.008800031
ENSG00000133056	PIK3C2B	-0.981242108	0.008835169
ENSG00000199753	SNORD104	-1.304484153	0.008842684
ENSG00000161912	ADCY10P1	-0.791950249	0.008877956

ENSG00000182541	LIMK2	0.882307148	0.008877956
ENSG00000185839		1.374076737	0.008912945
ENSG00000272645	GTF2IP20	-0.761703148	0.008913296
ENSG00000220201	ZGLP1	-1.087373653	0.008952935
ENSG00000109046	WSB1	0.77860666	0.008980635
ENSG00000142552	RCN3	-1.137756026	0.008980635
ENSG00000254402	LRRC24	-1.138701897	0.00899864
ENSG00000167965	MLST8	-0.572348225	0.00899864
ENSG00000142534	RPS11	-0.481796891	0.00899864
ENSG00000178015	GPR150	-3.272440266	0.009045584
ENSG00000073536	NLE1	-0.906236214	0.009045584
ENSG00000188846	RPL14	-0.503545819	0.009050934
ENSG00000140612	SEC11A	-0.509756232	0.009075581
ENSG00000142684	ZNF593	-0.813535566	0.009136119
ENSG00000152082	MZT2B	-0.630803266	0.009147456
ENSG00000262919	FAM58A	-0.610755004	0.009147456
ENSG00000160932	LY6E	1.413642928	0.009350999
ENSG00000056972	TRAF3IP2	0.998618502	0.009460534
ENSG00000264812		-1.508854906	0.009474529
ENSG00000163584	RPL22L1	-0.677480246	0.009493084
ENSG00000196141	SPATS2L	2.044626281	0.009502716
ENSG00000004838	ZMYND10	-1.401441026	0.009527713
ENSG00000266677		-1.098088957	0.009584396
ENSG00000275056		-1.232632003	0.009590821
ENSG00000135390	ATP5G2	-0.451582539	0.00966023
ENSG00000233830	EIF4HP1	0.974566396	0.009667971
ENSG00000176490	DIRAS1	-3.326449375	0.009685261
ENSG00000146859	TMEM140	1.215696037	0.009686831
ENSG00000123545	NDUFAF4	-0.759418845	0.009694831
ENSG00000177595	PIDD1	-0.614003906	0.009983975
ENSG00000240342	RPS2P5	-0.667195142	0.009993581
ENSG00000214113	LYRM4	-0.589422964	0.009999842
ENSG00000149823	VPS51	-0.470844894	0.010006046
ENSG00000211787	TRAV8-3	-3.516730014	0.010017909
ENSG00000156482	RPL30	-0.504390862	0.010065681
ENSG00000211796	TRAV16	-3.513118229	0.010065732
ENSG00000230074		-0.989848975	0.010070915
ENSG00000155792	DEPTOR	-1.977539954	0.010071985
ENSG00000227191	TRGC2	-1.939551456	0.010072877
ENSG00000204272	LINC01420	-0.623368116	0.010084068
ENSG00000147576	ADHFE1	-0.987441136	0.010084068
ENSG00000230099	TRBV5-4	-3.512069871	0.010137044
ENSG00000106785	TRIM14	1.227136586	0.010137044
ENSG00000228775	WEE2-AS1	-2.286094723	0.01015285
ENSG00000161640	SIGLEC11	2.079317375	0.01015285
ENSG00000229638	RPL4P4	-0.573320279	0.010165869
ENSG00000188313	PLSCR1	1.414142798	0.01029454
ENSG00000211793	TRAV9-2	-3.519189578	0.01029454

ENSG00000109184	DCUN1D4	-0.617892413	0.010307042
ENSG00000235508	RPS2P7	-1.03657292	0.010393145
ENSG00000211792	TRAV14DV4	-3.522500562	0.010395799
ENSG00000271347		-2.509799967	0.010395799
ENSG00000127554	GFER	-0.520493058	0.010395799
ENSG00000115268	RPS15	-0.581637692	0.010395799
ENSG00000276557	TRBV18	-3.512673109	0.010537167
ENSG00000117519	CNN3	-3.145971047	0.010615606
ENSG00000250317	SMIM20	-0.680619236	0.010660228
ENSG00000211776	TRAV2	-3.514952937	0.010738187
ENSG00000111863	ADTRP	-2.187277092	0.010784797
ENSG00000204899	MZT1	-0.653170484	0.010790587
ENSG00000225756	DBH-AS1	-3.525047568	0.010809409
ENSG00000244701		-1.141700309	0.010855666
ENSG00000080854	IGSF9B	-3.528749915	0.010855666
ENSG00000211797	TRAV17	-3.264627863	0.010855666
ENSG00000140931	CMTM3	-0.492798671	0.010855666
ENSG00000267321		-0.803758289	0.010855666
ENSG00000105364	MRPL4	-0.518903762	0.010855666
ENSG00000198938	MT-CO3	-0.626052709	0.010855666
ENSG00000113532	ST8SIA4	0.784943979	0.010858983
ENSG00000147677	EIF3H	-0.338769488	0.010861233
ENSG00000104960	PTOV1	-0.576627852	0.010894709
ENSG00000211801	TRAV21	-3.511473158	0.010922375
ENSG00000068079	IFI35	1.15231457	0.010939216
ENSG00000135486	HNRNPA1	-0.417812673	0.010948901
ENSG00000273253		-1.081207127	0.010998336
ENSG00000231500	RPS18	-0.54357054	0.011034398
ENSG00000175866	BAIAP2	-0.813323971	0.011171871
ENSG00000136758	YME1L1	0.566346097	0.011177577
ENSG00000173726	TOMM20	-0.472514253	0.011191411
ENSG00000215030	RPL13P12	-1.140904216	0.011216558
ENSG00000211724	TRBV6-6	-3.502610175	0.011251369
ENSG00000132109	TRIM21	1.218594881	0.01129568
ENSG00000134644	PUM1	0.416965865	0.011327546
ENSG00000163050	COQ8A	-0.499149956	0.011327546
ENSG00000242324		2.037023439	0.011327546
ENSG00000269343	ZNF587B	-0.811509516	0.011327546
ENSG00000234785	EEF1GP5	-1.594136713	0.011399882
ENSG00000100307	CBX7	-0.812090482	0.011399882
ENSG00000113494	PRLR	1.385197353	0.011453567
ENSG00000258297		-0.806255263	0.011549089
ENSG00000248578	NPM1P21	-1.520715588	0.011676495
ENSG00000086065	CHMP5	0.869300596	0.011710083
ENSG00000236552	RPL13AP5	-0.548815384	0.011710083
ENSG00000212802	RPL15P3	-0.609619552	0.0117448
ENSG00000235576		-3.250263954	0.011771528
ENSG00000172534	HCFC1	0.54318123	0.011779969

ENSG00000143437	ARNT	0.526541535	0.011829351
ENSG00000205702	CYP2D7	-0.966229542	0.011829351
ENSG00000186918	ZNF395	-0.78480884	0.011842238
ENSG00000189409	MMP23B	-2.018668172	0.011881165
ENSG00000275491		-2.857909953	0.011949426
ENSG00000156795	WDYHV1	-0.734407989	0.012025556
ENSG00000267858	MZF1-AS1	-1.014687982	0.012056385
ENSG00000182512	GLRX5	-0.597080126	0.012067336
ENSG00000245970		-1.596422478	0.012070909
ENSG00000273344	PAXIP1-AS1	-1.015181513	0.012207509
ENSG00000273015		-0.561246381	0.012207509
ENSG00000178226	PRSS36	-0.930012719	0.012207509
ENSG00000234912	SNHG20	-0.938012453	0.01221256
ENSG00000079337	RAPGEF3	-2.175191126	0.012234211
ENSG00000273486		-1.151880901	0.012269658
ENSG00000140990	NDUFB10	-0.611648871	0.012285141
ENSG00000166750	SLFN5	1.722154134	0.012285141
ENSG00000136960	ENPP2	2.168941175	0.012334015
ENSG00000267469		-2.881437085	0.012334015
ENSG00000213928	IRF9	0.62902664	0.012341389
ENSG00000126709	IFI6	1.794015013	0.012377059
ENSG00000105700	KXD1	-0.311496337	0.012377059
ENSG00000237276	ANO7L1	1.687105099	0.012382767
ENSG00000130725	UBE2M	-0.471654901	0.012382767
ENSG00000213949	ITGA1	2.013415713	0.01247131
ENSG00000277998		-0.797598276	0.012473961
ENSG00000273314		1.270971866	0.012473961
ENSG00000167094	TTC16	-1.517629408	0.012473961
ENSG00000171222	SCAND1	-0.628919313	0.012473961
ENSG00000260260	SNHG19	-1.405936689	0.012517745
ENSG00000211965	IGHV3-49	-3.258177567	0.012580941
ENSG00000178467	P4HTM	-0.513301586	0.012592552
ENSG00000254470	AP5B1	0.835257258	0.012592552
ENSG00000229117	RPL41	-0.517651162	0.012611787
ENSG00000256053	APOPT1	-0.616681079	0.012634351
ENSG00000257704	INAFM1	-0.838635791	0.012684804
ENSG00000105518	TMEM205	-0.607528472	0.012707897
ENSG00000258944		-0.848551425	0.012751867
ENSG00000168056	LTBP3	-1.005572136	0.012775509
ENSG00000120896	SORBS3	-0.842239839	0.012851865
ENSG00000183542	KLRC4	-3.519342173	0.012898851
ENSG00000087237	CETP	-1.662371153	0.012929308
ENSG00000233762		-0.748191464	0.012948624
ENSG00000215769		-0.520146682	0.012948624
ENSG00000149927	DOC2A	-1.315887536	0.012984483
ENSG00000169087	HSPBAP1	-0.597915642	0.01307667
ENSG00000272449		-0.971077091	0.013084774
ENSG00000055118	KCNH2	-3.506917944	0.013084774

ENSG00000165795	NDRG2	-0.79173943	0.013084774
ENSG00000210151	MT-TS1	-1.737677285	0.013085773
ENSG00000065526	SPEN	0.563420163	0.013224068
ENSG00000147180	ZNF711	-2.16869665	0.013224068
ENSG00000277394	NA	-1.089742096	0.013258969
ENSG00000213190	MLLT11	-0.858086348	0.013296148
ENSG00000204217	BMPR2	0.733837102	0.013296148
ENSG00000164054	SHISA5	0.70956252	0.013296148
ENSG00000273437		-1.66056545	0.013296148
ENSG00000007520	TSR3	-0.588449868	0.013296148
ENSG00000081985	IL12RB2	-3.073461965	0.013317586
ENSG00000198677	TTC37	0.552528011	0.013317586
ENSG00000237190	CDKN2AIPNL	-0.520466358	0.01336401
ENSG00000166912	MTMR10	0.514013269	0.013380965
ENSG00000162736	NCSTN	0.480432559	0.013407314
ENSG00000114779	ABHD14B	-0.494157949	0.013409732
ENSG00000271553		-1.510245242	0.013409732
ENSG00000240682	ISY1	-0.360776119	0.013435811
ENSG00000258199		-0.622151467	0.013435811
ENSG00000211669	IGLV3-10	-3.782053925	0.013435811
ENSG00000259658		-1.677475795	0.013483798
ENSG00000163382	NAXE	-0.575187072	0.013484458
ENSG00000184897	H1FX	-0.727008081	0.013506469
ENSG00000084207	GSTP1	-0.731848515	0.013506469
ENSG00000273149		-2.045756456	0.013506469
ENSG00000187840	EIF4EBP1	-0.780402165	0.013530932
ENSG00000113615	SEC24A	0.778597553	0.01354129
ENSG00000127884	ECHS1	-0.653674984	0.013647942
ENSG00000148019	CEP78	-0.899135607	0.013734942
ENSG00000265206	MIR142	-1.187633688	0.013734942
ENSG00000078487	ZCWPW1	-0.789108602	0.013792294
ENSG00000163565	IFI16	1.190751141	0.01382954
ENSG00000122543	OCM	-2.267874837	0.01382954
ENSG00000118181	RPS25	-0.477755511	0.013890027
ENSG00000167969	ECI1	-0.645584092	0.013949731
ENSG00000270605		-1.346253765	0.013978934
ENSG00000144034	TPRKB	-0.537903195	0.013997871
ENSG00000170667	RASA4B	-1.240791366	0.013997871
ENSG00000198964	SGMS1	0.705460877	0.014002392
ENSG00000103264	FBXO31	-0.560267772	0.014002392
ENSG00000198455	ZXDB	-0.672364179	0.014052034
ENSG00000241343	RPL36A	-0.670842947	0.014052034
ENSG00000177990	DPY19L2	-3.250715843	0.014052034
ENSG00000180228	PRKRA	-0.459795781	0.01407087
ENSG00000141560	FN3KRP	-0.504027964	0.01407087
ENSG00000214389	RPS3AP26	-0.595167186	0.014071635
ENSG00000124713	GNMT	-0.859496577	0.014142833
ENSG00000251867		-1.065093226	0.014142833

ENSG00000160307	S100B	-2.579918498	0.014260542
ENSG00000211666	IGLV2-14	-3.150814001	0.014275095
ENSG00000078043	PIAS2	-0.509689419	0.014284663
ENSG00000165874	FAM35BP	-0.957846385	0.014362961
ENSG00000128602	SMO	-2.47765222	0.014538935
ENSG00000140092	FBLN5	-2.600765358	0.014558065
ENSG00000273192		-1.08003371	0.014558065
ENSG00000162032	SPSB3	-0.464910746	0.014732074
ENSG00000125356	NDUFA1	-0.455288831	0.01475699
ENSG00000257151	PWAR6	-1.432849402	0.014785091
ENSG00000204564	C6orf136	-0.538589027	0.014811965
ENSG00000228224	NACAP1	-0.739638833	0.014834346
ENSG00000225151	GOLGA2P7	-1.565221751	0.014834346
ENSG00000167555	ZNF528	-0.992826985	0.014834346
ENSG00000064651	SLC12A2	-0.793090469	0.014917375
ENSG00000182196	ARL6IP4	-0.952481405	0.015075074
ENSG00000134709	HOOK1	-2.319506226	0.015107209
ENSG00000102901	CENPT	-0.468266771	0.015117836
ENSG00000139725	RHOF	-0.580107087	0.015132893
ENSG00000211649	IGLV7-46	-3.531368153	0.015286754
ENSG00000137449	CPEB2	0.888801964	0.01530045
ENSG00000124507	PACSIN1	-3.250094497	0.01530045
ENSG00000176058	TPRN	-0.729942091	0.01530045
ENSG00000166166	TRMT61A	-0.526308335	0.01530045
ENSG00000213977	TAX1BP3	-0.780254217	0.01530045
ENSG00000168610	STAT3	0.627057265	0.01530045
ENSG00000196684	HSH2D	1.097571501	0.01530045
ENSG00000186806	VSIG10L	1.094711625	0.01530045
ENSG00000245904		-0.995704509	0.015323231
ENSG00000196998	WDR45	-0.375541741	0.015340111
ENSG00000095139	ARCN1	0.669919323	0.01539867
ENSG00000152154	TMEM178A	-2.620103925	0.015501269
ENSG00000141576	RNF157	-1.207778393	0.015555059
ENSG00000148290	SURF1	-0.443691169	0.015577545
ENSG00000278740		-2.274704525	0.015577545
ENSG00000143185	XCL2	-3.599545714	0.015751381
ENSG00000118640	VAMP8	-0.606932233	0.015751381
ENSG00000144199	FAHD2B	-1.343568358	0.015790049
ENSG00000234771	SLC25A25-AS1	-0.68349797	0.015790049
ENSG00000144659	SLC25A38	-0.550482755	0.015833578
ENSG00000211970	IGHV4-61	-3.507718434	0.015879956
ENSG00000145431	PDGFC	-1.318507701	0.015933389
ENSG00000006062	MAP3K14	-0.474045138	0.015933389
ENSG00000161010	MRNIP	-0.970403607	0.015979724
ENSG00000171219	CDC42BPG	2.720608889	0.015989686
ENSG00000274227		-1.37366901	0.015989686
ENSG00000243477	NAT6	-0.585740782	0.016012226
ENSG00000138443	ABI2	-0.745451512	0.016092459

ENSG00000163872	YEATS2	0.48064512	0.016092459
ENSG00000131876	SNRPA1	-0.593698081	0.016092459
ENSG00000160917	CPSF4	-0.38248416	0.016274831
ENSG00000271895		-0.94121064	0.016288805
ENSG00000131469	RPL27	-0.45630887	0.016304221
ENSG00000263528	IKBKE	0.810852139	0.016342747
ENSG00000117543	DPH5	-0.6276373	0.016344359
ENSG00000143344	RGL1	2.221136158	0.016534809
ENSG00000134070	IRAK2	3.119286725	0.016534809
ENSG00000153560	UBP1	0.526902565	0.016650644
ENSG00000238105	GOLGA2P5	-1.202551167	0.016650644
ENSG00000265100		-1.356446539	0.016650644
ENSG00000099800	TIMM13	-0.749442818	0.016650644
ENSG00000173706	HEG1	0.970456093	0.016654306
ENSG00000167664	TMIGD2	-1.908609514	0.016661754
ENSG00000106992	AK1	-1.287630367	0.016663329
ENSG00000232022	FAAHP1	-2.037324409	0.016671838
ENSG00000184083	FAM120C	-0.673984701	0.016671838
ENSG00000130119	GNL3L	0.718507143	0.016671838
ENSG00000243678	NME2	-0.71329529	0.016671838
ENSG00000218891	ZNF579	-1.011762189	0.016674018
ENSG00000007312	CD79B	-1.218079983	0.016712204
ENSG00000211895	IGHA1	-3.770559918	0.016766434
ENSG00000179715	PCED1B	-1.164952896	0.016774318
ENSG00000152969	JAKMIP1	-3.326069988	0.016796722
ENSG00000133398	MED10	-0.446388581	0.016838577
ENSG00000126012	KDM5C	0.441850841	0.016902087
ENSG00000137502	RAB30	-1.489518732	0.016906639
ENSG00000181036	FCRL6	-2.954016753	0.016913816
ENSG00000273066		-1.115420026	0.016967723
ENSG00000185641		-0.722022497	0.017013121
ENSG00000260136		-1.1586933	0.017013121
ENSG00000111786	SRSF9	-0.290775124	0.01716427
ENSG00000136718	IMP4	-0.447974818	0.017191643
ENSG00000107672	NSMCE4A	-0.530124376	0.017191643
ENSG00000164904	ALDH7A1	-1.637303333	0.017204497
ENSG00000174775	HRAS	-0.608484071	0.017204497
ENSG00000213741	RPS29	-0.609704231	0.017272022
ENSG00000256060	TRAPPC2B	-0.890904051	0.017272022
ENSG00000164406	LEAP2	-1.156953371	0.017356553
ENSG00000179085	DPM3	-0.657218469	0.01736454
ENSG00000131368	MRPS25	-0.572713299	0.017405633
ENSG00000242660		-1.084394867	0.017591376
ENSG00000108773	KAT2A	-0.691814886	0.017591376
ENSG00000103253	HAGHL	-1.105040543	0.017644567
ENSG00000135185	TMEM243	-0.534280262	0.017692163
ENSG00000234444	ZNF736	-0.568903991	0.017776319
ENSG00000275764		-0.67758894	0.017776546

ENSG00000175262	C1orf127	-1.068850696	0.017884059
ENSG00000138688	KIAA1109	0.578015415	0.017884059
ENSG00000134545	KLRC1	-3.060498156	0.017884059
ENSG00000160948	VPS28	-0.482331785	0.017912834
ENSG00000198680	TUSC1	-1.63488801	0.017912834
ENSG00000244380		-1.210808817	0.017913014
ENSG00000279088		-0.7841835	0.017913014
ENSG00000214140	PRCD	-3.55381793	0.017913014
ENSG00000160113	NR2F6	-1.212454217	0.017945887
ENSG00000165775	FUNDC2	-0.418516625	0.018028659
ENSG00000086619	ERO1B	-0.585984108	0.018068441
ENSG00000105443	CYTH2	-0.466271863	0.018074086
ENSG00000090971	NAT14	-0.839564025	0.01810492
ENSG00000027075	PRKCH	-0.683584209	0.018111054
ENSG00000266998		-1.625654577	0.018288125
ENSG00000224420	ADM5	-1.025623561	0.018441558
ENSG00000275055		-1.160901851	0.018536175
ENSG00000173818	ENDOV	-0.614846388	0.018655075
ENSG00000215421	ZNF407	0.484096377	0.018655075
ENSG00000057757	PITHD1	-0.534868587	0.018773235
ENSG00000203791	METTL10	-0.543901435	0.018789729
ENSG00000247092	SNHG10	-1.005768906	0.018809841
ENSG00000116096	SPR	-0.969625501	0.018816244
ENSG00000066135	KDM4A	0.499997993	0.018864213
ENSG00000149970	CNKSR2	-2.458232537	0.018864213
ENSG00000279576		-2.385761474	0.01887525
ENSG00000171428	NAT1	0.969547683	0.018880774
ENSG00000278611		-1.463296899	0.018880774
ENSG00000012061	ERCC1	-0.522775716	0.018880774
ENSG00000171680	PLEKHG5	-2.557725989	0.019029394
ENSG00000074071	MRPS34	-0.739213557	0.019083891
ENSG00000153827	TRIP12	0.389922822	0.019096363
ENSG00000101132	PFDN4	-0.597024801	0.019096363
ENSG00000271335		-1.052149329	0.019336499
ENSG00000253948		-1.081048827	0.019360299
ENSG00000101444	AHCY	-0.670269235	0.019465108
ENSG00000173653	RCE1	-0.534707693	0.019468974
ENSG00000138385	SSB	0.58752727	0.019510674
ENSG00000148334	PTGES2	-0.439576683	0.019510674
ENSG00000268061	NAPA-AS1	-1.312402907	0.019510674
ENSG00000241370	RPP21	-0.727817679	0.019566259
ENSG00000100429	HDAC10	-0.663306707	0.019566259
ENSG00000102103	PQBP1	-0.428839085	0.019568386
ENSG00000157500	APPL1	0.524342498	0.019609845
ENSG00000106603	COA1	0.60388005	0.019609845
ENSG00000181074	OR52N4	4.200486331	0.01962332
ENSG00000008324	SS18L2	-0.537276551	0.019633656
ENSG00000260081		-1.673892894	0.019668701

ENSG00000104408	EIF3E	-0.441967395	0.019908287
ENSG00000230629	RPS23P8	-0.677825922	0.019953543
ENSG00000004059	ARF5	-0.355552505	0.020012146
ENSG00000183826	BTBD9	0.69115264	0.020024064
ENSG00000105552	BCAT2	-0.681559824	0.020066095
ENSG00000248015		-1.806609329	0.020078169
ENSG00000124602	UNC5CL	-0.856182346	0.020130699
ENSG00000153339	TRAPPC8	0.512528972	0.020179015
ENSG00000011478	QPCTL	-0.732466564	0.020200488
ENSG00000258352		-2.125330043	0.020205048
ENSG00000132635	PCED1A	-0.528318267	0.02023885
ENSG00000164405	UQCRQ	-0.49806451	0.020393923
ENSG00000158062	UBXN11	-0.447512591	0.020627017
ENSG00000127445	PIN1	-0.41622989	0.020669226
ENSG00000108700	CCL8	4.226346124	0.020937793
ENSG00000151131	C12orf45	-0.578411949	0.020964103
ENSG00000180902	D2HGDH	-0.831927348	0.021049121
ENSG00000196756	SNHG17	-0.679295818	0.02112766
ENSG00000165949	IFI27	4.770825258	0.021135298
ENSG00000095066	HOOK2	-0.664706979	0.021227486
ENSG00000130770	ATPIF1	-0.4965366	0.021237623
ENSG00000239855	IGKV1-6	-3.375942046	0.021303799
ENSG00000100393	EP300	0.549368911	0.021388682
ENSG00000169902	TPST1	2.406367558	0.021675445
ENSG00000089289	IGBP1	-0.365223557	0.021732681
ENSG00000179526	SHARPIN	-0.464015576	0.02174475
ENSG00000242259	C22orf39	-0.477965785	0.021754934
ENSG00000142089	IFITM3	1.848748676	0.021904001
ENSG00000207110	RNU1-106P	-2.150701395	0.02193966
ENSG00000239951	IGKV3-20	-2.719894061	0.021964437
ENSG00000248124	RRN3P1	-0.723521661	0.021980056
ENSG00000128191	DGCR8	-0.445673273	0.021983008
ENSG00000165914	TTC7B	0.899027111	0.022018751
ENSG00000038219	BOD1L1	0.59881334	0.022030571
ENSG00000060140	STYK1	-3.490852114	0.022077729
ENSG00000170906	NDUFA3	-0.466914138	0.022114575
ENSG00000176101	SSNA1	-0.527596025	0.022180152
ENSG00000054690	PLEKHH1	1.179106108	0.022366335
ENSG00000273759		-1.389663899	0.022366335
ENSG00000008128	CDK11A	-0.661219865	0.022470092
ENSG00000185187	SIGIRR	-0.597020231	0.022470092
ENSG00000105677	TMEM147	-0.514631162	0.022617544
ENSG00000261416		-0.743872654	0.022847321
ENSG00000226419	SLC16A1-AS1	-0.59680562	0.022882342
ENSG00000160131	VMA21	-0.424717368	0.022882342
ENSG00000050426	LETMD1	-0.514785631	0.022883852
ENSG00000183763	TRAIP	-1.366846428	0.022910014
ENSG00000164048	ZNF589	-0.710868126	0.022916539

ENSG00000083838	ZNF446	-0.561847927	0.022922934
ENSG00000266340		-1.228944183	0.022948669
ENSG00000268713		-0.724836241	0.023065269
ENSG00000213066	FGFR1OP	-0.727638304	0.023151449
ENSG00000280325		-1.149338207	0.023299174
ENSG00000255760		-1.565508151	0.023299174
ENSG00000103363	TCEB2	-0.531490165	0.023394081
ENSG00000272221		-1.56219835	0.023413879
ENSG00000174109	C16orf91	-0.630689789	0.023529239
ENSG00000265241	RBM8A	-0.328568895	0.023582225
ENSG00000234389		-2.778933482	0.023634995
ENSG00000277511		1.541838154	0.023634995
ENSG00000202198		-1.454250762	0.023697983
ENSG00000123338	NCKAP1L	0.45373088	0.023697983
ENSG00000135636	DYSF	1.700525736	0.023771406
ENSG00000271964		-1.568971033	0.023771406
ENSG00000243449	C4orf48	-1.020338705	0.023771406
ENSG00000147155	EBP	-0.61772847	0.023771406
ENSG00000159399	HK2	0.626258385	0.023851496
ENSG00000105583	WDR83OS	-0.414973876	0.023851496
ENSG00000231584	FAHD2CP	-1.220708606	0.023855496
ENSG00000248019	FAM13A-AS1	-0.581646659	0.023855496
ENSG00000109390	NDUFC1	-0.53457162	0.023855496
ENSG00000145592	RPL37	-0.453959462	0.024162562
ENSG00000175602	CCDC85B	-0.666596767	0.024201645
ENSG00000183617	MRPL54	-0.601750994	0.024201645
ENSG00000105379	ETFB	-0.557088643	0.024201645
ENSG00000100226	GTPBP1	0.860357193	0.024258111
ENSG00000233483		-3.000399729	0.024368275
ENSG00000272533	SNORA28	1.931306279	0.024373057
ENSG00000198556	ZNF789	-0.647327969	0.024401395
ENSG00000087460	GNAS	-0.282287506	0.024401395
ENSG00000129515	SNX6	0.508605882	0.02440616
ENSG00000127415	IDUA	-0.545859643	0.024551469
ENSG00000120733	KDM3B	0.438746364	0.024564399
ENSG00000213442	RPL18AP3	-0.597550007	0.024569108
ENSG00000217094	PPIAP31	-0.489413968	0.024617783
ENSG00000168101	NUDT16L1	-0.508281781	0.024765368
ENSG00000260793		-0.691976833	0.024765368
ENSG00000266402	SNHG25	-1.096040587	0.024765368
ENSG00000100379	KCTD17	-0.717785173	0.024765368
ENSG00000142856	ITGB3BP	-0.879563266	0.024801954
ENSG00000103037	SETD6	-0.605072025	0.024838144
ENSG00000184702	Sep-05	4.670108675	0.02489078
ENSG00000188690	UROS	-0.439894774	0.024999856
ENSG00000130165	ELOF1	-0.533120665	0.025007238
ENSG00000211799	TRAV19	-3.018557236	0.02501746
ENSG00000177548	RABEP2	-0.547405608	0.025031193

ENSG00000115750	TAF1B	0.661856621	0.025236953
ENSG00000177272	KCNA3	1.044102485	0.025240999
ENSG00000267074		-2.394314047	0.025240999
ENSG00000249855	EEF1A1P19	-0.730287065	0.025253359
ENSG00000134313	KIDINS220	0.538479177	0.025324746
ENSG00000067829	IDH3G	-0.301618797	0.025430056
ENSG00000103254	FAM173A	-0.927221358	0.025566554
ENSG00000053501	USE1	-0.4832401	0.025566554
ENSG00000182173	TSEN54	-0.706992645	0.025744107
ENSG00000115042	FAHD2A	-0.563994819	0.025773698
ENSG00000243466	IGKV1-5	-2.781513217	0.025848782
ENSG00000126768	TIMM17B	-0.409820478	0.025848782
ENSG00000204344	STK19	-0.519017861	0.025894128
ENSG00000107537	PHYH	-0.634643832	0.025894128
ENSG00000124243	BCAS4	-1.002085367	0.025916237
ENSG00000179954	SSC5D	-1.765948635	0.025981434
ENSG00000198546	ZNF511	-0.428775611	0.026051472
ENSG00000140030	GPR65	0.7729409	0.026108199
ENSG00000117410	ATP6V0B	-0.484516882	0.02615247
ENSG00000117228	GBP1	1.651625141	0.02615247
ENSG00000268403		-0.747181123	0.02615247
ENSG00000152766	ANKRD22	1.646745787	0.02615247
ENSG00000131979	GCH1	0.716517277	0.02615247
ENSG00000006282	SPATA20	-0.829620121	0.02615247
ENSG00000250506	CDK3	-0.952524997	0.02615247
ENSG00000229299		-1.395536895	0.02615247
ENSG00000100483	VCPKMT	-0.648172718	0.026160239
ENSG00000164114	MAP9	-2.175604169	0.0261846
ENSG00000134516	DOCK2	0.441522733	0.0261846
ENSG00000156508	EEF1A1	-0.475740995	0.0261846
ENSG00000162654	GBP4	1.243618971	0.026437529
ENSG00000005810	MYCBP2	0.504227306	0.02654205
ENSG00000270170	NCBP2-AS2	-0.548623003	0.02655931
ENSG00000085465	OVGP1	-1.160938159	0.026573702
ENSG00000129484	PARP2	-0.691438455	0.026573702
ENSG00000103485	QPRT	-0.898589475	0.026573702
ENSG00000104964	AES	-0.427359696	0.026574843
ENSG00000162415	ZSWIM5	-2.559771739	0.026613747
ENSG00000243244	STON1	-0.906717588	0.026613747
ENSG00000126262	FFAR2	1.838254989	0.026613747
ENSG00000100596	SPTLC2	0.823111971	0.026648202
ENSG00000276259		-1.140236704	0.026648202
ENSG00000140853	NLRC5	0.588146336	0.026665938
ENSG00000069493	CLEC2D	-0.772199969	0.026715137
ENSG00000072195	SPEG	-1.707509795	0.026798481
ENSG00000173762	CD7	-0.887402753	0.026843681
ENSG00000169592	INO80E	-0.680623857	0.026884029
ENSG00000279377		-3.228588384	0.026946663

ENSG00000120699	EXOSC8	-0.495694196	0.027067486
ENSG00000135930	EIF4E2	-0.334957596	0.027132009
ENSG00000218426		-0.566652354	0.027132009
ENSG00000135144	DTX1	-2.360087427	0.027132009
ENSG00000174365	SNHG11	-0.601499649	0.027132009
ENSG00000204136	GGTA1P	-1.548244445	0.027168336
ENSG00000173141	MRPL57	-0.545204375	0.027218216
ENSG00000171476	HOPX	-1.814377133	0.02725711
ENSG00000153815	CMIP	0.446230822	0.02725711
ENSG00000099840	IZUMO4	-0.761666539	0.02725711
ENSG00000174718	KIAA1551	0.801038844	0.027293241
ENSG00000125611	CHCHD5	-0.596177327	0.027449928
ENSG00000274943		-1.251394839	0.027449928
ENSG00000151773	CCDC122	-0.999013396	0.027559353
ENSG00000226360	RPL10AP6	-0.78768006	0.027649747
ENSG00000115307	AUP1	-0.266297755	0.027976139
ENSG00000129559	NEDD8	-0.442027223	0.028238449
ENSG00000168890	TMEM150A	-0.582318444	0.028273716
ENSG00000244733		-0.93714824	0.028307948
ENSG00000011028	MRC2	-2.970589196	0.028437908
ENSG00000112294	ALDH5A1	-1.585114286	0.028502674
ENSG00000214535	RPS15AP1	-0.78077908	0.028502674
ENSG00000159374	M1AP	-0.956396706	0.028673255
ENSG00000102554	KLF5	2.351671248	0.02875739
ENSG00000113161	HMGCR	0.529940949	0.028764719
ENSG00000182154	MRPL41	-0.731952208	0.028781917
ENSG00000182010	RTKN2	-1.804522747	0.028930998
ENSG00000167107	ACSF2	-0.76704756	0.028960748
ENSG00000218227		-0.546770316	0.029115269
ENSG00000218208	RPS27AP11	-1.482902823	0.02955142
ENSG00000256039		-2.381107855	0.02955142
ENSG00000260778	MIR940	-1.170978183	0.02955142
ENSG00000109927	TECTA	1.566860643	0.029701385
ENSG00000177732	SOX12	-0.922954635	0.029830304
ENSG00000110777	POU2AF1	-1.398913506	0.029950324
ENSG00000035720	STAP1	2.025980146	0.029990802
ENSG00000213366	GSTM2	-0.893209751	0.030044717
ENSG00000138031	ADCY3	1.254956405	0.030417542
ENSG00000204852	TCTN1	-0.761580057	0.030417542
ENSG00000037749	MFAP3	0.77347169	0.030526444
ENSG00000100100	PIK3IP1	-0.88295031	0.030526444
ENSG00000275832	ARHGAP23	1.352456469	0.030574038
ENSG00000224032	EPB41L4A-AS1	-0.690196819	0.030603847
ENSG00000139626	ITGB7	0.88692263	0.030603847
ENSG00000233276	GPX1	-0.686458178	0.030638697
ENSG00000159714	ZDHHC1	-1.665590938	0.030669032
ENSG00000183401	CCDC159	-0.541278862	0.03069316
ENSG00000123219	CENPK	-1.610867724	0.030743588

ENSG00000100345	MYH9	0.597007844	0.030743588
ENSG00000084623	EIF3I	-0.416293458	0.030748141
ENSG00000171453	POLR1C	-0.646887259	0.030924664
ENSG00000100417	PMM1	-0.47264246	0.030924898
ENSG00000112378	PERP	-2.635919782	0.03095675
ENSG00000007080	CCDC124	-0.466497542	0.03095675
ENSG00000276819	TRBV15	-3.145480665	0.030959377
ENSG00000270550	IGHV3-30	-2.957175826	0.030980999
ENSG00000251259		-0.675131068	0.031101143
ENSG00000272936		-1.075203312	0.031194165
ENSG00000214941	ZSWIM7	-0.594116675	0.031194165
ENSG00000274104		-0.910782647	0.031194165
ENSG00000189403	HMGB1	-0.453300593	0.031260233
ENSG00000255987	TOMM20P2	-3.003159261	0.031260233
ENSG00000169885	CALML6	-2.07770488	0.031301221
ENSG00000136149	RPL13AP25	-0.875344528	0.031360857
ENSG00000137501	SYTL2	-1.024627903	0.031654105
ENSG00000182957	SPATA13	0.620572627	0.031654105
ENSG00000211706	TRBV6-1	-3.141847884	0.031793589
ENSG00000100403	ZC3H7B	0.41059234	0.031793589
ENSG00000154511	FAM69A	0.942666024	0.03179971
ENSG00000196131	VN1R2	-1.355878047	0.03179971
ENSG00000211767	TRBJ2-3	-3.139457305	0.03189769
ENSG00000185972	CCIN	-1.214521614	0.031949625
ENSG00000163041	H3F3A	-0.341386509	0.03204973
ENSG00000175283	DOLK	0.864708389	0.032067566
ENSG00000232613		-3.126739335	0.032087895
ENSG00000105875	WDR91	-0.500434143	0.032105275
ENSG00000255857	PXN-AS1	-1.156244751	0.032110066
ENSG00000005436	GCFC2	-0.547052323	0.032224574
ENSG00000262484	CCER2	-1.238904014	0.032293073
ENSG00000241741	RPL7AP30	-0.54297574	0.032676997
ENSG00000204977	TRIM13	-0.703229421	0.032676997
ENSG00000073605	GSDMB	-0.916128152	0.032676997
ENSG00000205089	CCNI2	-3.132477657	0.032699469
ENSG00000213654	GPSM3	-0.350420323	0.032699469
ENSG00000228623	ZNF883	-1.666408122	0.032699469
ENSG00000176994	SMCR8	0.614262407	0.032762852
ENSG00000013306	SLC25A39	-0.406357824	0.032994327
ENSG00000177954	RPS27	-0.508490461	0.03302649
ENSG00000282133	TRBJ1-3	-3.122122768	0.03302649
ENSG00000013288	MAN2B2	0.458291424	0.033037605
ENSG00000215252	GOLGA8B	-0.960610528	0.033108971
ENSG00000272106		-0.544321447	0.033155345
ENSG00000174669	SLC29A2	-0.778754721	0.033155345
ENSG00000223496	EXOSC6	-0.60991536	0.033155345
ENSG00000176171	BNIP3	-0.714475273	0.033256389
ENSG00000274712		-0.79595103	0.033330956

ENSG00000255569	TRAV1-1	-3.157950142	0.033368408
ENSG00000183049	CAMK1D	-0.527030404	0.033368662
ENSG00000267787		0.735793459	0.033478936
ENSG00000232586	KIAA1614-AS1	1.348247254	0.033492287
ENSG00000213533	TMEM110	0.744363971	0.033620468
ENSG00000087191	PSMC5	-0.391997405	0.033733249
ENSG00000196642	RABL6	-0.356265258	0.033745323
ENSG00000177700	POLR2L	-0.603986621	0.033789369
ENSG00000169740	ZNF32	-0.471885165	0.033860637
ENSG00000133466	C1QTNF6	-0.916499763	0.033862151
ENSG00000154153	FAM134B	1.041060832	0.033871397
ENSG00000122034	GTF3A	-0.386096365	0.033902402
ENSG00000114923	SLC4A3	-2.051646499	0.033909632
ENSG00000185495		-0.84349115	0.033927887
ENSG00000124374	PAIP2B	-1.228347126	0.033969352
ENSG00000238578	SNORD4A	-0.922500975	0.034027811
ENSG00000159884	CCDC107	-0.574303765	0.034072779
ENSG00000128253	RFPL2	-2.198988304	0.034094497
ENSG00000182827	ACBD3	0.610083456	0.034193911
ENSG00000095787	WAC	0.350905793	0.034205993
ENSG00000267128		-3.170802408	0.034232141
ENSG00000280033		-0.950904987	0.034287409
ENSG00000165813	CCDC186	0.623636172	0.034411608
ENSG00000211765	TRBJ2-2	-3.127492404	0.03460093
ENSG00000001497	LAS1L	-0.335848855	0.03460093
ENSG00000107014	RLN2	-3.124462695	0.034609656
ENSG00000196557	CACNA1H	-3.13753811	0.034682679
ENSG00000135423	GLS2	-3.134050793	0.034688976
ENSG00000182195	LDOC1	-2.564106141	0.034741283
ENSG00000234936		-1.215175687	0.034762409
ENSG00000224650	IGHV3-74	-3.013773143	0.034804186
ENSG00000132256	TRIM5	1.293361757	0.034851519
ENSG00000119862	LGALSL	-1.980550104	0.034865078
ENSG00000171049	FPR2	1.784377785	0.03487645
ENSG00000103145	HCFC1R1	-0.580117969	0.034891638
ENSG00000120899	PTK2B	0.419871291	0.034929569
ENSG00000274349	ZNF658	1.776641156	0.034967937
ENSG00000164151	ICE1	0.430903588	0.034988015
ENSG00000127054	CPSF3L	-0.350677884	0.035066143
ENSG00000166669	ATF7IP2	-0.586445406	0.035067442
ENSG00000125901	MRPS26	-0.637862287	0.035067442
ENSG00000084072	PPIE	-0.906582112	0.035143519
ENSG00000213889	PPM1N	-1.131579967	0.035212093
ENSG00000131504	DIAPH1	0.451591587	0.035247204
ENSG00000166317	SYNPO2L	-1.023135003	0.035247204
ENSG00000211941	IGHV3-11	-3.140047156	0.035247204
ENSG00000175886	RPL7AP66	-0.900023271	0.035317555
ENSG00000204311	DFNB59	-1.283434607	0.035362496

ENSG00000273428		-2.552165904	0.035362496
ENSG00000183762	KREMEN1	1.345888704	0.035488619
ENSG00000187608	ISG15	1.574771231	0.035526645
ENSG00000106537	TSPAN13	-1.752663132	0.035546336
ENSG00000104133	SPG11	0.540094832	0.035546336
ENSG00000165996	HACD1	1.579230811	0.035559015
ENSG00000033178	UBA6	0.415569765	0.035559978
ENSG00000250318		-0.949237485	0.03568527
ENSG00000173915	USMG5	-0.480903549	0.035790317
ENSG00000198840	MT-ND3	-0.637821133	0.035790317
ENSG00000165113	GKAP1	-0.844105722	0.035813908
ENSG00000162191	UBXN1	-0.397399403	0.035830161
ENSG00000157734	SNX22	-1.134628183	0.035852094
ENSG00000105135	ILVBL	-0.614590821	0.035878418
ENSG00000206573	THUMPD3-AS1	-0.50434949	0.035878721
ENSG00000225783	MIAT	-1.514720714	0.035903707
ENSG00000010818	HIVEP2	0.707613798	0.035934976
ENSG00000213398	LCAT	-0.610943667	0.035934976
ENSG00000210154	MT-TD	-1.252216756	0.03613769
ENSG00000004487	KDM1A	-0.325836012	0.036151703
ENSG00000160058	BSDC1	0.386309303	0.036151703
ENSG00000162714	ZNF496	0.797889866	0.036151703
ENSG00000166394	CYB5R2	2.156661439	0.036151703
ENSG00000256525	POLG2	-0.595708069	0.036375289
ENSG00000228863		-0.916112411	0.036647666
ENSG00000272282		-3.14211798	0.036647666
ENSG00000066084	DIP2B	0.623097424	0.036647666
ENSG00000240403	KIR3DL2	-3.01134738	0.036647666
ENSG00000242252	BGLAP	-1.303679641	0.036820939
ENSG00000151923	TIAL1	-0.350926698	0.036966771
ENSG00000259865		-0.615671285	0.037040779
ENSG00000225663	MCRIP1	-0.506342952	0.037040779
ENSG00000165637	VDAC2	-0.405267524	0.037064592
ENSG00000175634	RPS6KB2	-0.359565405	0.037097045
ENSG00000129244	ATP1B2	2.452024696	0.037097045
ENSG00000173281	PPP1R3B	1.00650917	0.037148637
ENSG00000138942	RNF185	0.438314908	0.03717129
ENSG00000110047	EHD1	1.190250834	0.037343987
ENSG00000125844	RRBP1	0.760931306	0.037419802
ENSG00000164008	C1orf50	-0.582178789	0.037429087
ENSG00000211943	IGHV3-15	-2.766614175	0.037449513
ENSG00000204348	DXO	-0.608004481	0.037464351
ENSG00000226525	RPS7P10	-0.665779571	0.037464351
ENSG00000104979	C19orf53	-0.392887873	0.037464351
ENSG00000126215	XRCC3	-0.778518787	0.037486446
ENSG00000070718	AP3M2	-0.755236337	0.037567436
ENSG00000050165	DKK3	-3.07002126	0.037722116
ENSG00000143353	LYPLAL1	-0.67515051	0.03773692

ENSG00000105851	PIK3CG	0.650600721	0.037782087
ENSG00000161618	ALDH16A1	-0.538054593	0.037782087
ENSG00000134193	REG4	-3.132365841	0.03779011
ENSG00000112851	ERBIN	0.407055497	0.03779011
ENSG00000231621		-3.108294666	0.037792476
ENSG00000103495	MAZ	-0.383223456	0.037792476
ENSG00000219665		-0.577610071	0.037792476
ENSG00000196456	ZNF775	-0.841372749	0.037894597
ENSG00000130299	GTPBP3	-0.560963736	0.038045497
ENSG00000164327	RICTOR	0.577728085	0.038089008
ENSG00000242686		-1.980835601	0.038230786
ENSG00000211934	IGHV1-2	-3.132088986	0.038230786
ENSG00000169689	CENPX	-0.677395594	0.038230786
ENSG00000139610	CELA1	-1.856303945	0.038291031
ENSG00000196459	TRAPPC2	-0.650900414	0.038295141
ENSG00000272010		-1.835313899	0.038367689
ENSG00000168672	FAM84B	-1.180598628	0.038423588
ENSG00000067900	ROCK1	0.496609082	0.038423588
ENSG00000231793	DOC2GP	-2.180461708	0.038429123
ENSG00000085831	TTC39A	3.959541993	0.038460999
ENSG00000106400	ZNHIT1	-0.454793794	0.038460999
ENSG00000163354	DCST2	-0.917820082	0.038577925
ENSG00000131495	NDUFA2	-0.506561418	0.038577925
ENSG00000256453	DND1	-0.968704311	0.038577925
ENSG00000105649	RAB3A	-1.295725834	0.038577925
ENSG00000167685	ZNF444	-0.539939093	0.038577925
ENSG00000211651	IGLV1-44	-2.342070004	0.038577925
ENSG00000171861	MRM3	-0.543922883	0.03879922
ENSG00000204314	PRRT1	-2.135957472	0.038925099
ENSG00000211710	TRBV4-1	-2.583422807	0.038925858
ENSG00000197381	ADARB1	0.752879288	0.038961365
ENSG00000235499		1.34965032	0.039043741
ENSG00000104886	PLEKHJ1	-0.591586089	0.039043741
ENSG00000125868	DSTN	-0.62240147	0.039070752
ENSG00000005513	SOX8	-3.13322069	0.039194706
ENSG00000171443	ZNF524	-0.57660138	0.039247214
ENSG00000180316	PNPLA1	1.508094716	0.039253828
ENSG00000167118	URM1	-0.352221087	0.039253828
ENSG00000127540	UQCR11	-0.456021005	0.039253828
ENSG00000205726	ITSN1	0.814969215	0.039253828
ENSG00000154134	ROBO3	-1.100776893	0.039299674
ENSG00000141696	P3H4	-1.281001714	0.039390117
ENSG00000136147	PHF11	0.604548822	0.039440758
ENSG00000213145	CRIP1	-0.823440506	0.039440758
ENSG00000171596	NMUR1	-2.218089977	0.039540801
ENSG00000079462	PAFAH1B3	-0.62658285	0.039540801
ENSG00000271869		-0.849887104	0.039619223
ENSG00000181038	METTL23	-0.400895686	0.039619223

ENSG00000234127	TRIM26	0.525215859	0.039621182
ENSG00000068305	MEF2A	0.430996933	0.039688689
ENSG00000086289	EPDR1	1.976097468	0.039701695
ENSG00000109832	DDX25	1.086776017	0.039702803
ENSG00000133216	EPHB2	2.16308285	0.039724731
ENSG00000120217	CD274	1.243001197	0.039751034
ENSG00000256427		-3.133555552	0.039751034
ENSG00000260404		0.813739748	0.039797937
ENSG00000111364	DDX55	-0.583195421	0.039797937
ENSG00000204859	ZBTB48	-0.433862951	0.039801849
ENSG00000181097		-1.263586778	0.039801849
ENSG00000253755	IGHGP	-3.00592943	0.039860683
ENSG00000183066	WBP2NL	-1.017491409	0.039860683
ENSG00000173511	VEGFB	-1.362026273	0.039932513
ENSG00000112695	COX7A2	-0.403153351	0.040112174
ENSG00000124802	EEF1E1	-0.748242645	0.040136221
ENSG00000111678	C12orf57	-0.665255545	0.0402159
ENSG00000089248	ERP29	-0.340637481	0.040305685
ENSG00000197774	EME2	-0.456831248	0.040496763
ENSG00000116688	MFN2	0.402679218	0.040519935
ENSG00000144061	NPHP1	-1.476097749	0.040648248
ENSG00000169964	TMEM42	-0.724959272	0.040709488
ENSG00000245937	LINC01184	-0.655871615	0.040709488
ENSG00000137411	VARS2	-0.683622059	0.040709488
ENSG00000120647	CCDC77	-0.607987808	0.040709488
ENSG00000159618	ADGRG5	-1.371534879	0.040709488
ENSG00000204435	CSNK2B	-0.62467718	0.041049268
ENSG00000125900	SIRPD	1.071804264	0.041148223
ENSG00000167601	AXL	2.128461992	0.041327364
ENSG00000158470	B4GALT5	0.908361385	0.041515923
ENSG00000107020	PLGRKT	-0.576859872	0.041560699
ENSG00000173272	MZT2A	-0.661258639	0.041601303
ENSG00000142039	CCDC97	0.536404253	0.041601303
ENSG00000278376		-0.906271269	0.041643198
ENSG00000225450		1.692333359	0.041643198
ENSG00000258301		-0.871121577	0.041831183
ENSG00000106333	PCOLCE	-0.704478829	0.041922642
ENSG00000106028	SSBP1	-0.363930247	0.042031514
ENSG00000272800		-1.712527844	0.042133816
ENSG00000272994		-0.79097072	0.042199206
ENSG00000206417	H1FX-AS1	-0.942880325	0.042202502
ENSG00000276278		-0.848138028	0.042286316
ENSG00000116514	RNF19B	0.832445142	0.042665573
ENSG00000167702	KIFC2	-0.586367448	0.042697787
ENSG00000162496	DHRS3	-2.953784123	0.042812027
ENSG00000136271	DDX56	-0.353571533	0.042812027
ENSG00000172009	THOP1	-0.569723692	0.04282179
ENSG00000101945	SUV39H1	-0.702152696	0.042843327

ENSG00000137877	SPTBN5	1.403632516	0.042903445
ENSG00000272821		-0.871258572	0.042944917
ENSG00000135632	SMYD5	-0.570038878	0.043090539
ENSG00000100731	PCNX1	0.483813871	0.043090539
ENSG00000242588		1.055293802	0.043117698
ENSG00000225079	FTH1P22	-3.140275162	0.043390405
ENSG00000126264	HCST	-0.654984793	0.043390405
ENSG00000233392		-2.558434638	0.04353287
ENSG00000107036	RIC1	0.43582337	0.043601244
ENSG00000105185	PDCD5	-0.532190421	0.043712374
ENSG00000165475	CRYL1	-0.539781942	0.043790511
ENSG00000205937	RNPS1	-0.341590993	0.043790511
ENSG00000244187	TMEM141	-0.685220211	0.043860002
ENSG00000159388	BTG2	-0.944487292	0.043897981
ENSG00000204856	FAM216A	-0.84682993	0.043897981
ENSG00000137962	ARHGAP29	1.051223956	0.043926876
ENSG00000227195	MIR663AHG	-1.411534919	0.043926876
ENSG00000104881	PPP1R13L	-0.780944437	0.043926876
ENSG00000197705	KLHL14	-2.316990813	0.04404741
ENSG00000137038	TMEM261	-0.694989459	0.044770388
ENSG00000164190	NIPBL	0.406450507	0.044985003
ENSG00000198959	TGM2	1.490232505	0.044985003
ENSG00000261584		-1.96951796	0.045126389
ENSG00000261526		-0.879334188	0.045126389
ENSG00000105173	CCNE1	-1.187241324	0.045126389
ENSG00000142252	GEMIN7	-0.802416502	0.045209433
ENSG00000106605	BLVRA	0.910923507	0.045212033
ENSG00000115944	COX7A2L	-0.356609109	0.045220017
ENSG00000155640	NA	0.951748226	0.0452337
ENSG00000198911	SREBF2	0.468409952	0.045313804
ENSG00000162913	C1orf145	-2.21393863	0.045386569
ENSG00000007541	PIGQ	-0.437298481	0.045538056
ENSG00000196236	XPNPEP3	-0.527608079	0.045538056
ENSG00000105393	BABAM1	-0.423358927	0.04567379
ENSG00000159199	ATP5G1	-0.51456978	0.045726191
ENSG00000198832	SELENOM	-1.121426814	0.045900198
ENSG00000104320	NBN	0.741137042	0.046047821
ENSG00000096746	HNRNPH3	-0.438036113	0.046047821
ENSG00000225721		-1.538240325	0.046107389
ENSG00000214182	PTMAP5	-0.588603403	0.046171062
ENSG00000263001	GTF2I	0.574629654	0.046399404
ENSG00000138623	SEMA7A	-1.368472432	0.046754566
ENSG00000250151	ARPC4-TTLL3	1.245367423	0.046975622
ENSG00000142156	COL6A1	-2.06833588	0.047099833
ENSG00000164466	SFXN1	-0.616506192	0.047297351
ENSG00000178209	PLEC	0.612449371	0.047297351
ENSG00000166133	RPUSD2	-0.802790943	0.047297351
ENSG00000188827	SLX4	0.472253708	0.047297351

ENSG00000059769	DNAJC25	-0.727050959	0.04734267
ENSG00000100348	TXN2	-0.431569558	0.047433324
ENSG00000259321		-0.893552333	0.047544863
ENSG00000135940	COX5B	-0.40428577	0.047670323
ENSG00000138796	HADH	-0.680955157	0.047670323
ENSG00000185507	IRF7	1.082613662	0.047670323
ENSG00000281026	N4BP2L2-IT2	-0.881571941	0.047670323
ENSG00000146433	TMEM181	-0.477544591	0.047703669
ENSG00000099795	NDUFB7	-0.525882947	0.047841331
ENSG00000231672	DIRC3	-1.523878666	0.047960102
ENSG00000125386	FAM193A	0.359108794	0.047960102
ENSG00000204392	LSM2	-0.629947929	0.047960102
ENSG00000108465	CDK5RAP3	-0.396188459	0.047960102
ENSG00000229980	TOB1-AS1	-0.975982243	0.048004744
ENSG00000231389	HLA-DPA1	-1.104625514	0.048179614
ENSG00000276997		0.932703116	0.048298129
ENSG00000163510	CWC22	0.462638555	0.048298129
ENSG00000130414	NDUFA10	-0.315100921	0.048298129
ENSG00000090097	PCBP4	-0.995523988	0.048298129
ENSG00000229119		-0.934256325	0.048298129
ENSG00000113761	ZNF346	0.700737438	0.048298129
ENSG00000146677		-0.736273637	0.048298129
ENSG00000239789	MRPS17	-0.600990221	0.048298129
ENSG00000279265		-0.835295678	0.048298129
ENSG00000253716	MINCR	-1.008397938	0.048298129
ENSG00000137145	DENND4C	0.502569146	0.048298129
ENSG00000136908	DPM2	-0.544418804	0.048298129
ENSG00000168062	BATF2	2.116146273	0.048298129
ENSG00000175463	TBC1D10C	-0.501811702	0.048298129
ENSG00000205846	CLEC6A	1.25079601	0.048298129
ENSG00000123104	ITPR2	0.54579055	0.048298129
ENSG00000188735	TMEM120B	-0.638201046	0.048298129
ENSG00000263264		0.854515448	0.048298129
ENSG00000213923	CSNK1E	-0.540789582	0.048298129
ENSG00000100413	POLR3H	-0.555760907	0.048298129
ENSG00000116863	ADPRHL2	0.719165735	0.048327459
ENSG00000132549	VPS13B	0.456118001	0.048327459
ENSG00000124562	SNRPC	-0.435171679	0.048385038
ENSG00000211892	IGHG4	-3.171870814	0.048421228
ENSG00000156453	PCDH1	-3.131173973	0.048505302
ENSG00000239672	NME1	-0.941710009	0.048505302
ENSG00000173786	CNP	0.831595126	0.048564692
ENSG00000178146		0.953944381	0.048565964
ENSG00000239523	MYLK-AS1	-1.295860527	0.048618705
ENSG00000131398	KCNC3	-0.876916763	0.048651234
ENSG00000235174	RPL39P3	-0.593208277	0.048837099
ENSG00000198265	HELZ	0.432727014	0.048936843
ENSG00000172215	CXCR6	-3.076835336	0.048950544

ENSG00000169857	AVEN	-0.739128209	0.048950544
ENSG00000214087	ARL16	-0.675812865	0.048950544
ENSG00000152454	ZNF256	-0.901045616	0.049059893
ENSG00000278356		-1.236502025	0.049492704
ENSG00000184384	MAML2	0.687402868	0.049581291
ENSG00000165724	ZMYND19	-0.668099105	0.049640255
ENSG00000236618	PITPNA-AS1	-0.985406381	0.049640255
ENSG00000107951	MTPAP	-0.426214188	0.049642973
ENSG00000166557	TMED3	-0.430704924	0.049642973
ENSG00000100024	UPB1	2.84838004	0.049642973
ENSG00000196655	TRAPPC4	-0.510673309	0.049802622
ENSG00000197057	DTHD1	-2.997926109	0.049825364
