# Supplementary material for: Role of CD14+ monocyte-derived oxidised mitochondrial DNA in the inflammatory interferon type 1 signature in juvenile dermatomyositis
Source: Ann Rheum Dis. 2022 Dec 23;82(5):658–69. doi: 10.1136/ard-2022-223469 (PMC10176342; doi:10.1136/ard-2022-223469)
Supplement: Supplementary data [file ard-2022-223469supp003.pdf]

Supplementary Table 2 - JDM on-treatment vs age-matched healthy control significantly DEG (p≤0.05)

Ensemble ID	hgnc_symbol	log fold change	Adjusted p value
ENSG00000180096		-2.052255975	2.09E-19
ENSG00000198821	CD247	-3.6853539	1.16E-18
ENSG00000167286	CD3D	-3.551669908	1.66E-16
ENSG00000172575	RASGRP1	-3.070795975	3.11E-15
ENSG00000211751	TRBC1	-3.419045734	2.20E-12
ENSG00000198286	CARD11	-2.796358277	5.84E-12
ENSG00000167984	NLRC3	-2.222497714	5.84E-12
ENSG00000146674	IGFBP3	-7.778707429	2.51E-11
ENSG00000211772	TRBC2	-2.989690802	2.61E-11
ENSG00000116824	CD2	-2.03086348	4.60E-11
ENSG00000160185	UBASH3A	-3.418915682	1.15E-10
ENSG00000135127	BICDL1	-2.590545427	1.25E-10
ENSG00000141293	SKAP1	-3.21275476	1.41E-10
ENSG00000107742	SPOCK2	-2.784990372	1.97E-10
ENSG00000211829	TRDC	-4.848737373	2.72E-10
ENSG00000100385	IL2RB	-4.05203449	3.01E-10
ENSG00000088387	DOCK9	-2.478453034	4.58E-10
ENSG00000107485	GATA3	-3.652286077	4.99E-10
ENSG00000111796	KLRB1	-4.239620129	4.99E-10
ENSG00000198851	CD3E	-3.527372282	1.25E-09
ENSG00000277734	TRAC	-3.315118258	1.58E-09
ENSG00000115085	ZAP70	-2.976472679	1.67E-09
ENSG00000245164	LINC00861	-3.746240843	1.67E-09
ENSG00000134954	ETS1	-1.916657616	2.44E-09
ENSG00000172005	MAL	-3.406085971	2.85E-09
ENSG00000159753	CARMIL2	-1.985304372	4.46E-09
ENSG00000139193	CD27	-3.232615925	4.63E-09
ENSG00000280135		-3.4001965	6.13E-09
ENSG00000185101	ANO9	-2.653857897	6.33E-09
ENSG00000227507	LTB	-1.880852284	2.48E-08
ENSG00000142173	COL6A2	-3.544316837	2.79E-08
ENSG00000145649	GZMA	-4.803964357	3.00E-08
ENSG00000165929	TC2N	-3.376202958	5.06E-08
ENSG00000067840	PDZD4	-3.586877431	5.94E-08
ENSG00000182866	LCK	-2.590769579	7.96E-08
ENSG00000100453	GZMB	-4.723558647	8.69E-08
ENSG00000152495	CAMK4	-2.462194431	8.92E-08
ENSG00000174917	C19orf70	-0.986150564	1.05E-07
ENSG00000101096	NFATC2	-1.775717851	1.24E-07
ENSG00000215788	TNFRSF25	-1.983277632	1.24E-07
ENSG00000100346	CACNA1I	-3.960522984	1.34E-07
ENSG00000110448	CD5	-2.699677477	1.67E-07
ENSG00000180644	PRF1	-3.978196809	1.67E-07
ENSG00000160856	FCRL3	-3.505239678	1.90E-07

ENSG00000112514	CUTA	-0.741980867	2.48E-07
ENSG00000274383		-1.929741987	2.48E-07
ENSG00000197635	DPP4	-3.321883831	4.53E-07
ENSG00000237702	TRBV3-1	-5.008641945	4.55E-07
ENSG00000181847	TIGIT	-4.389997803	4.99E-07
ENSG00000172428	COPS9	-0.788434886	5.05E-07
ENSG00000102245	CD40LG	-4.62875827	7.18E-07
ENSG00000175265	GOLGA8A	-1.927842692	7.18E-07
ENSG00000069667	RORA	-2.806765832	7.95E-07
ENSG00000130787	HIP1R	-2.317642191	9.45E-07
ENSG00000149527	PLCH2	-3.00759078	9.82E-07
ENSG00000127152	BCL11B	-3.159044333	9.85E-07
ENSG00000163600	ICOS	-3.065807292	1.89E-06
ENSG00000008517	IL32	-2.825328248	1.89E-06
ENSG00000013725	CD6	-2.598998759	1.89E-06
ENSG00000113263	ITK	-2.142174449	1.94E-06
ENSG00000089012	SIRPG	-2.841535191	1.95E-06
ENSG00000233355	CHRM3-AS2	-2.892835397	2.20E-06
ENSG00000157778	PSMG3	-0.925377317	2.32E-06
ENSG00000162894	FCMR	-2.313777415	2.39E-06
ENSG00000065675	PRKCQ	-2.086724153	2.49E-06
ENSG00000242485	MRPL20	-0.672956033	2.76E-06
ENSG00000163519	TRAT1	-2.572266006	2.93E-06
ENSG00000138795	LEF1	-2.744313922	3.13E-06
ENSG00000021300	PLEKHB1	-2.755212735	3.52E-06
ENSG00000172590	MRPL52	-1.039478499	3.75E-06
ENSG00000204922	UQCC3	-1.252279855	3.79E-06
ENSG00000168685	IL7R	-3.06562649	4.07E-06
ENSG00000115607	IL18RAP	-3.868129846	4.20E-06
ENSG00000163359	COL6A3	-3.042481553	5.16E-06
ENSG00000123545	NDUFAF4	-1.230931388	5.34E-06
ENSG00000188186	LAMTOR4	-0.896653885	5.75E-06
ENSG00000111371	SLC38A1	-1.333852375	6.23E-06
ENSG00000211747	TRBV20-1	-4.29557068	6.29E-06
ENSG00000147123	NDUFB11	-0.782339867	7.83E-06
ENSG00000108821	COL1A1	-5.839627398	1.19E-05
ENSG00000130332	LSM7	-0.840516981	1.19E-05
ENSG00000168813	ZNF507	-1.397131392	1.46E-05
ENSG00000027869	SH2D2A	-2.822038779	1.60E-05
ENSG00000099204	ABLIM1	-2.152294171	1.64E-05
ENSG00000073861	TBX21	-3.297294039	1.64E-05
ENSG00000100351	GRAP2	-2.003475841	1.78E-05
ENSG00000197540	GZMM	-2.01951176	1.84E-05
ENSG00000183648	NDUFB1	-0.807264541	2.03E-05
ENSG00000184260	HIST2H2AC	-1.373431101	2.65E-05
ENSG00000090266	NDUFB2	-0.824472141	2.75E-05
ENSG00000135426	TESPA1	-1.629451726	3.04E-05
ENSG00000116106	EPHA4	-3.313173692	3.48E-05

ENSG00000167515	TRAPPC2L	-0.735897306	3.59E-05
ENSG00000145494	NDUFS6	-1.015043482	3.67E-05
ENSG00000269911		-4.606404955	3.99E-05
ENSG00000162910	MRPL55	-0.83495544	4.65E-05
ENSG00000198780	FAM169A	-3.221200275	4.91E-05
ENSG00000100393	EP300	0.906548266	4.97E-05
ENSG00000149932	TMEM219	-0.585507926	5.00E-05
ENSG00000106624	AEBP1	-2.687495998	5.28E-05
ENSG00000280120		-1.334309218	5.39E-05
ENSG00000183918	SH2D1A	-3.218760784	5.93E-05
ENSG00000099624	ATP5D	-0.885647999	6.05E-05
ENSG00000185697	MYBL1	-2.184309898	6.07E-05
ENSG00000135972	MRPS9	-0.835375097	6.25E-05
ENSG00000237943	PRKCQ-AS1	-1.934661782	6.37E-05
ENSG00000188322	SBK1	-2.122566913	6.37E-05
ENSG00000081059	TCF7	-2.481024953	6.65E-05
ENSG00000106211	HSPB1	-1.187404627	6.97E-05
ENSG00000124203	ZNF831	-2.320006272	6.97E-05
ENSG00000172673	THEMIS	-2.063839691	7.28E-05
ENSG00000130635	COL5A1	-3.973796372	7.31E-05
ENSG00000007520	TSR3	-0.855987865	7.80E-05
ENSG00000174500	GCSAM	-4.046367946	7.97E-05
ENSG00000105369	CD79A	-2.076747185	8.79E-05
ENSG00000211655	IGLV1-36	-4.72487216	9.00E-05
ENSG00000137078	SIT1	-3.347098458	9.59E-05
ENSG00000131116	ZNF428	-0.760473447	9.82E-05
ENSG00000124181	PLCG1	-1.526371087	9.97E-05
ENSG00000214253	FIS1	-0.645207704	0.000104149
ENSG00000105379	ETFB	-0.859182741	0.000106419
ENSG00000076554	TPD52	-1.97094825	0.000121484
ENSG00000156110	ADK	-1.196227224	0.00012391
ENSG00000137288	UQCC2	-1.251893378	0.000125616
ENSG00000107223	EDF1	-0.632596227	0.000131092
ENSG00000110717	NDUFS8	-0.678684054	0.000140299
ENSG00000157570	TSPAN18	-3.716968277	0.000140877
ENSG00000146285	SCML4	-2.029414028	0.000144535
ENSG00000269293	ZSCAN16-AS1	-0.887401584	0.000145942
ENSG00000175854	SWI5	-0.98164604	0.000150055
ENSG00000211648	IGLV1-47	-4.162451755	0.000151866
ENSG00000154764	WNT7A	-4.452672944	0.000157734
ENSG00000135736	CCDC102A	-3.706893682	0.000177087
ENSG00000270189		-1.50166595	0.000185057
ENSG00000186265	BTLA	-2.860145709	0.000191827
ENSG00000173208	ABCD2	-2.674134864	0.000203269
ENSG00000124198	ARFGEF2	0.762532979	0.000206119
ENSG00000108961	RANGRF	-0.715893364	0.000220644
ENSG00000132704	FCRL2	-2.358634214	0.0002207
ENSG00000102030	NAA10	-0.808561228	0.0002207

ENSG00000103266	STUB1	-0.614031322	0.00022242
ENSG00000149541	B3GAT3	-0.715026991	0.000231571
ENSG00000189171	S100A13	-1.276796156	0.000232557
ENSG00000182004	SNRPE	-0.702748144	0.000241368
ENSG00000105851	PIK3CG	1.072334784	0.000256536
ENSG00000124172	ATP5E	-0.530371079	0.000256883
ENSG00000100075	SLC25A1	-0.814500073	0.000269424
ENSG00000142168	SOD1	-0.569101957	0.000277946
ENSG00000220205	VAMP2	-0.674680527	0.000278827
ENSG00000112182	BACH2	-1.875630295	0.000283723
ENSG00000268205		-0.897853759	0.000283723
ENSG00000241837	ATP5O	-0.627082799	0.000287438
ENSG00000112651	MRPL2	-0.754335639	0.000291318
ENSG00000278030	TRBV7-9	-4.257279795	0.000302609
ENSG00000143322	ABL2	0.930760122	0.00030897
ENSG00000170486	KRT72	-3.312303799	0.000310573
ENSG00000170291	ELP5	-0.585991686	0.000310573
ENSG00000127184	COX7C	-0.548030399	0.00031646
ENSG00000106355	LSM5	-0.69826303	0.000323502
ENSG00000137441	FGFBP2	-3.96553518	0.000341439
ENSG00000235162	C12orf75	-2.376290004	0.000341439
ENSG00000178982	EIF3K	-0.556702503	0.000346212
ENSG00000213626	LBH	-1.73764558	0.00035002
ENSG00000169397	RNASE3	-2.905581045	0.000353281
ENSG00000109390	NDUFC1	-0.778152395	0.000357875
ENSG00000280237	MIR4697HG	-3.6930875	0.000370798
ENSG00000165272	AQP3	-2.503749656	0.000388947
ENSG00000272034	SNORD14A	-1.246736407	0.000396447
ENSG00000161405	IKZF3	-1.248053926	0.000396447
ENSG00000130313	PGLS	-0.676717342	0.000409576
ENSG00000204392	LSM2	-1.002115829	0.000416218
ENSG00000143793	C1orf35	-0.872094409	0.000420175
ENSG00000106400	ZNHIT1	-0.695513813	0.000424608
ENSG00000176476	SGF29	-0.685619041	0.000424608
ENSG00000170906	NDUFA3	-0.663695681	0.000426697
ENSG00000153283	CD96	-2.727977245	0.000428379
ENSG00000123066	MED13L	0.969391831	0.000429434
ENSG00000254521	SIGLEC12	-3.383033923	0.000459732
ENSG00000243449	C4orf48	-1.427472708	0.000475129
ENSG00000167792	NDUFV1	-0.572069152	0.000498831
ENSG00000130748	TMEM160	-1.336986115	0.000500391
ENSG00000100450	GZMH	-4.033767324	0.00050574
ENSG00000135940	COX5B	-0.631352572	0.000505886
ENSG00000100372	SLC25A17	-0.872895189	0.000505886
ENSG00000228903	RASA4CP	-1.021244905	0.000523693
ENSG00000115523	GNLY	-3.381977425	0.00053024
ENSG00000145912	NHP2	-0.73974099	0.000533554
ENSG00000178922	HYI	-1.471570573	0.000585641

ENSG00000196456	ZNF775	-1.261727966	0.000600014
ENSG00000166136	NDUFB8	-0.630795623	0.000601971
ENSG00000173915	USMG5	-0.713718359	0.000602892
ENSG00000255198	SNHG9	-0.998551623	0.000612206
ENSG00000235621	LINC00494	-4.271244394	0.000612206
ENSG00000130731	METTL26	-0.900618751	0.00061549
ENSG00000125901	MRPS26	-0.941146865	0.000620267
ENSG00000130770	ATPIF1	-0.685815934	0.000630569
ENSG00000113088	GZMK	-3.38635086	0.000630569
ENSG00000211789	TRAV12-2	-3.568982501	0.000630569
ENSG00000135919	SERPINE2	-2.691219945	0.000660959
ENSG00000124782	RREB1	0.647120023	0.000660959
ENSG00000156411	C14orf2	-0.597727634	0.000660959
ENSG00000128185	DGCR6L	-0.863877878	0.000660959
ENSG00000050165	DKK3	-4.787872624	0.000663704
ENSG00000224877	NDUFAF8	-1.104881901	0.000701391
ENSG00000105258	POLR2I	-0.891264653	0.000707797
ENSG00000169976	SF3B5	-0.580444487	0.000721086
ENSG00000109686	SH3D19	-3.202135976	0.00072893
ENSG00000196498	NCOR2	0.790243881	0.000776069
ENSG00000173141	MRPL57	-0.769830645	0.000776121
ENSG00000034053	APBA2	-1.941769966	0.000803438
ENSG00000115685	PPP1R7	-0.618937955	0.000871845
ENSG00000136573	BLK	-2.535242251	0.000875463
ENSG00000174080	CTSF	-1.358855208	0.00092528
ENSG00000100027	YPEL1	-1.531721793	0.000929974
ENSG00000161381	PLXDC1	-1.758478027	0.000940066
ENSG00000129559	NEDD8	-0.618396927	0.000944673
ENSG00000204228	HSD17B8	-1.326715677	0.000949783
ENSG00000153207	AHCTF1	1.46492768	0.000964021
ENSG00000101082	SLA2	-1.510618291	0.000979842
ENSG00000120733	KDM3B	0.617003549	0.000986782
ENSG00000148362	C9orf142	-0.805896401	0.000986782
ENSG00000168646	AXIN2	-3.805669646	0.00101752
ENSG00000008083	JARID2	1.484396023	0.001018111
ENSG00000198258	UBL5	-0.518241849	0.001019919
ENSG00000158552	ZFAND2B	-0.530277112	0.001025477
ENSG00000140332	TLE3	1.13848898	0.001059245
ENSG00000232442		-1.292940585	0.001062457
ENSG00000178980	SELENOW	-0.592817766	0.001066345
ENSG00000188243	COMMD6	-0.529169941	0.001085746
ENSG00000163430	FSTL1	-3.126233143	0.001086546
ENSG00000205138	SDHAF1	-1.096661584	0.001088015
ENSG00000168002	POLR2G	-0.532471779	0.001096663
ENSG00000210151	MT-TS1	-2.200485555	0.00110886
ENSG00000170430	MGMT	-1.03459222	0.001126459
ENSG00000276136		-1.500710793	0.001137814
ENSG00000143515	ATP8B2	-1.300025565	0.001159268

ENSG00000067900	ROCK1	0.740341417	0.001159268
ENSG00000082458	DLG3	-2.943436749	0.001179363
ENSG00000087191	PSMC5	-0.552768277	0.001179363
ENSG00000173436	MINOS1	-0.646132624	0.001198012
ENSG00000106089	STX1A	4.590319195	0.001202837
ENSG00000259865		-0.875600656	0.001235819
ENSG00000099977	DDT	-0.821115861	0.001235819
ENSG00000250251	PKD1P6	1.093497086	0.001241575
ENSG00000178498	DTX3	-1.539877493	0.00125692
ENSG00000055609	KMT2C	0.798575161	0.00129472
ENSG00000136908	DPM2	-0.80553397	0.00129472
ENSG00000232869	TRBV29-1	-2.85196455	0.001310797
ENSG00000107872	FBXL15	-0.924591084	0.001321719
ENSG00000163534	FCRL1	-2.423717463	0.001386628
ENSG00000142546	NOSIP	-0.941657275	0.001420714
ENSG00000164692	COL1A2	-4.352695936	0.001435805
ENSG00000211896	IGHG1	-3.283908874	0.001435805
ENSG00000211797	TRAV17	-4.051771056	0.00146041
ENSG00000174946	GPR171	-3.131352456	0.001476075
ENSG00000106399	RPA3	-0.813822282	0.001476075
ENSG00000174886	NDUFA11	-0.941108386	0.001531853
ENSG00000148290	SURF1	-0.558868668	0.00153948
ENSG00000121671	CRY2	1.223840349	0.001581574
ENSG00000134809	TIMM10	-0.936517161	0.001586879
ENSG00000196655	TRAPPC4	-0.753344316	0.001586879
ENSG00000085231	AK6	-0.933091766	0.001587935
ENSG00000065526	SPEN	0.708670026	0.00162693
ENSG00000124151	NCOA3	0.657226203	0.001668737
ENSG00000196705	ZNF431	-0.693030839	0.001692454
ENSG00000105518	TMEM205	-0.736130692	0.001743982
ENSG00000130856	ZNF236	0.65320043	0.001744548
ENSG00000090273	NUDC	-0.61733871	0.001745767
ENSG00000140990	NDUFB10	-0.738105046	0.001755565
ENSG00000229809	ZNF688	-0.692887812	0.001793471
ENSG00000134545	KLRC1	-4.079430013	0.00179426
ENSG00000211750	TRBV24-1	-4.04677386	0.001844893
ENSG00000072364	AFF4	0.882034479	0.001890326
ENSG00000148291	SURF2	-0.711917554	0.001890326
ENSG00000187630	DHRS4L2	-0.951131089	0.001890326
ENSG00000164919	COX6C	-0.594725036	0.001996994
ENSG00000206828		-1.402511589	0.002073839
ENSG00000211899	IGHM	-2.516390861	0.00210415
ENSG00000132326	PER2	1.617704987	0.00211366
ENSG00000070961	ATP2B1	1.97538625	0.00211366
ENSG00000272579		-1.23634543	0.00214336
ENSG00000091972	CD200	-3.208271292	0.002217994
ENSG00000180992	MRPL14	-0.792139988	0.002217994
ENSG00000167799	NUDT8	-1.689812174	0.002217994

ENSG00000258199		-0.756805886	0.002217994
ENSG00000259959		-1.177368418	0.002219683
ENSG00000247774	PCED1B-AS1	-0.935354286	0.002219683
ENSG00000211898	IGHD	-2.516143722	0.002219683
ENSG00000095906	NUBP2	-0.704321541	0.002219683
ENSG00000105404	RABAC1	-0.71092015	0.002219683
ENSG00000238142		-1.232822857	0.002230551
ENSG00000213139	CRYGS	-1.076148013	0.002230551
ENSG00000211677	IGLC2	-3.176288711	0.002230551
ENSG00000164442	CITED2	1.009950175	0.002238492
ENSG00000151131	C12orf45	-0.74306226	0.002238492
ENSG00000164008	C1orf50	-0.804396737	0.002246465
ENSG00000211592	IGKC	-2.783545648	0.002246465
ENSG00000135960	EDAR	-2.855390559	0.002246465
ENSG00000066379	ZNRD1	-0.806445845	0.002246465
ENSG00000273466		-1.09284548	0.002253779
ENSG00000111674	ENO2	-1.749155517	0.002326211
ENSG00000169155	ZBTB43	1.527093901	0.002378582
ENSG00000156873	PHKG2	-0.532089104	0.002385462
ENSG00000125743	SNRPD2	-0.489067056	0.002385462
ENSG00000235499		1.888817223	0.002385723
ENSG00000160654	CD3G	-1.342797889	0.002385723
ENSG00000180089	TMEM86B	-0.911842024	0.002385723
ENSG00000180530	NRIP1	1.215876229	0.002385723
ENSG00000107833	NPM3	-0.969928202	0.002409829
ENSG00000241351	IGKV3-11	-3.615749194	0.002422507
ENSG00000160124	CCDC58	-0.92968934	0.002471394
ENSG00000128951	DUT	-0.889271259	0.002508397
ENSG00000185920	PTCH1	-2.127072769	0.002536447
ENSG00000215845	TSTD1	-0.975940459	0.002573252
ENSG00000204580	DDR1	-1.354183492	0.002573252
ENSG00000156650	KAT6B	0.721919037	0.002573252
ENSG00000143297	FCRL5	-1.841088613	0.002590936
ENSG00000101224	CDC25B	-0.683124858	0.002644042
ENSG00000205352	PRR13	-0.499615457	0.002659719
ENSG00000168061	SAC3D1	-1.605755115	0.0026856
ENSG00000213904	LIPE-AS1	-1.074855614	0.0026856
ENSG00000186501	TMEM222	-0.571760714	0.00270021
ENSG00000175602	CCDC85B	-0.840267144	0.00270021
ENSG00000141759	TXNL4A	-0.511850743	0.00270021
ENSG00000220201	ZGLP1	-1.232843285	0.00270021
ENSG00000227191	TRGC2	-2.280472747	0.002701707
ENSG00000237886	NALT1	-1.317446891	0.002707822
ENSG00000166912	MTMR10	0.619269305	0.002728082
ENSG00000099797	TECR	-0.749774487	0.002803735
ENSG00000002822	MAD1L1	-0.941831532	0.002863033
ENSG00000113319	RASGRF2	-1.911608929	0.002871806
ENSG00000113712	CSNK1A1	0.631575602	0.002886691

ENSG00000175193	PARL	-0.46027997	0.00290763
ENSG00000078596	ITM2A	-1.678050878	0.00290763
ENSG00000235245		1.18866358	0.002909427
ENSG00000071655	MBD3	-0.630403085	0.002979797
ENSG00000124201	ZNFX1	0.892768364	0.003031446
ENSG00000125650	PSPN	-1.058034777	0.003031446
ENSG00000173113	TRMT112	-0.572052472	0.003059826
ENSG00000115317	HTRA2	-0.68723354	0.003090521
ENSG00000118900	UBN1	0.503806277	0.003092371
ENSG00000143543	JTB	-0.474491257	0.003142449
ENSG00000164405	UQCRQ	-0.61097571	0.003180774
ENSG00000064490	RFXANK	-0.551589636	0.003258643
ENSG00000163794	UCN	-1.312929891	0.003284201
ENSG00000071859	FAM50A	-0.476889204	0.003284201
ENSG00000186049	KRT73	-3.206404934	0.00331186
ENSG00000091409	ITGA6	-1.636973357	0.003317157
ENSG00000117419	ERI3	-0.513444099	0.003318012
ENSG00000262814	MRPL12	-1.209334265	0.003350118
ENSG00000042753	AP2S1	-0.746866157	0.003350118
ENSG00000166405	RIC3	-1.752528741	0.003431247
ENSG00000211788	TRAV13-1	-3.553683346	0.003431247
ENSG00000174547	MRPL11	-0.595259725	0.003436893
ENSG00000211795	TRAV8-6	-3.805808293	0.003436893
ENSG00000214026	MRPL23	-0.789860978	0.003468972
ENSG00000126756	UXT	-0.523879046	0.003552209
ENSG00000188690	UROS	-0.552418701	0.003552209
ENSG00000280649		-1.069214829	0.003555564
ENSG00000142197	DOPEY2	0.716765403	0.003584705
ENSG00000156030	ELMSAN1	0.844193989	0.003618581
ENSG00000279865		-0.962117861	0.003623064
ENSG00000272800		-2.492216361	0.003623358
ENSG00000103254	FAM173A	-1.145538369	0.003651756
ENSG00000167258	CDK12	0.527733912	0.003651756
ENSG00000156639	ZFAND3	0.559111948	0.003698614
ENSG00000156738	MS4A1	-2.391621523	0.003698614
ENSG00000005339	CREBBP	0.63934494	0.003698614
ENSG00000271109		-2.376468688	0.003711817
ENSG00000106133	NSUN5P2	-1.307507949	0.003742354
ENSG00000099795	NDUFB7	-0.71305014	0.003746865
ENSG00000009413	REV3L	0.676640564	0.003780849
ENSG00000110711	AIP	-0.588127852	0.003811454
ENSG00000172586	CHCHD1	-0.959143303	0.003828244
ENSG00000143727	ACP1	-0.457656693	0.003831007
ENSG00000056050	HPF1	-1.194549826	0.003831007
ENSG00000257727	CNPY2	-0.602750121	0.003831007
ENSG00000184076	UQCR10	-0.694618584	0.003831007
ENSG00000210154	MT-TD	-1.64971623	0.003851474
ENSG00000074071	MRPS34	-0.879326358	0.003859763

ENSG00000115604	IL18R1	-2.291033659	0.003862624
ENSG00000109452	INPP4B	-1.778829582	0.003862624
ENSG00000234389		-3.587673289	0.003887425
ENSG00000171443	ZNF524	-0.7577136	0.003894411
ENSG00000029639	TFB1M	-1.05552499	0.003955229
ENSG00000168229	PTGDR	-3.346215593	0.004155694
ENSG00000265206	MIR142	-1.341339875	0.004208594
ENSG00000166557	TMED3	-0.586738861	0.004209418
ENSG00000196998	WDR45	-0.436944407	0.004247827
ENSG00000090238	YPEL3	-0.733561139	0.004298452
ENSG00000156983	BRPF1	0.635801863	0.004313453
ENSG00000111344	RASAL1	6.217511069	0.004375212
ENSG00000172500	FIBP	-0.638607312	0.004547109
ENSG00000205981	DNAJC19	-0.784600758	0.004598665
ENSG00000110497	AMBRA1	0.542060184	0.004598665
ENSG00000151500	THYN1	-0.919232373	0.004598665
ENSG00000182173	TSEN54	-0.866716776	0.004598665
ENSG00000133657	ATP13A3	1.464715552	0.004616096
ENSG00000198931	APRT	-0.623803831	0.004616096
ENSG00000175756	AURKAIP1	-0.569076116	0.004632373
ENSG00000131495	NDUFA2	-0.654725751	0.004653323
ENSG00000139537	CCDC65	-3.255032493	0.004672159
ENSG00000176903	PNMA1	1.067291673	0.004679466
ENSG00000125877	ITPA	-0.659170325	0.004679466
ENSG00000117322	CR2	-2.058452531	0.004697359
ENSG00000168090	COPS6	-0.520882211	0.004697359
ENSG00000169020	ATP5I	-0.581944407	0.004744626
ENSG00000163376	KBTBD8	1.69629381	0.004746086
ENSG00000070047	PHRF1	0.612452552	0.00478137
ENSG00000104872	PIH1D1	-0.576006924	0.004872391
ENSG00000213977	TAX1BP3	-0.896311437	0.004889339
ENSG00000186010	NDUFA13	-0.688117962	0.004922006
ENSG00000069956	MAPK6	1.602058488	0.004974083
ENSG00000172922	RNASEH2C	-0.87678512	0.004976652
ENSG00000167578	RAB4B	-0.735835132	0.004976652
ENSG00000131591	C1orf159	-0.64080934	0.005041661
ENSG00000198932	GPRASP1	-1.350685503	0.005041661
ENSG00000134899	ERCC5	-0.588623353	0.005041661
ENSG00000099385	BCL7C	-0.569823388	0.005041661
ENSG00000197457	STMN3	-1.053407853	0.005041661
ENSG00000114023	FAM162A	-0.691068522	0.005252977
ENSG00000178449	COX14	-0.690788727	0.005252977
ENSG00000130590	SAMD10	-1.307193	0.005261554
ENSG00000034713	GABARAPL2	-0.561366346	0.005265767
ENSG00000144214	LYG1	-1.120042901	0.00529096
ENSG00000104490	NCALD	-2.023883943	0.005293655
ENSG00000084112	SSH1	0.753636123	0.005359294
ENSG00000260228		-1.433661047	0.005382074

ENSG00000125445	MRPS7	-0.559504093	0.005382074
ENSG00000163788	SNRK	0.718576358	0.005393953
ENSG00000104517	UBR5	0.492054034	0.005395523
ENSG00000070785	EIF2B3	-0.88466032	0.00542296
ENSG00000186854	TRABD2A	-1.331249571	0.005429133
ENSG00000179526	SHARPIN	-0.548163304	0.005473194
ENSG00000118640	VAMP8	-0.685860856	0.005483552
ENSG00000245910	SNHG6	-0.551261653	0.005483552
ENSG00000100647	SUSD6	0.565222141	0.005483552
ENSG00000250318		-1.206752337	0.005522994
ENSG00000125611	CHCHD5	-0.725028072	0.005523755
ENSG00000255026		-2.264980191	0.005523755
ENSG00000100575	TIMM9	-0.60669243	0.005523755
ENSG00000235374	SSR4P1	-1.330021487	0.005523755
ENSG00000140992	PDPK1	0.520647984	0.005657625
ENSG00000106268	NUDT1	-0.815513472	0.005690673
ENSG00000150456	EEF1AKMT1	-1.281259874	0.005690673
ENSG00000137145	DENND4C	0.676878109	0.005760933
ENSG00000138821	SLC39A8	2.9969628	0.005789507
ENSG00000225864	HCG4P11	-2.075218108	0.005789507
ENSG00000102471	NDFIP2	-2.908439267	0.005789507
ENSG00000103024	NME3	-0.957076304	0.005789507
ENSG00000188599	NPIPP1	-1.032795375	0.005789507
ENSG00000167863	ATP5H	-0.547746035	0.005789507
ENSG00000164483	SAMD3	-2.057449628	0.005810141
ENSG00000211689	TRGC1	-2.531900415	0.005814115
ENSG00000159958	TNFRSF13C	-1.780009242	0.005852956
ENSG00000262049		-0.75359782	0.005874615
ENSG00000169884	WNT10B	-1.73390122	0.005889351
ENSG00000196683	TOMM7	-0.644165299	0.005916319
ENSG00000154122	ANKH	1.370777137	0.005924707
ENSG00000183826	BTBD9	0.815322268	0.005958191
ENSG00000008294	SPAG9	0.794114758	0.006039216
ENSG00000213654	GPSM3	-0.430822002	0.006218697
ENSG00000144645	OSBPL10	-1.758380303	0.006218903
ENSG00000125458	NT5C	-0.743265943	0.006225642
ENSG00000185761	ADAMTSL5	-2.661834133	0.006259272
ENSG00000273361		-1.100278211	0.006447564
ENSG00000171159	C9orf16	-0.627737641	0.006461552
ENSG00000107672	NSMCE4A	-0.599851448	0.006466577
ENSG00000122140	MRPS2	-0.636939704	0.006480008
ENSG00000250317	SMIM20	-0.729397288	0.006492425
ENSG00000180185	FAHD1	-0.968801184	0.006517199
ENSG00000002330	BAD	-0.581869768	0.006544074
ENSG00000243147	MRPL33	-0.536515258	0.006545909
ENSG00000213465	ARL2	-0.981755571	0.006588779
ENSG00000161677	JOSD2	-0.920446461	0.006604838
ENSG00000179361	ARID3B	0.927101168	0.006613523

ENSG00000006015	C19orf60	-0.780166117	0.006613523
ENSG00000174137	FAM53A	-1.375105073	0.006625594
ENSG00000124802	EEF1E1	-0.955883398	0.006667583
ENSG00000174109	C16orf91	-0.741345419	0.006716842
ENSG00000007312	CD79B	-1.347501038	0.0068066
ENSG00000141698	NT5C3B	-1.002899219	0.006808457
ENSG00000179715	PCED1B	-1.320473421	0.006820642
ENSG00000089057	SLC23A2	0.760865366	0.006831429
ENSG00000183506	PI4KAP2	0.893927285	0.006852984
ENSG00000114388	NPRL2	-0.623300389	0.006871054
ENSG00000176386	CDC26	-0.60545355	0.006871054
ENSG00000256576		-1.17913261	0.006929097
ENSG00000169660	HEXDC	-1.092809856	0.00695082
ENSG00000038219	BOD1L1	0.705169705	0.00697272
ENSG00000211746	TRBV19	-3.353187647	0.00697272
ENSG00000126353	CCR7	-1.751876737	0.00697272
ENSG00000163382	NAXE	-0.62706082	0.006983897
ENSG00000278942		-3.335752477	0.006983897
ENSG00000160318	CLDND2	-1.99093826	0.007042153
ENSG00000126088	UROD	-0.559917552	0.00704863
ENSG00000274752	TRBV12-3	-3.352646309	0.007049759
ENSG00000246898	LINC00920	-1.70753076	0.007090887
ENSG00000275055		-1.326554878	0.007090887
ENSG00000079337	RAPGEF3	-2.291477983	0.007138
ENSG00000167283	ATP5L	-0.39914685	0.007189213
ENSG00000121851	POLR3GL	-0.68194242	0.007224777
ENSG00000196313	POM121	0.456303883	0.007226902
ENSG00000211734	TRBV5-1	-3.083916417	0.007226902
ENSG00000011590	ZBTB32	-3.803327816	0.0072278
ENSG00000165006	UBAP1	1.016658482	0.00739764
ENSG00000122482	ZNF644	0.738126182	0.007402587
ENSG00000156603	MED19	-0.882511586	0.007402587
ENSG00000169288	MRPL1	-0.755986704	0.007405513
ENSG00000109111	SUPT6H	0.700370583	0.007405513
ENSG00000126768	TIMM17B	-0.480768	0.007419159
ENSG00000052749	RRP12	0.998350248	0.007481409
ENSG00000054523	KIF1B	0.850753922	0.007499987
ENSG00000117298	ECE1	0.684569348	0.007499987
ENSG00000167548	KMT2D	0.73715763	0.007499987
ENSG00000123374	CDK2	-0.702921321	0.007499987
ENSG00000179085	DPM3	-0.733500172	0.007541218
ENSG00000118689	FOXO3	1.100354544	0.007541218
ENSG00000118523	CTGF	-3.998429189	0.007541218
ENSG00000148308	GTF3C5	-0.396713262	0.007541218
ENSG00000244242	IFITM10	3.156558887	0.007541218
ENSG00000185475	TMEM179B	-0.528857069	0.007541218
ENSG00000069493	CLEC2D	-0.90173644	0.007541218
ENSG00000159884	CCDC107	-0.696373822	0.007634489

ENSG00000171222	SCAND1	-0.669945727	0.007694843
ENSG00000158373	HIST1H2BD	-1.387658487	0.007774524
ENSG00000085998	POMGNT1	-0.647726487	0.007945138
ENSG00000127720	METTL25	-0.741156437	0.00798012
ENSG00000123144	C19orf43	-0.503997248	0.008019324
ENSG00000151012	SLC7A11	4.902298063	0.008051755
ENSG00000197057	DTHD1	-3.805928592	0.008116641
ENSG00000102908	NFAT5	0.981381277	0.008116641
ENSG00000256053	APOPT1	-0.658065501	0.008183364
ENSG00000071994	PDCD2	-0.60987101	0.008244768
ENSG00000065057	NTHL1	-1.184372773	0.008314765
ENSG00000184897	H1FX	-0.775405838	0.008331622
ENSG00000092010	PSME1	-0.511324215	0.008331622
ENSG00000262919	FAM58A	-0.627878435	0.008362198
ENSG00000159055	MIS18A	-1.043089659	0.008366143
ENSG00000204475	NCR3	-2.116045347	0.00838112
ENSG00000163882	POLR2H	-0.909984956	0.00841218
ENSG00000103522	IL21R	-1.203079914	0.008440305
ENSG00000130779	CLIP1	0.546550403	0.008508457
ENSG00000112695	COX7A2	-0.495596793	0.008522097
ENSG00000160271	RALGDS	1.141697686	0.008577309
ENSG00000211679	IGLC3	-2.968040453	0.008577309
ENSG00000104823	ECH1	-0.718591918	0.008625612
ENSG00000181222	POLR2A	0.692733328	0.008689805
ENSG00000229980	TOB1-AS1	-1.256182501	0.008689805
ENSG00000154102	C16orf74	-1.149512594	0.008694687
ENSG00000130595	TNNT3	2.348720037	0.00872194
ENSG00000260404		1.022837811	0.008740797
ENSG00000182154	MRPL41	-0.848860402	0.008740797
ENSG00000119640	ACYP1	-1.116742955	0.008740797
ENSG00000198171	DDRGK1	-0.49552446	0.008740797
ENSG00000171988	JMJD1C	1.010236425	0.008917932
ENSG00000205643	CDPF1	-1.128518232	0.008956775
ENSG00000153406	NMRAL1	-0.595889722	0.008987048
ENSG00000126456	IRF3	-0.714217599	0.008998585
ENSG00000186660	ZFP91	0.597590983	0.009006979
ENSG00000204237	OXLD1	-1.025946823	0.009073642
ENSG00000110713	NUP98	0.882132592	0.009158382
ENSG00000164053	ATRIP	-0.728953802	0.009224283
ENSG00000078487	ZCWPW1	-0.845208243	0.009224283
ENSG00000123154	WDR83	-0.614399137	0.009242716
ENSG00000100429	HDAC10	-0.731867229	0.009335951
ENSG00000100836	PABPN1	-0.428739719	0.009491196
ENSG00000092841	MYL6	-0.504395967	0.009498655
ENSG00000173281	PPP1R3B	1.243839275	0.009543041
ENSG00000096070	BRPF3	0.707036581	0.009596317
ENSG00000101391	CDK5RAP1	-0.420646444	0.009629149
ENSG00000103145	HCFC1R1	-0.688743261	0.009640343

ENSG00000151694	ADAM17	0.569148211	0.009906186
ENSG00000004478	FKBP4	-0.89182819	0.009912238
ENSG00000267858	MZF1-AS1	-1.054222285	0.009939332
ENSG00000074755	ZZEF1	0.513853931	0.009965996
ENSG00000235576		-3.361892329	0.009986244
ENSG00000197180		-0.988773749	0.009986244
ENSG00000254815		-2.504189188	0.009986244
ENSG00000163832	ELP6	-0.764820394	0.010119668
ENSG00000167106	FAM102A	-0.873366138	0.010119668
ENSG00000129158	SERGEF	-0.764695252	0.010119668
ENSG00000185947	ZNF267	1.292854277	0.010119668
ENSG00000169738	DCXR	-0.782485701	0.010119668
ENSG00000007080	CCDC124	-0.542143805	0.010119668
ENSG00000218283	MORF4L1P1	0.715295624	0.010127251
ENSG00000211787	TRAV8-3	-3.515642452	0.010127251
ENSG00000211803	TRAV23DV6	-3.081398705	0.010127251
ENSG00000270550	IGHV3-30	-3.542578363	0.010127251
ENSG00000131873	CHSY1	1.018118022	0.010127251
ENSG00000211796	TRAV16	-3.512417221	0.010149637
ENSG00000139428	MMAB	-0.661591617	0.010151954
ENSG00000092148	HECTD1	0.466240494	0.010151954
ENSG00000007255	TRAPPC6A	-0.76496011	0.010151954
ENSG00000105552	BCAT2	-0.74845306	0.010177324
ENSG00000132185	FCRLA	-2.305145584	0.010194588
ENSG00000008018	PSMB1	-0.416022639	0.010304931
ENSG00000211793	TRAV9-2	-3.517548871	0.010432614
ENSG00000198900	TOP1	0.836552447	0.010465627
ENSG00000160218	TRAPPC10	0.637481657	0.010513856
ENSG00000100258	LMF2	-0.443550151	0.010547929
ENSG00000160075	SSU72	-0.348001585	0.010582203
ENSG00000167664	TMIGD2	-2.086310356	0.010582203
ENSG00000100426	ZBED4	0.497435326	0.010582203
ENSG00000157933	SKI	0.935934355	0.010665116
ENSG00000148396	SEC16A	0.643827032	0.010665116
ENSG00000141506	PIK3R5	0.828460647	0.010665116
ENSG00000228623	ZNF883	-1.996685019	0.010667322
ENSG00000244187	TMEM141	-0.829574021	0.010714819
ENSG00000211776	TRAV2	-3.513699695	0.010789534
ENSG00000278784		-0.768907227	0.01102224
ENSG00000169592	INO80E	-0.76604936	0.01105392
ENSG00000128218	VPREB3	-2.928684917	0.011115322
ENSG00000271869		-1.023913028	0.011145692
ENSG00000232788		-1.504401745	0.011197599
ENSG00000184281	TSSC4	-0.488502198	0.011209778
ENSG00000134709	HOOK1	-2.47967242	0.011225555
ENSG00000119431	HDHD3	-0.979936133	0.011238674
ENSG00000149474	KAT14	-0.716682945	0.011242405
ENSG00000181036	FCRL6	-3.124764485	0.011258843

ENSG00000164151	ICE1	0.511579922	0.011258843
ENSG00000211724	TRBV6-6	-3.503652548	0.011258843
ENSG00000089195	TRMT6	0.956671269	0.011258843
ENSG00000184702	Sep-05	5.290109037	0.011258843
ENSG00000136942	RPL35	-0.54378298	0.011451824
ENSG00000236778	INTS6-AS1	-0.795329139	0.011456401
ENSG00000211965	IGHV3-49	-3.335291477	0.011456401
ENSG00000140365	COMMD4	-0.7034297	0.011456401
ENSG00000104907	TRMT1	-0.553588205	0.011456401
ENSG00000152082	MZT2B	-0.623376319	0.011548949
ENSG00000169583	CLIC3	-2.689926713	0.011548949
ENSG00000176101	SSNA1	-0.577695533	0.011548949
ENSG00000149016	TUT1	-0.788231419	0.011596103
ENSG00000242660		-1.169198951	0.011596103
ENSG00000130159	ECSIT	-0.505207333	0.011596103
ENSG00000151135	TMEM263	-0.876701318	0.011648517
ENSG00000268861		2.61984339	0.011661629
ENSG00000182196	ARL6IP4	-1.001977368	0.011665313
ENSG00000169641	LUZP1	0.625289533	0.01167294
ENSG00000205544	TMEM256	-0.792489914	0.01167294
ENSG00000163508	EOMES	-3.275261219	0.011694319
ENSG00000054148	PHPT1	-0.776620283	0.01177888
ENSG00000186480	INSIG1	1.315479277	0.011783712
ENSG00000116251	RPL22	-0.463786509	0.011845418
ENSG00000128739	SNRPN	-0.707532418	0.011878046
ENSG00000116353	MECR	-0.807489511	0.011878208
ENSG00000149357	LAMTOR1	-0.434026736	0.011917173
ENSG00000100150	DEPDC5	0.52941313	0.011942502
ENSG00000232119	MCTS1	-0.742378736	0.01198379
ENSG00000187193	MT1X	-1.121972998	0.01198379
ENSG00000130755	GMFG	-0.501314106	0.012087163
ENSG00000263264		1.060693793	0.01213979
ENSG00000171425	ZNF581	-0.632109021	0.012171032
ENSG00000230797	YY2	-1.246828083	0.01221308
ENSG00000139697	SBNO1	0.418215004	0.01221308
ENSG00000250479	CHCHD10	-0.720388657	0.01221308
ENSG00000163156	SCNM1	-0.455904748	0.012274706
ENSG00000115760	BIRC6	0.574664816	0.012274706
ENSG00000144034	TPRKB	-0.555336328	0.012333286
ENSG00000238035		1.143984139	0.012333286
ENSG00000085662	AKR1B1	-0.608501557	0.012333286
ENSG00000211897	IGHG3	-3.059172484	0.012418681
ENSG00000141873	SLC39A3	-0.676751369	0.01242397
ENSG00000232112	TMA7	-0.425568338	0.01255484
ENSG00000234585	CCT6P3	-0.661809283	0.01255484
ENSG00000127540	UQCR11	-0.535392033	0.012592905
ENSG00000084093	REST	0.542868348	0.012616576
ENSG00000136929	HEMGN	-3.521369393	0.012616576

ENSG00000119650	IFT43	-0.679602136	0.012616576
ENSG00000128908	INO80	0.417690386	0.012616576
ENSG00000105364	MRPL4	-0.518232258	0.012616576
ENSG00000164091	WDR82	0.501613749	0.01275049
ENSG00000240509	RPL34P18	-1.100601614	0.01275049
ENSG00000226121		1.420788405	0.012753751
ENSG00000100348	TXN2	-0.519550688	0.012754773
ENSG00000088356	PDRG1	-0.692255991	0.012794362
ENSG00000063180	CA11	-0.913260126	0.012794362
ENSG00000204348	DXO	-0.704852622	0.012863791
ENSG00000239569	KMT2E-AS1	-0.780131937	0.012866918
ENSG00000183542	KLRC4	-3.514610921	0.012866918
ENSG00000113194	FAF2	0.508316541	0.012888579
ENSG00000055118	KCNH2	-3.506980657	0.012888579
ENSG00000228434		-0.926355879	0.01289901
ENSG00000162227	TAF6L	-0.634185948	0.012905998
ENSG00000079462	PAFAH1B3	-0.735947342	0.012905998
ENSG00000143353	LYPLAL1	-0.78320972	0.012963649
ENSG00000164897	TMUB1	-0.510471368	0.012994771
ENSG00000160446	ZDHHC12	-0.543488114	0.013011998
ENSG00000142303	ADAMTS10	-0.99278428	0.013011998
ENSG00000258352		-2.31256717	0.013380413
ENSG00000211669	IGLV3-10	-3.776343772	0.013380413
ENSG00000230074		-0.975183916	0.013450949
ENSG00000189227	C15orf61	-0.703454096	0.013483123
ENSG00000119705	SLIRP	-0.612477138	0.013497508
ENSG00000181754	AMIGO1	-1.557921234	0.013821976
ENSG00000205531	NAP1L4	-0.475105962	0.013911691
ENSG00000225200		-0.851500695	0.014016709
ENSG00000143575	HAX1	-0.501677597	0.01401709
ENSG00000175463	TBC1D10C	-0.598721484	0.01401709
ENSG00000253701		-3.479250159	0.01401709
ENSG00000104885	DOT1L	0.98944776	0.01401709
ENSG00000105185	PDCD5	-0.627200691	0.01401709
ENSG00000268516		-1.195863546	0.01401709
ENSG00000115548	KDM3A	0.857988301	0.014046053
ENSG00000132432	SEC61G	-0.630003682	0.014065801
ENSG00000259820		1.33531448	0.014065801
ENSG00000254402	LRRC24	-1.095698885	0.014065801
ENSG00000182957	SPATA13	0.708399388	0.014065801
ENSG00000265401		-2.260507505	0.014065801
ENSG00000105677	TMEM147	-0.551182542	0.014065801
ENSG00000135930	EIF4E2	-0.369012552	0.014080576
ENSG00000151881	TMEM267	-0.693398605	0.014147513
ENSG00000186184	POLR1D	-0.412131316	0.014221566
ENSG00000089163	SIRT4	-2.057745926	0.014309859
ENSG00000164406	LEAP2	-1.205092658	0.014399458
ENSG00000155640	NA	1.146410748	0.01449837

ENSG00000137764	MAP2K5	-0.501140706	0.014583377
ENSG00000204220	PFDN6	-0.783255514	0.014588639
ENSG00000131242	RAB11FIP4	0.656101092	0.014613544
ENSG00000108826	MRPL27	-0.80556646	0.014695844
ENSG00000183978	COA3	-0.974422621	0.014733691
ENSG00000138698	RAP1GDS1	-0.516958978	0.014770098
ENSG00000251259		-0.75282835	0.014797644
ENSG00000273338		-1.216803237	0.014867394
ENSG00000156467	UQCRB	-0.484809458	0.014871945
ENSG00000279605		-1.620703984	0.014871945
ENSG00000133065	SLC41A1	-0.638509472	0.014947076
ENSG00000181038	METTL23	-0.465127571	0.014947076
ENSG00000175575	PAAF1	-0.698798243	0.015137728
ENSG00000169964	TMEM42	-0.837805388	0.015166065
ENSG00000126012	KDM5C	0.459150422	0.015171416
ENSG00000267121		-0.888009605	0.015244267
ENSG00000149743	TRPT1	-0.671511952	0.015251061
ENSG00000087589	CASS4	1.291610171	0.015308293
ENSG00000272092		-1.212907017	0.015329451
ENSG00000272398	CD24	-2.290591013	0.0153805
ENSG00000163975	MELTF	2.767519441	0.015386305
ENSG00000113161	HMGCR	0.589442305	0.015439855
ENSG00000127554	GFER	-0.50603521	0.015439855
ENSG00000115274	INO80B	-0.791604845	0.015450086
ENSG00000166199	ALKBH3	-0.580863458	0.015540239
ENSG00000141040	ZNF287	-1.32951221	0.015554176
ENSG00000168028	RPSA	-0.489559504	0.015563532
ENSG00000211710	TRBV4-1	-3.063068168	0.015563532
ENSG00000181924	COA4	-0.638982455	0.01569311
ENSG00000280234		1.507619243	0.01569311
ENSG00000143314	MRPL24	-0.83250627	0.015760222
ENSG00000168672	FAM84B	-1.379975765	0.015760222
ENSG00000108510	MED13	0.546937903	0.015839186
ENSG00000178209	PLEC	0.733447941	0.015867444
ENSG00000107020	PLGRKT	-0.670750168	0.015919642
ENSG00000083720	OXCT1	-0.737619309	0.015928244
ENSG00000127125	PPCS	-0.514972908	0.016172749
ENSG00000116586	LAMTOR2	-0.797776193	0.016180097
ENSG00000089902	RCOR1	0.456121086	0.016234438
ENSG00000137094	DNAJB5	1.598453551	0.016268665
ENSG00000169189	NSMCE1	-0.740969212	0.016295056
ENSG00000187837	HIST1H1C	-1.033703419	0.016298479
ENSG00000010278	CD9	2.722071032	0.016312303
ENSG00000124486	USP9X	0.639318343	0.016378231
ENSG00000076944	STXBP2	-0.466986659	0.016440125
ENSG00000080573	COL5A3	-3.286877814	0.016492319
ENSG00000158417	EIF5B	-0.561929254	0.016548667
ENSG00000272936		-1.204234034	0.016548667

ENSG00000162461	SLC25A34	1.441304093	0.016719869
ENSG00000008324	SS18L2	-0.557987557	0.016719869
ENSG00000174238	PITPNA	0.662681943	0.016719869
ENSG00000167491	GATAD2A	0.454136142	0.016719869
ENSG00000147576	ADHFE1	-0.944342615	0.016725311
ENSG00000182208	MOB2	-0.483077133	0.016725877
ENSG00000260400		-1.851112734	0.016725877
ENSG00000177455	CD19	-1.56320814	0.016725877
ENSG00000176022	B3GALT6	-0.817116125	0.016749951
ENSG00000166428	PLD4	-1.624997893	0.016749951
ENSG00000163820	FYCO1	0.602913102	0.016838712
ENSG00000084207	GSTP1	-0.719923973	0.016838712
ENSG00000081692	JMJD4	-0.710916057	0.016859408
ENSG00000202198		-1.53550549	0.017038046
ENSG00000151690	MFSD6	0.663052075	0.017083799
ENSG00000162384	C1orf123	-0.583146387	0.017090057
ENSG00000267751		-1.126347789	0.017090057
ENSG00000163520	FBLN2	-1.798052601	0.017108446
ENSG00000167113	COQ4	-0.639611138	0.017197666
ENSG00000167930	FAM234A	-0.527185565	0.017197666
ENSG00000127445	PIN1	-0.432245487	0.017199534
ENSG00000214140	PRCD	-3.565630981	0.017206811
ENSG00000188933	USP32P1	2.68321166	0.017217238
ENSG00000211450	SELENOH	-0.656786736	0.017281296
ENSG00000167543	TP53I13	-0.668919347	0.017281296
ENSG00000005448	WDR54	-0.799870239	0.017356757
ENSG00000101126	ADNP	0.467245568	0.017356757
ENSG00000130255	RPL36	-0.590408855	0.017356757
ENSG00000167522	ANKRD11	0.42398405	0.017462204
ENSG00000081052	COL4A4	-3.311201244	0.017606123
ENSG00000211949	IGHV3-23	-2.685467977	0.017606123
ENSG00000156795	WDYHV1	-0.712728747	0.01774331
ENSG00000226777	FAM30A	-2.206740334	0.01774331
ENSG00000172543	CTSW	-1.576645984	0.017766059
ENSG00000134070	IRAK2	3.229985846	0.017776281
ENSG00000139343	SNRPF	-0.486220851	0.017776281
ENSG00000167685	ZNF444	-0.608755174	0.017776281
ENSG00000140511	HAPLN3	-1.313810827	0.017930992
ENSG00000260807		-2.312167126	0.017930992
ENSG00000101473	ACOT8	-0.5547786	0.017939198
ENSG00000198695	MT-ND6	-0.804090324	0.017939198
ENSG00000144591	GMPPA	-0.626791367	0.017964909
ENSG00000211651	IGLV1-44	-2.70687554	0.017996276
ENSG00000099381	SETD1A	0.584340349	0.018003584
ENSG00000166289	PLEKHF1	-1.778150216	0.018069848
ENSG00000197386	HTT	0.552496367	0.0181707
ENSG00000212694	LINC01089	-0.87676789	0.0181707
ENSG00000228205		-0.966419529	0.018234874

ENSG00000229659	RPL26P6	-0.778032572	0.018234874
ENSG00000105393	BABAM1	-0.488138017	0.018234874
ENSG00000231584	FAHD2CP	-1.291835289	0.01823871
ENSG00000254709	IGLL5	-2.733281699	0.018239823
ENSG00000173575	CHD2	0.697761212	0.018277794
ENSG00000138629	UBL7	-0.552734623	0.018375108
ENSG00000114956	DGUOK	-0.403319234	0.018399221
ENSG00000141378	PTRH2	-0.685700234	0.018399221
ENSG00000170604	IRF2BP1	-0.732467824	0.018490551
ENSG00000135999	EPC2	0.537027555	0.018496839
ENSG00000241468	ATP5J2	-0.583158339	0.0185079
ENSG00000128626	MRPS12	-0.734994349	0.0185079
ENSG00000187608	ISG15	-1.46119361	0.018532403
ENSG00000237190	CDKN2AIPNL	-0.509589406	0.01855445
ENSG00000083814	ZNF671	-0.853461458	0.018564748
ENSG00000197705	KLHL14	-2.750140245	0.018631271
ENSG00000189046	ALKBH2	-0.733517608	0.018739732
ENSG00000109475	RPL34	-0.523209863	0.018782154
ENSG00000134278	SPIRE1	1.74525424	0.018782154
ENSG00000119682	AREL1	0.461581929	0.01879741
ENSG00000211785	TRAV12-1	-3.101811298	0.018882497
ENSG00000269044		-0.738577114	0.018882497
ENSG00000214176	PLEKHM1P1	0.561640744	0.018926246
ENSG00000143222	UFC1	-0.36644964	0.018926496
ENSG00000226660	TRBV2	-2.870183762	0.018926496
ENSG00000182985	CADM1	4.984035986	0.018926496
ENSG00000272333	KMT2B	0.445024101	0.018926496
ENSG00000185608	MRPL40	-0.613151071	0.018926496
ENSG00000115464	USP34	0.425242877	0.01897551
ENSG00000267074		-2.544539019	0.01897551
ENSG00000105855	ITGB8	3.226866868	0.019044746
ENSG00000168036	CTNNB1	0.699145409	0.019070822
ENSG00000164182	NDUFAF2	-0.671904715	0.019070822
ENSG00000088833	NSFL1C	-0.513270334	0.019070822
ENSG00000215375	MYL5	-0.902588712	0.019100198
ENSG00000063438	AHRR	4.138876321	0.01912396
ENSG00000110871	COQ5	-0.740620258	0.01919413
ENSG00000205155	PSENEN	-0.540505486	0.01919413
ENSG00000123472	ATPAF1	-0.600531509	0.019213778
ENSG00000172939	OXSR1	0.928779097	0.019213778
ENSG00000078589	P2RY10	-1.516465551	0.019213778
ENSG00000110011	DNAJC4	-0.430690776	0.019213778
ENSG00000257698		-1.122545736	0.019213778
ENSG00000100442	FKBP3	-0.70410967	0.019213778
ENSG00000179044	EXOC3L1	-1.564527187	0.019213778
ENSG00000126267	COX6B1	-0.496403471	0.019213778
ENSG00000269968		-1.880810938	0.019223317
ENSG00000166747	AP1G1	0.764752289	0.019223317

ENSG00000271601	LIX1L	-0.587030616	0.019245139
ENSG00000169955	ZNF747	-0.58272777	0.019247213
ENSG00000272156		-2.24750863	0.019318046
ENSG00000145675	PIK3R1	1.141154294	0.019404171
ENSG00000167088	SNRPD1	-0.590306922	0.019404171
ENSG00000144741	SLC25A26	-0.508524868	0.019408097
ENSG00000153006	SREK1IP1	-0.452129897	0.019408097
ENSG00000230551		0.931141552	0.019408097
ENSG00000072786	STK10	0.431289025	0.019408097
ENSG00000159199	ATP5G1	-0.583927881	0.019408097
ENSG00000183751	TBL3	-0.442827112	0.019597081
ENSG00000120699	EXOSC8	-0.523296597	0.019599898
ENSG00000136158	SPRY2	3.763750349	0.019640602
ENSG00000184371	CSF1	2.794324569	0.019641343
ENSG00000014641	MDH1	-0.567221626	0.019641343
ENSG00000186577	C6orf1	-0.497074092	0.019641343
ENSG00000182774	RPS17	-0.467072548	0.019641343
ENSG00000181555	SETD2	0.456207411	0.019654511
ENSG00000260912		-1.257022729	0.019654511
ENSG00000167526	RPL13	-0.558412207	0.019654511
ENSG00000264608		-0.851528187	0.019654511
ENSG00000232388	LINC00493	-0.498133742	0.019654511
ENSG00000176533	GNG7	-0.948468332	0.019654511
ENSG00000079432	CIC	0.526654341	0.019654511
ENSG00000172071	EIF2AK3	1.236870324	0.019677201
ENSG00000162032	SPSB3	-0.452726034	0.019677201
ENSG00000141294	LRRC46	-1.182141108	0.019677201
ENSG00000152102	FAM168B	0.453109026	0.019756778
ENSG00000141933	TPGS1	-0.758279532	0.019756778
ENSG00000144674	GOLGA4	0.641678	0.019840151
ENSG00000140526	ABHD2	0.738472671	0.019840151
ENSG00000111879	FAM184A	-2.878102781	0.019870515
ENSG00000143947	RPS27A	-0.477264582	0.019974425
ENSG00000175634	RPS6KB2	-0.39429141	0.019995216
ENSG00000132436	FIGNL1	-1.187557042	0.020011769
ENSG00000133256	PDE6B	-0.745521031	0.020048245
ENSG00000186073	C15orf41	-1.060874064	0.020048245
ENSG00000157540	DYRK1A	0.394608175	0.020048245
ENSG00000178015	GPR150	-2.899968292	0.020186734
ENSG00000204356	NELFE	-0.378936214	0.020186734
ENSG00000182180	MRPS16	-0.515018401	0.020218243
ENSG00000011566	MAP4K3	1.174054156	0.020336903
ENSG00000183828	NUDT14	-0.776059832	0.020336903
ENSG00000228444		-1.852320423	0.020437818
ENSG00000139289	PHLDA1	3.023396358	0.020560825
ENSG00000266302		3.306370388	0.020560825
ENSG00000153201	RANBP2	1.120313459	0.020578159
ENSG00000237753		-1.217946732	0.020578159

ENSG00000145354	CISD2	-0.680419254	0.020578159
ENSG00000116688	MFN2	0.451240508	0.020653781
ENSG00000162191	UBXN1	-0.430653488	0.020653781
ENSG00000267598		-1.552476277	0.020653781
ENSG00000204536	CCHCR1	-0.692154496	0.020659197
ENSG00000058063	ATP11B	0.5765797	0.020860331
ENSG00000100416	TRMU	-0.503297854	0.020905118
ENSG00000060140	STYK1	-3.481975038	0.021009306
ENSG00000112078	KCTD20	0.482488032	0.021072367
ENSG00000147804	SLC39A4	-0.77013501	0.021137455
ENSG00000227678		-2.882857908	0.02114369
ENSG00000064393	HIPK2	1.005115978	0.021228159
ENSG00000147687	TATDN1	-0.567515132	0.021248518
ENSG00000115211	EIF2B4	-0.441372971	0.021272985
ENSG00000136295	TTYH3	0.855994704	0.021272985
ENSG00000272155		-1.373165187	0.021272985
ENSG00000165905	LARGE2	-2.444323027	0.021272985
ENSG00000183741	CBX6	0.67828884	0.02127616
ENSG00000272990		-1.166285837	0.0214968
ENSG00000163564	PYHIN1	-1.545648957	0.021593723
ENSG00000124562	SNRPC	-0.491495509	0.021593723
ENSG00000149150	SLC43A1	-0.919327556	0.021593723
ENSG00000179639	FCER1A	-2.346231754	0.021600619
ENSG00000223797	ENTPD3-AS1	-0.931001236	0.021600619
ENSG00000251580		-1.061409631	0.021600619
ENSG00000111196	MAGOHB	-0.647828677	0.021600619
ENSG00000169228	RAB24	-0.794945772	0.021640774
ENSG00000174292	TNK1	-2.337306501	0.021696075
ENSG00000164211	STARD4	1.08989202	0.021754687
ENSG00000152672	CLEC4F	-3.679629777	0.021793424
ENSG00000258704	SRP54-AS1	-1.029317941	0.021793424
ENSG00000262089		-0.744351419	0.021851801
ENSG00000178896	EXOSC4	-0.914135302	0.021867556
ENSG00000175352	NRIP3	3.048087477	0.021867556
ENSG00000225190	PLEKHM1	0.473557832	0.021867556
ENSG00000248015		-1.798970906	0.021867556
ENSG00000104886	PLEKHJ1	-0.643088924	0.021867556
ENSG00000169567	HINT1	-0.367376398	0.021974635
ENSG00000166435	XRRA1	1.937104971	0.022002878
ENSG00000174276	ZNHIT2	-0.982702503	0.022014493
ENSG00000148300	REXO4	-0.555414562	0.02201515
ENSG00000137449	CPEB2	0.864311924	0.022065141
ENSG00000104529	EEF1D	-0.392033023	0.022065141
ENSG00000103707	MTFMT	-0.867908231	0.022065141
ENSG00000174915	PTDSS2	-0.434865214	0.022115256
ENSG00000101464	PIGU	-0.780536522	0.022119203
ENSG00000080603	SRCAP	0.732573402	0.02217788
ENSG00000127481	UBR4	0.613054729	0.022203271

ENSG00000005075	POLR2J	-0.477421522	0.022203271
ENSG00000125846	ZNF133	-0.827304101	0.022203271
ENSG00000080189	SLC35C2	-0.364600267	0.022203271
ENSG00000105617	LENG1	-0.722161335	0.022203271
ENSG00000115350	POLE4	-0.524230201	0.022241406
ENSG00000168386	FILIP1L	1.962897175	0.022241406
ENSG00000196367	TRRAP	0.534933607	0.022268476
ENSG00000257764		-1.082228226	0.022345117
ENSG00000260711		-2.0245142	0.022345117
ENSG00000161956	SENP3	-0.557711345	0.022345117
ENSG00000180739	S1PR5	-2.739359395	0.022407855
ENSG00000172943	PHF8	0.36111685	0.022451374
ENSG00000112306	RPS12	-0.502564872	0.022526066
ENSG00000135018	UBQLN1	0.363803742	0.022526066
ENSG00000116918	TSNAX	-0.651962658	0.022543549
ENSG00000242588		1.178892176	0.022598144
ENSG00000279491		-1.167603432	0.022653507
ENSG00000170776	AKAP13	0.514985147	0.022653507
ENSG00000160113	NR2F6	-1.177526509	0.022653507
ENSG00000138495	COX17	-0.619447435	0.022731675
ENSG00000115594	IL1R1	2.939034403	0.022845218
ENSG00000104979	C19orf53	-0.423029129	0.0228483
ENSG00000174165	ZDHHC24	-0.502229558	0.023105361
ENSG00000198934	MAGEE1	-2.384939115	0.023117824
ENSG00000174437	ATP2A2	0.506316562	0.023120664
ENSG00000171163	ZNF692	-0.921576367	0.023154541
ENSG00000114902	SPCS1	-0.491996822	0.023157331
ENSG00000249592		-0.910765919	0.023157331
ENSG00000166886	NAB2	1.97677917	0.023286188
ENSG00000050327	ARHGEF5	-2.373990214	0.023341233
ENSG00000123908	AGO2	0.664061762	0.023341233
ENSG00000197818	SLC9A8	0.72859215	0.023341233
ENSG00000099800	TIMM13	-0.720533109	0.023341233
ENSG00000172508	CARNS1	-1.485707396	0.023400022
ENSG00000116539	ASH1L	0.547707622	0.023450197
ENSG00000103152	MPG	-0.535601238	0.023464566
ENSG00000107874	CUEDC2	-0.383210917	0.023576584
ENSG00000099377	HSD3B7	1.513181453	0.023798187
ENSG00000095059	DHPS	-0.602556942	0.023798187
ENSG00000162585	FAAP20	-0.468043532	0.023835383
ENSG00000198917	SPOUT1	-0.587528877	0.024111185
ENSG00000160050	CCDC28B	-0.971277019	0.024122808
ENSG00000116521	SCAMP3	-0.459754925	0.024207964
ENSG00000211598	IGKV4-1	-2.813064947	0.024207964
ENSG00000206560	ANKRD28	1.283861498	0.024207964
ENSG00000167272	POP5	-0.854945603	0.02432929
ENSG00000178974	FBXO34	0.617300566	0.024390219
ENSG00000173848	NET1	1.695556157	0.024456599

ENSG00000165516	KLHDC2	-0.485259048	0.024456599
ENSG00000104964	AES	-0.433751564	0.024456599
ENSG00000186222	BLOC1S4	-0.559900092	0.024470398
ENSG00000242686		-2.162350471	0.024487354
ENSG00000146072	TNFRSF21	3.710797389	0.024499016
ENSG00000101187	SLCO4A1	3.982247529	0.024499016
ENSG00000005436	GCFC2	-0.572646452	0.024565687
ENSG00000170860	LSM3	-0.489732634	0.024565687
ENSG00000281206	NA	-3.368165918	0.024565687
ENSG00000099849	RASSF7	-0.972206305	0.024595753
ENSG00000165813	CCDC186	0.663766732	0.024687163
ENSG00000221792	MIR1282	-2.544690167	0.024687163
ENSG00000041988	THAP3	-0.775738062	0.024738204
ENSG00000183513	COA5	-0.681995003	0.024769634
ENSG00000078269	SYNJ2	1.124244178	0.024769634
ENSG00000178429	RPS3AP5	-0.591234328	0.024769634
ENSG00000106733	NMRK1	-0.915429515	0.024808254
ENSG00000161618	ALDH16A1	-0.570966986	0.024808254
ENSG00000109927	TECTA	1.621176029	0.024825641
ENSG00000225921	NOL7	-0.357614315	0.024887423
ENSG00000125356	NDUFA1	-0.429324296	0.024903547
ENSG00000134109	EDEM1	0.588197648	0.0249238
ENSG00000272047	GTF2H5	-0.603290404	0.0249238
ENSG00000158716	DUSP23	-0.766487048	0.024952719
ENSG00000214293	APTR	-0.807379196	0.024957679
ENSG00000004779	NDUFAB1	-0.604613464	0.025040438
ENSG00000279800	BCLAF1P2	1.289804498	0.025040438
ENSG00000126934	MAP2K2	-0.457215538	0.025040438
ENSG00000173120	KDM2A	0.504028514	0.025124885
ENSG00000107862	GBF1	0.372153055	0.025348284
ENSG00000215421	ZNF407	0.473216201	0.025348284
ENSG00000280387		1.18402471	0.025378752
ENSG00000171621	SPSB1	2.114236491	0.025381006
ENSG00000164190	NIPBL	0.44846775	0.025381006
ENSG00000037749	MFAP3	0.805727863	0.025381006
ENSG00000206344	HCG27	-1.285257158	0.025381006
ENSG00000136936	XPA	-0.472543218	0.025381006
ENSG00000148450	MSRB2	-0.767262355	0.025381006
ENSG00000042088	TDP1	-0.431474674	0.025381006
ENSG00000246067	RAB30-AS1	-0.915751057	0.025399413
ENSG00000099331	MYO9B	0.482696177	0.025399413
ENSG00000176261	ZBTB8OS	-0.490437415	0.025431111
ENSG00000137496	IL18BP	0.880616844	0.025528674
ENSG00000134531	EMP1	2.55052526	0.025528674
ENSG00000141699	FAM134C	-0.673472558	0.025565125
ENSG00000173660	UQCRH	-0.384529578	0.025588813
ENSG00000213015	ZNF580	-0.601527207	0.025663166
ENSG00000167969	ECI1	-0.600681358	0.025743214

ENSG00000056972	TRAF3IP2	0.897113637	0.026002178
ENSG00000235065	RPL24P2	-0.851508615	0.02603448
ENSG00000104884	ERCC2	-0.624171592	0.026050533
ENSG00000166716	ZNF592	0.438000546	0.026062079
ENSG00000206417	H1FX-AS1	-1.010726089	0.026078839
ENSG00000163964	PIGX	-0.576009217	0.026078839
ENSG00000169902	TPST1	2.392070706	0.026078839
ENSG00000139546	TARBP2	-0.583239623	0.026078839
ENSG00000100288	CHKB	-0.584222445	0.026078839
ENSG00000171316	CHD7	1.073733163	0.026081784
ENSG00000267469		-2.554997863	0.026131442
ENSG00000077312	SNRPA	-0.558935214	0.026131442
ENSG00000184162	NR2C2AP	-0.726067336	0.02623281
ENSG00000136280	CCM2	-0.648850426	0.026343438
ENSG00000165113	GKAP1	-0.88964509	0.026344776
ENSG00000244625	MIATNB	-0.690042785	0.026501044
ENSG00000184613	NELL2	-1.740079443	0.026825109
ENSG00000138031	ADCY3	1.297865892	0.02690032
ENSG00000130520	LSM4	-0.506509294	0.027006809
ENSG00000277972	CISD3	-0.680067855	0.027043623
ENSG00000184508	HDDC3	-0.77332777	0.027124592
ENSG00000054267	ARID4B	0.421114391	0.02719172
ENSG00000075539	FRYL	0.475720877	0.02719172
ENSG00000272221		-1.528425184	0.02719172
ENSG00000130724	CHMP2A	-0.501909261	0.02719172
ENSG00000180098	TRNAU1AP	-0.49471064	0.027333643
ENSG00000204954	C12orf73	-0.952217125	0.027333643
ENSG00000122971	ACADS	-0.619653718	0.027333643
ENSG00000139597	N4BP2L1	-0.554044707	0.027333643
ENSG00000182512	GLRX5	-0.54314681	0.027405529
ENSG00000134262	AP4B1	-0.44425215	0.027669504
ENSG00000173992	CCS	-0.701176639	0.027669504
ENSG00000143373	ZNF687	0.479829483	0.027690243
ENSG00000152894	PTPRK	-1.937761807	0.027690243
ENSG00000176641	RNF152	3.483770873	0.027718994
ENSG00000114982	KANSL3	0.440411366	0.02776254
ENSG00000172348	RCAN2	-3.731697861	0.02779231
ENSG00000112667	DNPH1	-0.789091427	0.027850794
ENSG00000114841	DNAH1	0.534116579	0.027866299
ENSG00000164292	RHOBTB3	2.007126965	0.027866299
ENSG00000146067	FAM193B	-0.456480654	0.027866299
ENSG00000256338	RPL41P2	-1.007196144	0.027866299
ENSG00000141503	MINK1	0.498882757	0.027866299
ENSG00000125995	ROMO1	-0.653782255	0.027866299
ENSG00000105373	GLTSCR2	-0.508819392	0.027866299
ENSG00000162244	RPL29	-0.461104731	0.027897926
ENSG00000055208	TAB2	0.714201364	0.027922011
ENSG00000154723	ATP5J	-0.469016379	0.028012532

ENSG00000224094	RPS24P8	-0.613556698	0.028034635
ENSG00000176058	TPRN	-0.678657317	0.0280373
ENSG00000136444	RSAD1	-0.636304619	0.0280373
ENSG00000243466	IGKV1-5	-2.747646761	0.028041033
ENSG00000238045		-0.987291268	0.028041033
ENSG00000172534	HCFC1	0.491773922	0.028087681
ENSG00000132912	DCTN4	0.651916738	0.028094624
ENSG00000177600	RPLP2	-0.556323162	0.028094624
ENSG00000276819	TRBV15	-3.14436854	0.028126446
ENSG00000149922	TBX6	-0.932450331	0.028236084
ENSG00000198830	HMGN2	-0.473773275	0.028322698
ENSG00000198816	ZNF358	-0.737212475	0.028790984
ENSG00000132196	HSD17B7	-0.828725168	0.028858721
ENSG00000114383	TUSC2	-0.42975637	0.028864099
ENSG00000279648		1.261521369	0.028950354
ENSG00000211767	TRBJ2-3	-3.138841709	0.029129037
ENSG00000172296	SPTLC3	1.518676567	0.029177577
ENSG00000170242	USP47	0.524550822	0.029279897
ENSG00000135905	DOCK10	0.526719754	0.029487881
ENSG00000160948	VPS28	-0.453991722	0.029487881
ENSG00000107263	RAPGEF1	0.794697107	0.029487881
ENSG00000120647	CCDC77	-0.642454516	0.029487881
ENSG00000257303		-1.147572618	0.029487881
ENSG00000130052	STARD8	1.440779885	0.029531387
ENSG00000111641	NOP2	0.626715697	0.029693735
ENSG00000160131	VMA21	-0.412572078	0.029721222
ENSG00000231672	DIRC3	-1.669331958	0.029803167
ENSG00000205089	CCNI2	-3.132667856	0.029803167
ENSG00000164649	CDCA7L	-0.838360063	0.029861994
ENSG00000183617	MRPL54	-0.584433109	0.029921019
ENSG00000213079	SCAF8	0.568275652	0.029952713
ENSG00000211806	TRAV25	-3.150083487	0.029952713
ENSG00000245970		-1.424898207	0.029957007
ENSG00000124507	PACSIN1	-2.900353487	0.029967501
ENSG00000137970	RPL7P9	-0.468088405	0.030003886
ENSG00000168393	DTYMK	-0.809829934	0.030003886
ENSG00000101997	CCDC22	-0.528870618	0.030003886
ENSG00000272010		-1.909548289	0.030003886
ENSG00000110046	ATG2A	1.03334571	0.030003886
ENSG00000127884	ECHS1	-0.594112929	0.030003886
ENSG00000156535	CD109	2.355823865	0.030020281
ENSG00000105619	TFPT	-0.567208273	0.030052758
ENSG00000091732	ZC3HC1	-0.523477153	0.030117626
ENSG00000162636	FAM102B	0.809820526	0.030119011
ENSG00000163001	CFAP36	-0.658800152	0.030119011
ENSG00000170476	MZB1	-2.134287917	0.030119011
ENSG00000186432	KPNA4	0.449671953	0.030205799
ENSG00000173114	LRRN3	-1.945331465	0.030205799

ENSG00000196878	LAMB3	4.640058503	0.030307255
ENSG00000183207	RUVBL2	-0.693767535	0.030307255
ENSG00000184436	THAP7	-0.527344163	0.030408947
ENSG00000148334	PTGES2	-0.417092349	0.030449131
ENSG00000174721	FGFBP3	-0.89856109	0.030449131
ENSG00000103363	TCEB2	-0.511962579	0.030555376
ENSG00000187051	RPS19BP1	-0.483204817	0.030555376
ENSG00000243317	C7orf73	-0.356230575	0.030646771
ENSG00000137700	SLC37A4	-0.767660534	0.030768163
ENSG00000105655	ISYNA1	1.129457882	0.030924052
ENSG00000092203	TOX4	0.362644096	0.030980072
ENSG00000095564	BTAF1	0.614609346	0.031170324
ENSG00000125633	CCDC93	0.497019736	0.031231138
ENSG00000247828	TMEM161B-AS1	-0.906054292	0.031231138
ENSG00000105808	RASA4	-0.792830397	0.031231138
ENSG00000239697	TNFSF12	-0.602025011	0.031231138
ENSG00000164967	RPP25L	-0.945716033	0.031241588
ENSG00000211765	TRBJ2-2	-3.128743004	0.031268971
ENSG00000273437		-1.480941907	0.031323549
ENSG00000256690		-1.35061744	0.031323549
ENSG00000241370	RPP21	-0.686097162	0.031344836
ENSG00000135423	GLS2	-3.134035196	0.031408719
ENSG00000054654	SYNE2	-0.892314379	0.031408719
ENSG00000162066	AMDHD2	-0.561184696	0.031408719
ENSG00000211890	IGHA2	-3.043639092	0.031431437
ENSG00000167136	ENDOG	-0.988204244	0.031565691
ENSG00000159445	THEM4	-0.917400374	0.031650323
ENSG00000213609	RPL7AP50	-0.936589701	0.03166195
ENSG00000106346	USP42	0.699110953	0.03195095
ENSG00000198406	BZW1P2	1.057910367	0.031954257
ENSG00000272556	GTF2IP13	-0.919675828	0.031983814
ENSG00000104983	CCDC61	-0.810447964	0.031983814
ENSG00000036054	TBC1D23	0.661130285	0.031985522
ENSG00000196976	LAGE3	-0.81100185	0.031985522
ENSG00000226049	TLK2P1	1.406651258	0.031985522
ENSG00000164167	LSM6	-0.504758442	0.032074462
ENSG00000137133	HINT2	-0.631687853	0.032162283
ENSG00000132254	ARFIP2	-0.373804473	0.032187691
ENSG00000228889	UBAC2-AS1	-1.064954549	0.03229863
ENSG00000227766	HCG4P5	-1.819950371	0.032445553
ENSG00000086102	NFX1	0.665089384	0.032445553
ENSG00000115020	PIKFYVE	0.514275688	0.032463413
ENSG00000234506	LINC01506	-1.091454761	0.032561016
ENSG00000274425		-1.245238567	0.032561016
ENSG00000213689	TREX1	-0.868830453	0.032639315
ENSG00000004059	ARF5	-0.334508584	0.032658839
ENSG00000172985	SH3RF3	2.470593277	0.032675987
ENSG00000171858	RPS21	-0.520884107	0.032675987

ENSG00000130725	UBE2M	-0.419222937	0.032675987
ENSG00000272886	DCP1A	0.664263129	0.032713515
ENSG00000233830	EIF4HP1	0.855207374	0.032721838
ENSG00000115307	AUP1	-0.261371295	0.032800944
ENSG00000072818	ACAP1	-0.580695267	0.032800944
ENSG00000197417	SHPK	-0.817123117	0.033029423
ENSG00000131263	RLIM	0.802493937	0.033050614
ENSG00000187605	TET3	0.563025251	0.033157234
ENSG00000267547		-1.151716539	0.033274209
ENSG00000065717	TLE2	-1.826407142	0.033274209
ENSG00000237976		-1.087958933	0.033356063
ENSG00000257433		-0.936390659	0.033398898
ENSG00000135441	BLOC1S1	-0.583296421	0.033398898
ENSG00000258230		1.880877523	0.033398898
ENSG00000230124	ACBD6	-0.395426883	0.033433735
ENSG00000063241	ISOC2	-0.91618381	0.033433735
ENSG00000136715	SAP130	0.385084825	0.033508369
ENSG00000255760		-1.486432153	0.033526628
ENSG00000270091		-0.997941738	0.033526628
ENSG00000198680	TUSC1	-1.48592915	0.033540244
ENSG00000176928	GCNT4	-1.706038951	0.033542851
ENSG00000113615	SEC24A	0.699303865	0.033542851
ENSG00000276997		0.997353262	0.033575959
ENSG00000089009	RPL6	-0.362646871	0.033575959
ENSG00000233762		-0.663632072	0.033650837
ENSG00000243477	NAT6	-0.534245084	0.033798149
ENSG00000164530	PI16	-2.767710551	0.033798149
ENSG00000267216		1.599049828	0.033798149
ENSG00000163516	ANKZF1	-0.770054168	0.033980388
ENSG00000198498	TMA16	-0.85210163	0.033980388
ENSG00000165219	GAPVD1	0.449676267	0.033980388
ENSG00000261996		-3.133718706	0.033980388
ENSG00000205078	SYCE1L	-1.021721736	0.033991493
ENSG00000162496	DHRS3	4.235173009	0.034011857
ENSG00000134193	REG4	-3.134612984	0.034086813
ENSG00000229833	PET100	-0.583040601	0.03414091
ENSG00000171530	TBCA	-0.519696424	0.034205608
ENSG00000277496		-1.080679107	0.034217811
ENSG00000132465	JCHAIN	-2.282890009	0.034291752
ENSG00000121753	ADGRB2	-3.13423177	0.034323713
ENSG00000237765	FAM200B	-0.56472199	0.034323713
ENSG00000236810	TCEB3-AS1	-0.930230335	0.034447011
ENSG00000167680	SEMA6B	4.630329697	0.034447011
ENSG00000159082	SYNJ1	0.606780167	0.034478766
ENSG00000142684	ZNF593	-0.692685113	0.034509516
ENSG00000225873	LINC00694	-0.979060396	0.034509516
ENSG00000064313	TAF2	0.45085625	0.034509516
ENSG00000245205	EEF1A1P4	2.082654308	0.034509516

ENSG00000105202	FBL	-0.358253286	0.034509516
ENSG00000110200	ANAPC15	-0.412695928	0.034543209
ENSG00000116786	PLEKHM2	0.687089511	0.034720043
ENSG00000163344	PMVK	-0.849333057	0.034720043
ENSG00000002587	HS3ST1	4.214811479	0.034720043
ENSG00000118418	HMGN3	-0.558436062	0.034720043
ENSG00000198467	TPM2	-0.881114274	0.034720043
ENSG00000133321	RARRES3	-0.64986815	0.034720043
ENSG00000277662		-1.299843575	0.034720043
ENSG00000170175	CHRNB1	-0.828973863	0.034720043
ENSG00000204584		-0.871994181	0.034720043
ENSG00000144711	IQSEC1	0.576502418	0.034744985
ENSG00000109756	RAPGEF2	0.726793779	0.034776622
ENSG00000280734	LINC01232	-0.566448856	0.03483338
ENSG00000126264	HCST	-0.673432407	0.03483338
ENSG00000204859	ZBTB48	-0.442831744	0.034872497
ENSG00000163374	YY1AP1	0.323546435	0.034872497
ENSG00000008056	SYN1	3.570921175	0.034956775
ENSG00000104518	GSDMD	-0.77677151	0.034992233
ENSG00000186468	RPS23	-0.415367533	0.035065093
ENSG00000166233	ARIH1	0.632115774	0.035065093
ENSG00000188011	RTP5	5.027326556	0.03512535
ENSG00000170846		-0.7221086	0.035135891
ENSG00000140264	SERF2	-0.399073562	0.035135891
ENSG00000148690	FRA10AC1	-0.711114025	0.035296032
ENSG00000240972	MIF	-0.575281757	0.035296032
ENSG00000168488	ATXN2L	0.365518268	0.035431286
ENSG00000261643		-1.142226958	0.035494446
ENSG00000167700	MFSD3	-1.152338368	0.035494446
ENSG00000272750		-1.094186671	0.035509504
ENSG00000165874	FAM35BP	-0.849186131	0.035509504
ENSG00000185088	RPS27L	-0.403231651	0.035509504
ENSG00000233223		-0.984342917	0.035509504
ENSG00000167536	DHRS13	-0.787779416	0.035509504
ENSG00000101363	MANBAL	-0.44015654	0.035509504
ENSG00000169241	SLC50A1	-0.549755493	0.035522393
ENSG00000253200		1.080062197	0.035522393
ENSG00000198879	SFMBT2	1.009872758	0.035522393
ENSG00000166669	ATF7IP2	-0.587252968	0.035522393
ENSG00000140829	DHX38	0.370106386	0.035522393
ENSG00000005884	ITGA3	-1.158850528	0.035522393
ENSG00000115268	RPS15	-0.497812116	0.035522393
ENSG00000129932	DOHH	-0.655050634	0.035522393
ENSG00000105427	CNFN	-1.272617389	0.035734522
ENSG00000137502	RAB30	-1.33987915	0.035843763
ENSG00000082805	ERC1	0.529342446	0.035843763
ENSG00000075336	TIMM21	-0.622482362	0.03593511
ENSG00000100330	MTMR3	0.460353296	0.036049747

ENSG00000239246		-0.781696498	0.036090388
ENSG00000148600	CDHR1	-3.138213034	0.036102288
ENSG00000183691	NOG	-1.972410561	0.036117435
ENSG00000168060	NAALADL1	-0.723939726	0.036160311
ENSG00000102886	GDPD3	-0.986775891	0.036211636
ENSG00000050426	LETMD1	-0.482779896	0.036262099
ENSG00000160752	FDPS	-0.437762523	0.036362095
ENSG00000227057	WDR46	-0.605025324	0.036413212
ENSG00000180611	MB21D2	2.695103339	0.036440443
ENSG00000266472	MRPS21	-0.469383388	0.036549244
ENSG00000173166	RAPH1	1.555459747	0.036549244
ENSG00000160469	BRSK1	-2.008328061	0.036596343
ENSG00000244398		-0.505156221	0.036692324
ENSG00000160789	LMNA	2.260854059	0.036705214
ENSG00000161202	DVL3	0.505403401	0.036705214
ENSG00000176973	FAM89B	-0.658946552	0.036723107
ENSG00000164114	MAP9	-2.037066706	0.036748652
ENSG00000011405	PIK3C2A	0.526700347	0.036753474
ENSG00000134291	TMEM106C	-0.83027396	0.036753474
ENSG00000185187	SIGIRR	-0.555850669	0.036844175
ENSG00000176102	CSTF3	-0.521079157	0.036844175
ENSG00000186026	ZNF284	-1.06684792	0.036844175
ENSG00000170445	HARS	-0.357359517	0.037227683
ENSG00000227008		-0.639990128	0.037227683
ENSG00000250571	GLI4	-0.540572947	0.037227683
ENSG00000143622	RIT1	0.664447278	0.037230484
ENSG00000276728		-1.144631216	0.037230484
ENSG00000031698	SARS	-0.462762811	0.037233831
ENSG00000013588	GPRC5A	2.556559033	0.037233831
ENSG00000225614	ZNF469	1.661462497	0.037233831
ENSG00000173065	FAM222B	0.60139117	0.037233831
ENSG00000141446	ESCO1	0.497014238	0.037240263
ENSG00000244575	IGKV1-27	-3.104042085	0.037264327
ENSG00000104129	DNAJC17	-0.464809803	0.037321913
ENSG00000133935	C14orf1	-0.658710655	0.037491226
ENSG00000103260	METRN	-0.946916408	0.037605871
ENSG00000127774	EMC6	-0.614312464	0.037607826
ENSG00000152969	JAKMIP1	-2.882368512	0.037609403
ENSG00000167034	NKX3-1	2.341004939	0.037609403
ENSG00000277610	RNVU1-4	-1.001377715	0.037649498
ENSG00000125351	UPF3B	-0.554899265	0.037649498
ENSG00000054116	TRAPPC3	-0.386469139	0.037842764
ENSG00000182827	ACBD3	0.604497749	0.037842764
ENSG00000144161	ZC3H8	-0.581582319	0.037842764
ENSG00000118777	ABCG2	4.62118608	0.037842764
ENSG00000123219	CENPK	-1.552335559	0.037842764
ENSG00000196531	NACA	-0.324584756	0.037842764
ENSG00000263766		-1.013943188	0.037842764

ENSG00000242299		-0.600059343	0.037897903
ENSG00000104936	DMPK	-0.925328832	0.037925675
ENSG00000115286	NDUFS7	-0.41871695	0.037966589
ENSG00000225079	FTH1P22	-3.145246135	0.038144542
ENSG00000172803	SNX32	-1.691732441	0.038144542
ENSG00000168701	TMEM208	-0.586997584	0.038144542
ENSG00000015133	CCDC88C	0.518586117	0.038170442
ENSG00000112282	MED23	0.511116481	0.038177911
ENSG00000183154		0.858841273	0.038177911
ENSG00000268758	ADGRE4P	2.916243913	0.038177911
ENSG00000108175	ZMIZ1	0.997346408	0.038283131
ENSG00000264577		-2.047081656	0.038408804
ENSG00000101132	PFDN4	-0.543590927	0.038416475
ENSG00000159377	PSMB4	-0.326962868	0.038475571
ENSG00000143742	SRP9	-0.49710727	0.038475571
ENSG00000168894	RNF181	-0.569500978	0.038475571
ENSG00000152784	PRDM8	1.832420454	0.038475571
ENSG00000083845	RPS5	-0.479993169	0.038475571
ENSG00000142252	GEMIN7	-0.815185734	0.03856322
ENSG00000172992	DCAKD	-0.580232999	0.038775116
ENSG00000272256		-0.888298201	0.038849526
ENSG00000198964	SGMS1	0.618150485	0.038849526
ENSG00000075089	ACTR6	-0.554268402	0.038849526
ENSG00000099814	CEP170B	2.400863132	0.038849526
ENSG00000259972		-0.748618274	0.038849526
ENSG00000160307	S100B	-2.241887589	0.038849526
ENSG00000207652	MIR621	3.819437286	0.038891609
ENSG00000196396	PTPN1	0.628100761	0.038936863
ENSG00000143365	RORC	-2.50333728	0.038957382
ENSG00000213614	HEXA	-0.335577228	0.038985474
ENSG00000183856	IQGAP3	3.666347635	0.039159362
ENSG00000241973	PI4KA	0.371268385	0.039190661
ENSG00000090263	MRPS33	-0.603244457	0.039195073
ENSG00000143442	POGZ	0.368986727	0.039531694
ENSG00000056097	ZFR	0.368369844	0.039531694
ENSG00000176485	PLA2G16	-1.193605918	0.039531694
ENSG00000102977	ACD	-0.671711797	0.039531694
ENSG00000198918	RPL39	-0.458895957	0.039550276
ENSG00000173465	SSSCA1	-0.544505937	0.039693033
ENSG00000188211	NCR3LG1	2.352754732	0.0399304
ENSG00000102871	TRADD	-0.744357518	0.0399304
ENSG00000149600	COMMD7	-0.376624037	0.0399304
ENSG00000079616	KIF22	-0.494760231	0.04002808
ENSG00000089289	IGBP1	-0.335047747	0.04006359
ENSG00000125430	HS3ST3B1	2.154403761	0.040267757
ENSG00000176978	DPP7	-0.404499071	0.040267896
ENSG00000116288	PARK7	-0.437181375	0.040293696
ENSG00000267745		-0.8100025	0.040293696

ENSG00000066583	ISOC1	-0.627870879	0.04032593
ENSG00000125450	NUP85	-0.398775131	0.040592692
ENSG00000165644	COMTD1	-0.893950878	0.040610066
ENSG00000174788	PCP2	-1.020760564	0.040697684
ENSG00000260778	MIR940	-1.109840597	0.040734363
ENSG00000275963		-1.298079871	0.04075364
ENSG00000079313	REXO1	0.479942387	0.04075364
ENSG00000065518	NDUFB4	-0.396480005	0.040855389
ENSG00000268836		-1.393338904	0.040874666
ENSG00000125652	ALKBH7	-0.590172107	0.041000836
ENSG00000204498	NFKBIL1	-0.538349511	0.041101864
ENSG00000134056	MRPS36	-0.477634342	0.041129802
ENSG00000005889	ZFX	0.430223485	0.041129802
ENSG00000211956	IGHV4-34	-2.172525313	0.041136114
ENSG00000250151	ARPC4-TTLL3	1.278367205	0.04121907
ENSG00000053900	ANAPC4	-0.452468824	0.04122998
ENSG00000113716	HMGXB3	0.515021026	0.04122998
ENSG00000188994	ZNF292	0.646942274	0.04122998
ENSG00000111850	SMIM8	-0.709960319	0.04122998
ENSG00000164896	FASTK	-0.464674551	0.04122998
ENSG00000140093	SERPINA10	1.081806644	0.04122998
ENSG00000176182	MYPOP	-0.644262633	0.041252576
ENSG00000136068	FLNB	1.255746722	0.041307809
ENSG00000244313		-0.495374021	0.041307809
ENSG00000059573	ALDH18A1	-0.78946868	0.041307809
ENSG00000155366	RHOC	-0.999335826	0.041575484
ENSG00000151366	NDUFC2	-0.480518107	0.041775988
ENSG00000152484	USP12	0.612807653	0.041812197
ENSG00000152942	RAD17	-0.43926317	0.041876117
ENSG00000214837	LINC01347	0.683228702	0.042079574
ENSG00000064989	CALCRL	1.015284999	0.042079574
ENSG00000172215	CXCR6	-3.094011166	0.042079574
ENSG00000137411	VARS2	-0.677797605	0.042079574
ENSG00000137038	TMEM261	-0.697762687	0.042079574
ENSG00000178385	PLEKHM3	0.739302205	0.042112829
ENSG00000105701	FKBP8	-0.329476009	0.042139473
ENSG00000175556	LONRF3	1.623222537	0.042344602
ENSG00000163681	SLMAP	0.360179808	0.042350077
ENSG00000141295	SCRN2	-0.646756333	0.042433675
ENSG00000007392	LUC7L	-0.425680524	0.042435215
ENSG00000213339	QTRT1	-0.531245531	0.042437675
ENSG00000182584	ACTL10	-1.479857702	0.042639209
ENSG00000114391	RPL24	-0.374240223	0.042687469
ENSG00000196510	ANAPC7	-0.481863706	0.042687469
ENSG00000183604	SMG1P5	0.880796342	0.042687469
ENSG00000275895	U2AF1L5	5.913744174	0.042687469
ENSG00000147996	CBWD5	-0.688029137	0.042785673
ENSG00000187713	TMEM203	-0.636473079	0.042785673

ENSG00000164081	TEX264	-0.540537203	0.042968209
ENSG00000162407	PLPP3	3.145969591	0.043022223
ENSG00000138081	FBXO11	0.449977243	0.043022223
ENSG00000153563	CD8A	-2.050716022	0.043022223
ENSG00000156453	PCDH1	-3.12587616	0.043022223
ENSG00000211892	IGHG4	-3.166033524	0.043022223
ENSG00000125863	MKKS	-0.897783103	0.043022223
ENSG00000276712	MIR7111	-1.361628487	0.043024639
ENSG00000243302		0.718076454	0.043024639
ENSG00000083838	ZNF446	-0.512358189	0.043024639
ENSG00000138138	ATAD1	-0.479364235	0.04305438
ENSG00000100911	PSME2	-0.626742098	0.043168295
ENSG00000074054	CLASP1	0.476317638	0.043224672
ENSG00000100417	PMM1	-0.44781228	0.043234026
ENSG00000197756	RPL37A	-0.445030749	0.043352636
ENSG00000117543	DPH5	-0.549269986	0.043532228
ENSG00000038358	EDC4	0.486825432	0.04355646
ENSG00000270605		-1.147999537	0.043560072
ENSG00000169031	COL4A3	-2.784241315	0.043596329
ENSG00000185201	IFITM2	-0.680326556	0.043596329
ENSG00000089248	ERP29	-0.335387141	0.043596329
ENSG00000007376	RPUSD1	-0.479283752	0.043596329
ENSG00000165283	STOML2	-0.403867517	0.043602501
ENSG00000241839	PLEKHO2	1.038009555	0.043602501
ENSG00000181284	TMEM102	-0.911080072	0.043602501
ENSG00000134250	NOTCH2	0.552101049	0.04367452
ENSG00000165029	ABCA1	2.249596942	0.043707196
ENSG00000224051	CPTP	-0.728960139	0.043714595
ENSG00000159267	HLCS	0.687399117	0.043782987
ENSG00000204397	CARD16	-0.617636936	0.043795369
ENSG00000139514	SLC7A1	0.96426974	0.044117767
ENSG00000175518	UBQLNL	-1.764303285	0.044146853
ENSG00000106628	POLD2	-0.550058631	0.044175918
ENSG00000008513	ST3GAL1	0.614799761	0.044175918
ENSG00000076108	BAZ2A	0.500597808	0.04433456
ENSG00000173064	HECTD4	0.495802002	0.044356752
ENSG00000013619	MAMLD1	3.182839061	0.044431646
ENSG00000111906	HDDC2	-0.621072395	0.044687602
ENSG00000170515	PA2G4	-0.543868305	0.044687602
ENSG00000103160	HSDL1	-0.424304955	0.044687602
ENSG00000165821	SALL2	-2.520845648	0.0446893
ENSG00000108021	FAM208B	0.520706809	0.04473796
ENSG00000175416	CLTB	-0.441417134	0.044813376
ENSG00000108465	CDK5RAP3	-0.397619978	0.044890508
ENSG00000221983	UBA52	-0.378732299	0.044890508
ENSG00000162639	HENMT1	-0.614746534	0.045033552
ENSG00000204099	NEU4	4.571648435	0.045252122
ENSG00000130340	SNX9	1.710301274	0.045252122

ENSG00000164985	PSIP1	-0.477574706	0.045252122
ENSG00000100731	PCNX1	0.48188969	0.045254671
ENSG00000185808	PIGP	-0.579636581	0.045263533
ENSG00000279417		1.17850504	0.045319801
ENSG00000158042	MRPL17	-0.851891606	0.045339578
ENSG00000108559	NUP88	-0.410590254	0.045375128
ENSG00000005483	KMT2E	0.556782054	0.045426292
ENSG00000153815	CMIP	0.414299968	0.045426292
ENSG00000113312	TTC1	-0.565693253	0.045661177
ENSG00000214160	ALG3	-0.477797403	0.045678508
ENSG00000136271	DDX56	-0.348796062	0.045678508
ENSG00000113742	CPEB4	0.613652719	0.045793706
ENSG00000196459	TRAPPC2	-0.632702775	0.045793706
ENSG00000266094	RASSF5	0.481892776	0.045806928
ENSG00000169129	AFAP1L2	-2.635499441	0.045806928
ENSG00000146066	HIGD2A	-0.406287221	0.045985098
ENSG00000119632	IFI27L2	-0.661359919	0.045985098
ENSG00000100804	PSMB5	-0.626161402	0.046091549
ENSG00000169696	ASPSCR1	-0.532934677	0.046091549
ENSG00000141867	BRD4	0.394385805	0.046091549
ENSG00000198855	FICD	0.736091453	0.046333313
ENSG00000068120	COASY	-0.487687283	0.04633353
ENSG00000279838		1.125539749	0.046444112
ENSG00000165264	NDUFB6	-0.643801407	0.046444112
ENSG00000234608	MAPKAPK5-AS1	-0.578323069	0.046453159
ENSG00000163682	RPL9	-0.486288806	0.046509883
ENSG00000149806	FAU	-0.39010556	0.04656741
ENSG00000163960	UBXN7	0.469049934	0.046642192
ENSG00000198218	QRICH1	0.399087459	0.046739914
ENSG00000165288	BRWD3	0.461127357	0.046739914
ENSG00000253865		-1.512638706	0.046822565
ENSG00000124177	CHD6	0.496285604	0.046827136
ENSG00000276278		-0.830475578	0.046855934
ENSG00000261596		-0.8753326	0.046855934
ENSG00000127054	CPSF3L	-0.333532212	0.046870577
ENSG00000233184		-0.83810111	0.046870577
ENSG00000243943	ZNF512	-0.760413436	0.046870577
ENSG00000153827	TRIP12	0.343271928	0.046870577
ENSG00000235043	TECRP1	-0.89425472	0.046870577
ENSG00000140396	NCOA2	0.437324447	0.046870577
ENSG00000211792	TRAV14DV4	-2.825447806	0.046870577
ENSG00000158545	ZC3H18	0.370776374	0.046870577
ENSG00000175826	CTDNEP1	-0.281163998	0.046870577
ENSG00000132635	PCED1A	-0.467450736	0.046870577
ENSG00000174851	YIF1A	-0.580696444	0.047034157
ENSG00000158865	SLC5A11	4.463636688	0.047034157
ENSG00000105618	PRPF31	-0.250974817	0.047034157
ENSG00000062524	LTK	-1.752478068	0.047155952

ENSG00000174775	HRAS	-0.527554391	0.047199047
ENSG00000218208	RPS27AP11	-1.358157596	0.047273666
ENSG00000170962	PDGFD	-2.848460513	0.047273666
ENSG00000083750	RRAGB	-0.47812908	0.047298109
ENSG00000176542	USF3	0.552887719	0.047449321
ENSG00000176171	BNIP3	-0.674813715	0.047449321
ENSG00000117519	CNN3	-2.418513176	0.047549774
ENSG00000113648	H2AFY	0.409077499	0.047549774
ENSG00000211727	TRBV7-6	-2.8067004	0.047549774
ENSG00000228956	SATB1-AS1	-1.150655167	0.047632971
ENSG00000156469	MTERF3	-0.579078213	0.047632971
ENSG00000277147	LINC00869	-0.611240371	0.047848454
ENSG00000065613	SLK	0.482132723	0.047848454
ENSG00000177374	HIC1	2.512285629	0.047848454
ENSG00000099901	RANBP1	-0.527919484	0.047848454
ENSG00000146535	GNA12	0.715639718	0.04800415
ENSG00000165685	TMEM52B	1.930705573	0.048029777
ENSG00000212864	RNF208	-1.188753499	0.04803598
ENSG00000276557	TRBV18	-2.80364527	0.048122956
ENSG00000005007	UPF1	0.327711974	0.04816041
ENSG00000185909	KLHDC8B	-0.910884779	0.048210537
ENSG00000211749	TRBV23-1	-2.202724337	0.048210537
ENSG00000125485	DDX31	-0.705100038	0.048210537
ENSG00000105705	SUGP1	-0.446533939	0.048210537
ENSG00000163814	CDCP1	2.585311156	0.048273761
ENSG00000198034	RPS4X	-0.374882348	0.048273761
ENSG00000184224	C11orf72	-0.590217528	0.048273761
ENSG00000235194	PPP1R3E	-0.676363427	0.048273761
ENSG00000170160	CCDC144A	2.0941978	0.048273761
ENSG00000176083	ZNF683	-2.746475406	0.048297436
ENSG00000136938	ANP32B	-0.413056473	0.048297436
ENSG00000211799	TRAV19	-2.622837243	0.048297436
ENSG00000161970	RPL26	-0.425785903	0.048297436
ENSG00000095066	HOOK2	-0.586715222	0.048297436
ENSG00000123349	PFDN5	-0.378672844	0.048338906
ENSG00000246089		-0.859377388	0.048349634
ENSG00000174365	SNHG11	-0.548663065	0.048349634
ENSG00000232573	RPL3P4	-0.513539086	0.048400112
ENSG00000108107	RPL28	-0.498554608	0.048505086
ENSG00000141741	MIEN1	-0.433235287	0.048578998
ENSG00000175691	ZNF77	-0.661634871	0.048597452
ENSG00000221957	KIR2DS4	-2.836379083	0.048630853
ENSG00000171604	CXXC5	1.237317544	0.048660088
ENSG00000196405	EVL	-0.561072486	0.04878382
ENSG00000105372	RPS19	-0.450332596	0.04878382
ENSG00000170310	STX8	-0.562564209	0.048815188
ENSG00000138434	SSFA2	0.528129852	0.0488323
ENSG00000275210		1.076490581	0.049225823

ENSG00000123636	BAZ2B	0.560267986	0.04934826
ENSG00000111237	VPS29	-0.430740893	0.04934826
ENSG00000165795	NDRG2	-0.658068382	0.049404882
ENSG00000175334	BANF1	-0.649785154	0.049445404
ENSG00000055483	USP36	1.123912858	0.049483958
ENSG00000088826	SMOX	2.190063302	0.049483958
ENSG00000116213	WRAP73	-0.433489267	0.04952632
ENSG00000121743	GJA3	4.694189654	0.049555674
ENSG00000232164		1.005172495	0.04964665
ENSG00000166260	COX11	-0.576599855	0.049649613
ENSG00000161671	EMC10	-0.255675902	0.049649613
ENSG00000151612	ZNF827	-1.351829445	0.049771673
ENSG00000096080	MRPS18A	-0.526340363	0.049801233
ENSG00000143924	EML4	0.660037635	0.049852282
