# Supplementary material for: Role of CD14+ monocyte-derived oxidised mitochondrial DNA in the inflammatory interferon type 1 signature in juvenile dermatomyositis
Source: Ann Rheum Dis. 2022 Dec 23;82(5):658–69. doi: 10.1136/ard-2022-223469 (PMC10176342; doi:10.1136/ard-2022-223469)
Supplement: Supplementary data [file ard-2022-223469supp004.pdf]

Supplementary Table 3 - JDM pre-treatment vs on-treatment significantly DEG (p≤0.05)

Ensemble ID	hgnc_symbol	log fold change	Adjusted p value
ENSG00000185885	IFITM1	3.39862585	4.74E-33
ENSG00000055130	CUL1	1.164987546	3.41E-25
ENSG00000172183	ISG20	2.238676981	1.46E-23
ENSG00000183486	MX2	2.125286181	9.79E-22
ENSG00000136816	TOR1B	1.710487232	5.11E-21
ENSG00000134321	RSAD2	3.866490322	3.14E-20
ENSG00000124256	ZBP1	2.703954114	4.35E-20
ENSG00000138646	HERC5	3.179408173	9.85E-20
ENSG00000119922	IFIT2	3.804908949	2.40E-19
ENSG00000138035	PNPT1	1.77089402	3.41E-19
ENSG00000140464	PML	1.333071397	4.59E-19
ENSG00000165185	KIAA1958	1.555823901	5.45E-19
ENSG00000130487	KLHDC7B	2.573064724	5.45E-19
ENSG00000059378	PARP12	1.457083108	2.77E-18
ENSG00000184979	USP18	4.107072601	2.77E-18
ENSG00000163644	PPM1K	1.890727422	1.28E-17
ENSG00000187608	ISG15	3.03596484	1.51E-17
ENSG00000107201	DDX58	2.13754173	2.14E-17
ENSG00000137200	CMTR1	1.092391744	3.45E-17
ENSG00000115267	IFIH1	1.934848208	9.16E-16
ENSG00000138642	HERC6	2.337045112	1.80E-15
ENSG00000132274	TRIM22	1.28588343	1.95E-15
ENSG00000100220	RTCB	1.199853067	1.98E-15
ENSG00000188157	AGRN	2.05537899	2.86E-15
ENSG00000086065	CHMP5	1.371126076	5.01E-15
ENSG00000108771	DHX58	1.848937929	6.53E-15
ENSG00000163666	HESX1	3.695272206	6.61E-15
ENSG00000137628	DDX60	2.127830975	6.61E-15
ENSG00000157601	MX1	2.598859926	1.73E-14
ENSG00000160710	ADAR	0.977133921	2.02E-14
ENSG00000105726	ATP13A1	0.976478109	3.23E-14
ENSG00000173193	PARP14	1.576849779	3.99E-14
ENSG00000055332	EIF2AK2	1.601466148	6.89E-14
ENSG00000111912	NCOA7	1.360989368	7.47E-14
ENSG00000017483	SLC38A5	2.431681538	7.47E-14
ENSG00000172432	GTPBP2	0.890297882	8.81E-14
ENSG00000205413	SAMD9	2.142860258	9.27E-14
ENSG00000185745	IFIT1	4.09959233	1.09E-13
ENSG00000134326	CMPK2	2.888452244	1.40E-13
ENSG00000111335	OAS2	2.603698835	1.44E-13
ENSG00000130589	HELZ2	1.632039344	1.73E-13
ENSG00000164054	SHISA5	1.074129998	2.00E-13
ENSG00000160932	LY6E	1.955040544	3.81E-13
ENSG00000138385	SSB	0.93349414	3.88E-13

ENSG00000137198	GMPR	3.067849652	7.88E-13
ENSG00000068079	IFI35	1.613553029	1.41E-12
ENSG00000185201	IFITM2	1.26248619	2.89E-12
ENSG00000170581	STAT2	1.353047658	2.92E-12
ENSG00000118922	KLF12	-1.403238223	3.65E-12
ENSG00000111331	OAS3	2.585404184	6.99E-12
ENSG00000162614	NEXN	2.681249354	8.16E-12
ENSG00000154122	ANKH	-1.750924848	8.84E-12
ENSG00000105939	ZC3HAV1	0.950150676	1.11E-11
ENSG00000196116	TDRD7	1.251876971	1.20E-11
ENSG00000137959	IFI44L	3.167495964	1.32E-11
ENSG00000117010	ZNF684	1.305517008	1.37E-11
ENSG00000138496	PARP9	1.979935564	1.46E-11
ENSG00000100129	EIF3L	-0.808894816	1.60E-11
ENSG00000122643	NT5C3A	1.527976155	1.96E-11
ENSG00000162496	DHRS3	-7.188957132	2.02E-11
ENSG00000126709	IFI6	2.337677573	2.75E-11
ENSG00000076685	NT5C2	0.639429253	3.22E-11
ENSG00000137965	IFI44	2.419785274	3.35E-11
ENSG00000145287	PLAC8	1.458065947	4.91E-11
ENSG00000152102	FAM168B	-0.662531972	6.19E-11
ENSG00000119917	IFIT3	3.650993356	6.50E-11
ENSG00000146859	TMEM140	1.582845349	6.89E-11
ENSG00000185507	IRF7	1.716623152	7.29E-11
ENSG00000108700	CCL8	5.675703163	7.62E-11
ENSG00000108679	LGALS3BP	2.067284751	8.43E-11
ENSG00000133106	EPSTI1	2.031143247	9.29E-11
ENSG00000111344	RASAL1	-6.839063999	1.22E-10
ENSG00000163840	DTX3L	1.708226909	2.62E-10
ENSG00000188542	DUSP28	-0.714087951	2.62E-10
ENSG00000247317		2.306715849	2.85E-10
ENSG00000106603	COA1	0.850492432	3.15E-10
ENSG00000137414	FAM8A1	0.969940671	3.28E-10
ENSG00000102921	N4BP1	0.91262895	4.33E-10
ENSG00000130813	C19orf66	0.991247446	6.69E-10
ENSG00000132530	XAF1	2.318501921	9.68E-10
ENSG00000136147	PHF11	0.907090338	1.01E-09
ENSG00000163565	IFI16	1.503157981	1.02E-09
ENSG00000226950	DANCR	-1.353528168	1.36E-09
ENSG00000135899	SP110	1.265405777	1.37E-09
ENSG00000273314		1.60592131	1.39E-09
ENSG00000035720	STAP1	2.855107916	1.61E-09
ENSG00000105655	ISYNA1	-1.63253399	2.30E-09
ENSG00000168394	TAP1	1.113012556	2.48E-09
ENSG00000130595	TNNT3	-2.729036726	2.52E-09
ENSG00000131979	GCH1	0.985678555	2.66E-09
ENSG00000132109	TRIM21	1.466413305	2.80E-09
ENSG00000152766	ANKRD22	2.152048724	3.21E-09

ENSG00000204977	TRIM13	-1.089383113	3.93E-09
ENSG00000177409	SAMD9L	2.362901281	3.97E-09
ENSG00000168395	ING5	-0.888022519	6.13E-09
ENSG00000173786	CNP	1.218181809	6.45E-09
ENSG00000196684	HSH2D	1.342544902	6.51E-09
ENSG00000244242	IFITM10	-3.373797553	6.78E-09
ENSG00000173511	VEGFB	-2.291712704	6.87E-09
ENSG00000143153	ATP1B1	-1.982808024	7.41E-09
ENSG00000272666		2.340167326	7.95E-09
ENSG00000132256	TRIM5	1.7459755	8.59E-09
ENSG00000105875	WDR91	-0.747971626	9.11E-09
ENSG00000114554	PLXNA1	-1.546334268	9.98E-09
ENSG00000020577	SAMD4A	1.762586794	1.69E-08
ENSG00000011295	TTC19	-0.927237278	1.74E-08
ENSG00000002549	LAP3	1.358550585	1.77E-08
ENSG00000106785	TRIM14	1.384610627	2.06E-08
ENSG00000206190	ATP10A	1.571632761	2.10E-08
ENSG00000186918	ZNF395	-1.034639557	2.16E-08
ENSG00000120885	CLU	2.768088043	2.16E-08
ENSG00000136514	RTP4	2.01509088	2.26E-08
ENSG00000090621	PABPC4	-0.890861943	2.78E-08
ENSG00000118971	CCND2	-1.344484191	2.88E-08
ENSG00000115548	KDM3A	-1.014779224	2.98E-08
ENSG00000169116	PARM1	-3.366287644	3.15E-08
ENSG00000197381	ADARB1	1.035654568	3.36E-08
ENSG00000011028	MRC2	-5.213021828	3.50E-08
ENSG00000187109	NAP1L1	-0.673935184	3.87E-08
ENSG00000151012	SLC7A11	-4.836930734	4.24E-08
ENSG00000223960		1.301859947	4.28E-08
ENSG00000184205	TSPYL2	-2.231777018	4.52E-08
ENSG00000063046	EIF4B	-0.711048257	5.39E-08
ENSG00000167658	EEF2	-0.518344329	6.05E-08
ENSG00000152778	IFIT5	1.658173162	7.45E-08
ENSG00000160683	CXCR5	-3.467915731	9.19E-08
ENSG00000142303	ADAMTS10	-1.464790211	9.78E-08
ENSG00000181381	DDX60L	1.280730632	1.00E-07
ENSG00000249115	HAUS5	-0.672667761	1.03E-07
ENSG00000160223	ICOSLG	-2.039035137	1.08E-07
ENSG00000204252	HLA-DOA	-1.638926132	1.17E-07
ENSG00000225964	NRIR	2.436769194	1.19E-07
ENSG00000126351	THRA	-1.035777727	1.32E-07
ENSG00000106605	BLVRA	1.206214582	1.32E-07
ENSG00000168036	CTNNB1	-0.83374275	1.36E-07
ENSG00000183049	CAMK1D	-0.733665124	1.44E-07
ENSG00000138190	EXOC6	0.765153527	1.51E-07
ENSG00000186806	VSIG10L	1.262496147	1.60E-07
ENSG00000111224	PARP11	0.866476841	1.72E-07
ENSG00000135114	OASL	2.504420841	1.72E-07

ENSG00000136807	CDK9	-0.539151053	1.88E-07
ENSG00000274307		1.336375001	1.88E-07
ENSG00000173821	RNF213	1.004067082	2.07E-07
ENSG00000188042	ARL4C	-1.774995168	2.14E-07
ENSG00000114942	EEF1B2	-0.601299458	2.34E-07
ENSG00000173166	RAPH1	-1.95395738	2.63E-07
ENSG00000166889	PATL1	0.713042639	2.65E-07
ENSG00000122406	RPL5	-0.537680981	3.44E-07
ENSG00000142089	IFITM3	2.029849262	3.51E-07
ENSG00000177374	HIC1	-3.240136612	3.51E-07
ENSG00000169245	CXCL10	3.806084402	3.59E-07
ENSG00000196141	SPATS2L	2.014015163	3.90E-07
ENSG00000155252	PI4K2A	-1.562633789	4.07E-07
ENSG00000166016	ABTB2	2.093295717	4.48E-07
ENSG00000078043	PIAS2	-0.614212044	4.54E-07
ENSG00000104660	LEPROTL1	0.900080769	4.62E-07
ENSG00000099381	SETD1A	-0.666231938	4.67E-07
ENSG00000205837	LINC00487	3.087271877	4.93E-07
ENSG00000107554	DNMBP	-0.952033074	5.54E-07
ENSG00000277511		1.77450674	5.56E-07
ENSG00000137496	IL18BP	-1.040802477	5.67E-07
ENSG00000038210	PI4K2B	1.163066402	5.86E-07
ENSG00000213923	CSNK1E	-0.766043381	6.19E-07
ENSG00000141580	WDR45B	-0.558538853	6.34E-07
ENSG00000090975	PITPNM2	-1.130435881	6.35E-07
ENSG00000079263	SP140	1.246952671	6.36E-07
ENSG00000089127	OAS1	2.212336219	6.46E-07
ENSG00000237276	ANO7L1	1.779747225	6.51E-07
ENSG00000155363	MOV10	0.79261934	6.51E-07
ENSG00000105639	JAK3	0.821263537	6.59E-07
ENSG00000013619	MAMLD1	-4.085532493	6.96E-07
ENSG00000145016	RUBCN	0.595392711	7.12E-07
ENSG00000111907	TPD52L1	1.840165917	7.37E-07
ENSG00000156587	UBE2L6	1.215580969	7.71E-07
ENSG00000117226	GBP3	1.100639307	7.78E-07
ENSG00000174748	RPL15	-0.433326646	7.78E-07
ENSG00000150907	FOXO1	-0.79279773	7.78E-07
ENSG00000197111	PCBP2	-0.474091574	8.44E-07
ENSG00000072858	SIDT1	-1.108238212	8.66E-07
ENSG00000184441		-1.00444378	8.66E-07
ENSG00000007264	MATK	-2.898568619	8.87E-07
ENSG00000152061	RABGAP1L	0.880162684	9.09E-07
ENSG00000152484	USP12	-0.780864659	9.26E-07
ENSG00000137337	MDC1	-0.702141844	1.05E-06
ENSG00000089057	SLC23A2	-0.743401657	1.05E-06
ENSG00000153933	DGKE	-0.80495567	1.11E-06
ENSG00000163961	RNF168	-0.776970575	1.14E-06
ENSG00000099814	CEP170B	-2.880547134	1.18E-06

ENSG00000103264	FBXO31	-0.65283109	1.18E-06
ENSG00000198417	MT1F	1.419087813	1.35E-06
ENSG00000221963	APOL6	1.075556893	1.36E-06
ENSG00000108387	Sep-04	1.519492957	1.39E-06
ENSG00000188011	RTP5	-5.656952796	1.42E-06
ENSG00000101596	SMCHD1	0.715963375	1.65E-06
ENSG00000156650	KAT6B	-0.636735769	1.80E-06
ENSG00000111641	NOP2	-0.731968652	1.95E-06
ENSG00000140332	TLE3	-0.914136936	1.99E-06
ENSG00000137936	BCAR3	-2.532099701	2.01E-06
ENSG00000116863	ADPRHL2	0.892617688	2.07E-06
ENSG00000122224	LY9	-1.747438413	2.17E-06
ENSG00000117475	BLZF1	0.679762449	2.34E-06
ENSG00000136682	CBWD2	0.630063435	2.40E-06
ENSG00000135094	SDS	-2.9578259	2.74E-06
ENSG00000135622	SEMA4F	1.321107742	2.77E-06
ENSG00000116663	FBXO6	1.237564887	2.81E-06
ENSG00000149313	AASDHPPT	0.756579925	2.81E-06
ENSG00000115415	STAT1	1.426331566	3.14E-06
ENSG00000198087	CD2AP	0.867196522	3.45E-06
ENSG00000079616	KIF22	-0.656455595	3.46E-06
ENSG00000156983	BRPF1	-0.573671399	3.47E-06
ENSG00000152558	TMEM123	0.925174701	3.47E-06
ENSG00000182179	UBA7	0.612332345	4.01E-06
ENSG00000188315	C3orf62	-0.591170073	4.35E-06
ENSG00000171604	CXXC5	-1.490211538	4.50E-06
ENSG00000089157	RPLP0	-0.522383942	4.50E-06
ENSG00000070961	ATP2B1	-1.55794677	4.64E-06
ENSG00000185722	ANKFY1	0.640516461	4.64E-06
ENSG00000198604	BAZ1A	0.487198546	4.67E-06
ENSG00000117228	GBP1	1.713005252	4.87E-06
ENSG00000162407	PLPP3	-3.54686187	4.92E-06
ENSG00000062598	ELMO2	0.575344044	5.03E-06
ENSG00000178685	PARP10	1.188771447	5.16E-06
ENSG00000136877	FPGS	-0.904542002	5.28E-06
ENSG00000111371	SLC38A1	-1.052010974	5.88E-06
ENSG00000067066	SP100	0.654604751	6.07E-06
ENSG00000021355	SERPINB1	0.511822821	6.07E-06
ENSG00000089094	KDM2B	-0.504498644	6.11E-06
ENSG00000140280	LYSMD2	0.789711493	7.29E-06
ENSG00000170175	CHRNB1	1.014278816	7.61E-06
ENSG00000165804	ZNF219	-1.496984008	7.76E-06
ENSG00000100814	CCNB1IP1	-0.575396059	7.76E-06
ENSG00000008083	JARID2	-1.103933263	7.95E-06
ENSG00000141385	AFG3L2	-0.447760418	7.95E-06
ENSG00000115155	OTOF	3.857485425	7.98E-06
ENSG00000239713	APOBEC3G	1.184218522	7.98E-06
ENSG00000123609	NMI	1.231034648	8.36E-06

ENSG00000228106		-1.183741944	8.42E-06
ENSG00000115159	GPD2	0.718650372	8.49E-06
ENSG00000185880	TRIM69	1.291070632	8.53E-06
ENSG00000167680	SEMA6B	-4.661726901	9.16E-06
ENSG00000065923	SLC9A7	-1.232417107	1.03E-05
ENSG00000160271	RALGDS	-1.038228728	1.04E-05
ENSG00000100600	LGMN	-2.040086501	1.05E-05
ENSG00000040199	PHLPP2	-0.699169146	1.09E-05
ENSG00000166527	CLEC4D	0.977116827	1.14E-05
ENSG00000087589	CASS4	-1.238973567	1.15E-05
ENSG00000084112	SSH1	-0.658357856	1.15E-05
ENSG00000167601	AXL	2.291470244	1.18E-05
ENSG00000168118	RAB4A	-0.420845252	1.21E-05
ENSG00000164687	FABP5	-2.260390179	1.22E-05
ENSG00000179840	PIK3CD-AS1	1.535622504	1.23E-05
ENSG00000168234	TTC39C	0.668293223	1.23E-05
ENSG00000196329	GIMAP5	1.743179476	1.24E-05
ENSG00000182378	PLCXD1	-1.31576293	1.24E-05
ENSG00000179361	ARID3B	-0.817864248	1.26E-05
ENSG00000179144	GIMAP7	1.923890158	1.31E-05
ENSG00000269951		-1.54628417	1.32E-05
ENSG00000213689	TREX1	1.028890952	1.42E-05
ENSG00000148516	ZEB1	-2.091137645	1.44E-05
ENSG00000167747	C19orf48	-0.810855845	1.46E-05
ENSG00000186480	INSIG1	-1.210473156	1.49E-05
ENSG00000176102	CSTF3	0.614702984	1.51E-05
ENSG00000171316	CHD7	-1.114085896	1.57E-05
ENSG00000167208	SNX20	0.62475524	1.62E-05
ENSG00000165943	MOAP1	-0.726591275	1.62E-05
ENSG00000163319	MRPS18C	0.779359451	1.62E-05
ENSG00000174738	NR1D2	-1.272606588	1.67E-05
ENSG00000169251	NMD3	-0.595299337	1.71E-05
ENSG00000172053	QARS	-0.354545675	1.80E-05
ENSG00000127586	CHTF18	-0.730284598	1.86E-05
ENSG00000179044	EXOC3L1	1.717437121	1.89E-05
ENSG00000186567	CEACAM19	-1.267100062	1.89E-05
ENSG00000183655	KLHL25	-1.382444036	1.90E-05
ENSG00000204519	ZNF551	-0.875989431	1.95E-05
ENSG00000107371	EXOSC3	0.895054014	2.00E-05
ENSG00000174444	RPL4	-0.440854929	2.00E-05
ENSG00000168062	BATF2	2.240757993	2.02E-05
ENSG00000197121	PGAP1	1.447110583	2.06E-05
ENSG00000181045	SLC26A11	-1.166545948	2.06E-05
ENSG00000141542	RAB40B	-1.022591809	2.09E-05
ENSG00000106991	ENG	-1.122279696	2.15E-05
ENSG00000106089	STX1A	-2.870610165	2.24E-05
ENSG00000169100	SLC25A6	-0.436436931	2.25E-05
ENSG00000149131	SERPING1	2.288858944	2.25E-05

ENSG00000103942	HOMER2	-1.27813326	2.62E-05
ENSG00000136874	STX17	0.613744675	2.79E-05
ENSG00000170989	S1PR1	-1.784781687	2.81E-05
ENSG00000011566	MAP4K3	-1.132576152	2.84E-05
ENSG00000244968	LIFR-AS1	1.57107458	3.14E-05
ENSG00000197771	MCMBP	0.796348939	3.16E-05
ENSG00000172345	STARD5	-1.389809437	3.16E-05
ENSG00000142687	KIAA0319L	0.626951341	3.28E-05
ENSG00000163376	KBTBD8	-1.344776184	3.28E-05
ENSG00000090971	NAT14	-0.900750389	3.35E-05
ENSG00000141577	CEP131	-0.630148552	3.46E-05
ENSG00000100316	RPL3	-0.458586504	3.49E-05
ENSG00000013374	NUB1	0.718228941	3.51E-05
ENSG00000250575		-1.025266105	3.54E-05
ENSG00000187912	CLEC17A	-1.71599741	3.54E-05
ENSG00000174238	PITPNA	-0.629925673	3.58E-05
ENSG00000278948		-0.807242396	3.78E-05
ENSG00000081913	PHLPP1	-0.784347179	3.81E-05
ENSG00000115594	IL1R1	-2.656777092	3.86E-05
ENSG00000229677		1.466942666	3.87E-05
ENSG00000176903	PNMA1	-0.856810624	4.04E-05
ENSG00000047634	SCML1	-1.254746261	4.11E-05
ENSG00000136960	ENPP2	1.823721083	4.19E-05
ENSG00000263528	IKBKE	0.760962027	4.19E-05
ENSG00000041357	PSMA4	0.626588215	4.21E-05
ENSG00000168310	IRF2	0.893810835	4.22E-05
ENSG00000137094	DNAJB5	-1.441911986	4.27E-05
ENSG00000075275	CELSR1	-2.480099685	4.27E-05
ENSG00000181873	IBA57	-1.112273742	4.34E-05
ENSG00000133657	ATP13A3	-1.151906378	4.34E-05
ENSG00000133574	GIMAP4	1.73080655	4.34E-05
ENSG00000004660	CAMKK1	-1.252767227	4.34E-05
ENSG00000278133		-0.586839078	4.45E-05
ENSG00000179639	FCER1A	-3.168083362	4.48E-05
ENSG00000257167	TMPO-AS1	1.357880418	4.79E-05
ENSG00000260314	MRC1	-1.867118896	4.82E-05
ENSG00000213553	RPLP0P6	-0.567495616	4.92E-05
ENSG00000009844	VTA1	0.649836472	4.93E-05
ENSG00000120802	TMPO	0.764181361	4.95E-05
ENSG00000108984	MAP2K6	1.100308846	4.95E-05
ENSG00000130303	BST2	0.987622762	5.00E-05
ENSG00000185477	GPRIN3	-1.284802201	5.08E-05
ENSG00000125148	MT2A	1.454438015	5.08E-05
ENSG00000234771	SLC25A25-AS1	-0.705097171	5.29E-05
ENSG00000234076	TPRG1-AS1	1.581286793	5.35E-05
ENSG00000077782	FGFR1	-1.282063118	5.37E-05
ENSG00000153107	ANAPC1	-0.397941423	5.57E-05
ENSG00000011478	QPCTL	-0.777385003	5.63E-05

ENSG00000055147	FAM114A2	0.665563046	5.78E-05
ENSG00000105855	ITGB8	-2.718925735	5.93E-05
ENSG00000198133	TMEM229B	1.15327429	5.93E-05
ENSG00000169871	TRIM56	0.775608291	6.07E-05
ENSG00000010030	ETV7	2.594473037	6.15E-05
ENSG00000138050	THUMPD2	-0.771335101	6.25E-05
ENSG00000025770	NCAPH2	-0.726976069	6.41E-05
ENSG00000087502	ERGIC2	0.625494534	6.65E-05
ENSG00000178562	CD28	-1.954441242	6.71E-05
ENSG00000161996	WDR90	-0.852152073	6.87E-05
ENSG00000160408	ST6GALNAC6	-0.710024092	7.68E-05
ENSG00000279700		-1.454026647	7.68E-05
ENSG00000121858	TNFSF10	1.749419492	7.72E-05
ENSG00000151445	VIPAS39	0.699011261	7.72E-05
ENSG00000114127	XRN1	0.496051553	7.74E-05
ENSG00000118689	FOXO3	-0.901663426	7.81E-05
ENSG00000172175	MALT1	-0.580820234	7.81E-05
ENSG00000100083	GGA1	-0.561239318	7.93E-05
ENSG00000112576	CCND3	0.814158484	7.96E-05
ENSG00000173848	NET1	-1.562362603	8.11E-05
ENSG00000149798	CDC42EP2	1.156420117	8.60E-05
ENSG00000121486	TRMT1L	0.829774148	8.74E-05
ENSG00000178988	MRFAP1L1	0.746308646	9.32E-05
ENSG00000214193	SH3D21	-1.422035038	9.54E-05
ENSG00000165322	ARHGAP12	-0.65845662	9.72E-05
ENSG00000156030	ELMSAN1	-0.64073141	9.86E-05
ENSG00000228794	LINC01128	-0.868981877	9.93E-05
ENSG00000180354	MTURN	-0.774395092	0.000100761
ENSG00000104824	HNRNPL	-0.428226405	0.000102601
ENSG00000123240	OPTN	0.980441457	0.000103815
ENSG00000091844	RGS17	-1.163278233	0.000106195
ENSG00000135972	MRPS9	0.547856247	0.000106573
ENSG00000119392	GLE1	0.463023511	0.000110155
ENSG00000177294	FBXO39	2.166020902	0.000111065
ENSG00000167962	ZNF598	-0.383260068	0.000111582
ENSG00000121743	GJA3	-4.663854813	0.000112057
ENSG00000100084	HIRA	0.536134223	0.000115463
ENSG00000126804	ZBTB1	-0.634843883	0.000116396
ENSG00000263465	SRSF8	-0.548968652	0.000120269
ENSG00000204590	GNL1	-0.656763835	0.000124912
ENSG00000188428	BLOC1S5	0.713600295	0.00012625
ENSG00000089009	RPL6	-0.385750505	0.000127094
ENSG00000141664	ZCCHC2	0.798179934	0.000129966
ENSG00000137409	MTCH1	-0.564803158	0.00013145
ENSG00000169490	TM2D2	0.613147499	0.00013145
ENSG00000128394	APOBEC3F	1.001475441	0.000138154
ENSG00000235374	SSR4P1	1.152551738	0.000140558
ENSG00000137767	SQRDL	0.635331095	0.000142421

ENSG00000010165	METTL13	1.146182024	0.000142564
ENSG00000157693	TMEM268	0.533761546	0.000143969
ENSG00000128513	POT1	0.652853904	0.000144108
ENSG00000110435	PDHX	0.510902232	0.000145327
ENSG00000153207	AHCTF1	-0.955919729	0.000146229
ENSG00000168092	PAFAH1B2	-0.568709913	0.000146229
ENSG00000186395	KRT10	-0.601294301	0.000146229
ENSG00000142039	CCDC97	0.547267037	0.000151973
ENSG00000109046	WSB1	0.641472881	0.000153219
ENSG00000153914	SREK1	-0.602385998	0.000154176
ENSG00000100353	EIF3D	-0.275762782	0.000154477
ENSG00000169016	E2F6	-0.612782077	0.000155566
ENSG00000162654	GBP4	1.127807025	0.000158319
ENSG00000172366	MCRIP2	-0.760076904	0.000160855
ENSG00000221955	SLC12A8	1.163099192	0.000161946
ENSG00000136235	GPNMB	-2.321371038	0.000161946
ENSG00000166750	SLFN5	1.376716628	0.000164864
ENSG00000248124	RRN3P1	-0.732887093	0.000165366
ENSG00000136897	MRPL50	0.748939508	0.000166417
ENSG00000158042	MRPL17	0.948546956	0.000166417
ENSG00000189091	SF3B3	0.515032226	0.000166417
ENSG00000229638	RPL4P4	-0.525682406	0.00017015
ENSG00000107742	SPOCK2	-1.575424776	0.000173606
ENSG00000008130	NADK	0.662553271	0.000174577
ENSG00000105699	LSR	-2.709226241	0.000174577
ENSG00000229473	RGS17P1	-1.286837968	0.000175214
ENSG00000055483	USP36	-1.159074028	0.000177215
ENSG00000068394	GPKOW	0.6496998	0.000177994
ENSG00000188636	LDOC1L	-0.806442776	0.000190103
ENSG00000147180	ZNF711	-2.157212175	0.000190296
ENSG00000147604	RPL7	-0.446282287	0.000190296
ENSG00000079313	REXO1	-0.487040087	0.000194593
ENSG00000273015		-0.520152557	0.000195816
ENSG00000204264	PSMB8	0.546565892	0.000196593
ENSG00000165806	CASP7	0.73489208	0.000197413
ENSG00000275835	TUBGCP5	-0.647677587	0.000198525
ENSG00000167394	ZNF668	-0.754453899	0.000203653
ENSG00000188313	PLSCR1	1.115187823	0.000204169
ENSG00000152404	CWF19L2	0.595188127	0.000204169
ENSG00000007350	TKTL1	-2.676305808	0.000204704
ENSG00000154451	GBP5	1.209108397	0.000209658
ENSG00000008294	SPAG9	-0.60974498	0.000210192
ENSG00000174243	DDX23	0.582955734	0.000211991
ENSG00000198034	RPS4X	-0.412374986	0.000223443
ENSG00000087460	GNAS	-0.275284087	0.000233998
ENSG00000173575	CHD2	-0.608070916	0.00023434
ENSG00000100596	SPTLC2	0.750866178	0.000237427
ENSG00000170779	CDCA4	-1.196210439	0.000238064

ENSG00000150401	DCUN1D2	-0.455693008	0.000245156
ENSG00000225614	ZNF469	-1.540774895	0.000245156
ENSG00000116251	RPL22	-0.413756479	0.00025144
ENSG00000135486	HNRNPA1	-0.372166192	0.000253765
ENSG00000117479	SLC19A2	-1.170965105	0.000263044
ENSG00000260910	LINC00565	-2.346547811	0.000265889
ENSG00000244733		-0.970225992	0.000267499
ENSG00000006327	TNFRSF12A	-2.321590777	0.000275346
ENSG00000175061	LRRC75A-AS1	-0.44562655	0.000278005
ENSG00000240053	LY6G5B	-0.719395311	0.000278868
ENSG00000267745		0.857705384	0.000281583
ENSG00000106366	SERPINE1	-2.72667949	0.000284636
ENSG00000116983	HPCAL4	-2.407025788	0.00028785
ENSG00000065150	IPO5	-0.44573155	0.000299975
ENSG00000121671	CRY2	-0.800736661	0.000303268
ENSG00000170633	RNF34	0.719798696	0.000304054
ENSG00000159131	GART	0.7919839	0.000317404
ENSG00000225492	GBP1P1	1.959963119	0.000317887
ENSG00000186020	ZNF529	-0.728377255	0.000317935
ENSG00000088827	SIGLEC1	3.281898024	0.000319255
ENSG00000125812	GZF1	-1.008801698	0.000323248
ENSG00000188404	SELL	0.957574985	0.000327896
ENSG00000261526		-0.964661291	0.000331671
ENSG00000071575	TRIB2	0.972465536	0.000333713
ENSG00000242125	SNHG3	-0.591404545	0.000340212
ENSG00000133313	CNDP2	0.530637433	0.000342137
ENSG00000164002	EXO5	0.645052463	0.000349446
ENSG00000168040	FADD	0.746274455	0.000349446
ENSG00000183426	NPIPA1	-0.68477835	0.000349739
ENSG00000169635	HIC2	-0.771176053	0.000351248
ENSG00000075089	ACTR6	0.56638928	0.00035641
ENSG00000126767	ELK1	-0.465679119	0.000360931
ENSG00000186891	TNFRSF18	-3.215125936	0.000364104
ENSG00000179833	SERTAD2	-0.580610144	0.000364104
ENSG00000026103	FAS	1.089690817	0.000365387
ENSG00000113716	HMGXB3	-0.503814686	0.000371154
ENSG00000165934	CPSF2	0.643536001	0.00037176
ENSG00000185163	DDX51	-0.446355609	0.000376043
ENSG00000134001	EIF2S1	0.654161104	0.000377176
ENSG00000072364	AFF4	-0.587180662	0.000386373
ENSG00000150637	CD226	-2.062766121	0.000386373
ENSG00000069667	RORA	-2.062575504	0.000387295
ENSG00000184613	NELL2	-2.206263048	0.000398476
ENSG00000132661	NXT1	-0.76797271	0.000398476
ENSG00000146476	ARMT1	0.989242923	0.000401662
ENSG00000110060	PUS3	0.687919163	0.000403947
ENSG00000072415	MPP5	-0.662558609	0.000406205
ENSG00000186260	MKL2	-0.599016826	0.000406205

ENSG00000024862	CCDC28A	0.516838403	0.000407338
ENSG00000197536	C5orf56	0.952821793	0.00041531
ENSG00000183742	MACC1	-2.363135473	0.00041603
ENSG00000266947		1.070342082	0.00041603
ENSG00000008256	CYTH3	-0.839798658	0.000423775
ENSG00000102401	ARMCX3	0.627426885	0.000429185
ENSG00000099331	MYO9B	-0.431504685	0.000429185
ENSG00000125355	TMEM255A	2.438326014	0.000435395
ENSG00000095585	BLNK	1.473622657	0.000446772
ENSG00000110871	COQ5	0.679871355	0.000447428
ENSG00000120265	PCMT1	0.721502669	0.00045417
ENSG00000107651	SEC23IP	0.632687892	0.00045417
ENSG00000267278	MAP3K14-AS1	-1.20825475	0.00045417
ENSG00000099622	CIRBP	-0.369616515	0.000457678
ENSG00000116604	MEF2D	-0.888174324	0.000457805
ENSG00000124357	NAGK	0.636272269	0.000467215
ENSG00000184260	HIST2H2AC	0.834364766	0.000468306
ENSG00000140092	FBLN5	-2.619756347	0.000468306
ENSG00000112130	RNF8	0.636794264	0.000469517
ENSG00000127074	RGS13	-3.648287451	0.000485487
ENSG00000137154	RPS6	-0.400052554	0.000489491
ENSG00000166908	PIP4K2C	0.553617992	0.000492224
ENSG00000267787		0.673766048	0.000494224
ENSG00000168275	COA6	0.743198224	0.000496937
ENSG00000262001	DLGAP1-AS2	-1.25376464	0.000504685
ENSG00000059769	DNAJC25	-0.769789169	0.000507849
ENSG00000089220	PEBP1	-0.482333891	0.000510609
ENSG00000181350	LRRC75A	-1.098532463	0.000515296
ENSG00000121931	LRIF1	1.017077612	0.000516421
ENSG00000058799	YIPF1	0.494505475	0.000519794
ENSG00000162461	SLC25A34	-1.147552355	0.000521877
ENSG00000158290	CUL4B	0.434800778	0.00052673
ENSG00000168758	SEMA4C	-1.32914342	0.000534554
ENSG00000134278	SPIRE1	-1.368853735	0.000534554
ENSG00000129515	SNX6	0.446253597	0.000534651
ENSG00000119787	ATL2	-0.549171367	0.000536356
ENSG00000102699	PARP4	0.66138094	0.000536356
ENSG00000171988	JMJD1C	-0.755357307	0.000542702
ENSG00000141506	PIK3R5	-0.636028191	0.000546475
ENSG00000258839	MC1R	-1.121386423	0.000552469
ENSG00000105974	CAV1	-4.706892355	0.000555806
ENSG00000107951	MTPAP	-0.443357352	0.000567752
ENSG00000185972	CCIN	-1.2442068	0.000581306
ENSG00000085563	ABCB1	-2.9476247	0.000583079
ENSG00000162510	MATN1	-3.227287704	0.000583629
ENSG00000175573	C11orf68	0.735371849	0.000587954
ENSG00000125900	SIRPD	0.979969338	0.0005962
ENSG00000135636	DYSF	1.366026602	0.000602827

ENSG00000129465	RIPK3	0.645888137	0.000607302
ENSG00000183955	KMT5A	-0.487024526	0.000630381
ENSG00000172123	SLFN12	0.828206361	0.000641103
ENSG00000123908	AGO2	-0.56674282	0.00064834
ENSG00000140563	MCTP2	0.848449008	0.000652678
ENSG00000139354	GAS2L3	-2.020515813	0.000661021
ENSG00000158321	AUTS2	-1.493841901	0.000664983
ENSG00000267547		1.142301901	0.000664983
ENSG00000084733	RAB10	0.820251111	0.00066749
ENSG00000168913	ENHO	-1.328424093	0.00067624
ENSG00000196358	NTNG2	0.948743615	0.000679974
ENSG00000050405	LIMA1	1.125777182	0.000680458
ENSG00000145425	RPS3A	-0.457724792	0.000681416
ENSG00000142961	MOB3C	0.645737283	0.000698753
ENSG00000133059	DSTYK	-0.450296198	0.000704365
ENSG00000055609	KMT2C	-0.500068285	0.000704365
ENSG00000100744	GSKIP	0.573847976	0.000707955
ENSG00000146535	GNA12	-0.683447656	0.000708654
ENSG00000110046	ATG2A	-0.893817214	0.000708654
ENSG00000162804	SNED1	-1.375976858	0.000710093
ENSG00000123737	EXOSC9	0.516300174	0.000710093
ENSG00000196814	MVB12B	0.849846097	0.000710093
ENSG00000259004		0.976892706	0.000717706
ENSG00000124193	SRSF6	-0.420374983	0.000729468
ENSG00000276672		-1.210138361	0.000730102
ENSG00000227008		-0.709150465	0.000736821
ENSG00000152784	PRDM8	-1.631393144	0.00074195
ENSG00000173083	HPSE	0.837529447	0.000752794
ENSG00000111615	KRR1	-0.647860608	0.000755266
ENSG00000130489	SCO2	1.0201548	0.000755266
ENSG00000067334	DNTTIP2	-0.641553182	0.000756395
ENSG00000115368	WDR75	-0.409548566	0.00076425
ENSG00000087095	NLK	0.514866952	0.000765046
ENSG00000110721	CHKA	-1.108500102	0.000771349
ENSG00000206573	THUMPD3-AS1	-0.491845102	0.000778481
ENSG00000241127	YAE1D1	1.136148644	0.000778481
ENSG00000246731		1.057725225	0.000779455
ENSG00000153201	RANBP2	-0.903719437	0.000816183
ENSG00000177613	CSTF2T	0.522047078	0.00083215
ENSG00000260676	LINC01541	1.195670001	0.000835704
ENSG00000120217	CD274	1.097828593	0.000835806
ENSG00000271646		0.906616243	0.000837795
ENSG00000012983	MAP4K5	-0.463227343	0.000840541
ENSG00000165138	ANKS6	-0.988007477	0.000842167
ENSG00000006062	MAP3K14	-0.410631203	0.000842167
ENSG00000152904	GGPS1	0.706719827	0.000848915
ENSG00000224078	SNHG14	-0.704628226	0.000848915
ENSG00000151136	BTBD11	-0.881300506	0.000848991

ENSG00000108256	NUFIP2	-0.416079355	0.000848991
ENSG00000130024	PHF10	-0.613615016	0.000854446
ENSG00000139289	PHLDA1	-2.221942292	0.000855753
ENSG00000155827	RNF20	0.669528016	0.000859276
ENSG00000180917	CMTR2	0.760476344	0.000859616
ENSG00000109099	PMP22	-1.838299258	0.000859616
ENSG00000152256	PDK1	-0.693049817	0.000866111
ENSG00000137970	RPL7P9	-0.448387426	0.000871371
ENSG00000071242	RPS6KA2	-0.882373548	0.000871371
ENSG00000071462	WBSCR22	-0.455600095	0.000874122
ENSG00000205765	C5orf51	0.706774384	0.000877395
ENSG00000151065	DCP1B	0.763548342	0.000880076
ENSG00000267102		0.902218707	0.000881033
ENSG00000133121	STARD13	-1.892183647	0.000881427
ENSG00000105173	CCNE1	-1.230467594	0.000881915
ENSG00000180422	LINC00304	-1.495484368	0.000891161
ENSG00000125520	SLC2A4RG	-0.475698097	0.000893102
ENSG00000107282	APBA1	-1.313081527	0.000896928
ENSG00000069956	MAPK6	-1.056812301	0.000900227
ENSG00000188785	ZNF548	-0.584028283	0.000916573
ENSG00000166484	MAPK7	-0.608233711	0.000917422
ENSG00000145365	TIFA	1.010469623	0.000918278
ENSG00000151692	RNF144A	0.860448686	0.000920821
ENSG00000153767	GTF2E1	0.787214863	0.000920821
ENSG00000213203	GIMAP1	1.271800597	0.000924743
ENSG00000136158	SPRY2	-2.695449735	0.000924743
ENSG00000264247	LINC00909	0.582296252	0.000924743
ENSG00000005022	SLC25A5	-0.473894021	0.000927634
ENSG00000123095	BHLHE41	-3.018744279	0.000927634
ENSG00000125149	C16orf70	-0.324403793	0.000936368
ENSG00000114126	TFDP2	-0.51983228	0.000951987
ENSG00000131398	KCNC3	-0.907101678	0.000962088
ENSG00000250959	GLUD1P3	-0.679864207	0.000971482
ENSG00000170275	CRTAP	-0.401965428	0.000986032
ENSG00000019144	PHLDB1	-1.233928049	0.000987994
ENSG00000197272	IL27	1.517297463	0.000991351
ENSG00000217128	FNIP1	-0.729409442	0.000993379
ENSG00000219665		-0.556028562	0.000995326
ENSG00000121022	COPS5	0.443743237	0.001005794
ENSG00000104133	SPG11	0.476739099	0.00100747
ENSG00000140391	TSPAN3	-0.683726778	0.00100747
ENSG00000088826	SMOX	-1.89968936	0.001009907
ENSG00000146963	LUC7L2	-0.543136035	0.001012891
ENSG00000135469	COQ10A	-0.688085788	0.001012891
ENSG00000257093	KIAA1147	-0.54972848	0.00101516
ENSG00000123562	MORF4L2	-0.439129765	0.00101735
ENSG00000171049	FPR2	1.452303042	0.001027092
ENSG00000100422	CERK	-0.445472715	0.001027092

ENSG00000197279	ZNF165	-1.761980415	0.001031878
ENSG00000111716	LDHB	-0.477823687	0.001047689
ENSG00000116786	PLEKHM2	-0.60073958	0.00105791
ENSG00000164933	SLC25A32	-0.488959153	0.001058285
ENSG00000133103	COG6	0.505468791	0.001064513
ENSG00000101213	PTK6	0.930949624	0.001076405
ENSG00000197561	ELANE	1.376350969	0.001076405
ENSG00000135297	MTO1	0.581332808	0.001077131
ENSG00000163811	WDR43	-0.539315413	0.00108048
ENSG00000166886	NAB2	-1.530499477	0.001081394
ENSG00000070444	MNT	-0.675865847	0.001091473
ENSG00000169439	SDC2	-4.259772633	0.001093429
ENSG00000158669	GPAT4	-0.339264694	0.001094771
ENSG00000250182	EEF1A1P13	-0.628232724	0.001105663
ENSG00000162517	PEF1	0.425393853	0.001109924
ENSG00000157500	APPL1	0.425634044	0.001109924
ENSG00000129691	ASH2L	0.536818735	0.001109924
ENSG00000178202	KDELC2	0.762721399	0.001109924
ENSG00000125826	RBCK1	0.615426804	0.001109924
ENSG00000105135	ILVBL	-0.587168709	0.001109924
ENSG00000124103	FAM209A	-1.030997425	0.00111274
ENSG00000174080	CTSF	-1.048313878	0.0011172
ENSG00000128335	APOL2	0.856787768	0.0011172
ENSG00000064961	HMG20B	-0.47487092	0.001120428
ENSG00000182372	CLN8	-0.658905984	0.001121766
ENSG00000075391	RASAL2	-1.192013497	0.001127232
ENSG00000116883		-1.511997834	0.001134121
ENSG00000007944	MYLIP	-0.809367191	0.00113701
ENSG00000070756	PABPC1	-0.377539068	0.001144377
ENSG00000152147	GEMIN6	0.90137077	0.001158083
ENSG00000143322	ABL2	-0.508554195	0.001163089
ENSG00000132953	XPO4	-0.383135443	0.001163089
ENSG00000117394	SLC2A1	-0.761848025	0.001181968
ENSG00000156535	CD109	-1.822655014	0.001183654
ENSG00000119714	GPR68	-1.685719913	0.001184792
ENSG00000163166	IWS1	0.407905441	0.001185303
ENSG00000163464	CXCR1	1.854012177	0.001189675
ENSG00000159921	GNE	-0.43330716	0.001204356
ENSG00000143772	ITPKB	-0.432049661	0.001206639
ENSG00000114423	CBLB	-0.570091374	0.00121441
ENSG00000197343	ZNF655	-0.353369609	0.001241471
ENSG00000147140	NONO	-0.232742232	0.001243151
ENSG00000114982	KANSL3	-0.371761758	0.001245306
ENSG00000160789	LMNA	-1.819950417	0.001264951
ENSG00000022267	FHL1	-1.305068946	0.001264951
ENSG00000010404	IDS	-0.393210738	0.001264951
ENSG00000244165	P2RY11	-0.768019747	0.001272029
ENSG00000272367		-1.03514018	0.001285361

ENSG00000175352	NRIP3	-2.207187446	0.001290978
ENSG00000113312	TTC1	0.547392732	0.001293233
ENSG00000059691	GATB	0.593336028	0.001298333
ENSG00000143622	RIT1	-0.577800748	0.001298806
ENSG00000186468	RPS23	-0.386259313	0.001306387
ENSG00000099204	ABLIM1	-1.431707243	0.001315578
ENSG00000166582	CENPV	-1.192382512	0.001315578
ENSG00000073969	NSF	0.388039439	0.001315578
ENSG00000104814	MAP4K1	-0.550798338	0.001333933
ENSG00000154642	C21orf91	0.626782529	0.001333933
ENSG00000105793	GTPBP10	0.452614785	0.001336344
ENSG00000165898	ISCA2	0.618824577	0.001343971
ENSG00000112146	FBXO9	0.453498164	0.001360656
ENSG00000170542	SERPINB9	0.724667001	0.001361511
ENSG00000101187	SLCO4A1	-2.720257568	0.001361735
ENSG00000166123	GPT2	1.011548813	0.001365393
ENSG00000205846	CLEC6A	1.085465767	0.00136704
ENSG00000082516	GEMIN5	0.685484511	0.001370312
ENSG00000181467	RAP2B	-0.630428263	0.001373212
ENSG00000120860	CCDC53	0.604891082	0.001373212
ENSG00000180747	SMG1P3	-0.743595809	0.001373212
ENSG00000197566	ZNF624	0.803137893	0.001373212
ENSG00000159228	CBR1	1.002745593	0.001376735
ENSG00000266053	NDUFV2-AS1	-0.973932965	0.001381816
ENSG00000158373	HIST1H2BD	1.100422121	0.001383957
ENSG00000204619	PPP1R11	0.433403251	0.001383957
ENSG00000119383	PTPA	0.527811388	0.001383957
ENSG00000133884	DPF2	0.47490156	0.001383957
ENSG00000166579	NDEL1	-0.738023823	0.001383957
ENSG00000173065	FAM222B	-0.522863845	0.001402045
ENSG00000120833	SOCS2	-2.019377725	0.001425221
ENSG00000121060	TRIM25	0.618261981	0.001431688
ENSG00000173145	NOC3L	0.639078549	0.00143334
ENSG00000232472	EEF1B2P3	-0.5992394	0.001434122
ENSG00000135148	TRAFD1	0.869601981	0.001438844
ENSG00000272899		-1.519246156	0.001448549
ENSG00000205981	DNAJC19	0.569055062	0.00145506
ENSG00000197713	RPE	0.913021929	0.001485688
ENSG00000181284	TMEM102	0.878394958	0.001487092
ENSG00000237550	RPL9P9	-0.44735338	0.001487868
ENSG00000138375	SMARCAL1	0.634022228	0.001494783
ENSG00000163050	COQ8A	-0.398073839	0.001509736
ENSG00000144218	AFF3	-0.904581633	0.001518891
ENSG00000275111	ZNF2	1.256951591	0.001522456
ENSG00000272888	LINC01578	-0.549277646	0.001522703
ENSG00000148840	PPRC1	-0.520390533	0.001526429
ENSG00000168961	LGALS9	0.818247253	0.001526429
ENSG00000137309	HMGA1	-0.930714698	0.001526434

ENSG00000159479	MED8	0.514087846	0.001539389
ENSG00000239305	RNF103	-0.851279568	0.001539389
ENSG00000154065	ANKRD29	-1.95715992	0.001549441
ENSG00000151612	ZNF827	-2.253591244	0.00154998
ENSG00000178409	BEND3	-0.89701931	0.001552164
ENSG00000002587	HS3ST1	-3.008805433	0.001571953
ENSG00000164252	AGGF1	0.569976073	0.001589435
ENSG00000113368	LMNB1	1.297864941	0.001589435
ENSG00000139697	SBNO1	-0.308954423	0.001591807
ENSG00000197548	ATG7	0.490371174	0.00160687
ENSG00000280067		-1.019171967	0.001611687
ENSG00000179918	SEPHS2	0.642672415	0.001617053
ENSG00000178146		0.834389461	0.001625535
ENSG00000272941		0.949156805	0.001626683
ENSG00000196873	CBWD3	0.679897229	0.001638638
ENSG00000186166	CCDC84	-0.56568997	0.001638638
ENSG00000144843	ADPRH	0.840543649	0.001649275
ENSG00000198682	PAPSS2	-1.404597753	0.001656099
ENSG00000115421	PAPOLG	0.492905949	0.001669779
ENSG00000104290	FZD3	-1.021503905	0.001697901
ENSG00000101457	DNTTIP1	0.611726949	0.001697901
ENSG00000169155	ZBTB43	-0.902012578	0.001703492
ENSG00000151470	C4orf33	0.587556676	0.001716463
ENSG00000112419	PHACTR2	0.653629273	0.001722145
ENSG00000185482	STAC3	0.762572209	0.001722145
ENSG00000168386	FILIP1L	-1.424791854	0.001723347
ENSG00000240065	PSMB9	0.766967271	0.001738154
ENSG00000115317	HTRA2	0.472740871	0.001747398
ENSG00000184432	COPB2	0.731016808	0.001747398
ENSG00000152749	GPR180	0.890235212	0.001761127
ENSG00000152454	ZNF256	-0.878892329	0.001796186
ENSG00000182866	LCK	-1.472042556	0.00180276
ENSG00000102678	FGF9	-2.452433735	0.001818618
ENSG00000065978	YBX1	-0.356271045	0.001830965
ENSG00000172500	FIBP	0.44843236	0.00183207
ENSG00000124198	ARFGEF2	-0.396652744	0.001840387
ENSG00000008056	SYN1	-2.586175443	0.001884338
ENSG00000198019	FCGR1B	1.173608328	0.001890804
ENSG00000076716	GPC4	-1.5959933	0.00189146
ENSG00000142937	RPS8	-0.405359284	0.001896324
ENSG00000112685	EXOC2	0.3995941	0.001909865
ENSG00000175390	EIF3F	-0.323593766	0.001914783
ENSG00000117009	KMO	0.973774064	0.001915401
ENSG00000156671	SAMD8	-0.471733352	0.001915401
ENSG00000257242	LINC01619	-1.542005183	0.001915401
ENSG00000138623	SEMA7A	-1.37740608	0.001915401
ENSG00000101150	TPD52L2	-0.461200119	0.001916761
ENSG00000112033	PPARD	-0.549104566	0.001936739

ENSG00000132670	PTPRA	0.292647466	0.001941395
ENSG00000131263	RLIM	-0.658386329	0.001961048
ENSG00000123353	ORMDL2	0.65412774	0.001961092
ENSG00000099251	HSD17B7P2	1.086181995	0.001964812
ENSG00000197540	GZMM	-1.560272108	0.00197097
ENSG00000145431	PDGFC	-1.132217388	0.001976851
ENSG00000113532	ST8SIA4	0.556252089	0.001978767
ENSG00000140941	MAP1LC3B	-0.551219032	0.001978767
ENSG00000118900	UBN1	-0.317051554	0.001992637
ENSG00000166477	LEO1	0.667645751	0.002013906
ENSG00000204516	MICB	0.717911701	0.002043789
ENSG00000198455	ZXDB	-0.545110074	0.002057087
ENSG00000064651	SLC12A2	-0.652318238	0.002057444
ENSG00000005471	ABCB4	-2.158818941	0.002067146
ENSG00000242324		1.324755085	0.002067146
ENSG00000234444	ZNF736	-0.470181398	0.002071936
ENSG00000225032		-1.158091157	0.002071936
ENSG00000109184	DCUN1D4	-0.480659184	0.002073664
ENSG00000133112	TPT1	-0.373508402	0.002086916
ENSG00000155097	ATP6V1C1	0.441054363	0.002094667
ENSG00000198890	PRMT6	0.783825537	0.002111952
ENSG00000231721	LINC-PINT	-0.949654682	0.002111952
ENSG00000186642	PDE2A	-2.211339618	0.002111952
ENSG00000230551		-0.696209101	0.002113761
ENSG00000029363	BCLAF1	-0.457014873	0.00211579
ENSG00000214199	EEF1A1P12	-0.405441756	0.002132462
ENSG00000188522	FAM83G	-1.426268838	0.002133903
ENSG00000138795	LEF1	-1.705121542	0.002145146
ENSG00000085662	AKR1B1	-0.481464119	0.002148679
ENSG00000103489	XYLT1	-0.578515207	0.002148679
ENSG00000229299		-1.262747337	0.002148679
ENSG00000156381	ANKRD9	-0.917564001	0.002151057
ENSG00000167733	HSD11B1L	-0.735223028	0.002161375
ENSG00000136870	ZNF189	0.718656168	0.002179116
ENSG00000181523	SGSH	-0.533860181	0.002182772
ENSG00000188811	NHLRC3	0.727683633	0.002188172
ENSG00000228486	LINC01125	-0.727659466	0.002194321
ENSG00000104517	UBR5	-0.322859584	0.002194321
ENSG00000198734	F5	0.945549826	0.002196139
ENSG00000251022	THAP9-AS1	-0.550503054	0.002199729
ENSG00000155592	ZKSCAN2	-0.68618356	0.002199729
ENSG00000101096	NFATC2	-1.055769185	0.002201553
ENSG00000087903	RFX2	-0.782614828	0.002213087
ENSG00000181220	ZNF746	-0.439060526	0.002233386
ENSG00000107758	PPP3CB	0.339719388	0.002237911
ENSG00000147996	CBWD5	0.633264026	0.002241191
ENSG00000110079	MS4A4A	1.603026507	0.002245104
ENSG00000105808	RASA4	-0.718659743	0.002253892

ENSG00000156345	CDK20	-0.968509517	0.002253892
ENSG00000189079	ARID2	-0.496494458	0.002253892
ENSG00000260920		0.97796023	0.002259585
ENSG00000105705	SUGP1	0.413381891	0.002280656
ENSG00000135211	TMEM60	0.998917981	0.00231268
ENSG00000178429	RPS3AP5	-0.528079131	0.002321199
ENSG00000088298	EDEM2	0.35983172	0.002321199
ENSG00000163814	CDCP1	-2.046984228	0.002329915
ENSG00000154511	FAM69A	0.740266775	0.002337168
ENSG00000235245		-0.698754264	0.002338797
ENSG00000197043	ANXA6	0.758189956	0.002340164
ENSG00000143771	CNIH4	0.561687449	0.002353002
ENSG00000155463	OXA1L	-0.332314722	0.002364361
ENSG00000127452	FBXL12	-0.347490918	0.002364361
ENSG00000145779	TNFAIP8	0.691544159	0.002382236
ENSG00000185728	YTHDF3	-0.57907665	0.002382236
ENSG00000112406	HECA	-0.50456269	0.002386707
ENSG00000243811	APOBEC3D	0.900172025	0.002398436
ENSG00000063438	AHRR	-2.663526848	0.002404336
ENSG00000092330	TINF2	0.367548403	0.002405518
ENSG00000141503	MINK1	-0.39728031	0.002415639
ENSG00000171612	SLC25A33	-0.76273916	0.00242878
ENSG00000005302	MSL3	0.400123849	0.002436153
ENSG00000140931	CMTM3	-0.374850365	0.002436153
ENSG00000119878	CRIPT	0.536964804	0.002449795
ENSG00000134324	LPIN1	-0.463255715	0.002466378
ENSG00000038274	MAT2B	0.622065353	0.002466396
ENSG00000239521	GATS	-0.702756499	0.002466396
ENSG00000173726	TOMM20	-0.360701421	0.002468173
ENSG00000121067	SPOP	0.405001966	0.002475639
ENSG00000163159	VPS72	0.707506597	0.002517001
ENSG00000242689	CNTF	-0.946525195	0.002517377
ENSG00000145741	BTF3	-0.306689547	0.002541567
ENSG00000138413	IDH1	0.743664583	0.002545322
ENSG00000188282	RUFY4	1.339308965	0.002545322
ENSG00000170271	FAXDC2	-1.250922936	0.002547717
ENSG00000072818	ACAP1	0.503038547	0.002547717
ENSG00000148303	RPL7A	-0.342156243	0.002636741
ENSG00000163512	AZI2	0.431146161	0.002673478
ENSG00000095002	MSH2	0.870178884	0.002684521
ENSG00000065559	MAP2K4	0.332679557	0.002690452
ENSG00000048342	CC2D2A	-1.206130607	0.002693687
ENSG00000182742	HOXB4	1.424964527	0.002693687
ENSG00000129038	LOXL1	-2.389580353	0.002710877
ENSG00000163975	MELTF	-1.763904257	0.002722932
ENSG00000052802	MSMO1	0.577940857	0.002722932
ENSG00000151491	EPS8	-0.811184201	0.002722932
ENSG00000105186	ANKRD27	0.397796209	0.002722932

ENSG00000106263	EIF3B	-0.328586201	0.00273199
ENSG00000137642	SORL1	-0.675938827	0.00273199
ENSG00000183508	FAM46C	-1.873468474	0.002732891
ENSG00000116691	MIIP	0.446395452	0.002739519
ENSG00000175518	UBQLNL	1.688013065	0.002739519
ENSG00000225648	SBDSP1	-0.615524031	0.002740653
ENSG00000232613		-3.101347329	0.002742538
ENSG00000114270	COL7A1	-0.692185253	0.002742538
ENSG00000133985	TTC9	1.011320421	0.002742538
ENSG00000260911		-1.058791999	0.002742538
ENSG00000267701	NA	-1.385703261	0.002742538
ENSG00000104408	EIF3E	-0.357645225	0.002746011
ENSG00000128739	SNRPN	-0.583087158	0.002746011
ENSG00000119471	HSDL2	0.740598601	0.002756564
ENSG00000212802	RPL15P3	-0.471378113	0.00278304
ENSG00000196187	TMEM63A	0.693663309	0.002796133
ENSG00000204568	MRPS18B	0.746888019	0.002796133
ENSG00000133561	GIMAP6	1.258948028	0.002796133
ENSG00000277117		-1.90266652	0.002810758
ENSG00000035687	ADSS	0.390433351	0.00282683
ENSG00000178035	IMPDH2	-0.55879923	0.002832768
ENSG00000101574	METTL4	0.51907169	0.002850752
ENSG00000144741	SLC25A26	-0.428823486	0.002857066
ENSG00000077420	APBB1IP	0.488684342	0.002858359
ENSG00000106628	POLD2	-0.512006318	0.002882353
ENSG00000231607	DLEU2	0.752516441	0.002882353
ENSG00000253797	UTP14C	0.692443349	0.002882931
ENSG00000138760	SCARB2	0.5483248	0.002885948
ENSG00000111911	HINT3	0.551235273	0.002920095
ENSG00000120158	RCL1	-0.6376707	0.002920095
ENSG00000103353	UBFD1	0.557723342	0.002928257
ENSG00000138246	DNAJC13	0.546192067	0.002938018
ENSG00000110321	EIF4G2	-0.33140512	0.002949318
ENSG00000224032	EPB41L4A-AS1	-0.595186025	0.002959798
ENSG00000120254	MTHFD1L	-0.959522713	0.002959798
ENSG00000057757	PITHD1	-0.428899191	0.002981896
ENSG00000170606	HSPA4	0.403559175	0.002981896
ENSG00000033050	ABCF2	0.467389161	0.002981896
ENSG00000168259	DNAJC7	0.382558516	0.002981896
ENSG00000136717	BIN1	-0.901516662	0.002995912
ENSG00000121741	ZMYM2	-0.414711538	0.002995912
ENSG00000156508	EEF1A1	-0.395567064	0.003008415
ENSG00000168066	SF1	-0.389846469	0.003008415
ENSG00000151500	THYN1	0.630691037	0.003008415
ENSG00000242071	RPL7AP6	-0.360892463	0.003008415
ENSG00000185359	HGS	-0.402970899	0.003008415
ENSG00000132199	ENOSF1	-0.682445271	0.003008415
ENSG00000130749	ZC3H4	0.391554119	0.003008415

ENSG00000155876	RRAGA	0.436951518	0.003016512
ENSG00000152465	NMT2	-0.887883228	0.003016512
ENSG00000167461	RAB8A	0.504435874	0.003016512
ENSG00000169764	UGP2	0.595925211	0.003053978
ENSG00000105829	BET1	0.64074169	0.003062201
ENSG00000170677	SOCS6	-0.840702925	0.003063876
ENSG00000109084	TMEM97	-1.201365509	0.003071105
ENSG00000156711	MAPK13	-0.733256623	0.003090011
ENSG00000087263	OGFOD1	0.696571451	0.003090011
ENSG00000111196	MAGOHB	0.519461483	0.003090902
ENSG00000215271	HOMEZ	1.031949055	0.003102659
ENSG00000138594	TMOD3	0.57275193	0.003102659
ENSG00000131051	RBM39	-0.420589579	0.003114034
ENSG00000143155	TIPRL	0.433952927	0.003142887
ENSG00000085465	OVGP1	-1.008737082	0.003213631
ENSG00000112996	MRPS30	0.458430944	0.003213631
ENSG00000234797	RPS3AP6	-0.447115672	0.003213631
ENSG00000155093	PTPRN2	0.950989755	0.003217944
ENSG00000065491	TBC1D22B	0.324272694	0.003221871
ENSG00000032742	IFT88	-0.649316149	0.003302557
ENSG00000260708		-0.846713898	0.003320601
ENSG00000171115	GIMAP8	1.367493149	0.003327345
ENSG00000267040		0.73455108	0.003361249
ENSG00000151929	BAG3	-0.712147427	0.003365203
ENSG00000153561	RMND5A	-0.354876164	0.003368201
ENSG00000159164	SV2A	-1.245528172	0.003407143
ENSG00000146830	GIGYF1	-0.307077586	0.003407143
ENSG00000203814	HIST2H2BF	0.892231409	0.003471852
ENSG00000137492	THAP12	-0.357476833	0.003471852
ENSG00000161860	SYCE2	-1.856873737	0.003471852
ENSG00000109079	TNFAIP1	0.637721372	0.003500491
ENSG00000128272	ATF4	-0.458125728	0.003538844
ENSG00000105821	DNAJC2	-0.555727941	0.003589115
ENSG00000141994	DUS3L	-0.557466223	0.003594956
ENSG00000135828	RNASEL	0.944457289	0.003602617
ENSG00000090470	PDCD7	0.447409352	0.003607657
ENSG00000101224	CDC25B	-0.446356389	0.003626123
ENSG00000117318	ID3	-1.402232471	0.003626385
ENSG00000100266	PACSIN2	0.491889448	0.003626385
ENSG00000177733	HNRNPA0	-0.341285914	0.003646708
ENSG00000145675	PIK3R1	-0.799355782	0.003683009
ENSG00000102119	EMD	-0.681298894	0.003683229
ENSG00000270972		2.339624225	0.003683229
ENSG00000182809	CRIP2	-1.718237104	0.003683229
ENSG00000062485	CS	-0.310243883	0.003687181
ENSG00000110619	CARS	0.359987316	0.003696748
ENSG00000095139	ARCN1	0.472500928	0.003704925
ENSG00000100100	PIK3IP1	-0.757973921	0.003727115

ENSG00000075884	ARHGAP15	0.382479451	0.003728793
ENSG00000163738	MTHFD2L	-0.632850604	0.003728793
ENSG00000140992	PDPK1	-0.32678655	0.003728793
ENSG00000196428	TSC22D2	-0.567821406	0.003769818
ENSG00000177426	TGIF1	-0.928134831	0.003769818
ENSG00000226742	HSBP1L1	-0.754967928	0.00377588
ENSG00000025156	HSF2	-0.599702548	0.003797059
ENSG00000081059	TCF7	-1.502247146	0.003830239
ENSG00000167671	UBXN6	-0.348177671	0.00383292
ENSG00000115137	DNAJC27	-0.43848122	0.003864424
ENSG00000239857	GET4	-0.525152197	0.003864548
ENSG00000131931	THAP1	0.546022301	0.003864548
ENSG00000132357	CARD6	0.959201238	0.003887367
ENSG00000079215	SLC1A3	0.948336765	0.003895587
ENSG00000244486	SCARF2	-1.086773404	0.003910019
ENSG00000259330	INAFM2	-0.504622876	0.003926619
ENSG00000138138	ATAD1	0.415767959	0.003936665
ENSG00000037897	METTL1	-0.855166662	0.003965447
ENSG00000120063	GNA13	-0.797514915	0.003965447
ENSG00000240857	RDH14	0.552851645	0.00398134
ENSG00000100181	TPTEP1	-1.279376839	0.003991633
ENSG00000114120	SLC25A36	-0.472102694	0.003995881
ENSG00000142396	ERVK3-1	-0.512859788	0.003995881
ENSG00000226751		1.850187574	0.003995881
ENSG00000117143	UAP1	-0.870433256	0.003999211
ENSG00000280088		0.437007254	0.004009782
ENSG00000141378	PTRH2	0.527586739	0.004009782
ENSG00000172071	EIF2AK3	-0.863570188	0.004024549
ENSG00000169508	GPR183	-1.445374853	0.004032169
ENSG00000084093	REST	-0.368091238	0.004041988
ENSG00000279088		-0.620452528	0.004041988
ENSG00000144554	FANCD2	-0.577871221	0.004054148
ENSG00000089916	GPATCH2L	-0.407177448	0.004073434
ENSG00000131779	PEX11B	0.696371753	0.004115505
ENSG00000198755	RPL10A	-0.345006014	0.004120043
ENSG00000240849	TMEM189	-0.346427548	0.004132724
ENSG00000105127	AKAP8	-0.415689144	0.004133052
ENSG00000273559	CWC25	-0.526035563	0.004135911
ENSG00000228057	SEC63P1	-1.018442994	0.004150716
ENSG00000142655	PEX14	0.424557177	0.00415339
ENSG00000231113		-0.424773256	0.004185619
ENSG00000258944		-0.642554447	0.004212061
ENSG00000188554	NBR1	0.537043113	0.004248265
ENSG00000113790	EHHADH	1.02957952	0.004249646
ENSG00000143891	GALM	0.76978955	0.004262553
ENSG00000136802	LRRC8A	-0.426706411	0.004323039
ENSG00000184216	IRAK1	-0.46717127	0.004337294
ENSG00000129636	ITFG1	0.345955211	0.004337294

ENSG00000149932	TMEM219	0.288209132	0.004346287
ENSG00000095637	SORBS1	-1.364236544	0.004348138
ENSG00000158715	SLC45A3	-1.756009011	0.004364752
ENSG00000079785	DDX1	0.402876363	0.004365301
ENSG00000151893	CACUL1	-0.333772346	0.004421109
ENSG00000204257	HLA-DMA	-0.671203134	0.00445413
ENSG00000134014	ELP3	0.49249769	0.00445413
ENSG00000101413	RPRD1B	-0.696530138	0.00445413
ENSG00000123360	PDE1B	-0.980697582	0.004473053
ENSG00000130340	SNX9	-1.314108429	0.004479385
ENSG00000117533	VAMP4	0.546016805	0.004503412
ENSG00000105402	NAPA	0.479950895	0.004503412
ENSG00000048471	SNX29	-0.494968337	0.004541102
ENSG00000182118	FAM89A	0.776591098	0.004548564
ENSG00000235776		-0.403129559	0.004569934
ENSG00000086544	ITPKC	0.531837431	0.004578335
ENSG00000261067		-0.598860883	0.004592313
ENSG00000130561	SAG	-0.769700237	0.004636669
ENSG00000196498	NCOR2	-0.408760145	0.00464789
ENSG00000075292	ZNF638	0.481174779	0.004661128
ENSG00000115514	TXNDC9	0.648884912	0.004697421
ENSG00000131351	HAUS8	-0.617590196	0.004697421
ENSG00000274211	SOCS7	-0.430254139	0.004730424
ENSG00000160908	ZNF394	-0.633633542	0.004730995
ENSG00000204389	HSPA1A	0.996114962	0.004736582
ENSG00000053770	AP5M1	0.408331424	0.004736582
ENSG00000203395		-1.182160645	0.004738939
ENSG00000197380	DACT3	-1.211222538	0.004738939
ENSG00000162222	TTC9C	0.995410635	0.004741123
ENSG00000133216	EPHB2	1.536408503	0.004750334
ENSG00000184988	TMEM106A	0.813528041	0.004750334
ENSG00000141219	C17orf80	0.925637788	0.004750334
ENSG00000196313	POM121	-0.288348754	0.004750406
ENSG00000166313	APBB1	-0.812425969	0.00477059
ENSG00000169045	HNRNPH1	-0.332226899	0.004805989
ENSG00000129696	TTI2	0.536370621	0.00484213
ENSG00000138834	MAPK8IP3	-0.366391972	0.004843194
ENSG00000145495	Mar-06	-0.383230108	0.004856012
ENSG00000181896	ZNF101	0.555646753	0.004894174
ENSG00000169057	MECP2	-0.436106579	0.004909923
ENSG00000243335	KCTD7	-0.796191835	0.004919515
ENSG00000186767	SPIN4	-0.900213594	0.004924221
ENSG00000136840	ST6GALNAC4	-0.662363748	0.004924221
ENSG00000267194		0.999715344	0.004924221
ENSG00000263731		-0.645757012	0.004964164
ENSG00000065809	FAM107B	-0.539028823	0.004992053
ENSG00000172171	TEFM	0.619390759	0.005053308
ENSG00000119986	AVPI1	-1.715508023	0.005062354

ENSG00000170921	TANC2	-0.65379686	0.005062354
ENSG00000108604	SMARCD2	0.385815056	0.005062354
ENSG00000105193	RPS16	-0.366462054	0.005062354
ENSG00000074935	TUBE1	0.501642903	0.005079893
ENSG00000156011	PSD3	-1.141983907	0.005086192
ENSG00000170456	DENND5B	-0.932568281	0.005086192
ENSG00000148154	UGCG	-0.538806481	0.005094296
ENSG00000273173	SNURF	-2.217540928	0.005111438
ENSG00000179933	C14orf119	0.794324598	0.00511222
ENSG00000213261	EEF1B2P6	-1.038674193	0.0051182
ENSG00000115271	GCA	0.739496265	0.005168825
ENSG00000176641	RNF152	-2.261924743	0.005170812
ENSG00000120333	MRPS14	0.735598161	0.005197307
ENSG00000136448	NMT1	0.587891536	0.005218012
ENSG00000102575	ACP5	-0.726247964	0.005221542
ENSG00000135535	CD164	0.418337365	0.005244605
ENSG00000162139	NEU3	-0.436111378	0.005249626
ENSG00000116560	SFPQ	-0.450624477	0.005285127
ENSG00000165392	WRN	-0.508090059	0.005297533
ENSG00000140367	UBE2Q2	-0.414974025	0.005297533
ENSG00000250251	PKD1P6	-0.555179691	0.005297533
ENSG00000187583	PLEKHN1	0.995157948	0.00530671
ENSG00000144744	UBA3	0.481381467	0.00530671
ENSG00000280398		-1.24218104	0.00530671
ENSG00000103365	GGA2	-0.482086248	0.00530671
ENSG00000174871	CNIH2	-1.073034677	0.00531574
ENSG00000254685	FPGT	0.694235577	0.005325862
ENSG00000114956	DGUOK	0.296644351	0.005329528
ENSG00000251634		1.530203258	0.005329528
ENSG00000004766	VPS50	0.383330107	0.005329528
ENSG00000258875		-1.25041477	0.005369947
ENSG00000162664	ZNF326	-0.584624974	0.005415307
ENSG00000165637	VDAC2	-0.334719472	0.005420225
ENSG00000092067	CEBPE	1.049692247	0.005420225
ENSG00000260179		-0.990734441	0.005441017
ENSG00000146757	ZNF92	-0.646496131	0.005466108
ENSG00000258890	CEP95	-0.516769844	0.005481472
ENSG00000211668	IGLV2-11	-3.090378021	0.00549512
ENSG00000086102	NFX1	-0.497546651	0.005501067
ENSG00000162714	ZNF496	0.601448994	0.005509897
ENSG00000115524	SF3B1	-0.302908893	0.005509897
ENSG00000182568	SATB1	-0.605837848	0.005509897
ENSG00000103496	STX4	-0.497818001	0.005565844
ENSG00000173706	HEG1	0.648331689	0.005601333
ENSG00000164430	MB21D1	0.749612566	0.005606658
ENSG00000142546	NOSIP	0.55327582	0.005628744
ENSG00000028116	VRK2	0.471576161	0.005657054
ENSG00000176155	CCDC57	-0.350473698	0.005668479

ENSG00000198363	ASPH	-0.80004184	0.005679244
ENSG00000100387	RBX1	0.454776249	0.005679733
ENSG00000096717	SIRT1	-0.566389559	0.00568568
ENSG00000172828	CES3	-1.07716118	0.00568568
ENSG00000063180	CA11	-0.774703867	0.005720175
ENSG00000231925	TAPBP	0.349117523	0.005734262
ENSG00000105708	ZNF14	-0.707494272	0.005748332
ENSG00000107262	BAG1	0.347251125	0.005781448
ENSG00000140853	NLRC5	0.427735217	0.005814087
ENSG00000123136	DDX39A	-0.518246929	0.005823587
ENSG00000164104	HMGB2	0.586157999	0.005826264
ENSG00000154310	TNIK	0.684416319	0.005833838
ENSG00000143947	RPS27A	-0.358229086	0.005874523
ENSG00000139211	AMIGO2	0.671162168	0.005874523
ENSG00000167522	ANKRD11	-0.291400579	0.005874523
ENSG00000147457	CHMP7	0.553792076	0.005879205
ENSG00000231025	NA	-0.390402367	0.005888426
ENSG00000198677	TTC37	0.369855754	0.005902078
ENSG00000204099	NEU4	-2.993766429	0.00590791
ENSG00000213085	CFAP45	-1.362197238	0.005924911
ENSG00000126262	FFAR2	1.244244867	0.005924911
ENSG00000100425	BRD1	-0.604378712	0.005924911
ENSG00000144468	RHBDD1	0.384446412	0.005928463
ENSG00000166822	TMEM170A	-0.325968286	0.005928463
ENSG00000116754	SRSF11	-0.331662641	0.005944418
ENSG00000163069	SGCB	-0.75797434	0.006010373
ENSG00000112137	PHACTR1	-1.659830782	0.006085466
ENSG00000214783		-0.900235444	0.006085514
ENSG00000173801	JUP	1.40050464	0.006096912
ENSG00000176624	MEX3C	-0.505350944	0.006113141
ENSG00000187193	MT1X	0.811502077	0.006126881
ENSG00000169682	SPNS1	-0.530827012	0.006144655
ENSG00000244945		-1.138333755	0.006153577
ENSG00000254521	SIGLEC12	2.062237778	0.006153577
ENSG00000130517	PGPEP1	-0.505299159	0.006158499
ENSG00000049759	NEDD4L	-0.61833592	0.006167465
ENSG00000185404	SP140L	0.475448775	0.006176314
ENSG00000158186	MRAS	-0.985298402	0.006176314
ENSG00000163960	UBXN7	-0.373557167	0.006176314
ENSG00000106635	BCL7B	-0.534213873	0.006176314
ENSG00000147677	EIF3H	-0.23475146	0.006176314
ENSG00000140153	WDR20	-0.314328779	0.006181292
ENSG00000131558	EXOC4	0.491375214	0.006204808
ENSG00000139514	SLC7A1	-0.742072689	0.006211784
ENSG00000237940		-0.934675342	0.006279192
ENSG00000172936	MYD88	0.433265576	0.006314639
ENSG00000198218	QRICH1	-0.319064079	0.006314639
ENSG00000244005	NFS1	0.449698464	0.006314639

ENSG00000139370	SLC15A4	0.64431983	0.006323004
ENSG00000169371	SNUPN	0.482440001	0.006327888
ENSG00000100647	SUSD6	-0.335353982	0.006354977
ENSG00000273038		-1.36446766	0.006363872
ENSG00000117091	CD48	0.345558491	0.006404225
ENSG00000204315	FKBPL	0.982817143	0.006404225
ENSG00000112110	MRPL18	0.629604924	0.006404225
ENSG00000134575	ACP2	0.520967547	0.006404225
ENSG00000067057	PFKP	0.533079489	0.006418017
ENSG00000198301	SDAD1	0.435980877	0.006453446
ENSG00000124782	RREB1	-0.323654434	0.006453446
ENSG00000147168	IL2RG	0.406207749	0.006462908
ENSG00000198668	CALM1	0.392599644	0.006462908
ENSG00000020633	RUNX3	-0.649457449	0.006492187
ENSG00000203710	CR1	0.890272328	0.006492187
ENSG00000137806	NDUFAF1	0.76567439	0.006492187
ENSG00000089639	GMIP	0.270232922	0.006492187
ENSG00000070476	ZXDC	-0.276739323	0.006586063
ENSG00000103671	TRIP4	0.450119598	0.006602879
ENSG00000125779	PANK2	0.490713033	0.006605745
ENSG00000225828	FAM229A	-0.468384966	0.006608517
ENSG00000129116	PALLD	-1.117418915	0.006608517
ENSG00000262528		-0.933071539	0.006610113
ENSG00000171204	TMEM126B	0.458516949	0.00662034
ENSG00000100911	PSME2	0.521288919	0.006626801
ENSG00000174574	AKIRIN1	-0.320501246	0.00663208
ENSG00000181163	NPM1	-0.331979103	0.006645034
ENSG00000175550	DRAP1	0.552334676	0.006645034
ENSG00000140406	MESDC1	-0.629453814	0.00667554
ENSG00000132326	PER2	-0.823907868	0.006685867
ENSG00000161202	DVL3	-0.381516088	0.006748785
ENSG00000274943		-1.028371141	0.006768992
ENSG00000136485	DCAF7	0.630624909	0.006768992
ENSG00000154710	RABGEF1	-0.898492609	0.006785451
ENSG00000229754	CXCR2P1	1.70987682	0.006857107
ENSG00000077514	POLD3	0.693416471	0.006857107
ENSG00000111674	ENO2	-1.447008061	0.006870145
ENSG00000168237	GLYCTK	0.414668706	0.006879233
ENSG00000148229	POLE3	-0.397883651	0.006879233
ENSG00000246100	LINC00900	-1.183314972	0.006879233
ENSG00000100804	PSMB5	0.52485759	0.006879233
ENSG00000171497	PPID	0.459303006	0.006885191
ENSG00000006634	DBF4	-0.826507354	0.006885191
ENSG00000139567	ACVRL1	1.073481706	0.006885191
ENSG00000123836	PFKFB2	-0.636721554	0.006897326
ENSG00000135336	ORC3	0.457259274	0.006932821
ENSG00000122482	ZNF644	-0.443447853	0.00696676
ENSG00000168993	CPLX1	-2.794725464	0.00696676

ENSG00000157933	SKI	-0.577433559	0.006974959
ENSG00000119844	AFTPH	0.416343601	0.006981491
ENSG00000204217	BMPR2	0.47535105	0.00701143
ENSG00000204160	ZDHHC18	-0.343957698	0.0070219
ENSG00000163877	SNIP1	-0.776925784	0.007024228
ENSG00000227345	PARG	0.392048469	0.007057764
ENSG00000113761	ZNF346	0.542375262	0.007083275
ENSG00000170881	RNF139	-0.443427011	0.007108989
ENSG00000095397	WHRN	-1.258378992	0.007111994
ENSG00000185101	ANO9	-1.465364334	0.007111994
ENSG00000083635	NUFIP1	-0.624158305	0.007111994
ENSG00000114857	NKTR	-0.391024314	0.007148284
ENSG00000134644	PUM1	0.270884298	0.007152408
ENSG00000137500	CCDC90B	0.374120906	0.00715338
ENSG00000278864		-1.013063444	0.007154587
ENSG00000112303	VNN2	0.540578974	0.007223292
ENSG00000160602	NEK8	0.566476006	0.007238506
ENSG00000185753	CXorf38	0.556653198	0.007249285
ENSG00000204628	RACK1	-0.271909915	0.007350765
ENSG00000137817	PARP6	-0.313575325	0.007401721
ENSG00000163870	TPRA1	-0.792006276	0.007412809
ENSG00000157593	SLC35B2	-0.599545516	0.007415582
ENSG00000147403	RPL10	-0.349829086	0.007448887
ENSG00000185085	INTS5	0.742620433	0.007448887
ENSG00000137752	CASP1	0.631293426	0.007448887
ENSG00000175197	DDIT3	-0.90100809	0.007448887
ENSG00000119431	HDHD3	0.679148654	0.00745723
ENSG00000171262	FAM98B	0.716497362	0.007459158
ENSG00000180228	PRKRA	-0.326004434	0.007460649
ENSG00000176225	RTTN	-0.456910107	0.007475541
ENSG00000085231	AK6	0.544412458	0.007487801
ENSG00000149809	TM7SF2	-0.538699981	0.007525097
ENSG00000188529	SRSF10	-0.434248664	0.007563363
ENSG00000140807	NKD1	1.233724958	0.00758644
ENSG00000105722	ERF	-0.576947878	0.00758644
ENSG00000139163	ETNK1	0.516094283	0.007616156
ENSG00000106610	STAG3L4	-0.428683335	0.007623706
ENSG00000205808	PLPP6	0.964355791	0.007644434
ENSG00000269318		-1.178182003	0.007677121
ENSG00000144848	ATG3	0.433121761	0.007724529
ENSG00000105373	GLTSCR2	-0.389688994	0.007744356
ENSG00000179051	RCC2	-0.329498269	0.007778274
ENSG00000128284	APOL3	0.603843182	0.007778274
ENSG00000239557		-0.865559034	0.007801648
ENSG00000174327	SLC16A13	0.908652078	0.007801648
ENSG00000224470	ATXN1L	-0.517587353	0.007832762
ENSG00000168404	MLKL	0.689418162	0.007841578
ENSG00000182670	TTC3	-0.467301702	0.007905316

ENSG00000006704	GTF2IRD1	-1.532415753	0.007927114
ENSG00000104412	EMC2	0.459316077	0.007927114
ENSG00000204713	TRIM27	0.694329014	0.007954744
ENSG00000095209	TMEM38B	-0.569408975	0.0079627
ENSG00000004455	AK2	0.453083729	0.007988757
ENSG00000165355	FBXO33	-0.677448985	0.007988757
ENSG00000125952	MAX	0.319146789	0.00799144
ENSG00000129351	ILF3	-0.352154832	0.00799144
ENSG00000178700	DHFR2	0.855561913	0.007994493
ENSG00000110057	UNC93B1	0.494228505	0.008027609
ENSG00000086827	ZW10	0.677287112	0.008052886
ENSG00000144306	SCRN3	0.568366155	0.008082109
ENSG00000178127	NDUFV2	-0.848697106	0.00808596
ENSG00000255198	SNHG9	-0.585482553	0.008097207
ENSG00000104823	ECH1	0.470772207	0.008126122
ENSG00000261438		1.172591661	0.008140711
ENSG00000106723	SPIN1	-0.348618896	0.008164994
ENSG00000272047	GTF2H5	0.450985627	0.008175599
ENSG00000091129	NRCAM	-3.024302008	0.008199952
ENSG00000106399	RPA3	0.466299913	0.008202241
ENSG00000091732	ZC3HC1	0.398695686	0.008204437
ENSG00000028310	BRD9	-0.270845366	0.008212617
ENSG00000146072	TNFRSF21	-2.198826464	0.008234652
ENSG00000278740		-1.827269448	0.008247285
ENSG00000185418	TARSL2	-0.48121706	0.00824743
ENSG00000047579	DTNBP1	0.422815436	0.008253783
ENSG00000143845	ETNK2	-2.682277132	0.008254699
ENSG00000075188	NUP37	0.633313312	0.008264258
ENSG00000159708	LRRC36	-1.063043861	0.008264258
ENSG00000162402	USP24	-0.347743016	0.008293746
ENSG00000138073	PREB	0.623848643	0.008373587
ENSG00000117139	KDM5B	-0.496434231	0.00837567
ENSG00000168056	LTBP3	-0.717381812	0.008376262
ENSG00000247853		1.070975403	0.008420772
ENSG00000077380	DYNC1I2	0.596898507	0.008467845
ENSG00000187815	ZFP69	0.647831988	0.008502705
ENSG00000197021	CXorf40B	-0.480413194	0.008516244
ENSG00000259408		-0.910479905	0.008516244
ENSG00000102317	RBM3	-0.335360091	0.00853863
ENSG00000182584	ACTL10	1.260766808	0.008539241
ENSG00000008128	CDK11A	-0.494094753	0.008551978
ENSG00000183496	MEX3B	-1.139155318	0.008556313
ENSG00000109519	GRPEL1	-0.388683272	0.008624469
ENSG00000185100	ADSSL1	-0.617725868	0.008642763
ENSG00000136213	CHST12	0.64795469	0.008738581
ENSG00000109062	SLC9A3R1	-0.602428679	0.008744899
ENSG00000108946	PRKAR1A	0.462731826	0.008756258
ENSG00000139687	RB1	0.434396005	0.008773943

ENSG00000279549		-0.859289043	0.008833802
ENSG00000173457	PPP1R14B	-0.731435393	0.008833802
ENSG00000150337	FCGR1A	1.211071648	0.008875534
ENSG00000198026	ZNF335	-0.283195661	0.008875534
ENSG00000124641	MED20	0.760162398	0.008876664
ENSG00000177565	TBL1XR1	-0.469048301	0.009054239
ENSG00000254858	MPV17L2	-0.600213453	0.009068432
ENSG00000145945	FAM50B	0.832261192	0.009078325
ENSG00000038382	TRIO	-0.539720477	0.009125796
ENSG00000168016	TRANK1	0.682928547	0.009132193
ENSG00000161653	NAGS	0.925107721	0.009144376
ENSG00000115457	IGFBP2	1.481886829	0.009149893
ENSG00000100068	LRP5L	-0.552160339	0.009149893
ENSG00000231528	FAM225A	1.220437677	0.009191262
ENSG00000162777	DENND2D	0.778766002	0.009241897
ENSG00000145390	USP53	-1.204775386	0.009245362
ENSG00000096968	JAK2	0.548986531	0.009268721
ENSG00000100372	SLC25A17	0.460522789	0.0092832
ENSG00000153560	UBP1	0.345028817	0.009317584
ENSG00000197147	LRRC8B	-0.615159877	0.009322388
ENSG00000235725		-0.718634839	0.009325558
ENSG00000113263	ITK	-1.050194582	0.009330516
ENSG00000102760	RGCC	-1.435156318	0.009341918
ENSG00000099834	CDHR5	1.298712698	0.009352147
ENSG00000120686	UFM1	-0.284504998	0.009375994
ENSG00000134882	UBAC2	0.322924845	0.009386229
ENSG00000179409	GEMIN4	-0.410793182	0.009399867
ENSG00000213609	RPL7AP50	-0.909015854	0.009421707
ENSG00000132196	HSD17B7	0.631485767	0.009426284
ENSG00000204209	DAXX	0.670188983	0.009472685
ENSG00000166747	AP1G1	-0.497675827	0.009472685
ENSG00000175643	RMI2	1.431612797	0.009514466
ENSG00000186469	GNG2	-0.477545054	0.009532333
ENSG00000118496	FBXO30	-0.576723397	0.009567051
ENSG00000174010	KLHL15	-0.679360378	0.009661858
ENSG00000145780	FEM1C	-0.747152598	0.009691069
ENSG00000144749	LRIG1	-0.587848604	0.009697198
ENSG00000111875	ASF1A	0.645695143	0.009697198
ENSG00000278231		-0.6997846	0.009697198
ENSG00000143376	SNX27	0.482786415	0.009788204
ENSG00000237063		-1.48372325	0.009794862
ENSG00000102524	TNFSF13B	0.965208442	0.009847153
ENSG00000133466	C1QTNF6	-0.727627648	0.009847153
ENSG00000273216		-1.216175178	0.009889719
ENSG00000100664	EIF5	-0.472451271	0.009904268
ENSG00000249992	TMEM158	-2.37050781	0.009913004
ENSG00000173812	EIF1	-0.369588335	0.009917353
ENSG00000101945	SUV39H1	-0.568441761	0.009941305

ENSG00000165688	PMPCA	-0.295800822	0.009941305
ENSG00000065427	KARS	0.320291728	0.009965087
ENSG00000048405	ZNF800	-0.464089494	0.010007767
ENSG00000183647	ZNF530	-0.557820525	0.010011142
ENSG00000187626	ZKSCAN4	0.830452673	0.010033703
ENSG00000072422	RHOBTB1	-1.338649265	0.010033703
ENSG00000015479	MATR3	-0.640899689	0.010082307
ENSG00000010278	CD9	-1.522380008	0.010088354
ENSG00000152464	RPP38	0.447056634	0.010115254
ENSG00000119599	DCAF4	-0.578034756	0.010133434
ENSG00000155189	AGPAT5	0.653363719	0.010135705
ENSG00000198517	MAFK	-0.583097787	0.010144413
ENSG00000272106		-0.418522363	0.010157658
ENSG00000232119	MCTS1	0.49497145	0.010157658
ENSG00000170439	METTL7B	1.789405787	0.010157658
ENSG00000144597	EAF1	-0.715351769	0.010176749
ENSG00000151882	CCL28	-0.807333765	0.01023727
ENSG00000167333	TRIM68	0.623735924	0.01023727
ENSG00000123700	KCNJ2	1.65275555	0.01023727
ENSG00000100426	ZBED4	-0.300306997	0.01023727
ENSG00000176024	ZNF613	0.998190702	0.010246726
ENSG00000235750	KIAA0040	0.831901698	0.010248203
ENSG00000184371	CSF1	-1.629885219	0.010278565
ENSG00000139725	RHOF	-0.402502424	0.010278565
ENSG00000157350	ST3GAL2	0.408521934	0.010278565
ENSG00000139291	TMEM19	0.453262504	0.010295546
ENSG00000261490		-0.642723177	0.010304765
ENSG00000281205	LINC00950	-0.887529522	0.010322056
ENSG00000112079	STK38	0.48621714	0.010402968
ENSG00000130396	AFDN	-0.963852482	0.010417731
ENSG00000112078	KCTD20	-0.319183335	0.010431422
ENSG00000183665	TRMT12	0.548340864	0.010530936
ENSG00000151490	PTPRO	0.691981945	0.010551545
ENSG00000140450	ARRDC4	-0.956157033	0.010576656
ENSG00000091157	WDR7	0.366367055	0.010576656
ENSG00000227467	LINC01537	-1.124694812	0.010578054
ENSG00000118257	NRP2	-1.106177452	0.010661371
ENSG00000115275	MOGS	0.405759171	0.010747452
ENSG00000110987	BCL7A	-0.877960362	0.010750973
ENSG00000101247	NDUFAF5	-0.669208234	0.01076466
ENSG00000124181	PLCG1	-0.822056331	0.01076466
ENSG00000078061	ARAF	-0.297340064	0.010765021
ENSG00000112394	SLC16A10	-1.026965917	0.010772781
ENSG00000175606	TMEM70	-0.510251453	0.010772781
ENSG00000143498	TAF1A	0.801410434	0.010783983
ENSG00000279192	PWAR5	-1.023472312	0.010799938
ENSG00000204611	ZNF616	0.937894905	0.01085223
ENSG00000106560	GIMAP2	0.715697116	0.010863459

ENSG00000117859	OSBPL9	0.329513527	0.010922631
ENSG00000184678	HIST2H2BE	0.645713919	0.010929579
ENSG00000105576	TNPO2	-0.269337665	0.010929579
ENSG00000165685	TMEM52B	-1.376651884	0.010943869
ENSG00000111913	FAM65B	0.677645283	0.010947176
ENSG00000139793	MBNL2	-0.532411004	0.010947176
ENSG00000215021	PHB2	-0.238456254	0.011030923
ENSG00000152128	TMEM163	-2.723328171	0.011040311
ENSG00000196470	SIAH1	-0.608044219	0.011040311
ENSG00000149646	CNBD2	-0.587721586	0.011052374
ENSG00000136827	TOR1A	0.453389956	0.01114214
ENSG00000274677		-1.103887622	0.011217764
ENSG00000135047	CTSL	-2.178937835	0.011237565
ENSG00000224861	YBX1P1	-0.494580126	0.011255946
ENSG00000241878	PISD	0.330863577	0.011268269
ENSG00000093072	CECR1	0.507576421	0.011278599
ENSG00000172575	RASGRP1	-1.301316005	0.01132234
ENSG00000120616	EPC1	-0.306413322	0.011395791
ENSG00000205302	SNX2	0.348213872	0.011481776
ENSG00000108448	TRIM16L	-0.84121242	0.011533686
ENSG00000188981	MSANTD1	-0.744405338	0.011533869
ENSG00000118777	ABCG2	-2.444714128	0.011536587
ENSG00000130844	ZNF331	-1.743897304	0.011617529
ENSG00000138686	BBS7	0.564508275	0.011621882
ENSG00000134900	TPP2	-0.342898367	0.011621882
ENSG00000140993	TIGD7	0.827536126	0.011621882
ENSG00000279207		-0.836447548	0.011621882
ENSG00000169093	ASMTL	-0.435371444	0.01164282
ENSG00000204136	GGTA1P	-1.223767944	0.01164282
ENSG00000196659	TTC30B	0.89732534	0.011718918
ENSG00000197093	GAL3ST4	-1.1693339	0.011718918
ENSG00000146833	TRIM4	0.453707808	0.011735899
ENSG00000104518	GSDMD	0.590236684	0.011750986
ENSG00000196696		-0.407097022	0.011750986
ENSG00000041988	THAP3	0.560838981	0.011789126
ENSG00000013288	MAN2B2	0.320791342	0.011789126
ENSG00000081087	OSTM1	0.422499163	0.011789126
ENSG00000156603	MED19	0.558642716	0.011789126
ENSG00000152380	FAM151B	0.543876658	0.011813668
ENSG00000204475	NCR3	-2.498914882	0.011826822
ENSG00000067369	TP53BP1	-0.380611487	0.01192734
ENSG00000132581	SDF2	0.302887083	0.01192734
ENSG00000183458		-0.856682685	0.011929794
ENSG00000125746	EML2	0.371447771	0.011935647
ENSG00000112308	C6orf62	0.30896216	0.011970858
ENSG00000115020	PIKFYVE	-0.356137488	0.01199274
ENSG00000119138	KLF9	-0.742522499	0.012099882
ENSG00000196531	NACA	-0.245553903	0.012099882

ENSG00000170604	IRF2BP1	0.504883852	0.012099882
ENSG00000100483	VCPKMT	-0.475667155	0.012100588
ENSG00000137955	RABGGTB	-0.365197339	0.012145751
ENSG00000277476		-0.537037296	0.012170216
ENSG00000174233	ADCY6	-0.830295385	0.012191169
ENSG00000251867		-0.74901416	0.012202945
ENSG00000008710	PKD1	-0.447959481	0.012202945
ENSG00000139921	TMX1	0.52794344	0.012204141
ENSG00000277449	CEBPB-AS1	-0.797850142	0.012204141
ENSG00000181315	ZNF322	0.920967052	0.012229476
ENSG00000089280	FUS	-0.355957161	0.012231404
ENSG00000196345	ZKSCAN7	1.007529486	0.0122611
ENSG00000279117		-0.406535152	0.012270469
ENSG00000214485	RPL7P1	-0.608025118	0.012301001
ENSG00000219545	UMAD1	0.475322536	0.012306459
ENSG00000143575	HAX1	0.328578358	0.012327035
ENSG00000232573	RPL3P4	-0.418728312	0.01237718
ENSG00000183161	FANCF	0.79330166	0.012401158
ENSG00000276533		0.713304183	0.012410119
ENSG00000140948	ZCCHC14	-0.590063404	0.012447972
ENSG00000196365	LONP1	-0.38267872	0.012447972
ENSG00000162889	MAPKAPK2	-0.587050588	0.012454704
ENSG00000165244	ZNF367	-1.002459455	0.012461018
ENSG00000153944	MSI2	-0.318068756	0.012461018
ENSG00000197063	MAFG	-0.544762284	0.012486792
ENSG00000100403	ZC3H7B	0.284880562	0.012486792
ENSG00000277496		0.827818263	0.012508718
ENSG00000100644	HIF1A	-0.88007474	0.012556536
ENSG00000168502	MTCL1	-2.142307464	0.012604929
ENSG00000205581	HMGN1	-0.368462064	0.012690892
ENSG00000166716	ZNF592	-0.293002161	0.012692246
ENSG00000154124	OTULIN	-0.327974652	0.012748744
ENSG00000165533	TTC8	-0.562079816	0.012748744
ENSG00000127152	BCL11B	-1.647259628	0.012779145
ENSG00000177352	CCDC71	0.562554646	0.012842984
ENSG00000146425	DYNLT1	0.714478222	0.012842984
ENSG00000268043	NBPF12	-0.355930753	0.012863886
ENSG00000105202	FBL	-0.267343811	0.012898705
ENSG00000164056	SPRY1	-1.454047617	0.012936938
ENSG00000173295	FAM86B3P	-0.706379014	0.012936938
ENSG00000101298	SNPH	-1.216655006	0.012936938
ENSG00000184787	UBE2G2	-0.341447667	0.012936938
ENSG00000162408	NOL9	-0.448479554	0.012966884
ENSG00000244560		-0.716927664	0.012966884
ENSG00000272760		0.734128768	0.012966884
ENSG00000153902	LGI4	-1.311523116	0.012966884
ENSG00000276337		-1.258948073	0.012971689
ENSG00000273658		-1.212536159	0.012983829

ENSG00000132471	WBP2	0.229871493	0.012988148
ENSG00000170260	ZNF212	-0.502397834	0.013038051
ENSG00000275769		-2.783833153	0.013038051
ENSG00000158427	TMSB15B	-1.06668155	0.013066616
ENSG00000187514	PTMA	-0.298954347	0.013087953
ENSG00000227066		-1.020536245	0.01310982
ENSG00000107833	NPM3	-0.608106035	0.01310982
ENSG00000167978	SRRM2	-0.332279508	0.01310982
ENSG00000108469	RECQL5	0.442638305	0.01310982
ENSG00000259891		-0.7009997	0.013127815
ENSG00000152409	JMY	-0.882788541	0.013132208
ENSG00000079134	THOC1	-0.334198976	0.013132208
ENSG00000270681		-1.419972593	0.013162596
ENSG00000137364	TPMT	0.453440486	0.013162596
ENSG00000143653	SCCPDH	0.562189773	0.013198334
ENSG00000112200	ZNF451	-0.362848586	0.013198334
ENSG00000165233	CARD19	-0.402622244	0.013198334
ENSG00000167778	SPRYD3	0.572969903	0.013198334
ENSG00000184990	SIVA1	-0.356671344	0.013198334
ENSG00000150593	PDCD4	-0.483243738	0.013204388
ENSG00000136450	SRSF1	0.448100213	0.013204388
ENSG00000196557	CACNA1H	-2.727503936	0.013207141
ENSG00000119812	FAM98A	0.492063613	0.013250187
ENSG00000025708	TYMP	1.096598581	0.01332707
ENSG00000100342	APOL1	0.903648657	0.013351339
ENSG00000253982		-0.658066466	0.013360943
ENSG00000115364	MRPL19	0.561757287	0.013366095
ENSG00000101882	NKAP	0.388363916	0.013366095
ENSG00000133195	SLC39A11	0.494487776	0.013366095
ENSG00000066379	ZNRD1	0.445468328	0.013381335
ENSG00000167103	PIP5KL1	-1.239645214	0.013381335
ENSG00000166130	IKBIP	0.665234519	0.013381335
ENSG00000182400	TRAPPC6B	0.622586916	0.013381335
ENSG00000187775	DNAH17	-1.864960928	0.013381335
ENSG00000173818	ENDOV	-0.427237977	0.013381335
ENSG00000100393	EP300	-0.357179355	0.013381335
ENSG00000146054	TRIM7	0.677035115	0.013506502
ENSG00000131849	ZNF132	1.22111034	0.013517549
ENSG00000198815	FOXJ3	-0.273636812	0.013529208
ENSG00000196338	NLGN3	-1.01818573	0.013529208
ENSG00000196233	LCOR	-0.366372468	0.013529208
ENSG00000278991		-1.07672584	0.013529208
ENSG00000076604	TRAF4	-0.905454061	0.013529208
ENSG00000181894	ZNF329	-0.554832158	0.013529208
ENSG00000075415	SLC25A3	-0.408499397	0.013534613
ENSG00000261338		-0.629625597	0.013570525
ENSG00000141002	TCF25	-0.342872017	0.013570525
ENSG00000143553	SNAPIN	0.536914722	0.013653919

ENSG00000165410	CFL2	-0.774062258	0.013662343
ENSG00000169967	MAP3K2	-0.439452057	0.013703788
ENSG00000054967	RELT	-0.609656103	0.013767028
ENSG00000100815	TRIP11	0.562917154	0.013800363
ENSG00000180257	ZNF816	0.653206163	0.013800363
ENSG00000162396	PARS2	1.245998393	0.013850874
ENSG00000152520	PAN3	-0.287241218	0.013850874
ENSG00000214706	IFRD2	-0.385211141	0.013957369
ENSG00000162636	FAM102B	-0.53908749	0.01396362
ENSG00000114209	PDCD10	0.410498599	0.013976783
ENSG00000147526	TACC1	-0.474504481	0.013976783
ENSG00000256087	ZNF432	-0.417329495	0.013976783
ENSG00000160211	G6PD	0.371185381	0.013986122
ENSG00000177989	ODF3B	1.009484678	0.014039564
ENSG00000196776	CD47	0.283130588	0.014079474
ENSG00000156928	MALSU1	0.48104448	0.014079758
ENSG00000165406	Mar-08	-0.354790928	0.014079758
ENSG00000107566	ERLIN1	0.631271191	0.014079758
ENSG00000201457	SNORA55	-1.032818426	0.014086115
ENSG00000115085	ZAP70	-1.171039532	0.014153528
ENSG00000033867	SLC4A7	-0.452615794	0.014153528
ENSG00000211445	GPX3	-1.280226968	0.014153528
ENSG00000205352	PRR13	0.271583641	0.014202401
ENSG00000260841	NA	-0.480733308	0.014204917
ENSG00000106443	PHF14	0.35178097	0.014204917
ENSG00000125772	GPCPD1	-0.503125267	0.014204917
ENSG00000163435	ELF3	-0.584877662	0.014243064
ENSG00000145723	GIN1	0.85850129	0.014285957
ENSG00000101138	CSTF1	0.443844066	0.014297384
ENSG00000149823	VPS51	-0.296110127	0.014319643
ENSG00000178053	MLF1	-1.419331224	0.014330984
ENSG00000113621	TXNDC15	0.461729007	0.014330984
ENSG00000170234	PWWP2A	0.405766737	0.014330984
ENSG00000109787	KLF3	-0.524085349	0.014352391
ENSG00000147475	ERLIN2	0.318722922	0.014352391
ENSG00000092208	GEMIN2	0.45546431	0.014352391
ENSG00000126775	ATG14	-0.352099688	0.014352391
ENSG00000172116	CD8B	-1.5979435	0.014407037
ENSG00000056050	HPF1	0.699403302	0.014407037
ENSG00000172508	CARNS1	-1.569932352	0.014407037
ENSG00000279133		-0.728822529	0.01440922
ENSG00000141985	SH3GL1	-0.449249752	0.01440922
ENSG00000254503		1.164722053	0.01440922
ENSG00000167635	ZNF146	0.504505586	0.014438476
ENSG00000169914	OTUD3	-0.383907543	0.014587647
ENSG00000198286	CARD11	-1.184279797	0.014587647
ENSG00000131196	NFATC1	-0.31592961	0.014587647
ENSG00000105617	LENG1	0.498513414	0.014668496

ENSG00000073111	MCM2	-1.217496771	0.014680098
ENSG00000146834	MEPCE	-0.527876321	0.014737818
ENSG00000115165	CYTIP	-0.551921761	0.014783838
ENSG00000108433	GOSR2	0.346541467	0.014797495
ENSG00000165782	TMEM55B	-0.363227789	0.014829124
ENSG00000280434		-0.655165894	0.0148399
ENSG00000135249	RINT1	-0.369045954	0.014861567
ENSG00000197265	GTF2E2	0.35479877	0.014861567
ENSG00000103269	RHBDL1	-1.089234066	0.014861567
ENSG00000169715	MT1E	1.923426191	0.014861567
ENSG00000141034	GID4	0.58593624	0.014861567
ENSG00000165792	METTL17	0.449008687	0.014906342
ENSG00000164292	RHOBTB3	-1.188525264	0.014949876
ENSG00000006576	PHTF2	-0.332669249	0.014967093
ENSG00000099364	FBXL19	0.382539547	0.015011333
ENSG00000162438	CTRC	-0.894067243	0.015034065
ENSG00000168026	TTC21A	0.755254097	0.015056522
ENSG00000172530	BANP	-0.307086928	0.015087612
ENSG00000136244	IL6	2.590540282	0.01512651
ENSG00000269892		-0.911853643	0.015157579
ENSG00000186594	MIR22HG	-0.864102324	0.015157579
ENSG00000112787	FBRSL1	-0.343267063	0.015184556
ENSG00000175866	BAIAP2	-0.524368835	0.015184556
ENSG00000128602	SMO	-1.864855503	0.015188495
ENSG00000082153	BZW1	-0.478794844	0.015203847
ENSG00000144711	IQSEC1	-0.391513738	0.015203847
ENSG00000247240	UBL7-AS1	-0.556858484	0.015223055
ENSG00000177628	GBA	0.496043523	0.015233323
ENSG00000107882	SUFU	0.420421845	0.015283168
ENSG00000279432		-0.836283367	0.015299171
ENSG00000187257	RSBN1L	0.48697984	0.015333163
ENSG00000100401	RANGAP1	0.389883935	0.015333163
ENSG00000164080	RAD54L2	-0.291551856	0.015356081
ENSG00000279838		-0.787304177	0.015368636
ENSG00000085721	RRN3	-0.493160874	0.015368636
ENSG00000180423	HARBI1	0.601878338	0.015536786
ENSG00000100554	ATP6V1D	0.572694094	0.01554846
ENSG00000092929	UNC13D	0.340610142	0.01554846
ENSG00000204439	C6orf47	0.526600757	0.015572591
ENSG00000155792	DEPTOR	-1.398433478	0.015572591
ENSG00000141076	UTP4	-0.392375392	0.015674991
ENSG00000141622	RNF165	1.589739775	0.015683446
ENSG00000008513	ST3GAL1	-0.431940148	0.01569281
ENSG00000111727	HCFC2	0.456527358	0.015701377
ENSG00000183506	PI4KAP2	-0.476137755	0.015701377
ENSG00000259494	MRPL46	0.504004711	0.015811742
ENSG00000088854	C20orf194	-0.39912825	0.015811742
ENSG00000118961	LDAH	0.602369807	0.015819751

ENSG00000182903	ZNF721	0.545309845	0.015819751
ENSG00000175137	SH3BP5L	0.476062016	0.015822188
ENSG00000172086	KRCC1	0.664240453	0.015838878
ENSG00000110367	DDX6	-0.376656154	0.015839532
ENSG00000185864	NPIPB4	-0.503199348	0.015839532
ENSG00000265531	FCGR1CP	1.559938605	0.015942793
ENSG00000121966	CXCR4	-0.721284059	0.015946357
ENSG00000180346	TIGD2	0.531875458	0.015955515
ENSG00000198938	MT-CO3	-0.395812053	0.015966379
ENSG00000163704	PRRT3	-0.786552177	0.016048822
ENSG00000153140	CETN3	0.852668901	0.016048822
ENSG00000213024	NUP62	0.40494672	0.016048893
ENSG00000181274	FRAT2	0.507284178	0.016066377
ENSG00000129484	PARP2	-0.491812875	0.016066377
ENSG00000181826	RELL1	-0.809059904	0.016088239
ENSG00000136699	SMPD4	-0.382473078	0.016115153
ENSG00000073464	CLCN4	1.161370092	0.016127645
ENSG00000001084	GCLC	-0.323747123	0.016133034
ENSG00000272918		-0.838623919	0.016133034
ENSG00000111319	SCNN1A	-0.721269728	0.016166711
ENSG00000158417	EIF5B	0.362855097	0.01618331
ENSG00000181218	HIST3H2A	-1.062291012	0.016201031
ENSG00000164967	RPP25L	0.683096893	0.016201031
ENSG00000111011	RSRC2	-0.467810704	0.016201031
ENSG00000279800	BCLAF1P2	-0.768592816	0.016201031
ENSG00000204642	HLA-F	0.407509896	0.016202203
ENSG00000224691		-0.692950384	0.016204193
ENSG00000075142	SRI	0.304042721	0.016211066
ENSG00000120875	DUSP4	-2.801239695	0.016211066
ENSG00000125844	RRBP1	0.509592582	0.016211066
ENSG00000070413	DGCR2	0.326921561	0.016211066
ENSG00000052723	SIKE1	0.432385534	0.016239577
ENSG00000236383	LINC00854	-1.266444916	0.016239577
ENSG00000165118	C9orf64	0.919741941	0.016293021
ENSG00000083845	RPS5	-0.354023185	0.016314733
ENSG00000164169	PRMT9	-0.587412348	0.016322974
ENSG00000139131	YARS2	0.465530113	0.016322974
ENSG00000158792	SPATA2L	-0.705919474	0.016394691
ENSG00000184203	PPP1R2	-0.36902068	0.016416068
ENSG00000102316	MAGED2	0.33833894	0.016416068
ENSG00000103769	RAB11A	0.321496808	0.016416068
ENSG00000100650	SRSF5	-0.3209159	0.016506382
ENSG00000206337	HCP5	0.664002815	0.016509227
ENSG00000130203	APOE	-1.091477713	0.01654814
ENSG00000172059	KLF11	-0.580138614	0.016631185
ENSG00000114166	KAT2B	0.429569703	0.016662525
ENSG00000259820		-0.734705549	0.016669246
ENSG00000148090	AUH	0.439576971	0.016669246

ENSG00000269952		-2.270043005	0.016669246
ENSG00000167775	CD320	-0.737987358	0.016669246
ENSG00000116273	PHF13	-0.992391385	0.016718381
ENSG00000185127	C6orf120	0.400926239	0.016747397
ENSG00000141497	ZMYND15	-0.970023821	0.016821327
ENSG00000076382	SPAG5	-1.03583025	0.01685969
ENSG00000116918	TSNAX	0.440886285	0.01688591
ENSG00000141096	DPEP3	-0.833567185	0.01688591
ENSG00000104885	DOT1L	-0.567206574	0.01688591
ENSG00000131876	SNRPA1	-0.389797323	0.017032617
ENSG00000121104	FAM117A	0.508247772	0.017054741
ENSG00000178732	GP5	-1.670959392	0.017196185
ENSG00000196418	ZNF124	-0.414075236	0.017198039
ENSG00000102710	SUPT20H	0.483292986	0.017198039
ENSG00000100811	YY1	-0.207518234	0.017260482
ENSG00000261685	NA	1.491706019	0.017260482
ENSG00000141376	BCAS3	0.375653053	0.017421961
ENSG00000157045	NTAN1	-0.60119083	0.017436953
ENSG00000136504	KAT7	-0.406352366	0.017453075
ENSG00000184047	DIABLO	-0.634296834	0.017610596
ENSG00000100567	PSMA3	0.40580712	0.017610596
ENSG00000015285	WAS	0.258870058	0.017631433
ENSG00000073670	ADAM11	-0.961329301	0.017631433
ENSG00000125691	RPL23	-0.310335703	0.017639457
ENSG00000113638	TTC33	0.544562689	0.017646198
ENSG00000185024	BRF1	-0.309783019	0.017646198
ENSG00000245869		-2.616566371	0.017656779
ENSG00000241769	LINC00893	-0.515825387	0.017682945
ENSG00000068878	PSME4	-0.45453494	0.017683265
ENSG00000213862		-0.370541885	0.017683265
ENSG00000177335	C8orf31	0.970464927	0.017695275
ENSG00000253276	CCDC71L	-0.732688117	0.017719143
ENSG00000120159	CAAP1	0.289611932	0.017719143
ENSG00000196139	AKR1C3	-1.517936562	0.017823028
ENSG00000207110	RNU1-106P	-1.622779758	0.017839783
ENSG00000031691	CENPQ	0.864436863	0.017857698
ENSG00000176386	CDC26	0.352377835	0.017857698
ENSG00000140612	SEC11A	-0.309039911	0.017908005
ENSG00000169583	CLIC3	-2.705800289	0.018009952
ENSG00000116337	AMPD2	-0.477601392	0.018027739
ENSG00000142959	BEST4	-1.417811575	0.018040328
ENSG00000013306	SLC25A39	-0.286106099	0.018071353
ENSG00000183340	JRKL	0.738512202	0.018090103
ENSG00000074416	MGLL	-1.363793381	0.018108106
ENSG00000176407	KCMF1	-0.265092854	0.018167301
ENSG00000141971	MVB12A	0.440618437	0.018167301
ENSG00000266094	RASSF5	-0.337812271	0.018193693
ENSG00000136720	HS6ST1	-0.547600452	0.018234834

ENSG00000167491	GATAD2A	-0.271224717	0.018234834
ENSG00000269688		-0.582097789	0.018234834
ENSG00000273951		-1.029545713	0.01824163
ENSG00000118816	CCNI	-0.237801867	0.018256937
ENSG00000266962		0.680920342	0.018288538
ENSG00000213757		-0.564540219	0.018301577
ENSG00000167468	GPX4	-0.408200023	0.018310337
ENSG00000109065	NAT9	-0.429676626	0.018317588
ENSG00000171566	PLRG1	0.358582197	0.018364614
ENSG00000173991	TCAP	-0.712846571	0.018364614
ENSG00000177946	CENPBD1	0.715300498	0.018394013
ENSG00000088451	TGDS	0.56890934	0.018416028
ENSG00000259768		-1.013017853	0.018425453
ENSG00000178921	PFAS	-0.410135021	0.018441262
ENSG00000222937		-0.9341341	0.018462057
ENSG00000169223	LMAN2	0.384806715	0.0184917
ENSG00000161714	PLCD3	-0.496516052	0.018512165
ENSG00000158411	MITD1	0.624908717	0.018539502
ENSG00000236552	RPL13AP5	-0.34226092	0.018673763
ENSG00000151729	SLC25A4	-1.866841968	0.018733117
ENSG00000169641	LUZP1	-0.351038842	0.018739195
ENSG00000165949	IFI27	2.320325941	0.018773545
ENSG00000205268	PDE7A	0.425407236	0.018808594
ENSG00000151623	NR3C2	-0.922658593	0.018830467
ENSG00000254470	AP5B1	0.474571829	0.018830467
ENSG00000175582	RAB6A	0.319108684	0.018830467
ENSG00000101608	MYL12A	0.347208281	0.018830467
ENSG00000137331	IER3	-1.029027976	0.018846681
ENSG00000165417	GTF2A1	0.386868689	0.018900135
ENSG00000171155	C1GALT1C1	0.735623055	0.018934083
ENSG00000171608	PIK3CD	0.348353855	0.018941258
ENSG00000123066	MED13L	-0.407014276	0.01895409
ENSG00000139880	CDH24	-1.67539365	0.01895409
ENSG00000151702	FLI1	0.530637635	0.018983657
ENSG00000187676	B3GLCT	-0.461585486	0.019029083
ENSG00000108179	PPIF	-1.017125132	0.019053388
ENSG00000166974	MAPRE2	-0.266570637	0.019053388
ENSG00000248578	NPM1P21	-1.024446412	0.019115827
ENSG00000168297	PXK	0.33275745	0.019159152
ENSG00000151117	TMEM86A	-0.621612366	0.019165043
ENSG00000158270	COLEC12	-0.666440333	0.019165043
ENSG00000188419	CHM	0.356730362	0.019177252
ENSG00000157184	CPT2	0.570198494	0.019183087
ENSG00000164168	TMEM184C	0.359351343	0.019284442
ENSG00000196205	EEF1A1P5	-0.399105169	0.019284442
ENSG00000139180	NDUFA9	0.298971052	0.019284442
ENSG00000117697	NSL1	0.517642071	0.019310721
ENSG00000163312	HELQ	0.373691113	0.019310721

ENSG00000161395	PGAP3	-0.315618328	0.019310721
ENSG00000100997	ABHD12	-0.535997114	0.019310721
ENSG00000198814	GK	0.436026434	0.019401741
ENSG00000122692	SMU1	0.299079564	0.019420665
ENSG00000103018	CYB5B	0.484426125	0.019466041
ENSG00000178175	ZNF366	1.190809817	0.01947524
ENSG00000186105	LRRC70	-1.100009569	0.019522782
ENSG00000147471	PROSC	0.515760823	0.019591339
ENSG00000173230	GOLGB1	-0.321492999	0.019624597
ENSG00000119661	DNAL1	-0.398996421	0.019665708
ENSG00000157426	AASDH	0.517129099	0.019675039
ENSG00000247121		0.661638409	0.019684567
ENSG00000177479	ARIH2	-0.186122565	0.01977908
ENSG00000092096	SLC22A17	-2.60208744	0.01977908
ENSG00000157379	DHRS1	0.373563806	0.019797041
ENSG00000173875	ZNF791	-0.39314879	0.019828106
ENSG00000134369	NAV1	-0.683233996	0.019897792
ENSG00000234284	ZNF879	0.64586156	0.020069463
ENSG00000107937	GTPBP4	-0.480423599	0.020069463
ENSG00000170425	ADORA2B	-1.706511879	0.020069733
ENSG00000157191	NECAP2	0.386255306	0.020118242
ENSG00000130363	RSPH3	0.562413485	0.020126425
ENSG00000116809	ZBTB17	-0.414597622	0.020153725
ENSG00000186446	ZNF501	0.965879132	0.020156299
ENSG00000248275	TRIM52-AS1	-0.471480154	0.020156299
ENSG00000180992	MRPL14	0.41408436	0.020216891
ENSG00000101558	VAPA	-0.380920413	0.02022725
ENSG00000159596	TMEM69	0.521688007	0.020233855
ENSG00000187866	FAM122A	0.472488007	0.020233855
ENSG00000187624	C17orf97	2.130628705	0.020233855
ENSG00000114391	RPL24	-0.271198819	0.020269009
ENSG00000182473	EXOC7	0.249715879	0.020327167
ENSG00000166170	BAG5	-0.317726586	0.020382778
ENSG00000119321	FKBP15	0.510974894	0.020384951
ENSG00000138111	MFSD13A	0.574886224	0.020429119
ENSG00000088833	NSFL1C	0.326316343	0.020429119
ENSG00000213997	PGAM1P7	-2.21309187	0.020464326
ENSG00000111605	CPSF6	-0.301334396	0.020464326
ENSG00000206077	ZDHHC11B	-2.618992322	0.020482588
ENSG00000142173	COL6A2	-1.79095673	0.020482588
ENSG00000260774		-1.069450473	0.020494511
ENSG00000009413	REV3L	-0.333215416	0.020494511
ENSG00000109917	ZPR1	-0.339361333	0.020494511
ENSG00000136315		0.766664802	0.020494511
ENSG00000113194	FAF2	-0.288549321	0.020503567
ENSG00000156502	SUPV3L1	-0.564641129	0.020514345
ENSG00000205423	CNEP1R1	-0.404255672	0.020514432
ENSG00000170871	KIAA0232	-0.301810408	0.020601014

ENSG00000170027	YWHAG	0.573746765	0.020601014
ENSG00000166233	ARIH1	-0.41217038	0.020691743
ENSG00000067177	PHKA1	-1.229407724	0.020710192
ENSG00000102805	CLN5	0.525327911	0.020725826
ENSG00000161513	FDXR	0.679523345	0.020725826
ENSG00000156256	USP16	-0.44131167	0.020725826
ENSG00000143207	RFWD2	0.234044751	0.020737935
ENSG00000079332	SAR1A	-0.467551207	0.020737935
ENSG00000196387	ZNF140	0.411230208	0.020737935
ENSG00000069345	DNAJA2	0.275110674	0.020741048
ENSG00000100883	SRP54	0.406563763	0.020746166
ENSG00000225855	RUSC1-AS1	0.752496087	0.020760376
ENSG00000143633	C1orf131	0.409208278	0.020760376
ENSG00000269929		-0.834823707	0.020760376
ENSG00000134453	RBM17	-0.243963419	0.020760376
ENSG00000205659	LIN52	0.453779367	0.020820274
ENSG00000113360	DROSHA	0.340658186	0.020836986
ENSG00000069329	VPS35	0.425849386	0.020857591
ENSG00000186432	KPNA4	-0.288310839	0.020890966
ENSG00000116514	RNF19B	0.551486564	0.020905019
ENSG00000254901	BORCS8	0.440056124	0.020919707
ENSG00000143183	TMCO1	0.405759558	0.021009694
ENSG00000112367	FIG4	0.509480081	0.021009694
ENSG00000224596	ZMIZ1-AS1	-1.497825871	0.021009694
ENSG00000101337	TM9SF4	0.276242925	0.021009694
ENSG00000267796	LIN37	0.495393277	0.021009694
ENSG00000163872	YEATS2	0.283766269	0.021084019
ENSG00000132170	PPARG	-1.893252982	0.02110117
ENSG00000132467	UTP3	0.440011152	0.02110117
ENSG00000198408	MGEA5	-0.248404764	0.02110117
ENSG00000277589		-0.955567191	0.021148408
ENSG00000124356	STAMBP	0.405090848	0.021175472
ENSG00000112182	BACH2	-1.130464225	0.021180941
ENSG00000135439	AGAP2	0.504029867	0.021201289
ENSG00000137818	RPLP1	-0.336800552	0.021201289
ENSG00000130640	TUBGCP2	-0.24410695	0.021226663
ENSG00000140598	EFL1	0.37408397	0.021228401
ENSG00000114062	UBE3A	-0.345604533	0.021242539
ENSG00000127993	RBM48	-0.495433642	0.0212457
ENSG00000130734	ATG4D	-0.403996989	0.0212457
ENSG00000058056	USP13	-0.457445449	0.021248832
ENSG00000118217	ATF6	0.308472568	0.021256562
ENSG00000155545	MIER3	-0.4490495	0.021265933
ENSG00000163568	AIM2	1.344082769	0.021292802
ENSG00000164620	RELL2	0.612961146	0.021302466
ENSG00000071205	ARHGAP10	-0.722831525	0.021361324
ENSG00000240230	COX19	-0.267039542	0.021361324
ENSG00000230673	PABPC1P3	-1.002525544	0.021363196

ENSG00000196664	TLR7	1.101928589	0.021379262
ENSG00000163599	CTLA4	-2.178263584	0.021385236
ENSG00000172785	CBWD1	0.394417969	0.021402331
ENSG00000101654	RNMT	-0.546687576	0.021467019
ENSG00000168894	RNF181	0.401094468	0.021479942
ENSG00000041802	LSG1	-0.4162874	0.021479942
ENSG00000198888	MT-ND1	-0.398498691	0.021479942
ENSG00000205213	LGR4	-0.774479102	0.021490578
ENSG00000214389	RPS3AP26	-0.376833181	0.021493562
ENSG00000142541	RPL13A	-0.28957975	0.021569963
ENSG00000153130	SCOC	0.550316826	0.02167541
ENSG00000139675	HNRNPA1L2	-0.529448267	0.02167541
ENSG00000184451	CCR10	-1.217533483	0.02167541
ENSG00000196704	AMZ2	-0.347083637	0.02167541
ENSG00000169385	RNASE2	1.241545217	0.021682847
ENSG00000119013	NDUFB3	0.486258774	0.021705212
ENSG00000164117	FBXO8	0.48110913	0.021705212
ENSG00000197170	PSMD12	-0.464694453	0.021766819
ENSG00000187801	ZFP69B	0.743902167	0.021785285
ENSG00000105053	VRK3	0.323867275	0.021787551
ENSG00000013297	CLDN11	-0.669836342	0.021809272
ENSG00000279738		-0.516017051	0.021847684
ENSG00000105851	PIK3CG	-0.421734063	0.021919486
ENSG00000170632	ARMC10	0.449098915	0.021984546
ENSG00000169193	CCDC126	0.649092566	0.02199144
ENSG00000164144	ARFIP1	0.567571865	0.02201764
ENSG00000145632	PLK2	-1.241959776	0.02201764
ENSG00000278869		0.919738243	0.02207143
ENSG00000171453	POLR1C	-0.447139717	0.022094203
ENSG00000180573	HIST1H2AC	0.788502375	0.022198293
ENSG00000132773	TOE1	-0.525841403	0.022227068
ENSG00000136541	ERMN	-1.102525456	0.022305211
ENSG00000115520	COQ10B	-0.499709545	0.022312718
ENSG00000185839		0.679622288	0.022327724
ENSG00000140988	RPS2	-0.317461985	0.022327724
ENSG00000101193	GID8	-0.225897044	0.022330218
ENSG00000188305	C19orf35	1.348891479	0.022335458
ENSG00000167595	PROSER3	-0.3782278	0.022335458
ENSG00000105464	GRIN2D	0.514558638	0.022356099
ENSG00000223725		-0.812367236	0.022545798
ENSG00000135315	CEP162	0.519174322	0.022545798
ENSG00000272391	POM121C	-0.265144567	0.022545798
ENSG00000232187	FTH1P7	-0.672972284	0.022545798
ENSG00000155959	VBP1	0.3981992	0.022683588
ENSG00000166839	ANKDD1A	-1.291713553	0.022683588
ENSG00000146410	MTFR2	-1.0115123	0.022713833
ENSG00000104626	ERI1	0.448061077	0.022713833
ENSG00000163932	PRKCD	0.400200345	0.022714955

ENSG00000205726	ITSN1	0.52336164	0.022714955
ENSG00000188997	KCTD21	0.765791477	0.022724652
ENSG00000019995	ZRANB1	-0.366256244	0.022724652
ENSG00000182774	RPS17	-0.295880729	0.022777165
ENSG00000173221	GLRX	0.63629033	0.02282667
ENSG00000122557	HERPUD2	0.253590503	0.022957692
ENSG00000133874	RNF122	0.479239266	0.022957692
ENSG00000013364	MVP	0.340141533	0.022970899
ENSG00000176087	SLC35A4	0.346051007	0.022985359
ENSG00000117899	MESDC2	0.393788444	0.023012047
ENSG00000108239	TBC1D12	-0.661752119	0.023063219
ENSG00000113273	ARSB	0.354678537	0.023112697
ENSG00000146676	PURB	-0.465334907	0.023112697
ENSG00000120896	SORBS3	-0.52014262	0.023112697
ENSG00000125971	DYNLRB1	0.345618945	0.023112697
ENSG00000130348	QRSL1	0.506518894	0.023132118
ENSG00000102738	MRPS31	0.758228773	0.023174473
ENSG00000132155	RAF1	-0.211023508	0.023222519
ENSG00000123411	IKZF4	-0.467913854	0.023222519
ENSG00000162735	PEX19	0.277669731	0.023255297
ENSG00000122547	EEPD1	-0.705965223	0.023255297
ENSG00000118162	KPTN	0.548741734	0.023297651
ENSG00000255328		1.369051989	0.023329756
ENSG00000175262	C1orf127	-0.706589533	0.023495234
ENSG00000136842	TMOD1	1.383668339	0.023495234
ENSG00000140859	KIFC3	-1.26741219	0.023495234
ENSG00000125629	INSIG2	0.459492111	0.023542334
ENSG00000146842	TMEM209	0.423612446	0.023542334
ENSG00000138835	RGS3	0.855667684	0.023542334
ENSG00000163946	FAM208A	0.364706287	0.023550027
ENSG00000183431	SF3A3	-0.320324731	0.02363545
ENSG00000156860	FBRS	-0.362880135	0.023685765
ENSG00000164916	FOXK1	-0.354941799	0.023705842
ENSG00000160191	PDE9A	1.120258958	0.023707822
ENSG00000197696	NMB	-0.704113916	0.023718043
ENSG00000179115	FARSA	0.514515264	0.023718043
ENSG00000198467	TPM2	-0.647104822	0.023739607
ENSG00000177272	KCNA3	0.618896655	0.023956789
ENSG00000168214	RBPJ	-0.405724104	0.023986452
ENSG00000162650	ATXN7L2	-0.408080182	0.024025353
ENSG00000272636	DOC2B	-1.268083011	0.024075722
ENSG00000102878	HSF4	-0.521359852	0.024094552
ENSG00000275700	AATF	0.2766232	0.024121993
ENSG00000237296	SMG1P1	-0.550841726	0.024144141
ENSG00000100889	PCK2	0.55739065	0.024262385
ENSG00000229119		-0.701493551	0.024327632
ENSG00000101082	SLA2	-1.009456552	0.024355116
ENSG00000271976		0.673771471	0.024379757

ENSG00000163682	RPL9	-0.350746234	0.024384186
ENSG00000009307	CSDE1	-0.256954189	0.024612346
ENSG00000113441	LNPEP	0.361939856	0.024612346
ENSG00000148842	CNNM2	-0.459341286	0.024612346
ENSG00000138660	AP1AR	0.458831962	0.024641909
ENSG00000230207	RPL4P5	-0.418145803	0.024641909
ENSG00000181191	PJA1	0.415429543	0.024653772
ENSG00000165832	TRUB1	0.479650463	0.024653772
ENSG00000104177	MYEF2	-0.797859257	0.024653772
ENSG00000278311	GGNBP2	-0.358103322	0.024722274
ENSG00000213928	IRF9	0.348157723	0.024759535
ENSG00000153029	MR1	0.310943142	0.024761525
ENSG00000228903	RASA4CP	-0.514962978	0.024761525
ENSG00000174125	TLR1	0.559602298	0.024766902
ENSG00000126062	TMEM115	0.39084013	0.024822345
ENSG00000123349	PFDN5	-0.271430006	0.02486348
ENSG00000251417		-0.621119789	0.02486348
ENSG00000100823	APEX1	-0.294555944	0.02487951
ENSG00000107863	ARHGAP21	-0.530504992	0.024944584
ENSG00000274104		-0.632243527	0.02498281
ENSG00000196247	ZNF107	0.504961762	0.02510789
ENSG00000119397	CNTRL	0.418885224	0.02510789
ENSG00000113384	GOLPH3	0.320425559	0.025140179
ENSG00000160293	VAV2	-0.458190075	0.025140179
ENSG00000175354	PTPN2	0.229756156	0.025140179
ENSG00000124422	USP22	-0.263524063	0.025229455
ENSG00000164615	CAMLG	-0.272765501	0.025314596
ENSG00000278535	DHRS11	-0.652487715	0.025314596
ENSG00000196497	IPO4	-0.562927872	0.025349808
ENSG00000094880	CDC23	0.383285113	0.025536936
ENSG00000123610	TNFAIP6	1.424234372	0.025605474
ENSG00000226314	ZNF192P1	1.087709613	0.025636939
ENSG00000108021	FAM208B	-0.344585059	0.025636939
ENSG00000130005	GAMT	-0.938484288	0.025636939
ENSG00000137168	PPIL1	0.686970109	0.025661973
ENSG00000164051	CCDC51	0.566130309	0.025676093
ENSG00000188389	PDCD1	-1.953211853	0.025689771
ENSG00000148680	HTR7	-0.890837831	0.025689771
ENSG00000107719	PALD1	-1.670577392	0.025764563
ENSG00000122026	RPL21	-0.310778751	0.025794745
ENSG00000185122	HSF1	-0.213300943	0.025801434
ENSG00000176946	THAP4	-0.345401549	0.025839184
ENSG00000108666	C17orf75	0.362735547	0.025855212
ENSG00000105321	CCDC9	-0.540761326	0.02588555
ENSG00000064607	SUGP2	-0.26531027	0.025943907
ENSG00000198315	ZKSCAN8	-0.438919762	0.025983877
ENSG00000155265	GOLGA7B	-1.716421516	0.025987505
ENSG00000269858	EGLN2	-0.314913981	0.025997166

ENSG00000109381	ELF2	-0.317797947	0.026000377
ENSG00000113448	PDE4D	-1.072962291	0.026005266
ENSG00000266677		-0.67371878	0.026005266
ENSG00000163735	CXCL5	-2.600151247	0.026066841
ENSG00000182307	C8orf33	-0.279259188	0.026066841
ENSG00000141837	CACNA1A	1.203234089	0.026066841
ENSG00000257815		-0.501876584	0.026077939
ENSG00000143158	MPC2	-0.276519624	0.026088966
ENSG00000013441	CLK1	-0.507476583	0.026088966
ENSG00000113732	ATP6V0E1	0.387757413	0.026088966
ENSG00000111726	CMAS	0.400901302	0.026122911
ENSG00000204438	GPANK1	0.425195795	0.026350651
ENSG00000259834		0.485590811	0.026368849
ENSG00000267896	NA	-0.414558615	0.026443441
ENSG00000277916		-0.897174086	0.026506114
ENSG00000171863	RPS7	-0.249433943	0.026572158
ENSG00000277687		-0.591521533	0.026572158
ENSG00000162543	UBXN10	-1.372160057	0.026626892
ENSG00000171631	P2RY6	1.054444279	0.026626892
ENSG00000171456	ASXL1	-0.24031222	0.026626892
ENSG00000124920	MYRF	-1.291243572	0.026640077
ENSG00000096060	FKBP5	0.761525972	0.026658305
ENSG00000139112	GABARAPL1	-1.184924212	0.026665079
ENSG00000163257	DCAF16	-0.380003496	0.026705042
ENSG00000016864	GLT8D1	0.550054565	0.026784655
ENSG00000273272		0.974585173	0.026784655
ENSG00000138669	PRKG2	-2.329019701	0.026870125
ENSG00000261269		1.171418616	0.026870125
ENSG00000065665	SEC61A2	-0.544848296	0.026889249
ENSG00000167904	TMEM68	0.486023683	0.026919308
ENSG00000156886	ITGAD	-1.999947611	0.026950767
ENSG00000186063	AIDA	0.461306594	0.026964856
ENSG00000186660	ZFP91	-0.310424503	0.027003573
ENSG00000265100		-0.886260984	0.027009618
ENSG00000128654	MTX2	0.365727671	0.027073782
ENSG00000103194	USP10	0.254588142	0.027073782
ENSG00000169180	XPO6	0.239426151	0.027107494
ENSG00000130147	SH3BP4	-0.935306494	0.027118314
ENSG00000146409	SLC18B1	0.447270242	0.027179779
ENSG00000198171	DDRGK1	0.275723553	0.027206792
ENSG00000058804	NDC1	-0.383462833	0.027260404
ENSG00000214765	SEPT7P2	-0.373209107	0.027260404
ENSG00000117569	PTBP2	-0.45304669	0.027388161
ENSG00000167615	LENG8	-0.37100811	0.027439796
ENSG00000181074	OR52N4	1.625211081	0.027440053
ENSG00000113070	HBEGF	-1.285977231	0.027622845
ENSG00000148835	TAF5	0.492318204	0.027707634
ENSG00000255135		-0.76442528	0.027749908

ENSG00000214548	MEG3	-1.520985253	0.027749908
ENSG00000198886	MT-ND4	-0.379259049	0.027771769
ENSG00000130810	PPAN	-0.436886618	0.027786488
ENSG00000151116	UEVLD	0.453162667	0.027788711
ENSG00000171067	C11orf24	0.42830543	0.027788711
ENSG00000123104	ITPR2	0.358527349	0.027817322
ENSG00000136867	SLC31A2	0.634688867	0.027827955
ENSG00000152990	ADGRA3	-1.063321341	0.027862907
ENSG00000174945	AMZ1	-1.348541781	0.027862907
ENSG00000062716	VMP1	0.469120456	0.027862907
ENSG00000184293	CLECL1	1.133070863	0.027882627
ENSG00000112167	SAYSD1	0.357578897	0.0278962
ENSG00000180509	KCNE1	0.571797184	0.0278962
ENSG00000224531	SMIM13	-0.352099272	0.027912282
ENSG00000169313	P2RY12	1.546173895	0.02793253
ENSG00000169660	HEXDC	0.606761164	0.027966911
ENSG00000127526	SLC35E1	-0.322145481	0.027988018
ENSG00000053501	USE1	-0.313111258	0.028000792
ENSG00000157303	SUSD3	0.579862795	0.028110889
ENSG00000141699	FAM134C	0.430435057	0.028193106
ENSG00000163626	COX18	0.405050345	0.028216019
ENSG00000160446	ZDHHC12	0.315742373	0.028228875
ENSG00000196236	XPNPEP3	-0.371069213	0.028280139
ENSG00000204852	TCTN1	-0.511456294	0.028366996
ENSG00000198146	ZNF770	0.427674987	0.028387673
ENSG00000225978	HAR1A	-1.665351888	0.028387673
ENSG00000014138	POLA2	0.60777428	0.028406156
ENSG00000092148	HECTD1	-0.245694951	0.028406156
ENSG00000176049	JAKMIP2	-1.474742022	0.028409472
ENSG00000131018	SYNE1	0.454234127	0.028409472
ENSG00000146700	SSC4D	0.86910199	0.028409472
ENSG00000198730	CTR9	0.377058708	0.028409472
ENSG00000104164	BLOC1S6	-0.305417901	0.02847244
ENSG00000228956	SATB1-AS1	-1.028981217	0.028500066
ENSG00000234742		-0.784120689	0.028540322
ENSG00000273253		-0.649806281	0.028601951
ENSG00000257122	RRN3P3	-0.318107289	0.028605169
ENSG00000143554	SLC27A3	0.539087206	0.028616749
ENSG00000204272	LINC01420	-0.362599758	0.028616749
ENSG00000126005	MMP24-AS1	-0.3926302	0.028619222
ENSG00000189067	LITAF	-0.525767201	0.028670541
ENSG00000255150	EID3	-0.621062512	0.028684785
ENSG00000175283	DOLK	0.516196795	0.028746308
ENSG00000203879	GDI1	-0.250422903	0.02878944
ENSG00000010539	ZNF200	0.523783775	0.028795508
ENSG00000226287	TMEM191A	-0.7146285	0.028795508
ENSG00000183726	TMEM50A	0.283975219	0.028803055
ENSG00000112511	PHF1	-0.482948806	0.028828246

ENSG00000099365	STX1B	-0.871980215	0.028840055
ENSG00000257335	MGAM	1.073260963	0.028926551
ENSG00000111012	CYP27B1	-1.305897315	0.028947675
ENSG00000145979	TBC1D7	-0.604497722	0.028993276
ENSG00000123595	RAB9A	0.448067865	0.028993276
ENSG00000140694	PARN	0.254625456	0.028993276
ENSG00000073331	ALPK1	0.434869616	0.02903456
ENSG00000124785	NRN1	1.550322669	0.029077178
ENSG00000107643	MAPK8	-0.405130527	0.029077178
ENSG00000170191	NANP	-0.620072359	0.029077178
ENSG00000176915	ANKLE2	-0.272000226	0.029197116
ENSG00000110711	AIP	0.299719925	0.029229464
ENSG00000182149	IST1	-0.337666916	0.029296897
ENSG00000000457	SCYL3	0.577764804	0.029355785
ENSG00000115904	SOS1	0.536716466	0.029557764
ENSG00000172985	SH3RF3	-1.336202585	0.029695977
ENSG00000103196	CRISPLD2	0.700349816	0.029695977
ENSG00000187474	FPR3	1.447497325	0.029695977
ENSG00000101544	ADNP2	-0.749776404	0.029719861
ENSG00000107175	CREB3	0.396513403	0.029720362
ENSG00000026508	CD44	-0.339167412	0.029752372
ENSG00000237772		-0.79425688	0.029805361
ENSG00000128000	ZNF780B	0.779555945	0.029819982
ENSG00000109220	CHIC2	0.300941188	0.029990526
ENSG00000101811	CSTF2	0.453972316	0.029990526
ENSG00000198742	SMURF1	-0.61736846	0.030028506
ENSG00000144659	SLC25A38	-0.334018759	0.030066473
ENSG00000120705	ETF1	-0.688634342	0.030066473
ENSG00000204390	HSPA1L	0.748184995	0.030066473
ENSG00000127418	FGFRL1	-0.624712415	0.030121367
ENSG00000095574	IKZF5	-0.430843465	0.030121367
ENSG00000133935	C14orf1	0.445356615	0.030160124
ENSG00000229692	SOS1-IT1	1.074741804	0.030166
ENSG00000082213	C5orf22	0.485518004	0.030175761
ENSG00000198786	MT-ND5	-0.446921678	0.030175761
ENSG00000225830	ERCC6	-0.391245406	0.030201238
ENSG00000159403	C1R	-0.678303518	0.030207922
ENSG00000100490	CDKL1	0.706669142	0.030239253
ENSG00000198416	ZNF658B	1.263909834	0.030278931
ENSG00000119950	MXI1	-0.583828018	0.030302298
ENSG00000278727		0.704429101	0.030470559
ENSG00000102096	PIM2	-0.496537412	0.03051267
ENSG00000164877	MICALL2	-0.693862265	0.030543444
ENSG00000182979	MTA1	-0.221859244	0.030543444
ENSG00000151690	MFSD6	-0.362764016	0.030646186
ENSG00000135960	EDAR	-2.453701799	0.030682095
ENSG00000152683	SLC30A6	0.388203707	0.030711068
ENSG00000091527	CDV3	-0.272773725	0.030743659

ENSG00000096696	DSP	3.034074689	0.030743659
ENSG00000214063	TSPAN4	-0.516570707	0.030743659
ENSG00000205937	RNPS1	-0.231990593	0.030743659
ENSG00000280987		-1.051249936	0.030864774
ENSG00000120306	CYSTM1	0.53322688	0.030864774
ENSG00000152193	RNF219	0.519221184	0.030894578
ENSG00000115414	FN1	-2.834571821	0.030998612
ENSG00000123243	ITIH5	-0.613063205	0.030998612
ENSG00000125457	MIF4GD	0.257533342	0.031009977
ENSG00000128383	APOBEC3A	1.35979118	0.031125722
ENSG00000090447	TFAP4	-0.521785565	0.031224586
ENSG00000136040	PLXNC1	0.435314751	0.031228312
ENSG00000124571	XPO5	0.296271303	0.031244354
ENSG00000105609	LILRB5	0.527959495	0.031244354
ENSG00000117298	ECE1	0.336115966	0.031340187
ENSG00000168795	ZBTB5	-0.551230062	0.031340187
ENSG00000133704	IPO8	0.359383704	0.031346058
ENSG00000162736	NCSTN	0.261857201	0.031378131
ENSG00000145241	CENPC	-0.379886934	0.031433789
ENSG00000260966		-0.507962462	0.031457041
ENSG00000169860	P2RY1	-0.889190916	0.03147335
ENSG00000108788	MLX	0.346100545	0.03149664
ENSG00000101782	RIOK3	-0.355006561	0.031566207
ENSG00000096746	HNRNPH3	-0.300106202	0.031580246
ENSG00000264083		-0.498308093	0.031580246
ENSG00000249855	EEF1A1P19	-0.474554493	0.031599581
ENSG00000014919	COX15	0.536248365	0.031639168
ENSG00000271857		-1.644194035	0.031748889
ENSG00000066697	MSANTD3	-0.595873076	0.031748889
ENSG00000173349	SFT2D3	-0.410657097	0.03183109
ENSG00000166902	MRPL16	0.40260536	0.031855528
ENSG00000163930	BAP1	-0.25849132	0.03194476
ENSG00000272599		-0.878486036	0.03194476
ENSG00000266865		-0.564245685	0.031986649
ENSG00000180155	LYNX1	-0.825777428	0.032034376
ENSG00000069431	ABCC9	-0.516577769	0.032034376
ENSG00000066827	ZFAT	-1.406289372	0.032044384
ENSG00000141446	ESCO1	-0.30876805	0.032044384
ENSG00000198736	MSRB1	0.634456685	0.032056777
ENSG00000249915	PDCD6	0.313889614	0.032064611
ENSG00000124613	ZNF391	1.230859204	0.032064611
ENSG00000139178	C1RL	0.468290567	0.032064611
ENSG00000182324	KCNJ14	-0.830595214	0.032064611
ENSG00000182885	ADGRG3	-2.408081847	0.032133978
ENSG00000143190	POU2F1	-0.362145787	0.032135943
ENSG00000108175	ZMIZ1	-0.602906192	0.032135943
ENSG00000279430		-0.719495296	0.032164286
ENSG00000143702	CEP170	-0.351424507	0.032164286

ENSG00000225470	JPX	0.392503264	0.032164286
ENSG00000214517	PPME1	0.275012301	0.032164286
ENSG00000127311	HELB	0.418284283	0.032164286
ENSG00000261609	GAN	-0.333675452	0.032164286
ENSG00000162747	FCGR3B	1.316085001	0.032244992
ENSG00000068745	IP6K2	-0.242133281	0.032381398
ENSG00000182545	RNASE10	-0.879565137	0.032381398
ENSG00000196131	VN1R2	-0.941433439	0.032408689
ENSG00000163634	THOC7	0.314951738	0.032419453
ENSG00000163608	NEPRO	-0.275192484	0.032419453
ENSG00000107929	LARP4B	0.28600014	0.032419453
ENSG00000123200	ZC3H13	0.34482021	0.032419453
ENSG00000131473	ACLY	0.398885569	0.032444892
ENSG00000084073	ZMPSTE24	0.355223603	0.032485428
ENSG00000275202		-1.066937809	0.032529353
ENSG00000126001	CEP250	0.430556094	0.032698585
ENSG00000213420	GPC2	-0.815422857	0.032746985
ENSG00000092098	RNF31	0.342545948	0.032746985
ENSG00000103160	HSDL1	0.2871398	0.032746985
ENSG00000176783	RUFY1	0.260402364	0.032763325
ENSG00000180902	D2HGDH	-0.517436769	0.032777429
ENSG00000173208	ABCD2	-2.048978396	0.032777922
ENSG00000099250	NRP1	-0.943257226	0.032899142
ENSG00000113522	RAD50	0.399483748	0.03298804
ENSG00000082898	XPO1	0.326361107	0.033008491
ENSG00000051009	FAM160A2	0.357009297	0.033140632
ENSG00000123545	NDUFAF4	0.471512543	0.033188596
ENSG00000205534	SMG1P2	-0.635590571	0.03321358
ENSG00000163867	ZMYM6	-0.272634173	0.033381118
ENSG00000154305	MIA3	0.337401862	0.033381118
ENSG00000163539	CLASP2	-0.292578538	0.033381118
ENSG00000101844	ATG4A	0.372912658	0.033381118
ENSG00000267655		-0.968572962	0.033381118
ENSG00000151414	NEK7	0.378588385	0.033389883
ENSG00000103248	MTHFSD	0.444377192	0.03339768
ENSG00000243199		-0.536425096	0.033481638
ENSG00000180104	EXOC3	-0.234564915	0.03350296
ENSG00000204370	SDHD	0.381418429	0.03350296
ENSG00000274712		-0.53279755	0.03350296
ENSG00000135776	ABCB10	0.272773132	0.033522356
ENSG00000197258	EIF4BP6	-0.528029314	0.033522356
ENSG00000105856	HBP1	-0.490712686	0.033530528
ENSG00000107816	LZTS2	-0.510887076	0.033530528
ENSG00000198538	ZNF28	0.6917002	0.033530528
ENSG00000258727		-0.385686767	0.033551592
ENSG00000173846	PLK3	-0.725348799	0.033729844
ENSG00000092964	DPYSL2	0.734380955	0.03376967
ENSG00000154277	UCHL1	-2.751269243	0.03380761

ENSG00000173276	ZBTB21	-0.855492864	0.033811221
ENSG00000237775	DDR1-AS1	-0.729743869	0.033849878
ENSG00000137944	KYAT3	0.295793013	0.033864072
ENSG00000260060		-0.84288095	0.033876747
ENSG00000078618	NRDC	0.326231169	0.033910719
ENSG00000267281		-0.532362826	0.033910719
ENSG00000168477	TNXB	-0.81635374	0.033966612
ENSG00000136436	CALCOCO2	0.260044474	0.034006362
ENSG00000124151	NCOA3	-0.281400933	0.034006362
ENSG00000100221	JOSD1	-0.750025401	0.034046551
ENSG00000143811	PYCR2	0.441056012	0.034244687
ENSG00000213949	ITGA1	0.959570611	0.034261679
ENSG00000162927	PUS10	0.476444226	0.034276043
ENSG00000079950	STX7	0.526197441	0.034311451
ENSG00000143797	MBOAT2	-0.584851923	0.034457498
ENSG00000234506	LINC01506	0.721442219	0.034457498
ENSG00000170004	CHD3	-0.307720213	0.034457498
ENSG00000114023	FAM162A	-0.379082121	0.034463698
ENSG00000170485	NPAS2	-1.890029621	0.034483417
ENSG00000174137	FAM53A	0.77783232	0.034507504
ENSG00000105676	ARMC6	0.456727863	0.034514072
ENSG00000233426	EIF3FP3	-0.563626121	0.03451472
ENSG00000204444	APOM	-0.663761701	0.034547939
ENSG00000223496	EXOSC6	-0.395383401	0.034585486
ENSG00000141510	TP53	0.438092183	0.034585486
ENSG00000167862	MRPL58	0.430181856	0.034634626
ENSG00000113648	H2AFY	-0.26271846	0.034654127
ENSG00000165650	PDZD8	-0.432950607	0.034667086
ENSG00000080845	DLGAP4	0.318070336	0.034667086
ENSG00000131188	PRR7	-0.660548268	0.03471395
ENSG00000132017	DCAF15	-0.23660626	0.03471395
ENSG00000144029	MRPS5	0.373180698	0.034770812
ENSG00000010818	HIVEP2	0.422645257	0.034770812
ENSG00000173480	ZNF417	-0.359425575	0.034770812
ENSG00000273247		0.464835015	0.034793804
ENSG00000232022	FAAHP1	-1.3560911	0.034796865
ENSG00000180871	CXCR2	0.991782402	0.034819374
ENSG00000197620	CXorf40A	-0.635073339	0.034825891
ENSG00000130939	UBE4B	-0.189201101	0.034841365
ENSG00000106608	URGCP	-0.348512793	0.034930722
ENSG00000028839	TBPL1	0.297510227	0.035018789
ENSG00000135953	MFSD9	0.352192635	0.03502022
ENSG00000198727	MT-CYB	-0.42811006	0.03502022
ENSG00000091106	NLRC4	1.224301418	0.035023656
ENSG00000275400		-0.553786121	0.035026948
ENSG00000083457	ITGAE	-0.455838291	0.035026948
ENSG00000105556	MIER2	-0.274903286	0.035026948
ENSG00000259071		0.815400535	0.03505975

ENSG00000206560	ANKRD28	-0.704533634	0.035088987
ENSG00000153898	MCOLN2	0.833384175	0.035093441
ENSG00000196850	PPTC7	-0.275245906	0.035093441
ENSG00000170310	STX8	0.385711337	0.035103278
ENSG00000156531	PHF6	0.512954681	0.035200652
ENSG00000182985	CADM1	-1.874173353	0.035217779
ENSG00000109854	HTATIP2	0.404781777	0.035266431
ENSG00000247982	LINC00926	-0.805296695	0.035437367
ENSG00000198064	NPIPB13	-1.082238996	0.035443246
ENSG00000119203	CPSF3	0.365426479	0.035478434
ENSG00000131016	AKAP12	-1.700567064	0.035480675
ENSG00000260196		-1.284752133	0.035530483
ENSG00000148925	BTBD10	0.34361637	0.035608937
ENSG00000075568	TMEM131	0.243709744	0.035658151
ENSG00000146457	WTAP	-0.425833086	0.035658151
ENSG00000254528		1.037673527	0.035675321
ENSG00000148110	MFSD14B	0.423307197	0.035861701
ENSG00000170734	POLH	0.691014048	0.035898215
ENSG00000142544	CTU1	0.803372039	0.035898215
ENSG00000124486	USP9X	-0.340071551	0.035906046
ENSG00000090097	PCBP4	-0.704864045	0.035993517
ENSG00000152219	ARL14EP	0.40207731	0.035993517
ENSG00000162600	OMA1	0.507531477	0.036009694
ENSG00000231259		-0.548259879	0.036009694
ENSG00000186230	ZNF749	-0.711976209	0.036009694
ENSG00000064102	ASUN	0.393888645	0.036038849
ENSG00000231312		-0.664786088	0.03612226
ENSG00000164039	BDH2	0.510593212	0.03612226
ENSG00000187837	HIST1H1C	0.609776145	0.03612226
ENSG00000152240	HAUS1	0.461742977	0.03612226
ENSG00000185989	RASA3	-0.321502352	0.03612692
ENSG00000263266	RPS7P1	-0.346779398	0.03612692
ENSG00000204524	ZNF805	-0.541040595	0.03612692
ENSG00000147650	LRP12	-1.007822987	0.036132086
ENSG00000178966	RMI1	0.749174568	0.036132086
ENSG00000122970	IFT81	-0.52266654	0.036140154
ENSG00000255026		-1.658459218	0.036210494
ENSG00000148300	REXO4	0.332655646	0.036266311
ENSG00000163788	SNRK	-0.335001598	0.036278497
ENSG00000132589	FLOT2	0.415491164	0.036285974
ENSG00000002919	SNX11	0.470320926	0.036296613
ENSG00000132613	MTSS1L	-0.810139966	0.036350904
ENSG00000151576	QTRT2	-0.264858292	0.036403749
ENSG00000118985	ELL2	-1.187754745	0.036445232
ENSG00000163602	RYBP	-0.485028572	0.036525391
ENSG00000013725	CD6	-1.121645157	0.036590092
ENSG00000185650	ZFP36L1	-0.447460785	0.036590092
ENSG00000169246	NPIPB3	-0.521782738	0.036590092

ENSG00000154370	TRIM11	-0.286416628	0.03662827
ENSG00000183696	UPP1	-0.556061099	0.03662827
ENSG00000198168	SVIP	-0.502546133	0.03662827
ENSG00000168003	SLC3A2	-0.27871577	0.03662827
ENSG00000257017	HP	1.308981432	0.03662827
ENSG00000274370		-0.975292888	0.03662827
ENSG00000134769	DTNA	-0.913840759	0.036714672
ENSG00000135124	P2RX4	-0.509625459	0.036718554
ENSG00000161970	RPL26	-0.289614205	0.036718554
ENSG00000110063	DCPS	0.58696357	0.036721391
ENSG00000131732	ZCCHC9	0.293512874	0.0367471
ENSG00000173198	CYSLTR1	0.662927335	0.036876696
ENSG00000137269	LRRC1	0.676033089	0.036886105
ENSG00000115942	ORC2	0.53291029	0.036889902
ENSG00000115525	ST3GAL5	0.389047629	0.036943369
ENSG00000153250	RBMS1	0.288370037	0.036943369
ENSG00000226221	RPL26P19	-0.5273178	0.036943369
ENSG00000268069		-0.924631665	0.037012861
ENSG00000060642	PIGV	0.561898722	0.037079092
ENSG00000197557	TTC30A	0.711222383	0.037085677
ENSG00000083896	YTHDC1	-0.411977645	0.037085677
ENSG00000176248	ANAPC2	-0.277175863	0.037180211
ENSG00000173085	COQ2	0.391570205	0.037186513
ENSG00000253958	CLDN23	0.806079368	0.037186513
ENSG00000171169	NAIF1	0.363423337	0.037186513
ENSG00000110700	RPS13	-0.264627202	0.037186513
ENSG00000198879	SFMBT2	-0.589242124	0.037186513
ENSG00000183889		-1.069073754	0.037186513
ENSG00000152104	PTPN14	-0.619498542	0.037219728
ENSG00000272462		-0.532388084	0.037223404
ENSG00000138002	IFT172	-0.453193509	0.037275033
ENSG00000027847	B4GALT7	-0.281002539	0.037275033
ENSG00000149218	ENDOD1	0.54207209	0.037275033
ENSG00000126003	PLAGL2	0.326944029	0.037281579
ENSG00000216490	IFI30	-0.813367007	0.037284405
ENSG00000196705	ZNF431	-0.328650873	0.037284405
ENSG00000180370	PAK2	0.230585814	0.03733814
ENSG00000260257		-0.469113636	0.03733814
ENSG00000173762	CD7	-0.56245249	0.037553944
ENSG00000198715	GLMP	0.382378831	0.037575294
ENSG00000131368	MRPS25	-0.336828733	0.037832636
ENSG00000177383	MAGEF1	0.706738085	0.037832636
ENSG00000163947	ARHGEF3	0.547514663	0.037863261
ENSG00000080603	SRCAP	-0.398507369	0.037886148
ENSG00000134086	VHL	-0.262732875	0.037905577
ENSG00000170629	DPY19L2P2	-0.598018769	0.037905577
ENSG00000108100	CCNY	-0.263992683	0.037909922
ENSG00000227500	SCAMP4	-0.342359009	0.037923236

ENSG00000183741	CBX6	-0.370634398	0.037923236
ENSG00000153214	TMEM87B	0.397705533	0.038008573
ENSG00000156127	BATF	0.654899607	0.038008573
ENSG00000254911	SCARNA9	-0.592535024	0.038058197
ENSG00000140299	BNIP2	-0.285808905	0.038058197
ENSG00000130559	CAMSAP1	-0.305531077	0.038070276
ENSG00000177683	THAP5	0.420175908	0.038086572
ENSG00000072121	ZFYVE26	0.267627165	0.038192743
ENSG00000146223	RPL7L1	0.296922074	0.038226689
ENSG00000104687	GSR	0.377520887	0.03831234
ENSG00000136280	CCM2	0.395854229	0.038320988
ENSG00000128891	C15orf57	0.388431126	0.038320988
ENSG00000134265	NAPG	-0.302704768	0.038320988
ENSG00000277161	PIGW	-0.489908164	0.038496064
ENSG00000158528	PPP1R9A	-2.268325607	0.038510195
ENSG00000100629	CEP128	-0.774120272	0.038525345
ENSG00000140905	GCSH	-0.879119692	0.038525345
ENSG00000091640	SPAG7	-0.230907834	0.038549601
ENSG00000173692	PSMD1	0.374898571	0.038549777
ENSG00000141480	ARRB2	-0.24534073	0.038581481
ENSG00000157551	KCNJ15	0.66988429	0.038646068
ENSG00000177706	FAM20C	-1.202401645	0.038707361
ENSG00000226396		-0.655417463	0.03872307
ENSG00000163219	ARHGAP25	0.354671017	0.038768863
ENSG00000121310	ECHDC2	0.583513361	0.038824436
ENSG00000138286	FAM149B1	0.316611707	0.038824436
ENSG00000224614	TNK2-AS1	0.73618491	0.03904766
ENSG00000129219	PLD2	-0.370666711	0.03904766
ENSG00000108963	DPH1	-0.316337831	0.039087445
ENSG00000068120	COASY	0.322307976	0.039087445
ENSG00000187210	GCNT1	0.960829139	0.039130579
ENSG00000256262	USP30-AS1	0.64364203	0.039130579
ENSG00000162972	MAIP1	0.424773266	0.039155126
ENSG00000101310	SEC23B	0.359781143	0.039220051
ENSG00000069712	KIAA1107	0.586408918	0.03927648
ENSG00000198435	NRARP	-1.654212686	0.039316824
ENSG00000085788	DDHD2	-0.281584402	0.039357484
ENSG00000133895	MEN1	0.338780617	0.039440914
ENSG00000196588	MKL1	0.306818654	0.039510589
ENSG00000142686	C1orf216	0.721074747	0.039533234
ENSG00000155561	NUP205	0.252771452	0.039596997
ENSG00000278158		-1.853594391	0.039660029
ENSG00000102309	PIN4	0.437210493	0.039661773
ENSG00000211454	AKR7L	-0.95172348	0.039840362
ENSG00000160867	FGFR4	-1.141097483	0.039888239
ENSG00000114796	KLHL24	-0.416266687	0.039896402
ENSG00000068976	PYGM	-0.641340758	0.039924452
ENSG00000174718	KIAA1551	0.44710491	0.039924452

ENSG00000167258	CDK12	-0.236408477	0.039929247
ENSG00000227939	RPL3P2	-0.773690725	0.039932807
ENSG00000154429	CCSAP	-0.455182531	0.039947253
ENSG00000157837	SPPL3	-0.263162521	0.039947253
ENSG00000136463	TACO1	0.387622596	0.039979135
ENSG00000117305	HMGCL	0.470802752	0.040011381
ENSG00000104325	DECR1	0.333155619	0.040011381
ENSG00000225331		-0.762466392	0.040011381
ENSG00000172005	MAL	-1.971333026	0.040022908
ENSG00000164253	WDR41	0.388880869	0.040137917
ENSG00000135441	BLOC1S1	0.363617854	0.0401607
ENSG00000153790	C7orf31	0.36227258	0.04023191
ENSG00000185608	MRPL40	0.354022916	0.04023191
ENSG00000196912	ANKRD36B	-0.565924907	0.0403698
ENSG00000112053	SLC26A8	0.654299926	0.040380201
ENSG00000198205	ZXDA	-0.582631378	0.040386377
ENSG00000144120	TMEM177	0.981317947	0.040391303
ENSG00000161921	CXCL16	-1.165787698	0.040413343
ENSG00000227543	SPAG5-AS1	-0.853084203	0.040461498
ENSG00000002016	RAD52	0.56627894	0.040492207
ENSG00000230373	GOLGA6L5P	-1.271894843	0.040547271
ENSG00000235954	TTC28-AS1	-0.378263679	0.040568952
ENSG00000204954	C12orf73	0.596008074	0.040699447
ENSG00000108559	NUP88	0.268242492	0.040762732
ENSG00000176014	TUBB6	-1.307920894	0.040856661
ENSG00000132394	EEFSEC	0.342262871	0.040953629
ENSG00000120509	PDZD11	0.497368452	0.040953629
ENSG00000028528	SNX1	0.447987947	0.040953629
ENSG00000125319	C17orf53	-1.428027682	0.040953629
ENSG00000177369		-0.704985136	0.040953629
ENSG00000175221	MED16	0.44462848	0.040953629
ENSG00000227199	ST7-AS1	-0.841681688	0.040960335
ENSG00000105656	ELL	-0.326904548	0.0410085
ENSG00000178226	PRSS36	-0.531042414	0.041064117
ENSG00000168685	IL7R	-1.242043094	0.041087773
ENSG00000250771		0.775421951	0.041262274
ENSG00000166446	CDYL2	-0.582170322	0.041318856
ENSG00000162585	FAAP20	-0.280942397	0.041384979
ENSG00000145103	ILDR1	-1.098861341	0.041432673
ENSG00000234009	RPL5P34	-0.438800084	0.041509774
ENSG00000233476	EEF1A1P6	-0.3897842	0.041553561
ENSG00000125977	EIF2S2	0.387969379	0.041561104
ENSG00000099284	H2AFY2	0.545455547	0.041575839
ENSG00000106591	MRPL32	0.346527499	0.041611098
ENSG00000145817	YIPF5	0.33578122	0.041673088
ENSG00000172172	MRPL13	0.441746224	0.041674832
ENSG00000197324	LRP10	0.304316904	0.041674832
ENSG00000174446	SNAPC5	0.363669584	0.04169002

ENSG00000223865	HLA-DPB1	-0.688057405	0.041841627
ENSG00000116406	EDEM3	0.460639006	0.041847037
ENSG00000273356		-0.481645855	0.041847037
ENSG00000154099	DNAAF1	1.250435172	0.041847037
ENSG00000133193	FAM104A	0.317135673	0.041847037
ENSG00000108669	CYTH1	-0.198919909	0.041972874
ENSG00000280407		-1.033229289	0.042020308
ENSG00000101452	DHX35	-0.214841508	0.042276354
ENSG00000087299	L2HGDH	-0.367299609	0.042340539
ENSG00000111237	VPS29	0.283397043	0.042580232
ENSG00000032389	TSSC1	0.310229671	0.042693848
ENSG00000113013	HSPA9	-0.368502899	0.042693848
ENSG00000235863	B3GALT4	0.477679624	0.042693848
ENSG00000227741		-0.930529938	0.042696792
ENSG00000115145	STAM2	0.382387839	0.042774762
ENSG00000189007	ADAT2	-0.356462794	0.042774762
ENSG00000225541		2.249359447	0.042774762
ENSG00000175556	LONRF3	-0.918903565	0.042774762
ENSG00000275832	ARHGAP23	0.717112713	0.04279235
ENSG00000133639	BTG1	-0.460511089	0.042827912
ENSG00000198723	C19orf45	-1.567148549	0.042830057
ENSG00000105926	MPP6	-0.84294628	0.042862648
ENSG00000063177	RPL18	-0.277348455	0.042862648
ENSG00000088986	DYNLL1	0.525379326	0.042879728
ENSG00000198900	TOP1	-0.403948978	0.042889834
ENSG00000147533	GOLGA7	0.25392236	0.042949017
ENSG00000084463	WBP11	-0.380455483	0.042949017
ENSG00000196839	ADA	-0.824826609	0.04297269
ENSG00000181418	DDN	-1.099892677	0.043274246
ENSG00000143106	PSMA5	0.400959094	0.04336335
ENSG00000166503		-0.867732991	0.043396802
ENSG00000165929	TC2N	-1.428411299	0.043411892
ENSG00000226824		-0.823860615	0.043450248
ENSG00000108651	UTP6	0.34033531	0.0435784
ENSG00000196975	ANXA4	0.638797679	0.043673562
ENSG00000205560	CPT1B	0.623633115	0.043683729
ENSG00000188177	ZC3H6	0.449787696	0.043878307
ENSG00000185304	RGPD2	-1.768965475	0.043905488
ENSG00000124201	ZNFX1	0.375159117	0.04403241
ENSG00000163798	SLC4A1AP	0.301696422	0.044073242
ENSG00000278022		-0.980288454	0.044199742
ENSG00000118242	MREG	-0.617635401	0.044200879
ENSG00000279821		-1.008505044	0.044260872
ENSG00000187808	SOWAHD	1.218738067	0.044260872
ENSG00000259924		0.649498847	0.044260872
ENSG00000265681	RPL17	-0.322254002	0.044463052
ENSG00000182749	PAQR7	-0.528763541	0.044516311
ENSG00000160654	CD3G	-0.780630159	0.044516311

ENSG00000263990		-0.594817431	0.044516311
ENSG00000173114	LRRN3	-1.699094283	0.044539379
ENSG00000100095	SEZ6L	-0.938291307	0.044577054
ENSG00000139620	KANSL2	-0.462329484	0.044722008
ENSG00000100528	CNIH1	-0.248019123	0.044797941
ENSG00000156299	TIAM1	-0.509851187	0.04482844
ENSG00000012211	PRICKLE3	0.530638254	0.044855243
ENSG00000145901	TNIP1	0.301651002	0.044857373
ENSG00000160991	ORAI2	-0.3372836	0.044858279
ENSG00000114315	HES1	-1.512167804	0.044865232
ENSG00000274561		-0.587398073	0.044865232
ENSG00000121742	GJB6	-2.374130992	0.044871971
ENSG00000256039		-1.667006254	0.044874164
ENSG00000115685	PPP1R7	0.264195526	0.044926578
ENSG00000106330	MOSPD3	0.376196546	0.044933582
ENSG00000180198	RCC1	-0.472205636	0.044970514
ENSG00000174928	C3orf33	-0.682857018	0.044970514
ENSG00000134627	PIWIL4	0.444831463	0.044989199
ENSG00000139719	VPS33A	0.37666336	0.044989199
ENSG00000213213	CCDC183	-0.792931235	0.045171397
ENSG00000003402	CFLAR	0.293497243	0.045189933
ENSG00000166189	HPS6	0.708055471	0.045234117
ENSG00000162458	FBLIM1	-0.822791418	0.045286099
ENSG00000143742	SRP9	0.311417364	0.045306519
ENSG00000113649	TCERG1	-0.238592072	0.045376255
ENSG00000213714	FAM209B	-0.726170325	0.045396255
ENSG00000085832	EPS15	0.31207967	0.045516747
ENSG00000268001	CARD8-AS1	0.709165379	0.045516747
ENSG00000059588	TARBP1	-0.313006246	0.045585754
ENSG00000172878	METAP1D	-0.642964167	0.045585754
ENSG00000020129	NCDN	0.549441244	0.045627992
ENSG00000123933	MXD4	-0.356007475	0.045627992
ENSG00000168282	MGAT2	0.388705667	0.045731508
ENSG00000171806	METTL18	0.588245147	0.045745433
ENSG00000170915	PAQR8	0.932034789	0.045745433
ENSG00000086062	B4GALT1	-0.456301025	0.045745433
ENSG00000138615	CILP	-1.31464858	0.045745433
ENSG00000103528	SYT17	-0.898680906	0.045842189
ENSG00000280279		-0.505064265	0.045842189
ENSG00000273271		-0.526163077	0.045913818
ENSG00000259939		-0.92813522	0.046173337
ENSG00000176531	PHLDB3	-0.574883081	0.04619206
ENSG00000211459	MT-RNR1	-0.460542974	0.046385506
ENSG00000116106	EPHA4	-2.254713371	0.046492435
ENSG00000165775	FUNDC2	-0.236742852	0.046561541
ENSG00000150768	DLAT	0.464735805	0.046608097
ENSG00000180316	PNPLA1	0.838732404	0.046660785
ENSG00000130803	ZNF317	-0.321704863	0.046660785

ENSG00000197857	ZNF44	-0.375613915	0.046708887
ENSG00000112234	FBXL4	0.36697866	0.046778011
ENSG00000067221	STOML1	0.421421147	0.046778011
ENSG00000133134	BEX2	-1.149672845	0.046794426
ENSG00000136854	STXBP1	-1.050364583	0.046867269
ENSG00000179331	RAB39A	0.66645974	0.046867269
ENSG00000182899	RPL35A	-0.272608879	0.04690721
ENSG00000070770	CSNK2A2	-0.21445563	0.047047533
ENSG00000155329	ZCCHC10	0.303668517	0.047095574
ENSG00000279908		-0.943357879	0.047109696
ENSG00000247679		-0.506079651	0.047109696
ENSG00000146904	EPHA1	0.838391387	0.047109696
ENSG00000140525	FANCI	-0.777778724	0.047109696
ENSG00000152795	HNRNPDL	-0.203319536	0.047181915
ENSG00000145050	MANF	0.546621078	0.047198457
ENSG00000057019	DCBLD2	-0.772443534	0.047204011
ENSG00000170017	ALCAM	-0.588982674	0.047204011
ENSG00000130985	UBA1	0.245383755	0.047204011
ENSG00000172732	MUS81	0.357585623	0.047219096
ENSG00000165424	ZCCHC24	-0.467881975	0.047235937
ENSG00000119537	KDSR	-0.364971925	0.0472644
ENSG00000102879	CORO1A	0.316893985	0.047275306
ENSG00000066294	CD84	-0.603034975	0.047291975
ENSG00000177917	ARL6IP6	0.371794566	0.047334376
ENSG00000079459	FDFT1	0.397234506	0.047334376
ENSG00000251682		-0.736975765	0.047349932
ENSG00000117280	RAB29	0.405213701	0.047360416
ENSG00000104973	MED25	0.309300426	0.047375201
ENSG00000143774	GUK1	-0.28575131	0.047378261
ENSG00000132792	CTNNBL1	0.222409877	0.047412235
ENSG00000064490	RFXANK	0.255588988	0.047412235
ENSG00000100591	AHSA1	0.475421296	0.047453672
ENSG00000152767	FARP1	-0.543825983	0.047576994
ENSG00000276107		-1.900576921	0.047614533
ENSG00000107263	RAPGEF1	-0.433751587	0.047666734
ENSG00000120915	EPHX2	-1.489241779	0.047757715
ENSG00000163519	TRAT1	-1.497079091	0.047814511
ENSG00000185621	LMLN	-0.497515298	0.047823476
ENSG00000157764	BRAF	-0.368742043	0.047831449
ENSG00000204267	TAP2	0.419002327	0.047834583
ENSG00000005889	ZFX	-0.254474425	0.047835616
ENSG00000161021	MAML1	0.256537412	0.047835798
ENSG00000126266	FFAR1	-1.47947673	0.047922337
ENSG00000177663	IL17RA	0.371892279	0.048013504
ENSG00000101843	PSMD10	0.335532476	0.048090088
ENSG00000251143		-0.482046572	0.048097795
ENSG00000198700	IPO9	-0.210696739	0.048327272
ENSG00000103544	C16orf62	0.282296215	0.048350537

ENSG00000224546	EIF4BP3	-0.86005885	0.048355103
ENSG00000263823		-0.711279753	0.04835595
ENSG00000091622	PITPNM3	-0.9075971	0.048766873
ENSG00000169188	APEX2	0.367235417	0.048850196
ENSG00000076650	GPATCH1	0.445153009	0.048850196
ENSG00000234719	NPIPB2	-0.56032154	0.048944245
ENSG00000164077	MON1A	0.673529645	0.048984921
ENSG00000118507	AKAP7	0.454734326	0.048984921
ENSG00000188021	UBQLN2	0.299783638	0.048984921
ENSG00000183628	DGCR6	-0.533488766	0.048984921
ENSG00000102786	INTS6	0.289793148	0.048987491
ENSG00000181638	ZFP41	0.585588698	0.048994559
ENSG00000171311	EXOSC1	0.338695053	0.048994559
ENSG00000264112		-0.530351163	0.049155104
ENSG00000166068	SPRED1	-0.756548733	0.049207196
ENSG00000142208	AKT1	-0.197456006	0.049330083
ENSG00000167863	ATP5H	0.265483745	0.049392328
ENSG00000271122		0.549068993	0.049420702
ENSG00000274349	ZNF658	0.877733289	0.049443985
ENSG00000040531	CTNS	0.315283048	0.049443985
ENSG00000137054	POLR1E	-0.355078721	0.049445463
ENSG00000040608	RTN4R	-2.251146231	0.049445463
ENSG00000112304	ACOT13	0.540197293	0.049568218
ENSG00000120526	NUDCD1	0.527432194	0.049585109
ENSG00000157353	FUK	0.264948418	0.049662752
ENSG00000114316	USP4	-0.186229097	0.049723752
ENSG00000137824	RMDN3	-0.230261014	0.04972421
ENSG00000165512	ZNF22	0.380158705	0.049731719
ENSG00000087087	SRRT	-0.245257146	0.049888954
ENSG00000117153	KLHL12	0.255408858	0.049966643
